# A grounded theory approach to understanding in-game goods purchase

**DOI:** 10.1371/journal.pone.0262998

**Published:** 2022-01-27

**Authors:** Xiaowei Cai, Javier Cebollada, Mónica Cortiñas

**Affiliations:** 1 Institute for Advanced Research in Business and Economics (INARBE), Public University of Navarre (UPNA), Pamplona, Spain; 2 Department of Business Management, Public University of Navarre (UPNA), Pamplona, Spain; West Pomeranian University of Technology, POLAND

## Abstract

Video game companies are increasingly diversifying their profit models. Rather than relying exclusively on the sale of video game titles or the subscription model, video game companies are maximising the revenues and extending the lifecycle of their games by means of a strategy based on the sale of in-game goods. This study contributes to the theory on in-game goods purchases by explaining why and how video game players purchase different types of in-game goods. We used an inductive approach involving qualitative data analysis based on grounded theory. Six types of in-game goods are grouped into three categories: functional-based goods, probability-based goods, and ornamental-based goods. After acknowledging the heterogeneity of the categories, a conceptual framework is developed by conducting 21 in-depth interviews, from which it emerges that players purchase functional-based goods, probability-based goods, and ornamental-based goods for different motives and through the different behavioural processes. First, the purchase of functional-based goods is a strategy for entering the flow experience. Second, the purchase of probability-based goods is a compromise for purchase restrictions. Third, the purchase of ornamental goods is driven by the synergism of intrinsic motivations and exposure in the virtual world. Therefore, video game researchers should not treat in-game goods as a homogeneous concept. The findings also suggest that it is critically important for video game developers to strike a balance between the challenges of the gameplay and the skills of players because excessively raising (or lowering) the level of difficulty could pose a threat to the company’s sustainable profit.

## 1. Introduction

We are in an era of rapid development in the video game industry. Since the emergence of video games in the 1970s, the game market took over more than 35 years to grow into a 35 billion U.S. dollar business in 2007 [1]. However, 137.9 billion U.S. dollars were generated in 2018, which means that the 100 billion additional value was created in only 11 years [1].

Along with the growth of the market, video game is constantly evolving. For a long time, the business model in the videogame industry was very traditional. A typical traditional business model is assigning a fixed price for the game title and allowing players unlimited playtime [2]. However, in recent years, the new trend in the videogame industry is the freemium business model. The freemium business model offers players the chance to play the core game content for free, but the profit is generated through selling in-game goods and premium services [3]. With the advent of the freemium business model, where the economic cost of adopting a game is steadily decreasing, post-adoption behaviour is becoming increasingly important. Against this background, understanding why and how players purchase in-game goods is a pertinent practical issue for videogame companies [4].

The concept of in-game goods is derived from that of virtual goods, which may be defined as digital objects that commonly exist within virtual economies, such as videogames and virtual worlds, including but not limited to characters, avatar clothing, weapons, furniture, and tokens [4,5]. In this study, we define in-game goods as virtual goods in the videogame context.

There has been a clear increase in academic studies investigating purchase motivations of in-game goods during the last decade [6]. Researchers have approached this issue from both qualitative [7–12] and quantitative perspectives [6,13–26].

Although the research on in-game purchase motivations has developed rapidly in recent years, several gaps and unsolved problems remain.

First, the theoretical foundation of the studies of in-game goods purchase behaviour is unclear and confounded. Hamari and Keronen (2016) [4] reviewed 30 articles about virtual goods purchase and found that a considerable number of studies (n = 12) ‘failed to specify any clear theoretical foundation or simply selected a range of variables from different theoretical frameworks. Among the studies with a theoretical framework (n = 18), the technology acceptance model (TAM) was most used, followed by the stimulus organism response model, the theory of planned behaviour, the expectancy disconfirmation model, the unified theory of acceptance and use of technology (UTAUT), the transaction cost theory, the theory of consumption values, the virtual experiential marketing, the customer value theory, the self-presentation theory, and the social capital theory. However, none of the mentioned theories is developed in the context of in-game goods purchase. Transplanting a theory from the context where it is developed to a totally new context may reduce its explanatory power. Forcing theories from other contexts into studies of in-game goods purchase is a widespread phenomenon. For instance, the UTAUT has been used in some studies of in-game goods purchase [24,27]. However, the original theory of UTAUT [28] was developed to explain the acceptance of new technology instead of the purchase of virtual goods. We suspect that this is due to the lack of specific theoretical foundations for the video game context when addressing issues relating to in-game goods purchase behaviour.

Second, not all in-game goods are the same [5]. Industrial practitioners have identified different types of in-game goods, including *power-ups*, *expansion packages*, *playable characters*, *cosmetics/skins*, *loot boxes*, and *time-savers* [29]. Researchers, meanwhile, have so far classified in-game goods according to their different functionality, including functional-based goods [10], ornamental-based goods [10], and probability-based goods [30]. However, researchers have not yet determined whether the motivations identified so far affect the purchase of all or only certain types of in-game goods. Since there is a noticeable difference between different types of in-game goods, their associated purchase motives can also vary [5]. For instance, although flow experience (discussed in more detail in the following section) has been identified as a factor that is positively associated with the in-game purchase [5,8,9,12], it is not yet known whether this factor is a common driver of purchase behaviour in relation to all or only some types of in-game goods.

Acknowledging the research gaps, we propose our research questions:

- Why do video game players purchase different types of in-game goods?
- How do video game players purchase different types of in-game goods?

This study aims to establish a conceptual framework to explain videogame players’ purchase behaviour in relation to different types of in-game goods. An inductive qualitative approach, grounded theory [31–33], is used in this study. Grounded theory has been used to study social processes or actions and explain why things happen [32], which corresponds the aim of our study.

The results of this research are expected to provide video games researchers with a theoretical foundation for the quantitative analysis of players’ purchase behaviour in relation to in-game goods. It will also serve as a reference for practitioners in the videogame industry, as the excerpts from the informants may offer novel insights for game designers and marketing managers to improve the game mechanism and marketing practices.

This study is structured as follows. In the second section, we review the literature on in-game goods typology, players’ purchase motivations, and their subsequent purchase processes. Then, in the third section, we introduce the methodology of this study and its procedure. The research findings are presented in the fourth section, and the results are discussed in the fifth section. The last section indicates the limitations of this research and suggests future research directions.

## 2. Literature review

### 2.1. In-game goods typology

The term “in-game goods” is a general term that includes different types of virtual goods within video games. Video game researchers have assigned in-game goods to different categories according to distinct criteria.

Academic researchers have assigned in-game goods into two categories according to their functionality [10,34]: functional-based goods and ornamental-based goods. Functional-based goods are in-game items that can enhance players’ performance (numerical advantages) and functionality (new abilities and options) [10]. Ornamental-based goods are aesthetic, non-functional in-game items in games enabling players to create and communicate social distinctions and bonds. [10].

According to Jaeyoung Lee, Suh, Park, and Lee (2018) [30], in-game goods can be classified into two categories: probability-based and non-probability-based in-game goods. The main difference between probability-based in-game goods and non-probability-based in-game goods is the predictability of their expected value [30]. While the value of non-probability-based in-game goods is equal to the amount of money paid, the value of probability-based in-game goods can be either greater or smaller than the amount paid [30]. Probability-based in-game goods receive different names in different games and contexts, such as *Loot boxes*, *Card package*, *Gashapon machine*, and so on. In this paper, we use the term Loot boxes to refer to probability-based in-game goods.

Beyond the academic world, industry practice has traditionally classified in-game goods into six categories, namely, *power-ups*, *expansion packages*, *playable characters*, *cosmetics/skins*, *loot boxes*, and *time-savers* [29]. Power-ups are functional items that instantly enhance the gaming experience. For example, players can purchase diverse power-ups items (e.g. *power snow*, which freezes zombies) as a last-ditch effort when they face an overwhelming situation. Secondly, expansions are the extra story and new gaming mechanisms for an already released video game. For example, The *Fate of Atlantis* is an expansion of the videogame *Assassin’s Creed Odyssey*, which sets players against mythical creatures as they uncover the mysteries of the fabled sunken city of Atlantis. Thirdly, playable characters are functional characters that cannot be acquired in videogame through any gaming mechanism and necessarily involve spending real money. For instance, in *DEAD OR ALIVE 6*: *Core Fighters*, players start with a very limited number of characters but are able to purchase extra characters with real money. Fourth, cosmetics/skins are the non-functional items in video games that affect only the aesthetic appearance of in-game elements, such as characters and interface. For instance, *Dead or Alive 5 Last Round* is famous for having about 1130 costumes for its 36 characters, although most have to be paid for. Fifth, loot boxes function like a lucky draw since players do not know what is inside until they are opened. For example, players of *Hearthstone* are able to purchase card packages, and after unpacking them, players will receive five determined cards without knowing precisely which five cards they will be. Finally, time-savers are the in-game goods that enable players instant access to items that would require great effort to obtain through its original gaming mechanism. For instance, players of *Resident Evil 2* (2019 version) can purchase a key to unlock all the hidden features, which would be extremely challenging to acquire without paying real money.

Table 1 depicts the relationship between industry practice and a range of academic criteria, based on which we have classified in-game goods into three types: functional-based goods, ornamental-based goods, and probability-based goods. In the first place, while the narrow definition of functional-based goods [34] refers exclusively to *power-ups*, we include other three types of in-game goods, *expansion packages*, *playable characters*, and *time-savers*, in this concept according to the board definition of functional-based goods [10]. In the second place, ornamental-based goods correspond to the *cosmetics/Skins*, which is in line with the previous definitions of this concept [10,34,35]. In the third place, probability-based goods refer to loot boxes, which is consistent with the previous definition of this concept [30].

**Table 1:** Classification of in-game goods.

Industry practice types	Functional-based goods	Ornamental-based goods	Probability-based goods
*Power-ups*	X		
*Expansions packages*	X		
*Playable characters*	X		
*Time-savers*	X		
*Loot boxes*			X
*Cosmetics/Skins*		X	

### 2.2. In-game purchase motivations

Videogame players’ in-game purchase motivations are the driving force that impels them to make purchases within the games. Video game researchers have done much work to explore and verify the videogame players’ in-game purchase motivations. A series of in-game purchase motivations have been identified from different perspectives, including perceived values [17,26,36], psychological factors [5], videogame design [6], and service quality [3].

From the perspective of values, Park and Lee (2011) [26] found that the purchase intention of in-game goods is driven by the integrated values of purchasing game items, which includes character competency value, enjoyment value, visual authority value, and monetary value. Later, Hsiao and Chen (2016) [36] considered that in-game purchase intention was driven by emotional value, performance value, social value, value for money, and game loyalty. According to the results, two values, loyalty and good price were found to have a positive impact on players’ in-game purchase intention. Scholars also conducted experimental studies to explore the affective value of in-game goods. In one research design [17], players were randomly assigned to one of the two different mood groups: bored group and stressful group, and the initiated mood was manipulated using artificial simulation. Researchers found that stressed players are more likely to purchase ornamental in-game goods, and bored players are more likely to purchase functional in-game goods [17].

Starting from the psychological perspective, Hamari & Keronen (2017) [5] conducted a meta-analysis using 20 published research, from which they identified ten psychological constructs of in-game purchase motivations: service use enjoyment, subjective norm, flow, attitude toward purchase, service use intention, perceived ease of use, perceived network size, perceived value, self-presentation, and social presence. The terms “flow” or “flow experience” refer to those moments when everything comes together to create a special state of absorption and enjoyment in what one is doing [37]. Entry into the flow experience is characterised by: intense and focused concentration on the present moment, merging of action and awareness, loss of reflective self-consciousness, a sense of control over one’s actions, and distortion of temporal experience [38].

From the side of video game design, Hamari et al. (2017) [6] proposed a set of in-game purchase motivations through reviewing the literature. Their final list contained 19 in-game goods purchasing motivations, which were later condensed into four latent factors (unobstructed play, social interaction, competition, and economic rationale) and two manifest variables (indulging children and unlocking content). Among these variables, unobstructed play, social interaction, and economic rationale are positively associated with the purchase of in-game goods.

Meanwhile, it appears that the factors that drive players to play videogames are not necessarily the factors that drive them to purchase in-game goods. Hamari, Hanner, et al. (2017) [3] conducted survey research and found that although the four dimensions of game service quality (assurance, empathy, reliability, and responsiveness) are positively related to the intention to play, these factors do not significantly explain players’ in-game purchase intention.

Therefore, the literature informs us of the existence of different types of in-game goods and different playing and purchasing motivations. However, very little is yet known about the relationship between different types of in-game purchase motivations and different types of in-game goods. Just as the nature of in-game goods varies, so may the motivations for purchasing them [3,5]. The paucity of studies reporting on the typology of in-game goods [17] is a further hindrance to the analysis of behavioural differences across distinct types of in-game goods [5].

### 2.3. In-game purchase process

Videogame players’ in-game purchase process starts with motivation. However, in many situations, there are more components in the process of players’ in-game purchase.

Previous empirical evidence shows that, while unobstructed play, social interaction and economic rationale are significantly associated with in-game purchase behaviour, this is not the case with the other three types of motivations, competition, indulging children and unlocking content [6]. These results suggest the possibility of more interrelationships between identified motivations and purchase behaviour, depending on the specific type of motivation.

Guo and Barnes (2009) [11] conducted a qualitative study among 24 Chinese videogame players, and they found a series of factors for explaining in-game goods purchase behaviour in virtual worlds, including effort expectancy, character competency, personal real resource, performance expectancy, and self-actualisation. Moreover, in different stages of the purchase, including motivation and behavioural intention, there are different factors involved. In the later quantitative research [24], researchers found that effort expectancy, performance expectancy, perceived value, perceived enjoyment, and customisation are positively associated with purchase intention, while advancement is negatively associated with purchase intention. Moreover, purchase intention along with habit positively affect actual purchase behaviour.

Animesh et al. (2017) [23] used the Stimulus-Organism-Response (S-O-R) framework [39] to explore the impacts of technological and spatial environments on Intention to purchase in-game goods in *Second Life*, a 3D immersive video game. The central factor of their empirical model is flow experience [37], while other factors of environmental stimulus and virtual experience influence intention to purchase by affecting the flow experience.

Additionally, the life cycle of video game players in a specific game does not conclude with single in-game purchase behaviour. It is, in fact, characterised by repeated repurchase. All the consumable in-game goods are designed to be purchased multiple times. Until now, researchers found that user satisfaction and perceived value positively affect the repurchasing intention and recommendation intention within the game, while perceived enjoyment positively affects user satisfaction and perceived value [25].

### 2.4. Our contribution: How videogame players purchase different types of in-game goods

So far, we know that there are different types of in-game goods: power-ups, expansion packages, playable characters, time-savers, cosmetics/skins, and loot boxes. Moreover, these six types of in-game goods can be classified into three categories: functional-based goods, ornamental-based-goods, and probability-based goods. Additionally, in-game goods have different values, which impels players to purchase them. In the purchasing process, different psychological components, such as social influence and flow experience, play an important role.

At the same time, combining the knowledge of in-game goods typology and the in-game purchase behaviour, we found that researchers tended to treat in-game goods as a homogeneous concept in their empirical studies [5,6,23,24], ignoring the significant differences among in-game goods. Treating in-game goods as a homogeneous concept leads to several problems. For instance, players’ motivations could be different when purchasing different types of in-game goods [5,6], which means that the existing knowledge in the literature may not be applicable equally across all types of in-game goods.

Thus, in this study, we explore why and how video game players purchase different types of in-game goods using grounded theory, which will be introduced in the following sections.

## 3. Methodology

### 3.1. Grounded theory

In this research, we choose grounded theory as the research method. Grounded theory is suitable for situations in which interactional elements are involved [40]. Consequently, grounded theory has the potential for a number of directions and contexts in marketing and consumer behaviour [40]. Moreover, grounded theory has been applied in several video games studies [41–45].

Grounded theory methodology provides a tried-and-true set of procedures for constructing theory from data, which have been proven to be culturally sensitive and applicable to individuals as well as larger organisations and societies [32]. Moreover, grounded theory consists of systematic yet flexible guidelines for collecting and analysing qualitative data to construct theories from the data themselves [33]. There are three types of grounded theory: Classic [31], Straussian [32], and Constructivist Grounded Theory [33]. These grounded theory methods have different ontological and epistemological foundations [46,47], and they are neither homogenous nor interchangeable methods [48]. As these three grounded theory methods are based on different research philosophies, none is superior to the others. The basis of our study lies in constructivist grounded theory, which is described by Charmaz [33].

Grounded theory has several methodological characteristics, and theoretical sampling is a hallmark of this methodology [49]. Theoretical sampling is a concept-driven process, which enables researchers to discover the concepts that are relevant to the phenomena and population, and allows researchers to explore the concepts in depth [32]. Unlike conventional sampling methods, when using grounded theory, researchers do not collect all the data prior to the analysis [32]. In turn, researchers first identify the population of interest and settings, and conduct an initial purposive sampling [49]. Once the first wave of data is collected, the analysis begins [32]. Thus, data collection is followed by analysis; Analysis leads to concepts; Concepts generate questions; Questions lead to more data collection [32]. This circular process continues until the research reaches the point of theoretical saturation, at which all major theoretical categories are fully developed, and gathering data no longer sparks new theoretical insight nor reveals new properties of the categories [32,33]. Theoretical sampling involves a particular form of reasoning that characterises grounded theory, which is abductive reasoning [33]. “*Abductive inference entails considering all plausible theoretical explanations for the surprising data*, *forming hypothesis for each possible explanations*, *and checking these hypotheses empirically by examining data to arrive at the most plausible explanation* [33]”. Researchers should not confuse the theoretical sampling of grounded theory with positivist quantitative research [33]. Grounded theory is not a theory testing method [50]. In general, the ontological and epistemological assumptions of qualitative research are different from those of positivist quantitative research [51,52]. Qualitative research lacks a particular type of generalisability: the statistical-probabilistic generalisability, which therefore does not grant researchers the confidence about the representativeness of their sample and broader inferences of the results [51]. The type of generalisability that a qualitative research seek is usually the analytical generalizability: researchers generalise a particular set of results to the established or new concepts/theories [51].

We also would like to highlight the position of literature review in our research. The position of literature review in grounded theory research has long been both disputed and misunderstood [33]. The classic grounded theory [31,53] advocates that the researcher should delay the literature review after completing the data analysis [33]. The idea behind this approach is to avoid importing preconceived ideas and imposing them on the researcher’s work [33]. Classic grounded theory advocates that researchers should keep themselves uncontaminated by extant knowledge [53–56].

However, some researchers do not agree with Glaser and Strauss’s original pronouncement [31] and Glasser’s continued statements [53–55]. For instance, Suddaby (2006) [50] considered that the belief of not doing literature review before entering the field is a myth, which is based on the false premise that the researcher is a blank sheet devoid of experience or knowledge. Moreover, disregard of the previous literature could also lead researchers to “reinvent the wheel” and reproduce common-sense categories [33,57–59].

In this grounded theory research, our attitude toward the literature review is in line with that of Charmaz (2014) [33]. We do not treat prior knowledge as an obstacle to our creativity. Instead, we use a literature review to clarify our research boundary, propose research questions, and show how our work fits into and extends the current literature. Moreover, prior literature serves as an important component in the theoretical sampling phase, which helps to cultivate theoretical sensitivity and think abductively.

Some researchers in the area of videogame study have already applied grounded theory to explore players’ motivations to play social network games [12]. However, in general, grounded theory has not been widely applied in the discipline of consumer behaviour. Thus, details about this method and our research process will be provided in the next section.

### 3.2. Research process

**Fig 1:**
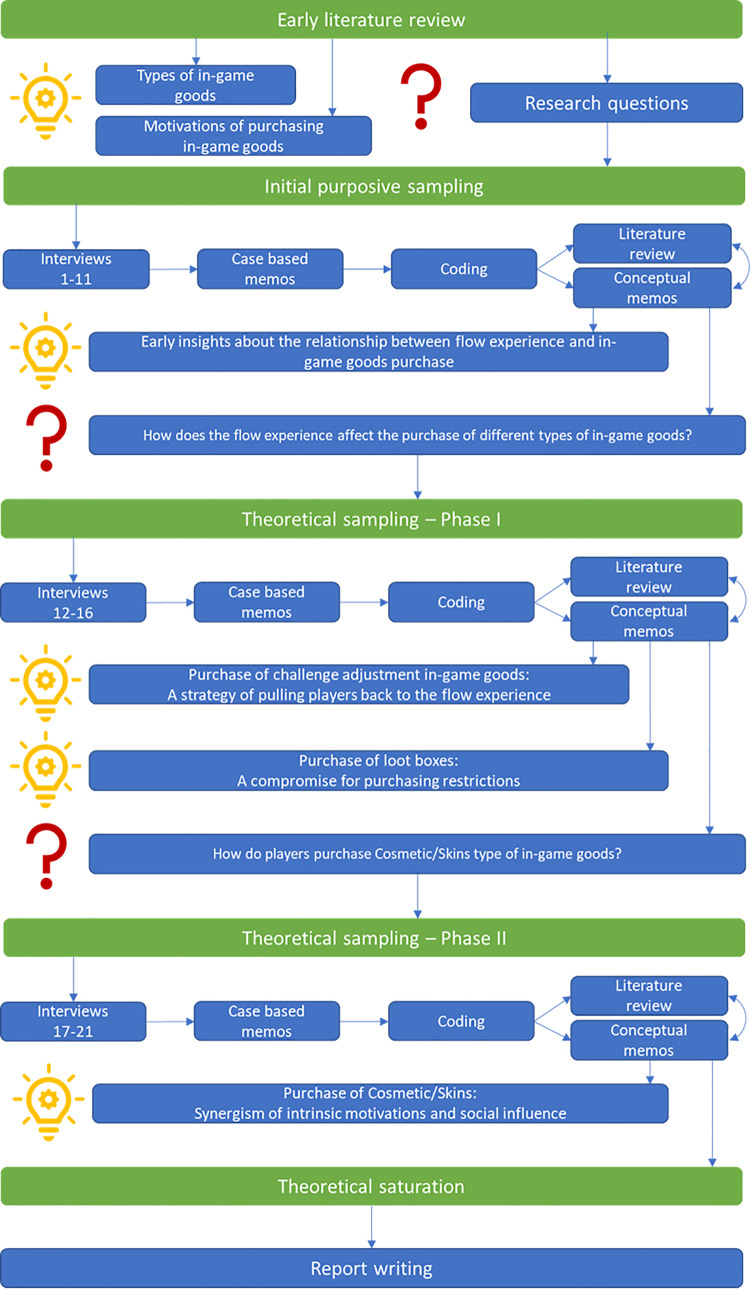
Research process.

Fig 1 shows the process of our research. After doing the literature review, we learned that industrial practitioners acknowledge the existence of six types of in-game goods (p*ower-ups*, *expansion packages*, *playable characters*, *time-savers*, *loot-boxes*, *cosmetics/skins*), which can be classified according to their nature: functional-based goods, ornamental based goods, and probability-based goods. Along with the research, we only mentioned the industrial classification approach with our informants to avoid cognitive bias, which may be caused by the obscure academic definitions. At the same time, we proposed our research questions because the existing knowledge could not explain well the in-game purchase of heterogeneous goods. Therefore, our research journey started with inductive reasoning to explore the answer to the research questions.

Videogame players from China were selected as informants in this research. Chinese informants were selected because China is the largest single market of video games in the world (Newzoo, 2018) [1].

In this research, we used the online semi-structured interview as the primary data collection method for several reasons. First, interviews fit well with grounded theory, because of their open-endedness [33]. In order to determine why and how video game players purchase different types of in-game goods, we needed to capture video game players’ perceptions, attitudes, and actions during the behavioural process. Therefore, in this case, interviews helped us to conduct an open-ended, in-depth exploration of an area in which the interviewee has substantial experience [33]. Second, we chose the semi-structured interview rather than unstructured or structured interview because this approach enabled us to ensure a degree of consistency among the concepts covered in each interview [32] while the main conversion was still open-ended. Third, we opted for online data collection because the temporal and spatial flexibility provided by the internet benefit qualitative research [60]. The online method allowed us to reach diverse, geographically scattered populations, as well as informants who were not easily available due to timetable issues. Moreover, virtual anonymity and higher private self-awareness foment the disclosure of personal information and deep feelings [61].

In the initial purposive sampling phase, data collection began in a videogame discussion group on WeChat, the most used social media platform in China. We enrolled three qualified members of the discussion group on our research, and all the following participants were drawn from snowball sampling. The research process put each informant through three steps: they were contacted on WeChat, answered the questionnaire on Qualtrics, and were interviewed on QQ. During the initial contact on WeChat, we presented the basic information of our research to probe the informants’ willingness to participate in the study, which was interspersed with some daily chats. This helped us to build some initial rapport with the informants, which is a crucial prerequisite of interviews with Asian informants [61]. Subsequently, we sent an anonymous link of survey to the informant via WeChat. The online questionnaire based on Qualtrics helped us to acquire some basic information related to informants’ gaming habits, demographic profiles, the personal contact information (QQ and email addresses), and a suitable time for the interview.

With respect to ethical considerations, we would like to mention that our research is a consumer behaviour study and not a clinical trial. The researchers of this study are affiliated with a Spanish research institute. Moreover, all the informants of our research are Chinese citizens and residents in China. Thus, we should comply with the corresponding regulations of Spain (URL: https://www.boe.es/buscar/doc.php?id=BOE-A-2015-14082) and China (http://www.nhc.gov.cn/fzs/s3576/201610/84b33b81d8e747eaaf048f68b174f829.shtml). However, as our research is a consumer behaviour study involving no clinical intervention with human subjects, the mentioned regulations are not applicable in this case. As a result, we did not seek approval from the ethics committee/IRB. However, all candidates who wished to participate in the research were required to sign a written informed consent at the beginning of the online survey, where the basic information of this study, the right of voluntary withdrawal, and the economic reward (An electronic gift card with a denomination of 100 RMB, about €15) for participating in this study were explicitly stated.

Later, semi-structured interviews were organised for eligible participants in the survey, who had played video games and purchased in-game goods in the last six months. The interviews were synchronously conducted online using QQ, a leading instant messaging software in China. Informants were encouraged to use emoticons to remedy the lack of nonverbal cues during the text-based online interview.

Of the eleven informants (001–011) who participated in the initial purposive sampling, there was one (006) who completed the questionnaire and was eligible to take part but, for some undisclosed reason, failed to show for the interview. Thus, we had ten interviews in the initial purposive sampling phase. In the first wave of theoretical sampling, we interviewed five informants who had already participated in the initial purposive sampling (002, 004, 007, 009, and 010). In the second wave of theoretical sampling, we interviewed six informants (007, 009, 010, 012, 013, and 014), including three informants that had already participated in the two previous sampling phases (007, 009, 010) and three new informants that had never been involved in our research (012, 013, and 014). It is worth mentioning that the number of interviews in each phase of the sampling was established not *a priori* but *a posteriori*. Analyses based on grounded theory should ideally begin after completing the first interview and continue that way throughout the research process [31,32]. Some grounded theorists warn of the danger of collecting all the data at once, because this approach limits the potential for theoretical sampling [32].

After the interviews, the transcriptions of the chat records were translated from Simplified Chinese to English. This enabled the initial coding which was followed by more focused coding, after which case-based and conceptual memoing were carried out [33]. We used NVivo 12 software to manage transcriptions, codes, and memos more conveniently. When conducting the initial coding, we followed the incident with incident coding. The incident with incident coding is especially ideal for grounded theory, which facilitates one of the core tenets of this qualitative method: constant comparison [33]. At each level of analysis, the data, including emerging codes, categories, and properties were constantly compared [31]. One of the authors, who is a native Chinese speaker, translated the original transcript from Chinese to English, conducted the coding, and wrote the memos. All the mentioned research materials were shared among the authors, who collaborated in the critical decision-making for each phase of the theoretical sampling and at the theory integration stage. The same procedure was used for the two following data collection phases.

After conducting the ten interviews in the initial purposive sampling phase, we acquired some early insights into the relationship between flow experience [37] and in-game goods purchase. However, we still did not know how the flow experience affected purchase behaviour across different types of in-game goods. With this question in mind, we started the first wave of theoretical sampling.

After analysing the data acquired from the first wave of theoretical sampling, we found that players purchasing functional-based goods is one of the strategies to alleviate boredom and anxiety and to reach a state of flow. Moreover, we also unanticipatedly found that the purchase of probability-based goods was a compromise for purchasing restrictions. However, although we had strengthened our conceptual framework by incorporating the new knowledge obtained from the theoretical sampling, it still fell short of explaining the purchase of ornamental-based goods. This theoretical crack pushed us to conduct the second wave of theoretical sampling in order to explore the purchase patterns for ornamental-based goods.

The results of the data analysis in the second wave of theoretical sampling showed that the purchase of ornamental-based goods is a synergism of intrinsic motivations and social influence. At that moment, we reached the point of theoretical saturation, in which fresh data no longer sparks new theoretical insights nor reveals new properties of the theoretical categories [32,33,62–64].

## 4. Profiles of informants

Fourteen informants participated in our research. Among all the informants, 001, 003, 005, 008, and 011 participated only in the Initial purposive sampling phase. Informants 002 and 004 participated in the Initial purposive sampling phase and theoretical sampling phase I. Informant 007, 009, and 010 participated in all the sampling phases. Informants 012, 013, and 014 only participated in the theoretical sampling phase II. Informant 006 filled the questionnaire but did not show in the interview.

The demographic profiles of the informants are summarised in Table 2. With respect to the demographic profile of the Informants, ten informants are male, and four are female. Their ages range from 23 to 31. Most of the informants are not students, except informant 009, who is a full-time university student. Most of the informants have received higher education, except for the informants 012 (Middle school) and 014 (Vocational school). Most of the informants have full-time jobs, except the informant 006, who is unemployed. The informants’ monthly incomes range from 2001 RMB to over 20001 RMB. Most of the informants live in Shanghai, China, except for the informants 006 (Tongren, Guizhou province of China) and 014 (Hunchun, Jilin province of China).

**Table 2:** Gaming profile of the informants.

Informant	Gender	Age	Student	Education	Job	Monthly income	Town	Initial sampling	Theoretical sampling I	Theoretical sampling II
001	Male	30	No	Undergraduate	Yes (Full time)	7001 RMB -9000 RMB	Shanghai	Yes		
002	Male	29	No	Undergraduate	Yes (Full time)	More than 20001 RMB	Shanghai	Yes	Yes	
003	Male	25	No	College	Yes (Full time)	5001 RMB -7000 RMB	Shanghai	Yes		
004	Male	31	No	Undergraduate	Yes (Full time)	11001 RMB -15000 RMB	Shanghai	Yes	Yes	
005	Male	30	No	Undergraduate	Yes (Full time)	7001 RMB -9000 RMB	Shanghai	Yes		
006	Male	30	No	College	No	2001 RMB -3000 RMB	Tongren, Guizhou			
007	Male	29	No	College	Yes (Full time)	7001 RMB -9000 RMB	Shanghai	Yes	Yes	Yes
008	Female	28	No	College	Yes (Full time)	3001 RMB -5000 RMB	Shanghai	Yes		
009	Female	29	Yes	Undergraduate	Yes (Full time)	11001 RMB -15000 RMB	Shanghai	Yes	Yes	Yes
010	Female	28	No	Undergraduate	Yes (Full time)	15000 RMB -20000 RMB	Shanghai	Yes	Yes	Yes
011	Male	23	No	College	Yes (Full time)	3001 RMB -5000 RMB	Shanghai	Yes		
012	Male	28	No	Middle school	Yes (Full time)	7001 RMB -9000 RMB	Shanghai			Yes
013	Male	26	No	Undergraduate	Yes (Full time)	Don’t Know/No Answer	Shanghai			Yes
014	Female	25	No	Vocational school	Yes (Full time)	Don’t Know/No Answer	Hunchun, Jilin			Yes

Table 3 summarises the gaming profile of the informants. They are heterogeneous in their choice of game platform, and thus include computer game players (001 and 012), mobile game players (014), console and mobile game players (008), computer and mobile game players (002, 009, 010, 011, and 013), and all-platform players (003, 004, 005, and 006). In term of the gaming habit, 7–12 hours a week is the most frequent pattern. Moreover, many Informants continuously play 1–2 hours a time. Finally, the profiles of the informants cover customers of all types of in-game goods.

**Table 3:** Demographic profile of the informants.

Informant	Platform	Gaming time per week	Continuous gaming time every time	Purchased in-game goods types
001	Windows	12–20 hours a week	1–2 hours at a time	Cosmetics/Skins, Loot boxes
002	Windows, Andriod, IOS	12–20 hours a week	30 minutes to 1 hour at a time	Cosmetics/Skins
003	Windows,PS4,Nintendo Switch,PS Vita,3DS,Andriod	More than 20 hours a week	More than 5 hours at a time	Power-ups,Expansion packages,Playable characters,Cosmetics/Skins,Loot boxes
004	Windows, PS4, IOS	More than 20 hours a week	2–5 hours at a time	Power-ups,Expansion packages,Playable characters,Cosmetics/Skins,Loot boxes,Time-savers
005	Windows, Nintendo Switch, Andriod, IOS	12–20 hours a week	1–2 hours at a time	Expansion packages, Playable characters, Cosmetics/Skins
006	Windows,MAC,PS4,Nintendo Switch,PS Vita,3DS,Andriod	7–12 hours a week	1–2 hours at a time	Cosmetics/Skins
007	Windows, PS4, Nintendo Switch,3DS, IOS	7–12 hours a week	30 minutes to 1 hour at a time	Expansion packages, Cosmetics/Skins
008	Nintendo Switch, IOS	4–7 hours a week	30 minutes to 1 hour at a time	Loot boxes
009	MAC, IOS	7–12 hours a week	2–5 hours at a time	Power-ups, Playable characters, Cosmetics/Skins, Loot boxes
010	Windows, Andriod, IOS	More than 20 hours a week	More than 5 hours at a time	Power-ups,Expansion packages,Playable characters,Cosmetics/Skins,Loot boxes,Time-savers
011	Windows, Andriod	7–12 hours a week	1–2 hours at a time	Power-ups,Expansion packages,Playable characters,Cosmetics/Skins,Loot boxes,Time-savers
012	Windows	More than 20 hours a week	1–2 hours at a time	Cosmetics/Skins
013	Windows, Andriod, IOS	7–12 hours a week	2–5 hours at a time	Power-ups,Expansion packages,Cosmetics/Skins,Loot boxes,Time-savers
014	Andriod	12–20 hours a week	30 minutes to 1 hour at a time	Power-ups, Playable characters, Cosmetics/Skins, Loot boxes

## 5. Findings

In general, our findings show that players are driven by disturbing external stimuli to purchase in-game goods. Moreover, we have identified three different purchase processes for functional-based goods, probability-based goods, and ornamental-based goods. For functional-based goods, the external stimulus is the imbalance of challenges and skills, which generates anxiety and boredom, to which players react by purchasing functional-based goods. For ornamental-based goods, the external stimulus is the exposure of cosmetics/skins in the virtual game world, which triggers feelings of envy and the need to be different and attractive. The resulting discomfort leads players to purchase ornamental-based goods. As for probability-based goods, the external stimulus is the purchase restrictions embedded in the game, which prevents players from alleviating their discomfort through the direct purchase of a coveted item. In this situation, the purchase of probability-based goods is an alternative way to respond to players’ discomforts.

We will now discuss these three purchase processes in more detail.

### Purchase of functional-based in-game goods: A strategy of entering the flow experience

Video game players do not purposively purchase functional-based goods. Instead, they purchase them to enter or to return to the flow state.

Our informants described the various states they entered during their flow experience, which correspond to the dimensions of flow. Informant 002 reported playing for very short periods (around 15 minutes), during which he would be very concentrated and his thoughts were focused on winning the game; Informant 010 mentioned that she was not aware of what was happening in the outside world, and she would unconsciously reply to questions from her friend; Informant 004 mentioned that he felt that he could easily kill the opposite players without considering their levels in the game, which made him feel invincible; Informant 010 mentioned that the time passed very quickly, and two hours could go by in the blink of an eye. Apart from their personal experience in the flow, our informants mentioned that having the flow experience itself is their main motivation to play video games because they can deliberately get isolated from the outside world and temporarily enter to their own virtual world. Through this way, videogame players can shut out real life for a while and enjoy their joy time.

However, players do not always acquire this enjoyable experience. Although players play the game to reach the flow state, the results of playing can also trigger negative emotions. On the one hand, players may experience anxiety when faced with challenging situations. For instance, players may be stuck in a game stage for a long time without having the chance to advance. On the other hand, players may experience boredom when facing some insufficiently challenging situations. For example, players usually feel bored when they find they are doing repetitive tasks without any progress, and in this circumstance, the game becomes a boring loop.

The basic ingredient of a flow-inducing environment is a challenging situation [65]. It is necessary to mention that in the context of videogames, the challenge not only refers to game difficulty but also refers to its explorable content. For example, in some games, players are required to select the level of difficulty (Easy, Normal, or Difficult) and explorable content (the stages) of the game before starting the game.

The player’s skill level is another key consideration in achieving the flow experience [37]. In the context of the video game, skill is determined not by the player’s personal gaming ability but by the mechanics of the game. For instance, many freemium games have mechanisms that limit a player’s capability in the game environment if they do not recharge. Informant 009 mentioned having played a game where some players were excluded from some major in-game activities because the abilities of their virtual characters were restricted until they were recharged.

The right balance between challenge and skills is one of the conditions to enter the flow state [37,38,65]. Otherwise, if challenges exceed skills, players will become anxious. Moreover, if skills exceed challenges, players will become bored. Informant 007 described that anxiety, boredom, and flow were three mutually exclusive states, and they could not co-exist at the same time.

Another condition to enter the flow state is having clear goals [37,38,65]. During the time and effort they devote to a game, players are guided by one or several goals. Every game is played according to the rules and mechanisms for reaching certain goals. Goals have many manifestations. For instance, in League of Legends, the goal is to win the current round, while in Luanshiwangzhe (乱世王者, a Chinese strategy mobile game developed by Tencent’s Tianmei studio group), the main goal is to win the tournament in the game server.

**Fig 2:**
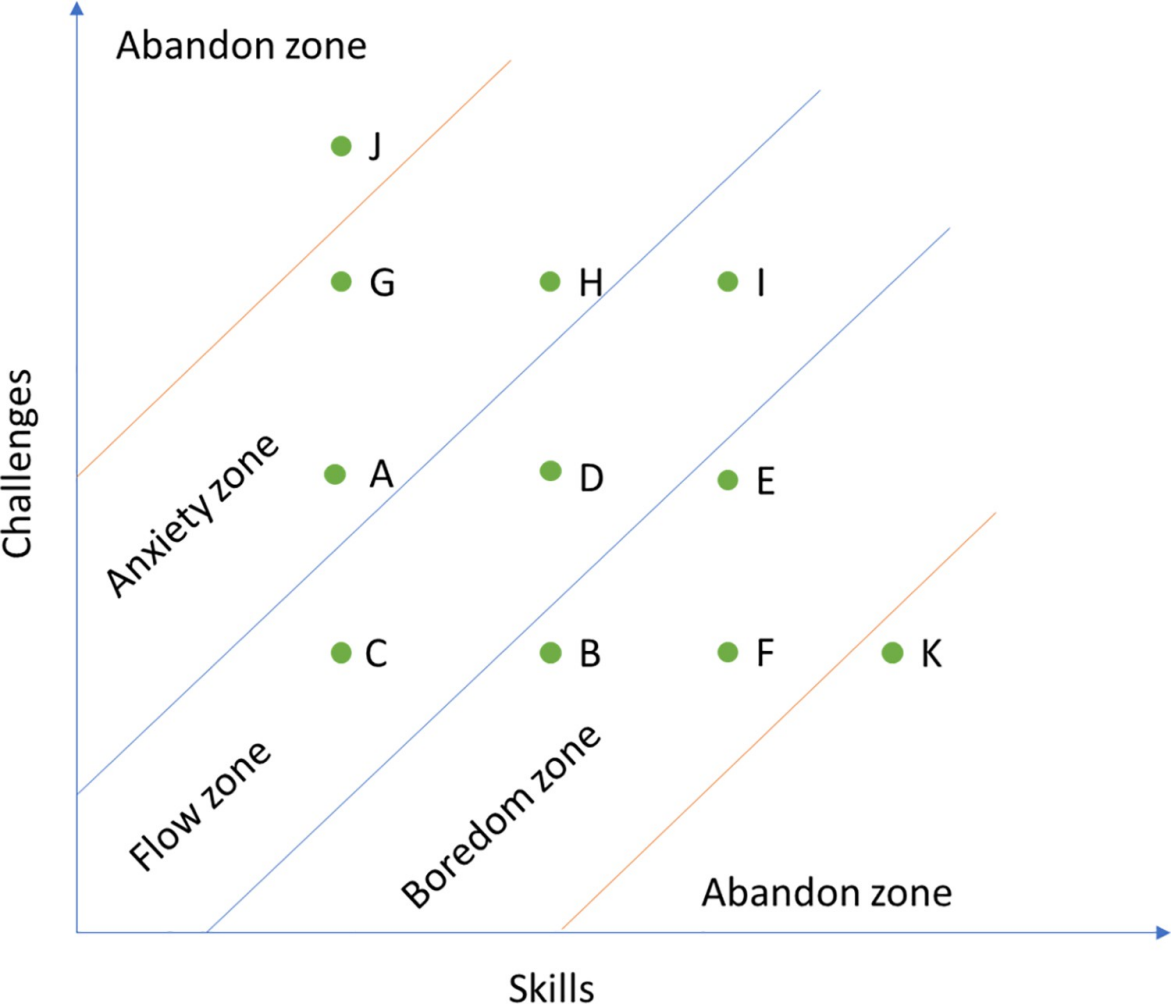
Flow and discomfort situations.

In ideal circumstances, with clear game goals, and a correct challenge/skill balance, players enter a state of flow. This situation can be visualised in Fig 2. We assume that the player’s initial state is point C (flow state). As the gameplay advances, not only do the player’s gaming skills increase, but also his/her capability within the game. At the same time, the difficulty of the game is also increasing, and new game content is being introduced. If skills and challenges increase at the same rate, the player enters the balance zone, where players acquire the flow experience. Throughout the gaming experience, the player starts from point C, moves to point D, and finishes at the point I. Thus, in ideal circumstances, the player is always in the flow state during his/her gameplay, and thus enjoys a pleasant gaming experience.

Beyond the ideal circumstance, players may feel anxious or bored. If the challenges increase at a higher rate than the skills, the player’s mental state moves from point C to point A, which is in the anxiety zone. If, on the contrary, the player skills increase at a higher rate than the game challenges, his/her mental state moves from point C to point B, which is in the boredom zone. In either of the above situations, the dynamic balance between challenge and skill is broken, and the player enters the discomfort state.

At that point, there are several possible strategies for players to re-enter the flow state. In the case of the anxious experience, the player can choose to hone his/her skills (Move from point A to point D) or switch to an easier stage of the game (Move from point A to point C) to re-enter the flow state. In the case of the boring experience, the player can choose to play more challenging stages to re-enter the flow state (Move from B to D).

The situation discussed so far occurs in traditional videogames, where no in-game goods are available. However, things change completely where there is a chance to purchase in-game goods, such as in freemium games. Some freemium games are notorious because their operators raise the game challenge to an extremely high level. For instance, informant 003 mentioned:

“*Some activities are beyond the limits of normal people*. *For example*, *the operator could design an activity that would require you to fight 7 days X 24 hours to gain the final reward*.”

Moreover, some freemium games tend to suppress players’ ability inside the game. For instance, informant 009 mentioned:

“*The ability that the game mechanism endows to the player mainly depends on whether [the player] has recharged enough money*. *Nothing is gained without recharging*.”

Under this circumstance, strategies such as honing one’s skills or shifting to a lower-level challenge are of no use. Due to the high challenge, the player’s state would be at point G, and the mentioned approaches may only help to move to points H or A, which means that the player still is in the anxious zone. Meanwhile, game operators have their own solutions to tackle the problem: offering in-game goods to players. After using, for example, Power-ups, players can become incredibly stronger, which helps to move from point G to point I. Similarly, after using Time-savers, the game challenge is significantly reduced, which helps ‘to move from points G to C. In either case, players would be at the flow zone after purchasing in-game goods.

A strategy used by some freemium video game operators is to lock certain items of game content and make them exclusively purchasable. This limits the game challenge level. As informant 010 reported:

“*Nowadays*, *there are a lot of games which are similar to each other*. *They tempt you to buy this or that*. *You only get a few minutes playing [the game] before they make you spending money to unlock the stages*. *In some very direct cases*, *you need to recharge a few RMBs to buy an item*, *and they let you continue*.”

In this case, the approach of shifting to a more challenging game state does not work, simply because the operator does not allow players to do so without purchasing in-game goods. Due to the low challenge, the player’s state would be at point F, and the free challenges in the game may only allow players to reach point E, which means that players still are at the boredom zone. At this point, there are expansion packages and playable characters available in the game, and each of them serves to increase the game’s challenge level. After purchasing these in-game goods, players will have a more challenging experience in the game, which enables a shift from points F to I. Thus, the player would reach the flow state after purchasing in-game goods.

However, in-game goods are not always magic wands. When the challenge far outweighs the skill (point J), players will remain in a state of high anxiety even after purchasing in-game goods, and the way they relieve this negative experience is to abandon the game. Informant 010 shared her experience:

“*When I found that*, *even after buying many Power-ups*, *I still could not catch up with the troop*, *I began to want to give up*. *This is the case with the game I’m playing now*. *I feel I’ve reached a bottleneck*. *All the attributes are not able to be enhanced*. *(I) can’t be part of the main team*. *It makes me very sad*.”

Additionally, when the challenge is far below the skill level (point K), players will continue to be extremely bored even after purchasing in-game goods, and the way they relieve this negative experience is to abandon the game. Informant 010 shared her experience:

“*For instance*, *[In a game called] Mr Love*: *Queen’s Choice*, *I recharged 2 or 3 times*, *(but) I still felt bored*. *So*, *I gave up*. *The same with Star Dream*.”

Although players’ post-purchase behaviour is not the focus of our study, for the integrity of the behavioural loop, we will describe a few of the details. First, even once attained, the player’s flow experience does not remain stable, which means it can be terminated due to a break in the balance between challenge and skill. In this case, players would re-enter the anxiety zone or boredom zone. Second, the flow experience can also be interrupted by external interferences (Calls from others, emergencies, and so on) and physiological needs (Sleep, eat, need for the bathroom, and so on), which consequently lead to the abandonment of the game.

There are two types of abandonment: *temporary abandonment* and *permanent abandonment*. We are currently concerned with temporary abandonment, which is the behavioural response to anxiety or boredom, external interferences, or physiological needs. Frequent occurrences of temporary abandonment due to anxiety or boredom can lead to permanent abandonment. When defined in terms of time, the boundary between temporary abandonment and permanent abandonment can be blurred. However, according to our Informants, a more effective way of defining these two concepts is based on the players’ mindset. When in the state of temporary abandonment, players are still missing the game and use this time to recover from the negative mood caused by anxiety or boredom. After a period of temporary abandonment, players still wish to return to the game. Players in a state of permanent abandonment, however, have no room to think about the game, and their most explicit behaviour is to uninstall it.

Having abandoned the game permanently, a player is very unlikely to return to it, except for one thing, namely, nostalgia; that is, emotions reflecting the positive bond previously existing between the player and the game. Even after permanently abandoning a game, players might check for recent updates of the game and take a chance on returning.

**Fig 3:**
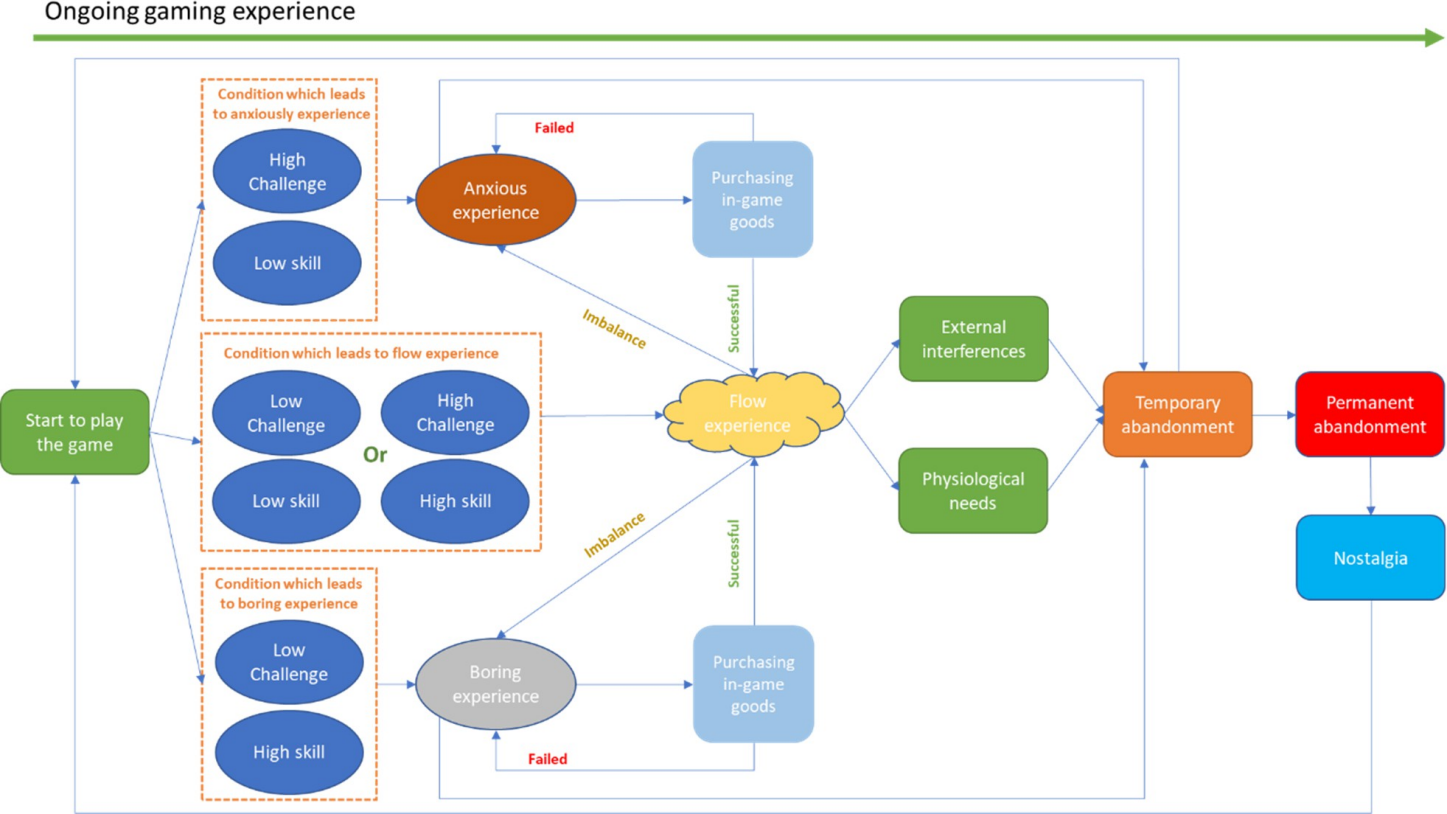
Purchase of challenge and skill adjustment in-game goods: A strategy of pulling players back to the flow experience.

The whole behavioural loop we have mentioned so far is illustrated in Fig 3.

We need to acknowledge some important findings regarding the transition from the discomfort zone (anxiety or boredom) to actual purchase. We will explain the details in the following paragraphs.

It is important to know that purchasing in-game goods is not the only way to get rid of the discomfort states and to enter the flow state. Freemium game players will also find their own ways to achieve flow, just as they do in traditional games where no in-game goods are available. This is because the mechanics of the game sometimes allow in-game items to be obtained free of charge. In-game items become in-game goods when players have to pay for them with real money. However, some players are aware that operators and developers of freemium games tend to set a series of obstacles to prevent them from having the flow experience without first purchasing in-game goods. There are three types of obstacles in the game: Preventing players from decreasing challenges, preventing players from developing skills, and preventing players from increasing challenges. The existence of these obstacles leads to the situations described previously: Instead of moving from point G to point C, players’ state only could reach to point A; Instead of moving from point G to point I, players’ state only could reach point H; Instead of moving from point F to point I, players’ state only could reach to point E. Informant 007 shared his experience with the obstacles created by the game mechanics:

*“I would evaluate the possibility to acquire in-game goods through a free method. But the reality is cruel. Zero investment is basically hopeless. If the alternative solution takes too much time and the requirement is too high, it is better to buy directly.”*

Under this circumstance, players will have an intention to purchase in-game goods.

Social factors also have an impact on the players’ purchasing process. The social influences that players receive are from the different reference groups, including family members, real-world friends, colleagues, network friends, members of the gaming league, the hosts of live broadcast platforms, and so on. There are various types of social influence. First, players are influenced by subjective norms. Players’ intent to purchase in-game goods could be reinforced by encouragement from opinion leaders in their reference groups. For instance, informant 009 was a member of her gaming league, and her purchasing intention was reinforced when other members of the league expected her in-game characters to be stronger. Similarly, players’ intent to purchase in-game goods could be reduced by their reference group opinion leaders advising against it. Informant 012, for instance, felt his purchasing intention weaken when his wife did not support his idea. Besides, players are influenced by positive and negative Word-Of-Mouth (WOM). Players value the opinions of those who have already purchased in-game goods. While positive WOM reinforces purchasing intention, negative WOM reduces it. For example, informant 007 reported that his purchasing decision was significantly affected by the positive and negative opinions given by those league members who had purchased the in-game goods that he coveted.

Promotions also play an important role in the in-game goods purchasing process. Players’ purchasing intention will strengthen at the sight of promotions. Nowadays, in online games, in-game festivals or activities are often synchronized with real-world festivals, and sometimes promotions are integrated into these in-game festivals. For instance, informant 008 mentioned that her purchasing intention was stronger when she saw that there were promotions available during the in-game festivals.

**Fig 4:**
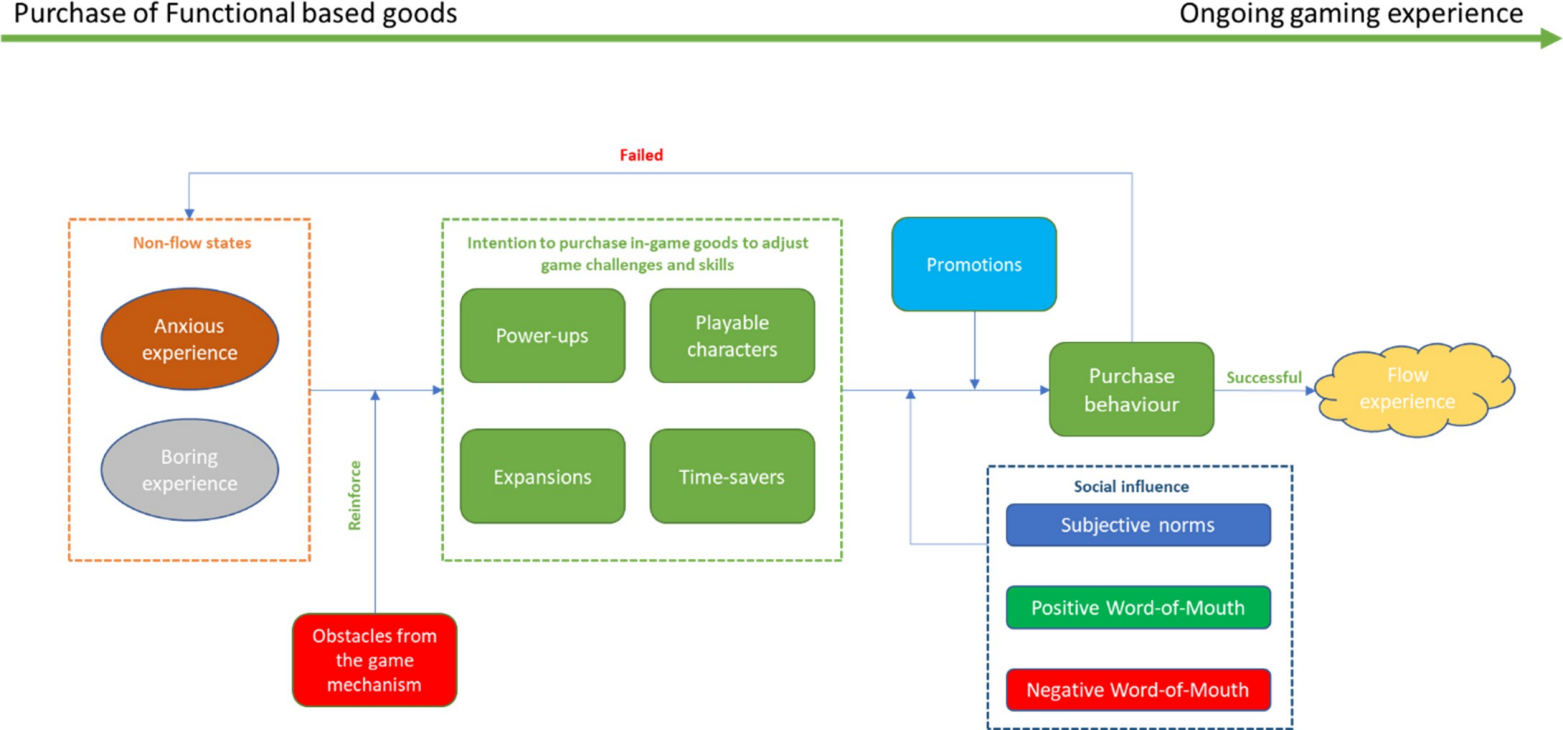
Purchase process between the non-flow state and actual purchase behavior.

The purchase process between the discomfort state and actual purchase behaviour is illustrated in Fig 4.

### Purchase of probability-based goods: A compromise for purchase restrictions

So far, our grounded theory has explained the purchase of certain types of in-game goods, which serve to adjust challenges and skills. However, to explain the purchase of probability-based goods, we need to expand our existing theory. Our research has shown that the purchase of probability-based goods is a behaviour driven by purchase restrictions and the gambling experience.

Players purchase loot boxes, not for the boxes themselves, but the items inside the boxes. As we have stated above, players at discomfort zones would like to get rid of the negative state, and purchasing in-game goods is one of the strategies. However, in many freemium games, there are purchase restrictions that prevent players from purchasing certain in-game goods.

A common purchase restriction that some game operators apply is to block the direct purchase of in-game goods and, instead, offer them as prizes in a lucky draw. For example, informant 009 mentioned that the operator of the game she played did not allow players to purchase directly new playable characters, and the only way to acquire them was to purchase loot boxes. Consequently, the intention to purchase functional-based goods turns to the intention to purchase probability-based goods.

Drummond and Sauer (2018) [66] considered that loot boxes were psychologically akin to gambling because this sort of in-game goods presents several striking similarities to real-world gambling, although, in many countries, they do not legally constitute gambling. Gambling motivation also plays an important role in the process of purchasing loot boxes because players want to spend less to win more. For instance, informant 008 mentioned that she had a gambling mentality when purchasing loot boxes, which she saw as a way to get more for her money than through direct purchase, which she perceived as more expensive. After purchasing and opening the loot boxes, players would enter a gambling-like experience. In this state, players feel very anxious and experience an uncertain pleasure, which consequently leads to their intention to purchase loot boxes again.

Moreover, the result of loot boxes is binary: players either obtain their desired items, or they do not. In case players win their desired items, they can use the items to alleviate their anxious or boring experience and thus enter the flow state. In the case players do not win their desired items, some players will quit the lucky draw, but others will try more times before finally winning the desired items. The reason why players would like to iterate the lucky draw is quite straightforward: Many Chinese freemium games are not completely based on a variable ratio reinforcement schedule. Instead, many of them are based on a fixed reinforcement schedule. The fixed reinforcement schedule enables players to acquire all the items in a loot box after a given number of rounds of lucky draw. Additionally, the result of loot boxes not only affects the players themselves but also other players because players would share their experience of playing loot boxes with their friends in the same reference group. Thus, the positive and negative results of loot boxes would lead to positive WOM and negative WOM respectively. The far-reaching impact of the positive or negative WOM is that these communications can affect other players’ intent to purchase loot boxes. For example, informant 010 shared her experience:

"*Some of us was really evil*. *He won the item and told [us] that the reward was really good*. *Consequently*, *he encouraged us to participate in the lottery*… *When I draw*, *I got some rubbish*! *I spent 20*,*000 ingots and gained nothing*. *Then I just needed one coupon* (Play one more round, because in some games, the lottery mechanism allows players to acquire all the items in the loot box after n rounds.) *to exchange the prize*, *and I moved on*! *We all scolded him*: *"Are you a capper*?*"* "

In addition to the above, promotions also play a key role in the purchase process of probability-based goods. Informant 008 shared her experience:

“*Playing mobile games is a common pastime*, *and occasionally paying to take part in a draw is very cool*. *For example*, *there is a price cut for loot boxes*… *Girls always do shopping during 11*.*11 and 6*.*18* (11.11 and 6.18 are two e-commerce promotional festivals in China.), *don’t they*?”

**Fig 5:**
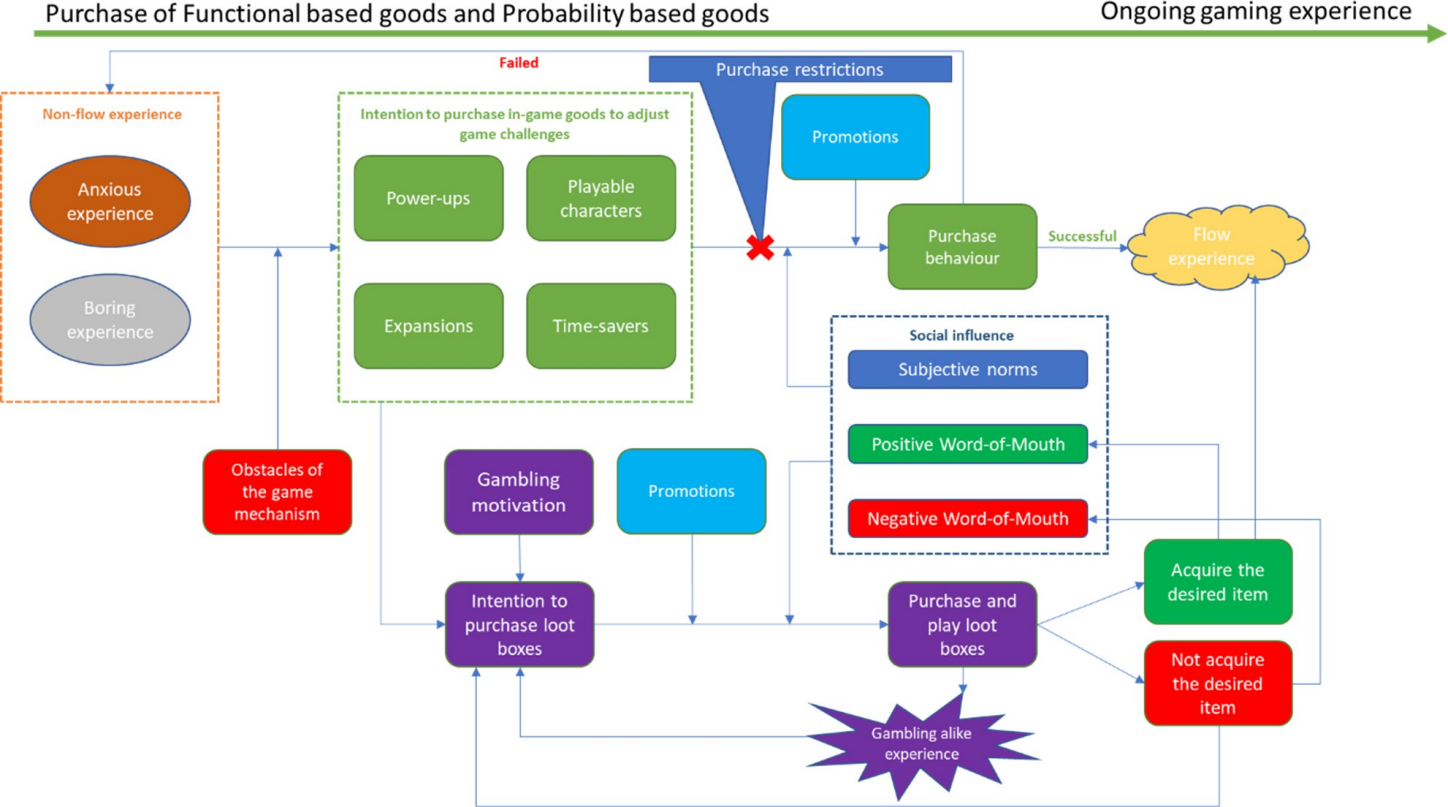
Purchase process between the non-flow state and actual purchase behaviour, with the existence of loot boxes.

The purchase process of probability-based goods extends the theory of Fig 5, which is illustrated in Fig 5.

### Purchase of ornamental-based goods: Synergism of intrinsic motivations and exposure

Although the theory described so far can explain the purchase of functional and probability-based goods, it still cannot explain the purchase of ornamental-based goods. This study reveals that the purchase of ornamental-based goods is a synergism of intrinsic motivations and social influence.

Video game players, like consumers the world over, cannot avoid the influence of other people. We found that there were three key factors (envy, the need to be unique, and the need to be attractive) that leaded players’ intention to purchase ornamental-based goods.

First, players purchase ornamental-based goods because of the envious emotion. Some players are very envious when they see other player’s characters wearing a coveted skin that they do not have, and consequently, they have the intention to purchase the same skin as well. “*People are jealous*. *If I didn’t have that [skin]*, *I certainly would be envious*.” said informant 007.

Second, players purchase ornamental-based goods to be different. Players in the videogames want their virtual appearance to be different from other players. For instance, informant 013, a *World of Warcraft* player, said he wanted *Astral Cloud Serpent* (A rare skin in *World of Warcraft*.) because of its unique appearance. Being unable to obtain this skin for free through the game mechanics, his intention was to purchase this skin directly at the in-game mall. However, he gave up this idea when he saw that many players in the virtual world already had this skin. As a result, although the need to be different firstly drives players to purchase ornamental-based goods, the intention of purchase can be weakened by the large size of avoidance group, who have already equipped the cosmetics/skins that players desired.

Third, players purchase ornamental-based goods to be attractive. During the interviews with the Informants, the word “Good looking” appeared repeatedly. The pursuit of beauty is a human instinct, and players transfer this need to their virtual characters or assets in video games. Some Informants mentioned that their virtual characters and assets should be attractive for that they could reach self-satisfaction.

One important outcome from the purchase of ornamental goods worth mentioning is exposure. Whether intentionally or unintentionally, the purchaser of in-game ornamental goods, exhibits the item to other players. The exposure of ornamental-based goods increases the size of the reference group (number of owners of a certain skin in the virtual world), thus fomenting other players’ envy and need to be different.

We also found that some theoretical components mentioned in the previous sections had the same roles in the purchase process of ornamental-based goods. First, the obstacles of acquiring ornamental-based goods through game mechanics reinforce players’ purchasing intention. Second, purchase restrictions of ornamental-based goods push players to purchase probability-based goods where there are ornamental-based goods, and the post-purchase behaviour remains consistent with what we have mentioned in the previous section. Third, social influences, including subjective norms, and positive and negative WOM, also influence players’ purchase intention of ornamental-based goods, just as they do in other categories. Fourth, promotions also reinforce players’ purchasing intentions.

**Fig 6:**
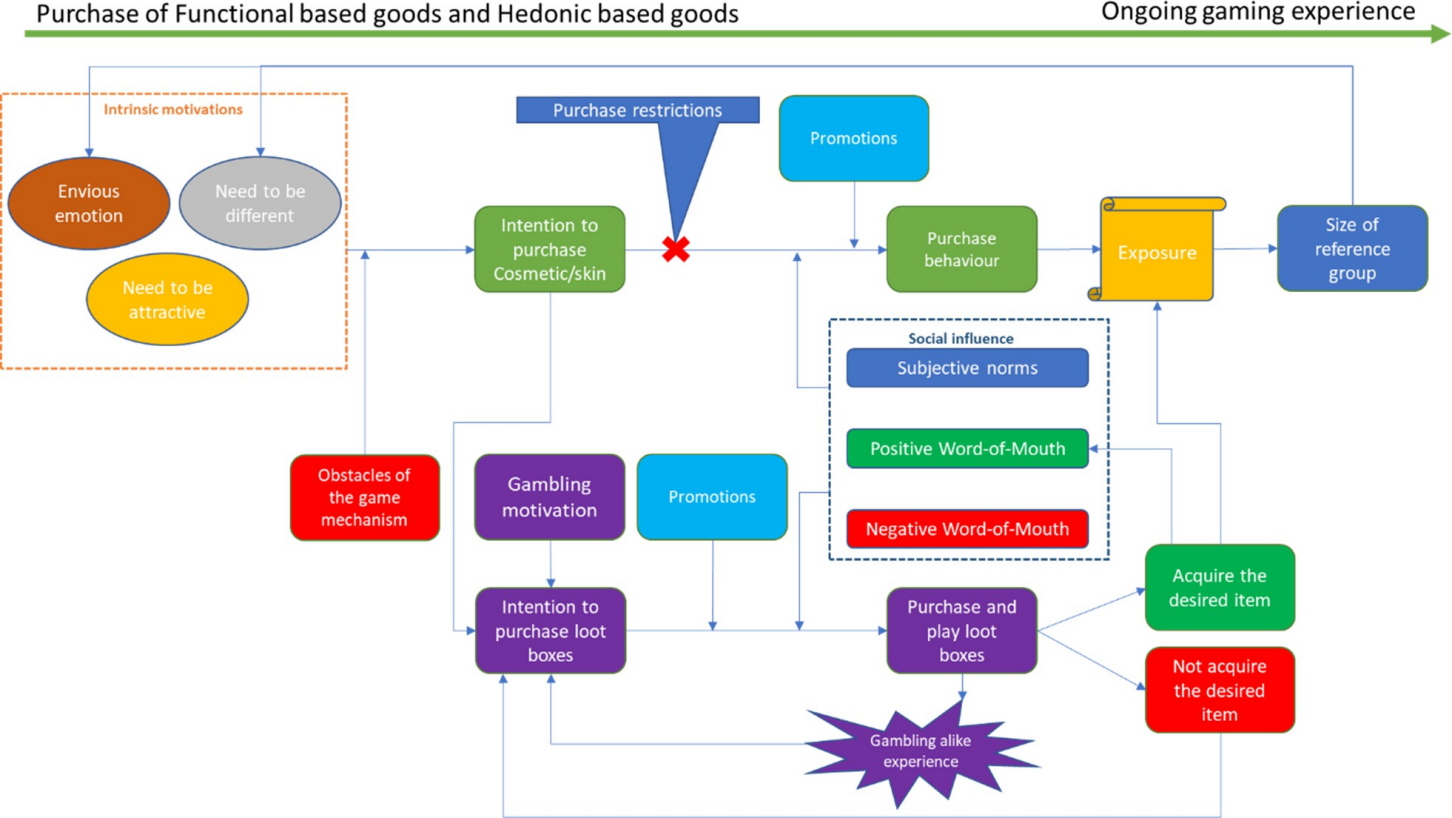
Purchase process of cosmetic/skins, with the existence of loot boxes.

The purchase process of ornamental-based goods is illustrated in Fig 6.

## 6. Discussion

In this study, we classified six types of in-game goods into three categories: functional-based goods, probability-based goods, and ornamental-based goods. After acknowledging the heterogeneity within each category of in-game goods, we used grounded theory to explore why and how video game players purchased each category of in-game goods. According to our conceptual framework, in-game goods purchase behaviour is a response to discomfort from external stimuli. Specifically, we found three players’ motivational patterns to response the discomfort: purchasing functional-based goods to achieve the flow experience, purchasing probability-based goods to unlock the restrictions, and purchasing ornamental-based goods for exposure in the virtual world. We also found three behavioural patterns of players when they purchase each of the three categories of in-game goods.

According to our findings, players’ purchase of functional-based goods is a response to a negative mental state (anxious and boring experience) caused by an external stimulus (Imbalance of challenge and skill). Moreover, the flow state is not stable and can be terminated by anxious or boring experiences, external interferences or physiological needs. This finding broadens the existing knowledge. Previous findings from two survey-based research show that the flow experience is positively related to purchase intention of in-game goods [23,67], while our theory considers that the purchase intention is driven by the desire to reach the flow state. A possible explanation for this discrepancy may be that the results of Animesh et al. (2017) [23] and Huang et al. (2017) [67] described a specific fragment of the purchase process of functional-based in-game goods: After having the flow experience, players re-enter the discomfort state due to the imbalance of challenges and skills, at which point they regain the intention to purchase in-game goods. Thus, we call for future research to verify the relationship between the purchase intention of in-game goods and the flow experience using experimental research designs. Our theory further shows that players purchase in-game goods when there is a sharp imbalance between challenges and skills; otherwise, they would consider acquiring the items through the game mechanism. This finding explains an unusual result in the study of Hamari et al. (2017) [6]: The empirical results of their study show that the unlocking content is not significantly associated with the amount of money spent. A very plausible explanation is that some players unlock in-game contents through game mechanics instead of spending real money to purchase them. In addition, according to our theory, anxious experience and boring experience only affect players’ purchase behaviour of functional-based goods without affecting the case of ornamental-based goods. Our results differ from the results of Bae et al. (2019) [17], in which researchers found while bored players intended to purchase functional-based goods, anxious players intended to purchase ornamental-based goods. This discrepancy may be related to the origin of anxiety and boredom. Whereas, in our study, both the anxious and boring experiences were derived from the video game, in the experimental design of Bae et al. (2019) [17] these mental states were derived from the factors not related to the video game. Thus, we would encourage future research on the relationship between in-game goods purchase behaviour and anxious/boring experiences with different origins. Our theory also explains other counterintuitive phenomena observed in previous studies. In the first place, researchers found that increasing the quality of a freemium game had surprisingly little effect on the demand for premium services directly [3]. A possible explanation is that due to the high quality of the game, players are always in the flow zone, where the challenges and skills are well balanced, which prevents them from having the intention to purchase in-game goods. In the second place, researchers found that regardless of how satisfied players were with the game itself, they did not necessarily have the intention to purchase in-game goods [26]. The explanation could be similar: the increase in satisfaction triggered by the flow experience does not directly enhance purchase intention unless the balance between challenges and skills is broken.

We also argue that the purchase of probability-based goods is a compromise due to purchase restrictions, and the behaviour is stimulated by gambling motivation and gambling experience. On the one hand, our findings are consistent with previous empirical evidence, which show that gambling motivation is positively related to the intention to purchase probability-based in-game goods [30]. On the other hand, we found that players did not purchase probability-based goods completely voluntarily. It is the purchase restrictions that prevent players from purchasing functional-based goods through a direct way and encourages them to participate the lottery. We suggest future researchers study the short- and long-term effects of inducing players to purchase probability-based goods by restricting direct purchase, especially in terms of player satisfaction and loyalty to the game itself. Our findings also echo those of Drummond and Sauer (2018) [66]: our informants considered that probability-based goods were psychologically very close to gambling. Moreover, we observed a dilemma: the profit-maximising strategy of blocking the direct purchase of functional items to force the purchase of probability-based goods can backfire, since players may abandon the game on finding that a loot box does not contain the item on which they were relying to take them out of the discomfort zone. Future researchers might try to find the balance point between profit maximisation and user retention for video game companies employing this strategy.

Our findings also show that the purchase of ornamental-based goods is a synergism of intrinsic motivations and exposure. Envy, the need to be different, and the need to be attractive, are three factors that lead players to purchase ornamental-based goods in video games. Moreover, the use of ornamental-based goods inevitably increases their exposure, which consequently leads to a bigger size of the reference group. Then, a bigger size of the reference group simultaneously increases the envious emotion and the need to be different. At the same time, envy will be greater within a smaller reference group, where the coveted item would be even rarer. The larger the reference group, the sooner the item loses its rarity value, thereby increasing the purchase intention for some other item to satisfy the need to be different. This result is partially consistent with previous empirical evidence [18], which reveals that when players can see the in-game goods inside the virtual world, they are more like to adopt these goods, which consequently leads to further exposure of these goods. Due to the limitation of the qualitative method, we are unable to estimate the effect of the relationship between the intention to purchase ornamental-based goods and these three variables. Thus, future researchers should conduct quantitative studies to confirm the relationship between the size of the reference group, the envious emotion, and the need to be different.

Apart from the mentioned three purchase processes of in-game goods, our research also highlights the importance of social influence on purchase intention: while subjective norms and positive WOM reinforce the purchase intention, negative WOM weakens the purchase intention. These results are consistent with those of Guo and Barnes (2009) [11], who found that players’ decision making was likely to be influenced by other players.

## 7. Implications

Our research has several academic and practical implications. From the academic side, first, our research provides, up to our knowledge, the first native research of in-game goods purchase that is grounded by qualitative data in the context of video games. Meanwhile, our study provides researchers with new directions for the exploration of players’ in-game purchase behaviour. Each of the major theoretical categories mentioned in this study, such as the role of flow experience in the in-game goods purchase process, warrants further investigation. Second, our research highlights the different nature of distinct categories of in-game goods. The results of our study clarify the purchase processes for different types of in-game goods, which is expected to change the prevailing approach in video game research, which is to treat in-game goods as a homogeneous concept.

In terms of the practical implications, based on our results, we have detected some common approaches in the videogame industry which turn out to be not advisable. First, a frequent current practice among video game operators, especially those of freemium games, is to increase challenges or establish obstacle to limit players’ skills in the game, and the purpose of doing this is to push players away from their comfort zone and profit from players’ response: the purchase functional in-game goods or probability-based in-game goods to acquire functional in-game goods. However, the results of our research show that this approach has its danger: prolonged or frequent bouts of anxiety or boredom may lead players to abandon the game permanently. To sustain their profits, therefore, videogame companies should avoid setting the game challenge to an extremely high level or excessively cramping players’ skill development in the game. Second, sometimes videogame operators would deliberately reduce the difficulty to obtain some rare cosmetics/skins through a range of different ways, especially during promotional campaigns. We recommend industrial practitioners not to significantly decrease the difficulty of obtaining rare items in their games because a sufficient number of owners could constitute an avoidance group, which would reduce the desirability of the item and place pressure on game developers to devise new items as others lose their rarity value. Limitations

As well as the above implications, this study also has several limitations.

First, this study is grounded on qualitative data obtained by semi-structured interviews through a convenience sample with a small number of players (21 interviews with 14 informants). This implies that the relationships among the categories in our study have not been verified statistically. Consequently, the results of this study might not be generalisable to the whole population of videogame players. Future researchers may gather data from a larger number of players and apply statistical approaches to verify our findings.

Second, the sample of our study is restricted to China. We cannot be sure that our findings are generalisable to other regions and cultures. Further research could verify their applicability in other cultural contexts, such as North America and Western Europe, which are the second and the third largest markets after the Asia-Pacific region [68].

Third, the interviews were conducted through a text messenger (QQ). Although this approach was ideally suited to reach Chinese players, it also deprived us of the opportunity to capture players’ body languages and facial expressions during the interviews. Thus, future researchers could conduct the similar study in a cultural setting where face-to-face interviews are more socially acceptable, which may generate fresh insights to the theory.

## Supporting information

S1 File (ZIP)
