# Supplementary material for: A grounded theory approach to understanding in-game goods purchase
Source: PLoS One. 2022 Jan 27;17(1):e0262998. doi: 10.1371/journal.pone.0262998 (PMC8794092; doi:10.1371/journal.pone.0262998)
Supplement: S1 File — (ZIP) [file pone.0262998.s001.zip › Transcript 1.pdf]

Interview: 001

Informant: 001

Please note that the original transcript is in Simplified Chinese. The English translation is for internal communication among the author of this research, and it is not proofread. Potential linguistic errors may exist in the English translation.

Researcher 9:49:15
Thank you for your willingness to participate and be interviewed here. My name is XXX XXX, and I'm a PhD student in the XXX University of XXX(XXX). Currently, I'm working on a research project which focuses on videogame players' purchase motivations of in-game goods. Throughout this interview, I will ask you a series of questions and you are encouraged to express your opinions freely with emoticons. If I have questions about what you've said or need clarification about a topic or concept, I'll ask you.
感谢您愿意参加并在此接受采访。 我叫 XXX，我是市场营销学的博士生，现在我在 XXX 大学就读。 目前，我正在开展一个研究项目，专注于电子游戏玩家对游戏内购买项目的购买动机。 在整个访谈中，我会问您一系列问题，我们鼓励您自由表达您的意见和观点。因为这不是一个当面访谈，所以我们也鼓励您用 QQ 表情来表达您的情绪。 在访谈过程中，如果我对你所说的内容有疑问或需要您澄清一个主题或概念，我会问您。

Researcher 9:49:27
Are you ready?
您准备好了吗？

Informant 001 9:49:42
Yes
好了

Researcher 9:50:09
In the previous survey, you mentioned that you purchased certain types of in-game purchases.
在之前的调查问卷中，您已经提到您购买了某些类型的游戏内购买项目。

Researcher 9:50:16
What is your motivation for purchasing a decorative / skin in-game purchase?
请问您购买装饰/皮肤类游戏内购买项目的动机是什么？

Informant 001 9:50:34
They look good
外观好看

Researcher 9:51:15

Can you tell me more about this?
您能就这个再说点细节吗？

Informant 001 9:53:43
When the game itself is set, the appearance of the character is uniform. Only by using the gold coin mechanism in the game or using money to purchase can change the appearance of the character and increase the playability of the game.
游戏本身设定的时候人物的外观是统一的，只有通过使用游戏里的金币机制或者使用金钱来购买才能改变人物的外观，增加游戏的可玩性

Researcher 9:55:15
Ok, how do you understand the word playability?
好的，您怎么理解可玩性这个词？

Informant 001 9:57:21
Make people have more skin choices and increase the visual sense without breaking the balance of the game
在不打破游戏平衡的情况下使人物有更多的皮肤选择 增加视觉感

Researcher 9:58:49
Ok. In other words, you think that changing the appearance of the characters and improving the playability of the game is linked. Is this correct?
ok。也就是说您认为改变人物外观和提高游戏可玩性是挂钩的，我这样理解对吗？

Informant 001 9:59:12
Yes
可以

Researcher 9:59:38
Ok, let's talk about the next topic.
好的，我们来说下一个话题

Researcher 10:02:45
How do you usually buy in-game purchases for decorative / skin type? Please tell me a general process.
您通常怎么样购买装饰/皮肤类的游戏内购？ 请告诉我一个一般流程。

Informant 001 10:03:33
Redeem the game through the gold coins of the game
通过游戏的金币去游戏的商店兑换

Informant 001 10:04:08
The gold coins of the game are given through the game task
游戏的金币是通过游戏任务给予

Researcher 10:04:10
In this case, is the gold coin in the game acquired through the in-game mechanism or purchased with real money?
在这种情况下游戏内的金币是通过游戏内机制获得的还是用真钱购买的呢？

Researcher 10:04:15
OK
好的。
Researcher 10:04:45
Have you ever used real money to purchase the decoration or skin in the game?
您有没有用过真钱来购买过游戏内的装饰或者皮肤？

Informant 001 10:06:34
Yes, real money can buy the gems in the game and then redeem them through the jewels. Different games use real money to buy them in different ways, but the purpose is basically the same.
也有，真钱可以购买游戏里的宝石然后通过宝石再兑换宝箱，不同的游戏用真钱购买的方式都有所不同，但是目的基本上都是一样的

Researcher 10:07:32
So generally speaking, you are using real money to buy the currency of the game first, and then use these virtual currencies to buy various products in the game?
所以一般来说您都是用真钱先购买游戏内的货币，再用这些虚拟货币购买游戏内的各种商品？

Informant 001 10:07:50
It can also be understood like this.
也可以这么理解

Researcher 10:09:06
Ok. So in this case, is your process of purchasing various in-game purchases similar in every case?
好的。那么在这种情况下，您购买各种游戏内购的过程都差不多吗？

Researcher 10:09:14
For example, is the process of buying a Cosmetic/Skins type in-game good and a Loot box type in-game good purchase the same?
比如您在购买装饰/皮肤类的内购和抽奖箱类的内购的过程都是一样的吗？

Informant 001 10:10:19
The process is the same, but the ways are different.

过程一样只是方式不同

Researcher 10:10:37
How do I understand the expression "different ways"?
我怎么理解“方式不同”这个表达？

Informant 001 10:13:08
One way is the gold coin obtained by completing the tasks in the game in the game. The other way is to buy directly using the real money.
一个是通过游戏里完成游戏里的任务来获得的金币 一个是用过现实中的金钱直接购买

Informant 001 10:13:18
Two ways
两种方式

Researcher 10:14:39
Understand, that is to say: when purchasing Cosmetic/Skins, you usually buy them using currency obtained through in-game mechanism. Moreover, the purchase of the loot box type in-game good is purchased directly from the real money. Am I right?
懂了，也就是说：在购买装饰/皮肤的时候，您一般是通过游戏内机制获取货币购买的。而购买抽奖箱类的内购是直接用现实中的货币直接购买的。我这样说对吗？

Informant 001 10:15:29
Yes
可以

Researcher 10:15:41
Ok. We have just mentioned the in-game purchase of the loot box type in-game good. What is your motivation for purchasing such type in-game good?
好的。我们刚才一直提到抽奖箱类的游戏内购。请问您购买这类内购的动机是什么呢？

Informant 001 10:18:24
Because there are a lot of uncertainties in this way
因为通过这种方式有很多不确定性

Researcher 10:19:30
So, do you think that the experience of uncertainty has led you to buy such in-game good?
所以您认为不确定性的体验导致了您购买这类游戏内购?

Informant 001 10:21:55

This mechanism in the game adds the playability of the game, just like gambling, spending very little money to win something better.
游戏内的这种机制添加了游戏的可玩性，好比赌博一样花很少的钱去赢得更好的东西

Researcher 10:24:04
Ok, so when you buy a loot box type in-game good, do you often search for specific information about such products? For example, get more information from your friends or the official website of game?
好的，那么在购买抽奖箱类的游戏内购的时候，您是否经常搜索这类商品的具体信息？ 例如，从您的朋友或游戏的官方网站那边获取更多信息？

Informant 001 10:25:10
In the game, there are built-in table for getting items. The information can be viewed there.
游戏内部都有自带的为获取物品浏览表 可以通过这里来查看

Researcher 10:25:35
So this is the only way for your to get the information of the loot box type in-game good, right?
所以这个是您获取抽奖箱类的游戏内购信息的唯一途径，对吗？

Informant 001 10:26:13
Yes
对的

Researcher 10:27:04
Then, when buying cosmetic/Skins type in-game good, do you often search for specific information about such products?
那么，那么在购买装饰/皮肤类的内购的时候，您是否也经常搜索这类商品的具体信息？

Informant 001 10:28:00
Yes
有

Researcher 10:29:02
So, when you buy a Cosmetic/Skins type in-app purchase, in addition to obtaining the official information of the operator in the game, is there any other channel to obtain information?
所以您在购买购买装饰/皮肤类的内购的时候，除了游戏内获取运营商的官方信息外，还有通过其它的渠道获取信息吗？

Informant 001 10:29:49

At this moment, no.
目前没有

Researcher 10:31:21
Ok. So , do you often evaluate alternative offers during the purchasing process?
好的。那么您在装饰/皮肤类内购的购买过程中，您是否经常评估这类商品的替代商品？
Informant 001 10:34:03
No, these virtual items have the same skin except for the same color, and there are no other special functions, so there is no difference.
没有，这些虚拟物品同一款皮肤除了颜色不一样其他都一样，也没有其他特殊的功能，所以有和没有都是一样的

Researcher 10:35:24
I understand. So, do you often evaluate alternative offers during the purchasing process of the loot boxes?
我明白了。那么您在抽奖箱类内购的购买过程中，您是否经常评估这类商品的替代商品？

Informant 001 10:37:53
If I have something I really want, I could redeem the currency in the game using real money and buy them directly. So there is no such process of evaluating the products.
如果有特别想要的东西 可以直接用钱购买游戏里的货币来直接购买，所以没有评估商品的替代商品一说

Researcher 10:38:40
Ok , this means that when you buy these two types of in-game goods, you never evaluate other alternative offers. Is this correct?
ok，也就是说您在购买这两类游戏内购的时候，从来都不评估其它的可替代方案，这样说对吗？

Informant 001 10:39:06
Yes
对

Researcher 10:39:30
We know that there are different types of in-game goods. When you purchase in-game goods, have you got a priority order. in mind which indicates you to buy certain types of goods first than others?
好的。我们知道有不同类型的游戏内商品。 当您购买游戏内商品时，您是否心里有一个优先顺序。比如比起一类游戏内商品您会优先购买另一类商品？

Informant 001 10:41:48
There are many things you can buy in the game, such as skin. Voice, spray, and mounts.

But my first consideration should be skin.
游戏内有很多可以购买的比如皮肤 语音 喷涂 还有坐骑 但是我第一考虑的应该是皮肤

Informant 001 10:42:12
Others can be obtained by loot boxes.
其他的可以通过抽奖方式来获得
Informant 001 10:43:04
But there are also special circumstances, such as when there are other items that you want. I still will buy them through the game gold coins
但也有特殊情况，比如有特别想要的其他物品时 还是会第一时间通过游戏金币来购买

Researcher 10:43:42
Generally, in which circumstance this special situation happens?
请问特别想要的情况一般是在什么场合下发生？

Informant 001 10:45:09
For example, some games will introduce new skins and mounts from time to time. Once you have seem the items you want, you will go directly to buy them.
比如游戏会不定期出来新的皮肤和坐骑，一旦有自己看的上的物品出现时就会去直接购买

Researcher 10:46:15
In other words, apart from the loot boxes, which give you the uncertainty experience like gambling, you still want othertypes of game items, right?
也就是说，您购买抽奖箱类内购除了体验不确定性，像赌博那样的刺激感，您还想要获得其它类型的游戏物品，对吗？

Informant 001 10:47:09
Yes
对

Researcher 10:47:29
However, when you really want certain items, you will still prefer to buy them directly with real money. Is that true?
但是，有特别想要的物品的时候，您还是会优先选择直接用真钱购买，是这样吗？

Informant 001 10:49:39
Yes
对

Researcher 10:50:10
Ok. In addition, for the voice, spray, and mount you have just mentioned, are these

items functional or non-functional, just like skins?
好的。另外，您刚才提到的语音，喷涂，还有坐骑。这些是功能性的道具还是非功能性的，像皮肤那样的道具呢？

Informant 001 10:51:10
They are non-functional
都是非功能性的

Researcher 10:51:24
These are all the questions. Thank you very much for participating in our research. Please confirm that your email address is XXXXXX@XXXXXX.com, because later we will send the JD electronic gift card to this address.
这就是全部的问题。 非常感谢您参与我们的研究。请确认您的电子邮件地址是XXXXXX@XXXXXX.com，因为稍后我们把京东电子礼品卡发送到这个地址。
