# Supplementary material for: A grounded theory approach to understanding in-game goods purchase
Source: PLoS One. 2022 Jan 27;17(1):e0262998. doi: 10.1371/journal.pone.0262998 (PMC8794092; doi:10.1371/journal.pone.0262998)
Supplement: S1 File — (ZIP) [file pone.0262998.s001.zip › Transcript 10.pdf]

Interview: 010

Informant: Informant 011

Please note that the original transcript is in Simplified Chinese. The English translation is for internal communication among the author of this research, and it is not proofread. Potential linguistic errors may exist in the English translation.

Researcher 13:32:22
Thank you for your willingness to participate and be interviewed here. My name is XXX XXX, and I'm a PhD student in the XXX University of XXX(XXX). Currently, I'm working on a research project which focuses on videogame players' purchase motivations of in-game goods. Throughout this interview, I will ask you a series of questions and you are encouraged to express your opinions freely with emoticons. If I have questions about what you've said or need clarification about a topic or concept, I'll ask you.
感谢您愿意参加并在此接受采访。 我叫 XXX，我是市场营销学的博士生，现在我在 XXX 大学就读。 目前，我正在开展一个研究项目，专注于电子游戏玩家对游戏内购买项目的购买动机。 在整个访谈中，我会问您一系列问题，我们鼓励您自由表达您的意见和观点。因为这不是一个当面访谈，所以我们也鼓励您用 QQ 表情来表达您的情绪。 在访谈过程中，如果我对你所说的内容有疑问或需要您澄清一个主题或概念，我会问您。

Researcher 13:32:29
Are you ready?
您准备好了吗？

Informant 011 13:32:54
Ok
Ok

Researcher 13:32:57
Yes, I'm ready
请问您贵姓？

Informant 011 13:33:03
Chen
陈

Researcher 13:33:19
Ok Mr. Chen, are you taking this interview at home?
好的陈先生，您是在家里接受这个访谈吗？

Informant 011 13:33:24
Yes.

是的

Researcher 13:33:41
Ok :). In the previous survey, you mentioned that you purchased certain types of in-game purchases, including Power-ups, Expansion package, Playable characters, Cosmetic/Skins, Loot boxes, and Time-saver.
好的：）。在之前的调查问卷中，您已经提到您购买了某些类型的游戏内购买项目，包括增强道具，扩展包，可游玩的角色，装饰/皮肤，抽奖箱，以及省时道具。

Researcher 13:33:45
What are your motivations for purchasing Power-ups type in-game goods?
请问您购买增强道具游戏内购买项目的动机是什么

Informant 011 13:33:56
Impulsively buying.
冲动消费

Researcher 13:34:15
Can you tell me more about this?
您能就这个再说点细节吗？

Researcher 13:34:26
For example, in which circumstances such impulsively buying occurs
比如在哪种情况下会发生这样的冲动消费

Informant 011 13:34:42
Premise: I am interested in this game
前提，这个游戏令我感兴趣

Informant 011 13:35:01
Secondly, the in-game goods make me want to buy
其次，内购的东西，让我忍不住想买

Informant 011 13:35:08
For example
比如说

Informant 011 13:35:28
Time-limited characters, skin, out-of-print items, etc.
限时的人物，皮肤，绝版道具等

Informant 011 13:35:36
Which may cause me a certain degree of impulsive consumption.
会造成我一定程度上的冲动消费

Researcher 13:36:10
I see.
双击查看原图原来如此。

Researcher 13:36:47
I noticed that some of the items you mentioned here are limited editions. Is this related to your impulsive consumption?
我注意到这边您说的有一些道具是限量版的，这和您冲动消费有关联吗？

Informant 011 13:37:17
There is a strong association.
有很大一部分关联

Researcher 13:38:02
What is the difference of your mindset when buying these limited-edition items and unlimited edition items?
请问在购买这些限量版道具的时候，您的心态和购买一般不限量的道具时候有什么差异？

Informant 011 13:39:04
The limited edition makes people think that if you miss it, you have to wait for a long time or there will never be anymore. Unlimited-edition items are available all the time, and I would consider more the wallet.
限量版的会让人觉得，错过了就要等好久或者没有了，不限量的时候任何时间都有，可能会比较考虑钱包

Researcher 13:39:45
I see. I also sometimes have the same feeling.

原来如此。我也经常有这样的感觉。

Researcher 13:40:19
In addition, I would like to know what changes in your mood when you are spending impulsively?
另外，我想知道一下您在冲动消费的时候，情绪方面会有什么变化?

Informant 011 13:41:29
Starting from the discovery of the limited-edition items, surprise → impulsive consumption → feeling very cool → very regret
从发现更新了限量道具开始，惊喜→冲动消费→感觉很爽→很后悔

Researcher 13:42:52

I think this process is very interesting. In the game, what kind of situations will give you a pleasant surprise?
我觉得这个过程很有趣诶。在游戏内，哪些情况下会给您很惊喜的感觉呢？

Informant 011 13:44:06
For example, a character which I have always been wanting. I missed that the last time, and this time it is finally there again.
比如一直很想要的一个人物，错过了上次，这次终于重新上架了

Informant 011 13:44:11
There will be a feeling of surprise
会有种惊喜的感觉

Researcher 13:44:55
Ah, I understand. I thought so when I missed the discount on PSN.
啊我懂了。我在 PSN 上错过打折的时候也这么想。

Informant 011 13:45:32
Yes.
是的

Researcher 13:45:36
In addition, in the process of feeling very cool, what specific mentality or behaviour is different from the general situation?
另外，在感觉很爽的过程中，您具体有哪些心态上或者行为上和一般情况下不一样的地方？

Informant 011 13:46:08
Mentally, it should be satisfaction.
心态上，应该算满足感

Informant 011 13:46:23
The behaviour of course should be: Using the limited-edition items as soon as possible.
行为当然是赶紧去使用限量道具

Researcher 13:46:47
Will there be any increase in gaming time after purchase?
会不会有购买后增加游玩时间的情况呢？

Informant 011 13:47:23
There will be. The game in which I consumed makes me more reluctant to leave.
会有的，消费过的游戏会更让我不舍

Researcher 13:48:46

It turned out to be the case. I am very interested in the process of "very regrettable" that you just said. As we said earlier, in the process of feeling very cool, it will be very satisfying. But why did you regret it at the end?
原来是这样。其次，我对您刚才说的“很后悔”这个过程很有兴趣。刚才我们说到了，在感觉很爽的过程中，会很满足。但是为什么最后又很后悔呢？

Informant 011 13:49:18
Because after experiencing cool and satisfaction,
因为在爽完，满足完之后

Informant 011 13:49:23
the wallet is found to be empty.
发现钱包空了

Informant 011 13:49:29
Of course I regret it.
当然就后悔了

Researcher 13:50:17
It turned out to be the case. Is this behavior mode only applicable to in-game goods such as Power-ups? Or it's still applicable to all types of in-game goods?
原来如此。这个行为模式只对增强道具这一类的内购还是适用呢？还是对于所有类型的游戏内购都适用？

Informant 011 13:50:34
All the types.
所有类型

Researcher 13:51:30
Ok, the impulsively buying is the only reason for all types of in-game purchase? What other reason do you need to add?
好的，您对于所有类型的游戏内购的购买原因都是冲动消费这一个吗？您还有什么其它的原因需要补充吗？

Researcher 13:52:22
Here the in-game goods refer to Power-ups, Expansion packages, playable characters, Cosmetics/skins, Loot-boxes, and Time-savers.
这边说的游戏内购指的是增强道具，扩展包，可游玩的角色，装饰/皮肤，抽奖箱，以及省时道具

Informant 011 13:52:39
One more is the love to the game.
还有就是，对游戏的喜爱

Informant 011 13:53:06
If a game allows me to feel its attentive development, production, and operation,
如果这款游戏让我能感觉到开发和制作以及运行上的用心

Informant 011 13:53:23
and I could play without in-game goods,
即便没有内购的必要也能玩

Informant 011 13:53:40
I would also purchase and consume items in a supportive way.
我也会以支持的方式购买消费道具

Researcher 13:54:40
I see. In this case, can I understand that it is more to support the game development team than to get the in-game goods themselves?
原来如此。在这种情况下，我能不能理解为比起获得内购本身，更多的是为了支持这个游戏开发团队？

Informant 011 13:55:02
In contrast, I hate the games and and their operators who force players to buy game items by increasing the time-limit activities, launching frequently limited-time items, and increasing the difficulty.
相对的，我很讨厌提高限时活动难度，频繁推出限时道具，提高难度逼迫玩家购买消费道具的游戏和运营商

Informant 011 13:55:24
In different circumstances, there are different states of mind.
在不同的情况下，是不同的心境

Informant 011 13:55:45
Facing different games, I may purchase impulsively or supportively.
面对不同的游戏，我可能是冲动消费，也有可能是支持消费

Researcher 13:57:07
I see, I understand. So, the relatively free consuming environment is important to you?
原来如此，我懂了。所以相对自由的消费环境对您来说很重要？

Informant 011 13:57:27
Yes
是的

Informant 011 13:57:47
A non-free consuming environment could ruin a good game.
不自由的消费环境会毁掉一款好游戏

Informant 011 13:58:01
which is also a behaviour of profaning game maker's artwork.
也是褒渎游戏制作者的艺术品的行为

Researcher 13:58:37
Ok. What are the general conditions for the games you like?
好的。一般您喜欢的那类游戏一般要满足哪些条件？

Informant 011 13:59:52
Excellent game content with excellent operators. Only these two points need to be met, but currently there are very few in the market that can meet these two points , especially the latter.
优秀的游戏内容配上优秀的运营商，只需要满足这两点，但目前市面上能满足这两点的十分少，特别是后者

Researcher 14:01:39
Ok. I see that you generally play games on Windows and Android platforms. On which platform do you usually have more games you like?
好的。我看到您一般在 Windows 和安卓平台上玩游戏，一般在哪个平台上有更多您喜欢的游戏？

Informant 011 14:02:37
On both.
这个都有

Informant 011 14:02:51
More or less, on each platform there are excellent games.
没有多少之分，每个平台都有优秀的游戏

Researcher 14:03:49
Ok. Let's change the subject. How do you usually buy in-game goods? Please tell me a general process.
好的。我们换个话题。您通常怎么样购买游戏内购？ 请告诉我一个一般流程。

Informant 011 14:04:44
Buy directly through the in-game purchasing channel.
直接通过游戏内部的购买渠道进行购买

Researcher 14:05:20
Ok.
好的。

Researcher 14:05:21

In the course of purchasing in-game goods, from which channels do you generally know about the existence of in-game goods?
在游戏内商品的购买过程中，您一般从哪些渠道了解这些内购的存在？

Informant 011 14:06:06
Promotions of the internal bulletin board in the game, Weibo, WeChat accounts, notices from friends.
游戏内部公告栏的宣传，微博，微信公众号，朋友告知

Researcher 14:06:53
Ok, generally, in which circumstances your friends would tell you?
好的，这边所说的朋友告知一般是在什么场景下发生的？

Informant 011 14:07:33
QQ group, WeChat group, usually social discussion
QQ 群，微信群，平时社交讨论提起

Researcher 14:07:52
The social discussion refers to the offline communication, right?
平时社交讨论指的是线下对吧？

Informant 011 14:08:08
Yes
是的

Researcher 14:08:22
I see. When you purchase in-game goods, will you evaluate the alternative solutions of in-game goods? For example, acquiring the same item in a free way?
好的，我们继续。您在游戏内商品的购买过程中，您是否经常评估游戏内商品的替代方案？比如用免费的方式去获取道具？

Informant 011 14:08:46
Yes.
是的

Informant 011 14:09:10
This is necessary because not all players have sufficient funds.
这是必要的，因为不是所有玩家都有充裕的资金

Researcher 14:10:17
You mentioned that when you buy, you will evaluate the plan to acquire items in a free way. However, why would you still buy the in-game goods?
您提到在购买的时候，的确会评估用免费的方案去获取道具的方案，但是为什么还是会购买内购呢?

Informant 011 14:10:43
Because of love, impulsive consumption.
因为喜爱，冲动消费

Researcher 14:13:40
Ok. I see. So what would you do for those games that you don't like?
好的。原来是这样。那么对于哪些您刚才说的不喜欢的游戏呢？

Informant 011 14:14:04
For the game I don't like, of course I am not going to play.
不喜欢的游戏，当然不会去玩了

Informant 011 14:14:13
Neither am I going to consume.
更不会去消费

Researcher 14:15:55
That's it, that is, your preference for the game depends on two factors. The first is the quality of the game itself, and the second is the way operators sell the in-game goods. Is that true?
原来如此，也就是说，您对于游戏的喜好取决于两个因素。第一个是游戏本身的素质，第二是厂商销售游戏内购的方式，是这样吗？

Informant 011 14:16:04
Yes.
是的

Researcher 14:17:26
Yes. Are these two points indispensable to meet your definition of a good game?
好的。这两点是不是缺一不可才能满足您心中定义的好游戏？

Informant 011 14:17:45
Yes.
是的

Informant 011 14:17:56
It's like horse racing
这就好比赛马

Informant 011 14:18:05
Good horse with a good rider
好的马配上好的骑手

Researcher 14:20:27
Ok, I almost finished asking. Do you have anything else to add?
好的，我差不多问完了。您还有什么其它的想补充的吗？

Informant 011 14:21:07
I hope this research will go smoothly and bring you some help, thank you.
希望这次调研能顺利并且给你带来一定程度上的帮助，谢谢

Researcher 14:22:05
Thank you very much. These are all the questions. Thank you very much for participating in our research. Please confirm that your email address is XXXXXX@XXXXXX.com, because later we will send the JD electronic gift card to this address.

太谢谢了。这就是全部的问题。 非常感谢您参与我们的研究。请确认您的电子邮件地址是 XXXXXX@XXXXXX.com，因为稍后我们把京东电子礼品卡发送到这个地址。
