# Supplementary material for: A grounded theory approach to understanding in-game goods purchase
Source: PLoS One. 2022 Jan 27;17(1):e0262998. doi: 10.1371/journal.pone.0262998 (PMC8794092; doi:10.1371/journal.pone.0262998)
Supplement: S1 File — (ZIP) [file pone.0262998.s001.zip › Transcript 11.pdf]

Interview: 011

Informant: Informant 007

Please note that the original transcript is in Simplified Chinese. The English translation is for internal communication among the author of this research, and it is not proofread. Potential linguistic errors may exist in the English translation.

Researcher 7:59:06
Thank you for your willingness to participate and be interviewed here. My name is XXX XXX, and I'm a PhD student in the XXX University of XXX(XXX). Currently, I'm working on a research project which focuses on videogame players' purchase motivations of in-game goods. Throughout this interview, I will ask you a series of questions and you are encouraged to express your opinions freely with emoticons. If I have questions about what you've said or need clarification about a topic or concept, I'll ask you.
感谢您愿意参加并在此接受采访。 我叫 XXX，我是市场营销学的博士生，现在我在 XXX 大学就读。 目前，我正在开展一个研究项目，专注于电子游戏玩家对游戏内购买项目的购买动机。 在整个访谈中，我会问您一系列问题，我们鼓励您自由表达您的意见和观点。因为这不是一个当面访谈，所以我们也鼓励您用 QQ 表情来表达您的情绪。 在访谈过程中，如果我对你所说的内容有疑问或需要您澄清一个主题或概念，我会问您。

Researcher 7:59:11
Are you ready?
您准备好了吗？

Informant 007 7:59:58
Yes. Ok.
嗯 好的

Researcher 8:00:05
"Flow experience" has been used by psychologist to describe a state of mind experienced by people who are deeply involved in an activity. Instance, sometimes while playing videogames, the player's action and awareness are merged, and he/she is totally connected on the gaming tasks at hand. In this state, the player loses his/her consciousness, and his/her perception of time becomes faster or slower than usual. Also, the player perceives a feeling of being in control, which empowers him/her from the fear of failure.
心理学家使用"心流体验"来描述深度参与某项活动的人所经历的心理状态。 例如，有时玩家在玩电子游戏时，他/她的动作和意识会融为一体，并且他/她完全关注手头的游戏任务。 在这种状态下，玩家失去他/她的自我意识，他/她对时间的感知变得比平时更快或更慢。 此外，玩家会感受到一种掌控全局的感觉，这使他/她免于对失败的恐惧。

Researcher 8:00:13
Think about your own gaming experience for a moment. Have you ever experienced flow while playing videogames?
请回想一下您自己的游戏经验。 您玩电子游戏时有没有经历过心流体验？

Informant 007 8:01:35
This is the case when playing a moba games.
在玩 moba 类游戏时 会有这类情况

Researcher 8:02:15
Is there only this type of game? Is this not the case when playing other game types?
只有这一类游戏吗?其它游戏类型不存在这种情况吗？

Informant 007 8:02:42
对我而言 只有这类情况会有

Researcher 8:02:59
Please tell me what happened when you came to the flow state? I mean your behavioural and psychological activities during this course.
好的。请告诉我您在进入到心流体验的时候发生了什么？ 我的意思是您在这个过程中的行为和心理活动。

Informant 007 8:03:25
Other games may not be too restrictive, so I'm not focused during the whole process.
其他游戏 可能会因为没有太多的限制 所以不是全程都全神贯注的

Researcher 8:04:08
I see. What do you mean "too restrictive" ?
原来如此。这边说的"太多的限制"主要体现在那些方面呢？

Informant 007 8:08:46
moba type games emphasise on the competition among players. At the same time, the final victory is determined by players' own skills, peer cooperation, and strategic situation and layout. In contrast, in some stand-alone games or "Amaranth cutting" online games, what you need to do is doing monotonous tasks without having a loft of "activities". Even if you leave the computer, it will not affect the whole situation. When playing a moba game with headphones, I am isolated from the outside world. I feel that the time passes very fast, but I don't have the conscious. After entering the game, I'm also integrated into the game character. Because it's a Real-time strategy game and it has many uncertain factors, my mental movements are very frequent.
moba 类游戏 因为是玩家与玩家之间进行竞争 在相同的时间内凭借自己的技术，同伴的合作，局势走向和布局安排，来决定最终的胜利归属。而一些单机游戏，或“割韭菜”网游，你只要单一做任务就可以了，并不需要很多的“活动”，就

算你离开电脑，也不会影响整体。在进行 moba 类游戏时，同时带上耳机，与外界隔绝，会觉得时间过得非常快，而自己本身缺没有意识到。进入游戏后，自己也融入了游戏角色内。因为是即时游戏，存在很多不确定因素，心里活动会很频繁。

Informant 007 8:09:58
The writing is a bit messy. I just talk what I think.
写得有些乱　只是想到什么说什么

Researcher 8:10:36
I see. Don’t worry. I can understand :)
原来如此。不要紧的，我能看明白：）

Researcher 8:11:05
We talked about the fun of the game in the last interview, you talked about “Personally, I think if the playability of the game doesn't reach my expectation, there is no place for the fun. Similarly, I will search for the games which correspond to the fun I define. So, during the game design process, they must grab buyers' heart, don't they? The "fun" of the buyers must be reflected in the game.”
我们在上一个访谈内谈到了游戏的乐趣，您谈到“我个人认为噢，单单针对我个人，如果这款游戏的游戏性不达到我的心里预期　谈何乐趣？同样的　我也会找符合我乐趣的游戏　所以在游戏设计过程中　肯定要抓住购买者的心里吧？把购买者的“乐趣”在游戏中充分体现出”

Informant 007 8:11:38
Yes.
嗯嗯

Researcher 8:11:42
I want to know if the fun of the game you perceive is related to the flow experience we just said?
我想知道您感知的游戏乐趣和我们刚才说的心流体验有关系吗？

Informant 007 8:12:16
There is associations more or less.
或多或少会存在一些关联

Researcher 8:13:12
Can you say more in detail? For example, if you think that A is the result of B, or if A is included in B.
可以说得详细一些吗？比如说您认为是 A 导致了 B，或者是 A 包含在 B 中这样的关系。

Informant 007 8:16:38

Most of the players of moba type games are males, because most males like the pleasure of being "Real hard fighting" and "Fighting to the last moment and being the winner". This type of games has done it and successfully have attracted the target population. At the same time, these games have good portrayed characters and skills with dazzling special effects, etc., which make player easily enter to the state (also can be said to be integrated into the game).
大多数玩 moba 类游戏的都是男孩子 因为多数男孩子都喜欢这种“真打实干”“拼到最后成为胜利者”的快感。这类游戏全部都做到了。成功的吸引了受众群体。同时这类游戏有着不错的角色技能刻画，炫目的特效等，更能让游玩的人进入状态（也可以说是融入游戏）

Informant 007 8:16:49
It should be said that they complement each other.
应该说是相辅相成的吧

Researcher 8:17:53
I see.
原来如此。我明白了。

Researcher 8:19:07
Another point I am paying attention to is, when I last asked you “So what do you think will happen when you feel that playing games is "fun"? For example, will you increase the gaming time?” Your answer was “There may be some "naive" behaviours. . . Reluctant to clear the game.” “Or delete the file after clearing the stages and start again. ”
另外一个我很注意的点是。当我上次问您“那么您感觉玩游戏有“乐趣”的时候，您还会有什么反应？比如说会增加游玩时间吗？” 您的回答是“可能会有一些“幼稚”行为。。。舍不得通关”“或者通关后 删档 重新开搞。。。”

Researcher 8:19:31
The fun of game cannot be perceived again after clearing the game.
原因是通关后再也体会不到乐趣了

Informant 007 8:19:36
When it comes to the flow experience, I think that it is the concentration of attention. Children are actually more concentrated. Instead, they are less concentrated with the rise of age.
说到心流体验 我认为就是注意力集中不集中 小孩子其实更能集中 反而随着年龄的上升 注意力不那么集中了

Informant 007 8:20:08
Yes yes.
对对

Researcher 8:21:22
So, when you are reluctant to clear a game, have you had flow experience?
原来如此，您在舍不得通关的时候，有没有过心流体验？

Informant 007 8:23:51
Because many games are exaggerated when they are advertised. Actually, the real inside feelings are not very good... If let me choose, I definitely choose to continue playing the existing one instead of looking for the "New World."
I don't whether it's flow experience, but there are some psychological activities. For example, have I missed some stages? Have I missed some treasures? How many branch tasks that I didn't do? etc.
因为很多游戏在宣传的时候 都会夸大 其实内在的真实感受并不是非常好 让我选择 我肯定选择继续玩现有的 而不是去寻找“新大陆”。
心流体验不知道算不算得上，但是会有一些心理活动。比如马上要通关了，我是不是还漏了一些关卡？漏了一些宝物没有拿？支线任务还有多少没有做？等等。

Researcher 8:25:18
Ok. The last time you mentioned that when you are reluctant to clear the game or have the feeling of Good to the last drop, if there is Expansion packages available, you want to buy them.
好的。然后您上次说在舍不得通关的时候，或者对一个游戏意犹未尽的时候，如果有扩展包，您就想购买。

Informant 007 8:25:21
Now many games have explicit and hidden strong lines. Depending on your choices, the endings are different. Actually playing this kind of game should be considered as a kind of flow experience.
现在很多游戏 都会有明线和暗线 根据你的选择不同 结局不同 其实在玩这种游戏 应该也算是一种心流体验吧

Informant 007 8:25:46
Yes.
是的

Researcher 8:26:32
In this case, is the purpose of purchasing an expansion pack to increase the difficulty of the game or to add new challenges?
请问在这种情况下，您购买扩展包的目的是为了提高游戏的难度或者增加新的挑战吗？

Informant 007 8:27:20

Adding new challenges. Forget increasing the difficulty. 😂 It's too self-abuse.

增加新的挑战　增加难度这种我还是算了吧😂太自虐了

Informant 007 8:27:43

I am not a technical geek 😳.

本人并非技术宅😳

Researcher 8:28:39
Ok :) Have you ever had the experience of boredom in the game?
原来如此：）您在游戏时有过无聊的经历吗？

Informant 007 8:29:03
Do you refer to games in general? Or to a specific game?
是指所有游戏？还是单指？

Researcher 8:29:13
Games in general.
所有种类的游戏

Informant 007 8:29:19
Yes.
有

Researcher 8:29:23
Under what circumstances does it usually occur?
通常在什么情况下有？

Informant 007 8:29:27
I would be bored to give up the game.
会无聊到放弃这款游戏

Informant 007 8:30:12
When the difficulty of the game is very high, and when the gaming enters to a very obvious repeated loop.
当游戏难度非常难的时候 还有就是游戏进入一种很显而易见的循环的时候

Researcher 8:31:13
So, when the gaming enters an obvious repeated loop, how is the difficulty of the game at this time?
原来如此，游戏进入一个显而易见的循环的时候您认为这时候的游戏难度怎么样？

Informant 007 8:31:13
If the difficulty is too high, it is not very friendly to some players. It does not raise the challenge. In turn, it will make some people feel disgusted, including myself.
难度太高 对一些玩家就不是很友好 不但不会提高挑战性 反而会让一些人觉得反感 包括我自己

Informant 007 8:32:10
When the game has enterded a loop, it has been irrelevant to the difficulty.
游戏已经进入一种循环的时候 其实已经跟难度大小无关紧要了

Researcher 8:32:47
I see. If the difficulty is too high, will you have other psychological feelings besides being bored?
原来如此。难度太高的情况下，您除了无聊还会有其它的心理感受吗？

Informant 007 8:33:02
For example, when I arrive to a place, I take something and gets the stage cleared. Then I move to another place and get the stage cleared using the same method, and so on. I would feel bored.
比如我到了这个地方 拿了东西就通关 到了另外一个地方 以相同的方式拿了东西 又通关 以此类推 就会感觉到枯燥

Informant 007 8:33:58
Things will definitely go against it, some will make me give up, and some will increase my interest.
物极必反吧，一些会令我放弃，而又一些反而会提高我的兴趣

Researcher 8:34:04
I can understand this process as the in-game challenge is unchanged, your game level is constantly improving, it is possible to enter the loop?
我能把这个过程理解成游戏内挑战不变的情况下，您的游戏水平不断提高，就有可能进入循环？

Informant 007 8:34:33
Yes yes

是的 是的

Informant 007 8:34:45
Too boring.
太枯燥了

Researcher 8:34:53
I see.
原来如此。

Informant 007 8:34:56
The loop is too obvious.
套路太明显了

Informant 007 8:34:59
and makes it not interesting.
就没意思了

Researcher 8:35:23
So you think that the boring feelings in the game has its levels, right?
所以您认为游戏中感受到的无聊是有级别的，对吗？

Informant 007 8:35:58
Yes.
嗯

Researcher 8:36:25
The level of boring leads to your interest or abandoning the game directly, right?
无聊程度高低导致了您有兴趣或者直接放弃游戏，是吗？

Informant 007 8:36:33
Yes.
是的

Researcher 8:37:36
Generally, which levels of boring can lead to an increase in your interest or make you give up the game directly?
一般无聊程度在什么样的时候，能分别导致提高您的兴趣或者让您直接放弃游戏呢？

Informant 007 8:38:21

This is a bit confusing.

这个有点说不清 道不明诶

Informant 007 8:38:39
I feel that it should be similar to my own interests.
感觉还是应该和自身的兴趣比较相近吧

Researcher 8:39:51
Ok. Let's change a topic.
好的。我们换一个话题。

Informant 007 8:39:51
If you let me play some simulation business type (of games). Maybe I would fall asleep while playing. 🤭

让我去玩一些模拟经营类的 可能我都会玩着玩着睡着🤭

Researcher 8:40:20
I see. Ha ha.
原来如此，哈哈

Researcher 8:40:21
What would you do if you have had a boring experience in the game? Would you buy in-game good to alleviate the boredom?
如果您在游戏中有无聊的经历，您会怎么做?您会购买游戏内的商品来缓解无聊吗？

Informant 007 8:41:07
Unless buying the in-game goods can "stimulates" me.
除非购买游戏内的商品可以来“刺激”我

Researcher 8:41:43
Ok. Under this circumstance, which types of in-game goods would you purchase (Power-ups, Expansion packages, Cosmetics/Skins, Loot boxes, Time-savers)?
好的。在这种情况下，您会购买哪些类型的游戏内商品呢？（增强道具，扩展包，可游玩的角色，装饰/皮肤，抽奖箱，省时道具）？

Researcher 8:42:02
Which type of goods is more likely to stimulate you when having the boring experience?
哪种类型的商品更容易在无聊的体验下刺激你？

Informant 007 8:43:31
I thought about it. In fact, no matter what kind of game, it will be boring for a while. It' s just more or less. I don' t know whether it' s a kind of high or low mental activity.

If the purchase of an in-game good can stimulate myself, I will still buy it. For example, Power-ups, decoration?
我想了一下 其实无论什么游戏都会有那么一段时间会觉得无聊 只不过或多或少而已 不知道算不算心理活动的一种高低 高情绪 低情绪
购买内购物品 如果可以刺激一下自己 还是会去购买的 比如增强道具 装饰？、

Informant 007 8:43:48
Cosmetics/Skins, Time-savers
装饰/皮肤 省时道具

Researcher 8:44:53
Ok. I understand. Do you think the concept of "stimulating me" is related to "challenges in the game"?
嗯。我明白了。您觉得"刺激我"这个概念和"游戏内的挑战"有关系吗？

Informant 007 8:45:19
There are some relationships.
会有那么一些关系吧

Informant 007 8:45:54
If when I feel bored, the challenges in the game are actually irrelevant.
如果当我觉得无聊的时候，游戏内的挑战其实已经无关紧要了

Researcher 8:46:15
Why?
为什么呢？

Informant 007 8:47:24
If it makes me feeling bored, certainly the game itself has made me dissatisfied, or it has not reached my expectations.
会让我觉得无聊 肯定因为自身或游戏本身另我感觉不满 或者说没有到达我的预期。

Researcher 8:47:48
I understand. Let's move on to the next topic.
我懂了。我们继续下一个话题。

Researcher 8:47:52
Have you had an anxious experience during the game?
您在游戏时有过焦虑的经历吗？

Informant 007 8:48:17

I have! Very anxious! Broken heart!

有！非常焦虑！操碎了心！

Researcher 8:48:23
Under what circumstances does it usually occur?
通常在什么情况下有？

Informant 007 8:48:38
When playing DOTA2...It's really hard to say.
打 DOTA2 呀 真的是一言难尽啊

Researcher 8:49:08
When you feel anxious, generally what happens?
您觉得焦虑的时候，一般发生在什么情况下？

Researcher 8:49:22
Can you describe the situation at the time?
可不可以描述一下当时的情况？

Informant 007 8:49:47
It usually happens in the moba type games. When I can't communicate well with my teammates, I will feel very anxious.
一般发生在这种 moba 类游戏中，与队友交流不通，会觉得很焦虑。

Researcher 8:50:34
Why the obstacle of communication leads to your anxiety?
为什么沟通有障碍会导致您的焦虑呢？

Informant 007 8:51:23
This type of game is based on communication. If the teammates don't listen to suggestions and advices, they will bury the round.
这类游戏就是靠沟通 配合的 有队友不听建议和劝告 会葬送掉这一场比赛

Informant 007 8:52:22
Although it is said that the win or lose in the virtual world consists of a bunch of data, but the Eager Cui still makes you feel anxious and unhappy.
说是说虚拟世界的输赢指示一堆数据 但是好胜心的崔氏 还是会让你感到一些焦虑与不快

Researcher 8:52:36

I would like to know whether the performance of the teammates is related to the game difficulty you perceive.
我想知道队友的表现和您感知的游戏难度有关系吗？

Informant 007 8:53:38
There will be some.
会有一些把

Researcher 8:55:15
Have you had any experience of supportive purchasing? I mean purchasing in-game goods for supporting the game marker than acquiring the in-game goods themselves.
原来如此。您有没有过支持性购买的经历？我的意思是为了支持游戏开发商而购买游戏内商品，而不是为了获得游戏内商品本身。

Informant 007 8:55:52
There is only one so far.
至今为止只有一款

Researcher 8:56:16
Do you think supportive purchasing is related to flow experience?
好的，您认为支持性购买和心流体验有关吗？

Informant 007 8:56:41
There is no relationship.
没有关系

Researcher 8:57:08
Ok. What do you think led to your supportive purchasing?
好的。您认为什么导致了您的支持性购买？

Informant 007 8:58:14
Nostalgia? World of Warcraft is such a game.
情怀吧？魔兽世界就是这样一款游戏

Informant 007 8:58:46
The content of the game is actually familiar to the heart, but when the nostalgic service was open, I recharged as usual and silently lining up. 😂
游戏内容其实已经烂熟于心 但是怀旧服一开 还是一如既往的充值 然后默默排队😂

Researcher 8:59:24

Me too.

我也是。

Informant 007 8:59:37
True fragrance series.
真香系列

Researcher 8:59:44
The last question: Is acquiring the flow experience your purpose of playing videogames?
最后一个问题：获得心流体验是否是您玩电子游戏的目的？

Informant 007 9:00:29
Yes.
是的

Informant 007 9:00:45
I play videogames to be isolated from the outside world, and to temporarily enter my own game world.
玩电子游戏 就是想与外界隔绝 暂时进入自己的游戏世界

Researcher 9:01:07
Ok. The interview is almost over. Do you have any viewpoints to add?
好的。访谈差不多要结束了。您还有什么观点需要补充吗？

Informant 007 9:01:54
It seems that there is no point to add. In turn, if you have any more questions, you can continue to ask.
好像没有什么观点要补充 不过倒是你这边 如果还有什么问题 也可以继续问

Informant 007 9:02:03
If you let me to add, I definitely cannot think more at this moment.
让我想补充 肯定一时半会想不出来了

Researcher 9:02:31
So, I am sorry to delay you for a while. Because I still want to ask a little more about some problems.

那不好意思耽误你一会儿时间。因为对刚才有一些问题我还想问得稍微细一些

Informant 007 9:02:49
Yes, no problem. Please.
可以的 没问题 你说

Researcher 9:03:00
When I asked “I would like to know whether the performance of the teammates is related to the game difficulty you perceive.”, you said “There will be some.”.
我刚才问道“我想知道队友的表现和您感知的游戏难度有关系吗？”您说“会有一些把”

Researcher 9:03:06
Specifically, what sort of relationship is it?
具体是什么样的关系呢？

Informant 007 9:05:42
The plan is that I use the role A. When attacking the roles C, D, and E of the enemy camp, I need my teammate role B. When I raid the enemy, he/she raise a high wall behind them to block the back road. However, he/she got a high wall in front of me, and I couldn' t beat others, and they ran away. Ended with failure.
计划是我使用的角色 A，在突袭敌方阵营的角色 C,D,E 时，我需要己方角色 B，在我突袭敌方的同时在他们身后竖起高墙 阻断后路。 结果他在我面前起了一到高墙，我打不到别人 别人也逃跑了 失败告终

Researcher 9:06:24
That is to say, the poor performance of teammates leads to your high perceived difficulty of the game, right?

也就是说队友表现差导致了感知到的游戏难度提高，对吗？

Informant 007 9:06:35

Maybe it is this kind of situation, in which the teammates don't perform well. Or maybe it also can be a problem of the game difficulty itself.
可能类似的这种情况 会让我觉得队友的表现不佳 也可能会是游戏难度的问题

Researcher 9:07:06
I see. On the other hand, In the case of feeling anxious, will you buy in-game goods to alleviate anxiety?
原来如此。另外。在感觉焦虑的情况下，您会购买游戏内的商品来缓解焦虑吗？

Informant 007 9:08:12
There are many reasons, so I would say that there are some relationships. Maybe because the release of the "high wall" is too precise, may be the distance of the casting of the enemy is too far, or maybe it may also be a handicap of hands. Hahaha
多方面的原因　所以我回答会存在一些关系。　可能是因为“高墙”的释放精准度过高　己方的施法距离过远　也可能是手残 哈哈哈

Informant 007 9:08:17
Multiple reasons.
多种原因

Researcher 9:08:42
Which types of in-game goods would you purchase in this case (Power-ups, Expansion packages, Playable characters, Cosmetics/Skins, Loot boxes, Time-savers)?
在这种情况下，您会购买哪些类型的游戏内商品呢？（增强道具，扩展包，可游玩的角色，装饰/皮肤，抽奖箱，省时道具）？

Informant 007 9:09:50
Maybe Power-ups Maybe Playable characters.
可能会买增强道具 也可能会增加角色选择吧

Researcher 9:11:47
It turned out to be the case. We just said that buying in-game goods to alleviate anxiety or boredom, then I would like to ask the final purpose is to re-enter the state of flow experience?
原来如此。我们刚才说到购买游戏内的商品来缓解焦虑或者无聊，那么我想请问最终目的是为了重新进入心流体验的状态吗？

Informant 007 9:13:35
Yes.
是的

Researcher 9:13:47
OK~~~I have done!
好的~~~我问完啦！

Informant 007 9:14:19

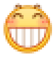

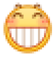

Informant 007 9:14:30
Ready to organise the documents?
开始整理文案了咯？

Researcher 9:14:33
These are all the questions. Thank you very much for participating in our research. Please confirm that your email address is XXXXXX@XXXXXX.com, because later we will send the JD electronic gift card to this address.
这就是全部的问题。 非常感谢您参与我们的研究。请确认您的电子邮件地址是XXXXXX@XXXXXX.com，因为稍后我们把京东电子礼品卡发送到这个地址。
