# Supplementary material for: A grounded theory approach to understanding in-game goods purchase
Source: PLoS One. 2022 Jan 27;17(1):e0262998. doi: 10.1371/journal.pone.0262998 (PMC8794092; doi:10.1371/journal.pone.0262998)
Supplement: S1 File — (ZIP) [file pone.0262998.s001.zip › Transcript 12.pdf]

Interview: 012

Informant: 010

Please note that the original transcript is in Simplified Chinese. The English translation is for internal communication among the author of this research, and it is not proofread. Potential linguistic errors may exist in the English translation.

Researcher 14:57:43
Thank you for your willingness to participate and be interviewed here. My name is XXX XXX, and I'm a PhD student in the XXX University of XXX(XXX). Currently, I'm working on a research project which focuses on videogame players' purchase motivations of in-game goods. Throughout this interview, I will ask you a series of questions and you are encouraged to express your opinions freely with emoticons. If I have questions about what you've said or need clarification about a topic or concept, I'll ask you.
感谢您愿意参加并在此接受采访。 我叫 XXX，我是市场营销学的博士生，现在我在 XXX 大学就读。 目前，我正在开展一个研究项目，专注于电子游戏玩家对游戏内购买项目的购买动机。 在整个访谈中，我会问您一系列问题，我们鼓励您自由表达您的意见和观点。因为这不是一个当面访谈，所以我们也鼓励您用 QQ 表情来表达您的情绪。 在访谈过程中，如果我对你所说的内容有疑问或需要您澄清一个主题或概念，我会问您。

Researcher 14:57:48
Are you ready?
您准备好了吗？

Informant 010 14:58:02
Ok。
OK

Researcher 14:58:03
"Flow experience" has been used by psychologist to describe a state of mind experienced by people who are deeply involved in an activity. Instance, sometimes while playing videogames, the player's action and awareness are merged, and he/she is totally connected on the gaming tasks at hand. In this state, the player loses his/her consciousness, and his/her perception of time becomes faster or slower than usual. Also, the player perceives a feeling of being in control, which empowers him/her from the fear of failure.
心理学家使用"心流体验"来描述深度参与某项活动的人所经历的心理状态。 例如，有时玩家在玩电子游戏时，他/她的动作和意识会融为一体，并且他/她完全关注手头的游戏任务。 在这种状态下，玩家失去他/她的自我意识，他/她对时间的感知变得比平时更快或更慢。 此外，玩家会感受到一种掌控全局的感觉，这使他/她免于对失败的恐惧。

Researcher 14:58:05
Think about your own gaming experience for a moment. Have you ever experienced flow while playing videogames?
请回想一下您自己的游戏经验。 您玩电子游戏时有没有经历过心流体验？

Informant 010 14:59:04
Yes. When I am playing the videogame, I actually don't know what's happening in the outside world.
是的，我竟然玩游戏玩的不知道外界发生了什么

Informant 010 14:59:17
Sometimes I would unconsciously answer my friends' words.
有时候会无意识回答朋友的话

Informant 010 14:59:30
But I forget all while playing.
但是玩好游戏忘记了

Informant 010 14:59:33
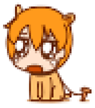

Researcher 15:00:02
I see. What else behavioural characteristics would be?
原来如此，请问还有什么行为方面的特征吗？

Informant 010 15:00:18
When playing smoothly, the time goes very fast. When the gaming is not smooth, time goes very slow.
在玩的顺的时候时间非常快的，玩的不顺的时候，时间感觉很慢

Researcher 15:01:19
I remember the last time we talked about this topic.
我记得上一次我们也有谈到这个话题。

Informant 010 15:01:39
Yes.
是的

Informant 010 15:01:43
(We also) talked about the time.
也有说到过时间

Researcher 15:01:57
You mentioned "Otherwise, some (people) cannot go on and stop in the middle way.","When being stuck in a stage for too long time, I would like to abandon the play.","I can be very annoyed when I cannot continue, not happy."
您说到 "不然有的打了一半就玩不下去了", "有时候关卡卡太久，就会不想玩了", "打不下去的时候就会很烦躁，不开心"

Informant 010 15:02:12
It's very easy to put aside the things in real life when playing games.
玩游戏的时候非常容易抛开现实中的一些事情

Informant 010 15:02:34
Yes.
是的

Researcher 15:02:40
Can I understand the psychological activities I just mentioned as that you are very anxious?
我能把刚才说的那些心理活动理解成您很焦虑吗?

Informant 010 15:02:56
Yes, you can.
可以的

Informant 010 15:03:04
It is very uncomfortable.
就是会很不爽啊

Researcher 15:03:08
What would you do if you had an anxious experience in a game?
如果您在游戏中有焦虑的经历，您会怎么做?

Informant 010 15:03:21
Generally, there are three situations.
一般三种情况

Informant 010 15:03:33
The first (situation) is giving up for a while and wait to see what’s going on.
第一种放弃，过一会儿再说

Researcher 15:03:34
Ok. I’m listening.
嗯嗯，洗耳恭听

Informant 010 15:03:40
The second (situation) is seeking for the help from friends..
第二种求助朋友吧

Informant 010 15:03:46
The third (situation) is recharging money
第三种就是充钱

Informant 010 15:04:07
Generally there are items that can directly unlock the access to the next stage or give the tips.
一般都会有道具可以直接下一关，或者给提示

Researcher 15:04:28
I see. In the first case you mentioned, here "giving up" refers to the temporary abandonment, and you will return to the game after that, right?
原来如此。您说的第一种情况，这边的放弃指的是暂时放弃，之后还会回到游戏的意思吗？

Informant 010 15:04:34
Yes.
是的

Informant 010 15:04:42
(To) adjust the mindset.
调整一下心态

Informant 010 15:04:47
(To) go to do something else.
先去做一些别的事情

Researcher 15:04:55
有没有想要永久放弃的情况？
Informant 010 15:05:10
Are there circumstances which lead you want to give up permanently?
有

Researcher 15:05:58
I would like to ask whether there are differences between the anxiety that you can alleviate through the mentioned three methods and the anxiety of wanting to give up the game permanently?
我想请问想要通过上述三种途径去环节焦虑的情况和想要永久放弃游戏的焦虑的情况会有不一样的地方吗？

Informant 010 15:06:05
Previously, I found that in some games, I couldn't research the state that I wanted, no matter how much money I had recharged. Similarly, I didn't want to continue playing if my level was far away from my friends.
之前有的游戏就是你发现自己不管冲多少钱也不达不到自己要的效果，或者实力和一起的小伙伴相差很多的时候就会不想玩了

Researcher 15:06:07
*Alleviate
*缓解

Informant 010 15:06:42
There are differences.
会有区别

Researcher 15:07:25
What differences do you think between the two different anxieties that lead to different outcomes?
您认为这两种导致不同结果的焦虑感有哪方面有区别?

Informant 010 15:08:01
The temporarily one is temporarily unhappy, and it's fine after the problem being solved.
一种只是暂时的不开心，解决了问题之后可能就好了

Informant 010 15:08:30
The permanent one is that the game cannot give me the experience that I want.
永久的话，就是这个游戏给不了我想要的那种体验了

Researcher 15:09:04
Can I interpret the anxiety has its levels? The anxiety which leads you wanting to give up is stronger?
我能不能理解成焦虑的程度有高低？想要放弃游戏的那种焦虑感更强一些？

Informant 010 15:09:13
Yes.
是的

Informant 010 15:09:29
Some are just minor problems which can be solved easily.
有些只是小问题，很容易解决

Informant 010 15:09:48
Some may take a relatively large effort.
有些可能要花费比较大的精力，

Informant 010 15:09:53
and I want to give up.
就会想要放弃了

Informant 010 15:10:11
The efforts and the rewards are not proportional
付出和得到不成正比

Informant 010 15:10:16
Or there is a big difference.
或者偏差太大

Researcher 15:11:18
Ok. I understand this point. Let’s talk about the second situation: ask for the help from other players. We also mentioned it the last time: “Some powerful (persons) in the game league.”, “Sometimes he/she can lead five (persons)”,” If it’s a group (mission), then the Big God leads us.”.
原来如此，我明白这一点了。我们来讨论一下第二种情况。就是请其它的玩家带。我们上次也说到了这一点：“游戏联盟里的特别厉害的”，“有时候他可以一拖五”，“团队的就是大神带”

Informant 010 15:11:34
Yes.
嗯

Informant 010 15:12:09
The game I play now has a lot of in the league competitions.
我现在玩的游戏就是联盟赛比较多

Informant 010 15:12:14
The thighs are required.
就会很需要大腿

Informant 010 15:12:18
(hahahaha)

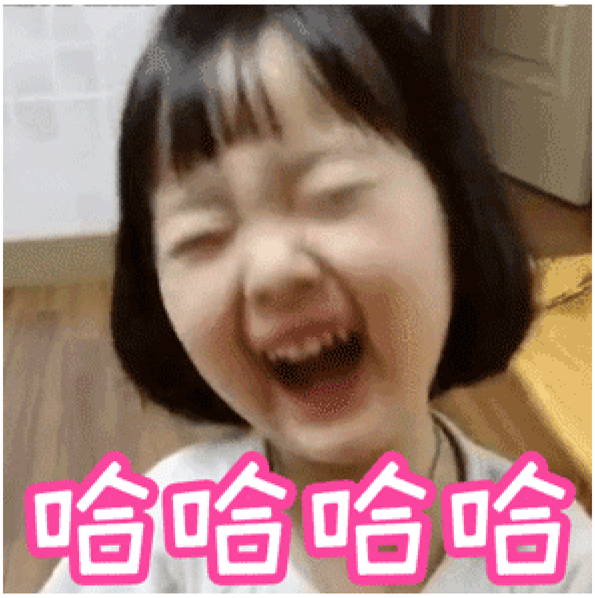

Researcher 15:12:28
In the case of seeking for the help from other players, do you think the game difficulty is lower than playing solely?
在邀请其他玩家帮助的情况下，您是否觉得游戏的难度比一个人玩要低一些？

Informant 010 15:12:37
Yes
是的

Informant 010 15:12:49
Because Dungeons allow 1-5 people to enter.
因为副本他可以是 1-5 人进去

Informant 010 15:12:55
It's very slow when playing solo.
你一个人打就会很慢

Informant 010 15:13:06
When there are a lot of people, it goes faster.
人多的时候就非常的快

Informant 010 15:13:26
Of course, it is also needed to consider whether there are more Big Gods or more rookies in the team.
当然人多也要看队伍里面是大神多还是菜鸟多

Researcher 15:14:09

Ok. After seeking the help from Big God players, will it help you to enter the flow state as we have mentioned?
恩恩。请求大神玩家帮忙之后，会帮助您进入我们刚才所说的心流体验的状态吗？

Informant 010 15:14:10
The rookies can't help, and the mood will be very uncomfortable when they drag your legs. (If the situation) keeps going on (like this), I would have the intention to give up.
菜鸟多帮不上忙,还拖后腿的时候心情也会很不爽，时间久了也会有汽油的心态

Informant 010 15:14:32
When Big God leads the team, it is just like "lie down and win"
大神带队就是躺着赢

Researcher 15:14:56
I see. If you are teaming up with rookies, will you feel that the difficulty of the game is higher?
原来如此。如果是和菜鸟组队，会感觉游戏难度反而变得更大吗？

Informant 010 15:15:03
Yes.
是的

Informant 010 15:15:13
I used to lead the second team.
我之前就因为一直带二队

Informant 010 15:15:17
The mood was blown up
心情炸了
Informant 010 15:15:23
(I) wanted to give up the game.
想汽油

Informant 010 15:15:31

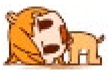

Informant 010 15:15:56
The main team went to participate the competition of the full server.
主力代表区参加全服的比赛了

Informant 010 15:16:20
I leaded the second team at home.
我在家里带二队，

Researcher 15:17:17
Are there more rookies in the second team?
二队的队友比较多菜鸟吗？

Informant 010 15:18:03
Yes.
是的，

Informant 010 15:18:11
And they don’t listen to the command
而且不听指挥

Informant 010 15:18:18
Generally (when conducting) league activities, (we would speak in) yy.
一般联盟活动会 yy

Informant 010 15:18:30
They don't listen at all to what you are talking about in YY.
你在 yy 里怎么说都没人搭理你

Informant 010 15:18:53
You finished the battle plan at the first place, but no one cared about you. (They) didn't do what you arranged.
你早早写完了作战计划，也没人理你。不安安排走

Informant 010 15:19:01
If (we) lose, someone would say that my command was not good.
输了还会有人说你指挥不好

Researcher 15:19:17
I understand. They didn't listen to the command, and the final result is that you felt the game difficulty was very high?
我懂了，他们不听指挥，让后最终导致的结果是您感觉游戏难度很大？

Informant 010 15:19:41
Yes, my mind was very fried.
嗯，心态很炸

Researcher 15:19:47
Ok. Right.
ok，好的。

Researcher 15:20:01
Alright, I understand. Let's talk about the third situation, which is to buy in-game goods by recharging money. Which types of in-game goods would you purchase in this case (Power-ups, Expansion packages, Playable characters, Cosmetics/Skins, Loot boxes, Time-savers)?
好的，我明白了。我们来谈谈第三种情况，就是通过充钱来购买游戏内道具。在这种情况下，您会购买哪些类型的游戏内商品呢？（增强道具，扩展包，可游玩的角色，装饰/皮肤，抽奖箱，省时道具）？

Informant 010 15:20:02
I felt that it was a waste of time.
就会觉得浪费时间

Informant 010 15:20:24
Power-ups
增强道具

Researcher 15:20:59
好的，您认为购买增强道具可以帮助您重新进入心流状态吗？

Informant 010 15:21:21
(They) help partially.
有一部分的帮助

Informant 010 15:22:22
The attributes can be strengthened.
可以加强属性

Researcher 15:22:34
As you said previously. It is the case that no matter how much you have bought, you cannot reach the state you want, right?
也有如您之前所说，就是无论怎么购买，也达不到自己想要的效果的情况，对吧？

Informant 010 15:23:02
Yes.
是的

Informant 010 15:23:06
The boss is too strong.
boss 太厉害

Informant 010 15:23:16
(I) loss even after having purchased (them).
买了还是输啊

Informant 010 15:23:34
I can only go back to practice basic attributes.
就只能回去在连连基本属性了

Researcher 15:24:24
I see.
原来如此。

Researcher 15:25:07
Let's talk about the boring experience in the game. In the last interview, you said “It doesn't make sense to play anymore. There are a lot of games nowadays, and many of them are similar. They let you to buy this or to buy that. You only spend a few minutes playing before they let you to the money for opening the stages.”, “Mr Love: Queen's Choice… It's just like reading a novel book.”, “There is also Star Dream . Every day doing the repetitive things”
我们再来谈谈游戏中无聊的体验。在上次访谈中，您说到"感觉玩下去没意义了。现在很多游戏吧。雷同很多，还动不动就让你买这个买那个，你才玩了几分钟就让花钱开启关卡","很直接的就是你充几块钱买个道具才让你下去","恋于制作人吧...感觉就是在看小说书", "还有一个明星志愿也是。每天都是重复的事情"

Informant 010 15:25:10
Especially time-level cross-level challenge
特别有时间跨级挑战

Informant 010 15:25:26
Yes.
对

Researcher 15:25:33
What would you do if you had a boring experience in a game?
如果您在游戏中有无聊的经历，您会怎么做?

Informant 010 15:25:40
Give up.

放弃

Informant 010 15:25:46
(Please give up)

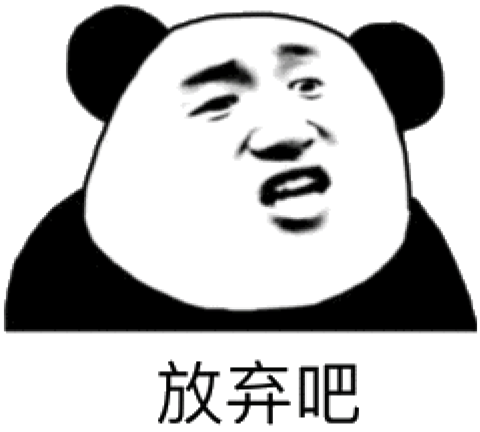

Researcher 15:26:04
Will you buy in-game goods to alleviate boredom?
您会购买游戏内的商品来缓解无聊吗？

Informant 010 15:26:04
It depends on the game types.
要看游戏类型

Informant 010 15:26:16
To see whether it is worth or not.
要看值不值得吧

Informant 010 15:26:43
Like Mr Love: Queen's Choice, I recharged 2-3 times.
像恋与制作人我就是充了 2-3 次钱

Informant 010 15:26:48
I still felt boring.
感觉还是很无聊

Informant 010 15:26:55
so I gave up.
我就不玩了

Informant 010 15:27:01
I did the same to Star Dream.
明星志愿也是

Researcher 15:27:08
Well, have you ever had the experience that previous you were bored, but after recharging you didn't feel bored?
恩，以前有没有过很无聊的情况下，充钱后变得不无聊的情况？

Informant 010 15:27:19
The Luanshiwangzhe I’m playing now.
现在玩的乱世

Informant 010 15:27:32
In the first place I didn’t recharge.
以前一开始不充钱

Informant 010 15:27:39
I felt as if I were playing QQ farm.
感觉就在玩 qq 农场

Informant 010 15:27:53
Currently, (I need to) attention many league activities.
现在是很多联盟活动要参加

Informant 010 15:28:33
In particular, some activities have quotas。
特别是有些活动有名额限制，

Researcher 15:28:48
In other words, when playing Luanshiwangzhe, it is boring during the period of farming merchants?
也就是说，在玩乱世王者的时候，在做种田商人的期间比较无聊？

Informant 010 15:28:55
Yes.
对

Researcher 15:29:16
How do you feel the difficulty of the game at this time?
您这个时候觉得游戏的难度怎么样？

Informant 010 15:29:29
Some of us now feel that there are too many activities, and we have to participate them all. Then they retire.
我们现在有些人是觉得活动太多了，什么都要参加，然后退游了

Informant 010 15:29:47
It's very boring. Go up every day to collect vegetables and fight with the wild (enemies).
就很无聊，每天上去收菜收兵打野

Informant 010 15:29:55
It takes little time.
花不了多少时间

Researcher 15:30:14
Did you feel that you were doing things in a loop?
觉得游戏里一直在做循环往复的事情？

Informant 010 15:32:04
Yes.
对的

Informant 010 15:32:16
and I had to be beaten by combat accounts.
而且还要老被战斗号打

Informant 010 15:32:25
I wanted to fight back but failed.
想还手打不过

Researcher 15:33:07
Yes, this is what you mentioned last time. Some bad experiences when being a farming merchant: “Farming merchants' gaming method is being beaten...being robbed…”
对，这是您上次也提到过的。当种田商人时候的一些不好的体验："种田商人的玩的方式就是挨打…被打劫…"

Researcher 15:33:22
Was your inner feeling at that moment anxious?
这个时候内心的感受是不是焦虑的？

Informant 010 15:33:24
Yes.
是的

Informant 010 15:33:31
Super super bad mood.
超级超级不爽

Informant 010 15:33:50
So I fought back by recharging money.
所以就充钱打回来啊

Informant 010 15:33:59
Moreover, when you called him/her not to beat you,
而且你去让他别打你

Informant 010 15:34:05
he/she would ridicule you.
他还会讽刺你

Researcher 15:34:43
When you were a farmer, did you always feel bored or anxious?
请问您在当种田商人期间，心里总是要么是很无聊，要么是很焦虑的状态？

Informant 010 15:35:15
I wouldn’t go online several times a day.
一天都不会上几次游戏

Informant 010 15:35:49
It feels like playing QQ farm.
感觉就是玩 qq 农场

Informant 010 15:35:56
Harvest the vegetables when reaching the time.
到点上去收菜

Informant 010 15:36:09
(I was) anxious when being beaten.
被打就焦虑

Researcher 15:36:32
Did you have the flow feeling when you were a farming merchant? The state of entering to the flow experience?
请问您在当种田商人的时候有没有过那种玩得很爽的感觉？就是进入心流体验的状态？

Informant 010 15:37:06
No.
没有

Informant 010 15:37:35
So I have always been very curious
所以我一直很好奇

Informant 010 15:37:50
How could I insist on playing at the period of farming.
还在种田的怎么玩的下来的

Informant 010 15:38:02
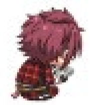

Researcher 15:38:09
Subsequently, what was the opportunity that led to flowing gaming?
后来是什么契机导致了您玩得很爽？

Informant 010 15:38:19
(I) changed the league.
换了联盟

Informant 010 15:38:24
(I) knew the new friends.
认识了新的伙伴

Informant 010 15:38:46
I always remember that when I just joined the new league, I couldn't play (well).
我一直记得我刚刚到新联盟，不会玩

Informant 010 15:38:52
(I was) being beaten every day.
天天挨打。

Informant 010 15:39:31
Some friends of the league taught me a lot of things, and they protected me every day.
盟里几个小伙伴就教我很多东西。还每天保护我

Informant 010 15:39:46
They would beat whoever had beaten me.
谁打我，他们就去打谁

Informant 010 15:40:02

Over time, I felt that I couldn't drag my legs for a long time.
时间久了感觉不能拖后腿

Informant 010 15:40:15
Later I (began to) recharge to buy military commanders and so on.
后面就充钱买武将买什么什么

Researcher 15:40:39
I would like to ask in that period, was the game difficulty you perceived different to the period of being farming merchant?
请问在这个时期您感知到的游戏难度和种田商人时期有什么变化吗？

Informant 010 15:40:50
(I) could go together with them to participate league competitions.
就可以和他们一起去打联盟赛

Informant 010 15:41:13
After being combatant, I began to get busier.
战斗号之后就忙了很多

Informant 010 15:41:29
(I) had many gaming tasks and competitions.
有很多很多的游戏任务和比赛

Informant 010 15:41:50
It was not just playing the game solely.
就不单单的只是一个人玩游戏了

Researcher 15:42:17
Ok. Did you feel that the challenges in the game had been increased?
恩恩。感觉游戏内的挑战变多了？

Informant 010 15:42:22
Instead, a group of people played the game together, and sometimes you were essential in the team.
而是一群人一起玩游戏，有时候你会成为团队里必须存在的一名

Informant 010 15:42:32
Yes.
是啊

Informant 010 15:42:36
Regarding to the farming,

种田的话

Informant 010 15:42:40
(I) couldn’t participate many activities.
很多活动参加不了

Researcher 15:43:23
Ok.
原来如此。

Researcher 15:44:11
I have a question: For you, with the increasing of the challenges in the game, do you think the game difficulty is getting higher?
我有一个问题: 对您来说，游戏内的挑战变得更多了，以为着游戏的难度也更大吗？

Researcher 15:44:19
*意味

Informant 010 15:44:51
Yes.
是的

Informant 010 15:45:02
More time and effects are required.
需要花费更多的时间精力

Informant 010 15:45:07
Including money.
包括金钱

Researcher 15:45:36
I see. At the period of being farming merchant, when you were bullied by others, did you think the game was difficult?
原来如此。那在当种田商人被别人欺负的那一刻，您觉得游戏的难度大吗？

Informant 010 15:46:13
I didn’t think the game was difficult.
也不会觉得游戏有啥难度

Informant 010 15:46:20
Just hate the person who beat me.
就是讨厌打自己的人

Researcher 15:47:39
Ok. Do you remember the type of in-game goods that you bought when you first decided to recharge the money? (Power-ups, Expansion packages, Playable characters, Cosmetics/Skins, Loot boxes, Time-savers)
好的。您还记得第一次决定充钱不当种田商人时买的是哪种类型的游戏内商品吗？（增强道具，扩展包，可游玩的角色，装饰/皮肤，抽奖箱，省时道具）

Informant 010 15:48:02
I purchased a military commander.
我买了个武将

Informant 010 15:48:08
Playable character.
角色

Informant 010 15:48:25
(I bought) a monthly card.
买了个月卡

Informant 010 15:48:30
and bought a military commander.
然后买了个武将

Researcher 15:48:34
What are the differences between this role and the role of farming merchant?
这个角色和您种田商人时候的角色有什么不一样的地方?

Informant 010 15:48:50
When being farming merchant, what the system gave was developmental commander.
种田时候系统给的武将都是发展武将

Informant 010 15:49:03
When fighting, you have to spend money to buy military commanders.
战斗时候就要花钱买战斗武将

Informant 010 15:49:08
The commanders given by the system are very weak.
系统给的武将都很菜

Informant 010 15:50:01
The commanders of this game are generally expensive.
这游戏的武将都蛮贵的

Informant 010 15:50:05
(I refer to) the useful commanders.
有用的武将

Researcher 15:50:59
Do you think that after purchasing the military commander, did it help you re-enter the flow state?
您认为购买了那个战斗武将后，帮助了您重新进入心流状态吗？

Informant 010 15:51:15

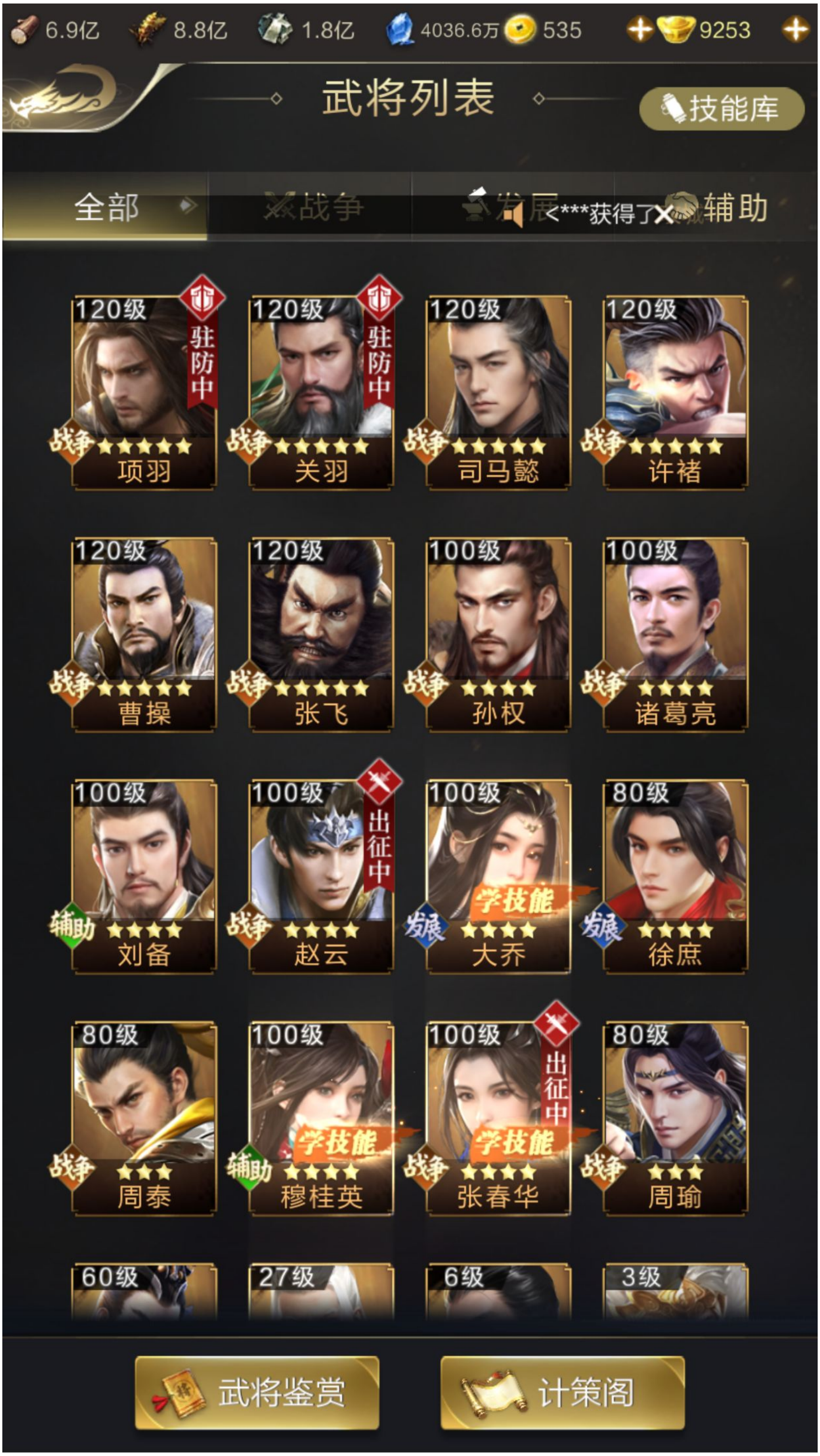
6.9亿
8.8亿
1.8亿
4036.6万
535
9253
武将列表
技能库
全部
战争
发展
<***获得了
辅助
120级 驻防中 战争 项羽
120级 驻防中 战争 关羽
120级 战争 司马懿
120级 战争 许褚
120级 战争 曹操
120级 战争 张飞
100级 战争 孙权
100级 战争 诸葛亮
100级 辅助 刘备
100级 出征中 战争 赵云
100级 学技能 发展 大乔
80级 发展 徐庶
80级 战争 周泰
100级 学技能 辅助 穆桂英
100级 出征中 学技能 战争 张春华
80级 战争 周瑜
60级
27级
6级
3级
武将鉴赏
计策阁

Informant 010 15:51:17
Yes.
有

Informant 010 15:51:25
These (commanders) in orange need to be purchased by money.
这些橙色都是要花钱买的

Informant 010 15:51:30
The most expensive costs 3000.
最贵的 3000

Informant 010 15:51:38
The most cheap costs 100.
便宜的 100 多吧

Researcher 15:51:50
Are we talking about RMB?
我们说的是人民币吗？

Informant 010 15:51:54
Yes.
对

Informant 010 15:52:10
Xiangxu costs 3200.
那个项羽是 3200

Informant 010 15:52:20
(After purchasing it), (it is rated as) one star.
买来一星

Informant 010 15:52:24
Then upgrading is required.
然后再升级

Researcher 15:52:35
Ok. I see.
好的，我明白了。

Researcher 15:52:49
Once you have a flow experience, would you want to go back to the game in order to regain this experience?
一旦获得过心流体验，您会想回到游戏中为了重新获得这种体验吗？

Informant 010 15:53:00
Yes.
会

Informant 010 15:53:09
(I would repeat) the dungeons and cannot stop.
副本什么的都会刷不停

Informant 010 15:53:18
The time goes very fast when playing in the dungeon.
刷副本时间过的很快

Researcher 15:53:39
I see. Is acquiring the flow expensive your purpose of playing videogames?
原来如此。获得心流体验是否是您玩电子游戏的目的？

Informant 010 15:54:08
There are relationships
有关系

Informant 010 15:54:14
After all, it is very tired to go to work.
毕竟上班很累

Informant 010 15:54:24
(I) play videogames to adjust the mindset.
玩游戏调整心态

Informant 010 15:54:33
Something in the real life can be put aside.
可以抛开现实的一些事情

Informant 010 15:54:56
I don't have to think about some unhappy things in real life.
不用去想现实的一些不开心的事情

Informant 010 15:55:03
After completely get involved in game,
完全投入游戏之后

Informant 010 15:55:31
These things can be forgotten temporarily.
这些东西都是可以暂时忘记

Researcher 15:55:38
Ok. I see.
原来如此，我明白了。

Researcher 15:55:50
Have you had any experience of supportive purchasing? I mean purchasing in-game goods for supporting the game marker than acquiring the in-game goods themselves.
我们换一个话题。您有没有过支持性购买的经历？我的意思是为了支持游戏开发商而购买游戏内商品，而不是为了获得游戏内商品本身。

Informant 010 15:56:14
Nope.
这个没有

Informant 010 15:56:52
Emmm, there is one case: a stand-alone game that I played when I was a child. When the online version was released, I went to play it.
emmm，我只有小时候玩的单机游戏。除了网游版本去玩

Informant 010 15:57:00
And I recharged some money.
然后冲了点钱

Informant 010 15:57:09
Does this count?
这个算么

Researcher 15:57:22
What is the purpose of recharging money in this case?
请问这个时候充钱的目的是？

Informant 010 15:57:46
Like the Star Dream which I mentioned before.
就是我之前说的明星志愿

Informant 010 15:57:54
If you don't pay, they won't let you continue playing.
你不充钱他不让你继续玩下去啊

Informant 010 15:58:21
To pursue the old feeling of playing the stand-alone version.
为了追寻以前玩单机版本的感受

Informant 010 15:58:27

Finally, I found that there is no connection.
最后发现完全没关系

Researcher 15:58:30
There are two different versions of Star Dream, right? One is a stand-alone version that you previously played, and the other is a mobile version, isn't it?
这个明星志愿有两个不同的版本是吗？一个是以前玩的单机版，一个是手游版？

Informant 010 15:58:38
Yes.
对

Informant 010 15:58:48
There titles were published before.
他以前单机一共出了三部

Informant 010 15:58:52
I bought them all.
我都有买

Researcher 15:59:01
I see. Have you ever had the flow experience when playing the stand-alone version?
原来如此。以前在玩单机版的时候，有过心流体验吗？

Informant 010 15:59:06
Yes.
有

Informant 010 15:59:12
I could play (it) all night.
可以玩通宵

Informant 010 15:59:17
And I didn't feel tried.
都不会觉得累

Informant 010 15:59:26
It has many different brunches.
他会有很多不同的支线

Informant 010 15:59:32
Which lead to different endings.
会出现不同的结局

Informant 010 15:59:49
Moreover, there are albums.
然后会有相册

Informant 010 16:00:03
Which include different endings.
把不同的结局收入其中

Informant 010 16:00:12
When the collection was complete, I was full of satisfaction.
收集满的时候很有满足感

Informant 010 16:00:27
I was playing continually for acquiring the desired endings.
为了想要的结局会不停的刷

Researcher 16:00:38
Ok. If there is a game like this that gives you the flow experience, would you make a support purchase if you have the option of in-game goods? No matter what you buy inside.
好的。如果现在还有一个这样子让您获得心流体验的游戏，如果有内购的选项，您会进行支持性购买吗？无论是买什么内购。

Informant 010 16:00:53
Yes.
会

Researcher 16:01:19
Alright, I understand. We return to the concept of permanently giving up the game as just mentioned. For you, what kind of situation refers to the "Permanent abandonment"?
好的，我明白了。我们回到刚才所说的永久放弃游戏这个概念上。对您来说，什么样的情况算是永久放弃游戏了？

Informant 010 16:02:16
Generally, a game which,
一般一个游戏

Informant 010 16:02:21
I have not played more than three days,
我超过三天没有上线

Informant 010 16:02:26
I will not play anymore.
我就不会玩了

Researcher 16:02:42
What is your attitude towards this game at this time?
这时候心里对这款游戏是什么态度？

Informant 010 16:02:52
Tired.
厌倦了

Informant 010 16:03:13
Or I feel that there is no need to continue to pay money for repeating the same thing.
或者是觉得没必要继续晒钱重复同一件事

Researcher 16:03:24
What about the temporary abandonment of the game? Under what circumstances is the temporary abandonment of the game?
那暂时性放弃游戏的情况呢？什么样的情况下算是暂时性放弃游戏？

Informant 010 16:03:55
Just get stuck in one stage.
只是单一关卡过不去

Informant 010 16:04:14
Which can be solved.
能够解决的

Informant 010 16:04:21
In fact, it is mainly a problem of the mindset.
其实主要也是一个心态问题

Researcher 16:04:24
What is your attitude towards this game at this moment?
那这时候心里对这款游戏是什么态度？

Informant 010 16:04:40
What a fool planning.
什么傻子策划

Informant 010 16:04:44
What’s going on with this planning.
策划的什么鬼

Researcher 16:05:03
But you still care about it?
但是依然会有牵挂吗？

Informant 010 16:05:07
Yes.
对

Informant 010 16:05:18
But I still find a way to clear (the stage).
还是会想办法去过

Informant 010 16:05:34
However, when this sort of temporarily abandonment is accumulated,
但是这种短暂的积累久了

Informant 010 16:05:43
It may lead to permanent abandonment.
就会想永久不玩了

Researcher 16:05:50
Ok, I see.
好的，我明白了。

Researcher 16:05:54
The interview is almost over. Do you have any ideas to add?
访谈差不多要结束了。您还有什么观点需要补充吗？

Informant 010 16:06:34
No.
没了

Researcher 16:06:56
These are all the questions. Thank you very much for participating in our research. Please confirm that your email address is XXXXXX@XXXXXX.com, because later we will send the JD electronic gift card to this address.
这就是全部的问题。 非常感谢您参与我们的研究。请确认您的电子邮件地址是XXXXXX@XXXXXX.com，因为稍后我们把京东电子礼品卡发送到这个地址。
