# Supplementary material for: A grounded theory approach to understanding in-game goods purchase
Source: PLoS One. 2022 Jan 27;17(1):e0262998. doi: 10.1371/journal.pone.0262998 (PMC8794092; doi:10.1371/journal.pone.0262998)
Supplement: S1 File — (ZIP) [file pone.0262998.s001.zip › Transcript 13.pdf]

Interview: 013

Informant: 002

Please note that the original transcript is in Simplified Chinese. The English translation is for internal communication among the author of this research, and it is not proofread. Potential linguistic errors may exist in the English translation.

Researcher 13:45:13
Thank you for your willingness to participate and be interviewed here. My name is XXX XXX, and I'm a PhD student in the XXX University of XXX(XXX). Currently, I'm working on a research project which focuses on videogame players' purchase motivations of in-game goods. Throughout this interview, I will ask you a series of questions and you are encouraged to express your opinions freely with emoticons. If I have questions about what you've said or need clarification about a topic or concept, I'll ask you.
感谢您愿意参加并在此接受采访。 我叫 XXX，我是市场营销学的博士生，现在我在 XXX 大学就读。 目前，我正在开展一个研究项目，专注于电子游戏玩家对游戏内购买项目的购买动机。 在整个访谈中，我会问您一系列问题，我们鼓励您自由表达您的意见和观点。因为这不是一个当面访谈，所以我们也鼓励您用 QQ 表情来表达您的情绪。 在访谈过程中，如果我对你所说的内容有疑问或需要您澄清一个主题或概念，我会问您。

Researcher 13:45:19
Are you ready?
您准备好了吗？

Informant 002 13:45:27
Yes.
我准备好了。

Researcher 13:45:31
"Flow experience" has been used by psychologist to describe a state of mind experienced by people who are deeply involved in an activity. Instance, sometimes while playing videogames, the player's action and awareness are merged, and he/she is totally connected on the gaming tasks at hand. In this state, the player loses his/her consciousness, and his/her perception of time becomes faster or slower than usual. Also, the player perceives a feeling of being in control, which empowers him/her from the fear of failure.
心理学家使用"心流体验"来描述深度参与某项活动的人所经历的心理状态。 例如，有时玩家在玩电子游戏时，他/她的动作和意识会融为一体，并且他/她完全关注手头的游戏任务。 在这种状态下，玩家失去他/她的自我意识，他/她对时间的感知变得比平时更快或更慢。 此外，玩家会感受到一种掌控全局的感觉，这使他/她免于对失败的恐惧。

Researcher 13:45:32
Think about your own gaming experience for a moment. Have you ever experienced flow while playing videogames?
请回想一下您自己的游戏经验。 您玩电子游戏时有没有经历过心流体验？

Informant 002 13:46:05
(I) have the similar experience.
有类似经历。

Researcher 13:46:10
Please tell me what happened when you came to the flow state? I mean your behavioural and psychological activities during this course.
请告诉我您在进入到心流体验的时候发生了什么？ 我的意思是您在这个过程中的行为和心理活动。

Informant 002 13:46:29
It is very focused and I feel that the time passes very fast.
就是会非常的专注，感觉时间过的很快。

Informant 002 13:46:37
An immersive experience.
沉浸式的一种体验。

Informant 002 13:46:51
However, it has been a long time without this feeling.
不过我最近。已经很久没有这种感觉了。

Researcher 13:47:01
Why haven't you felt this for a long time?
为什么很久没有这种感觉了呢？

Informant 002 13:47:07
I had this experience more frequently when I played on the computer.
还是在电脑端玩的时候会更多的有这种体验。

Researcher 13:47:28
Don't you play games on the computer now?
现在不经常在电脑端玩游戏了吗？

Informant 002 13:47:36
Now I play more on mobile platform. The game lasts for a short time. So even there is this feeling, the duration is short.
现在更多玩的是手机端的游戏。游戏本身持续的时间很短，所以就算有这种感觉，

持续的时间也很短。

Informant 002 13:47:46
The computer has not been opened for a long time.
电脑已经很久没有开过了。

Researcher 13:48:08
I see.
原来如此。

Informant 002 13:48:09
The same for the mobile games. The duration of each game is usually around 15 minutes. During this time, you will be very focused on how to win this game.
手机上玩也会这样，就是每盘游戏持续的时间一般在 15 分钟左右，在这个时间当中你会非常的专注想如何去赢这盘游戏。

Researcher 13:48:19
I understand.
我明白了。

Researcher 13:48:21
Have you had an anxious experience during the game?
您在游戏时有过焦虑的经历吗？

Informant 002 13:48:49
I don't quite understand what anxiety means here.
我不太明白焦虑在这里是指什么

Researcher 13:49:09
It refers to an anxious mentality.
指的是焦虑的心态

Informant 002 13:49:22
Sometimes I have, because in addition to playing games, you have other things to do.
有的时候会有，因为除了玩游戏，你还有其他的一些事情要做。

Informant 002 13:49:35
Psychologically you want to play games, and in the same time you want to complete other things. But playing games takes a lot of time.
在心理上你会既想玩游戏又想完成其他的事情，但是玩游戏也会占据很多的时间。

Informant 002 13:49:49
So there will be contradictory and anxious mentality.
所以会有矛盾和焦虑的事情。

Informant 002 13:49:52
心情

Researcher 13:50:49
I see. The last time we talked about the horse you bought in Baozou Yingxiong Tan.
原来如此。我们上一次提到了暴走英雄坛里您买了一个马。

Researcher 13:51:05
which serves for speeding up the gaming process.
作用是加快游戏进度。

Informant 002 13:51:09
嗯

Researcher 13:51:24
Have you had any feeling of anxiety before buying this horse?
请问如果您在购买这个马之前有过焦虑的感觉吗？

Informant 002 13:51:46
I entangled in whether or not to buy.
有纠结过要不要买

Informant 002 13:51:49
There was no anxiety.
没有焦虑

Researcher 13:52:19
Do you think the functionality of this horse is to reduce the difficulty of the game?
您觉得这个马的作用是降低游戏难度吗？

Informant 002 13:52:34
To increase the efficiency of the game.
提高游戏的效率

Informant 002 13:52:59
There is no changing regarding to the game difficulty, but the time difficulty has been changed.
游戏难度没有变化　时间难度降低

Researcher 13:53:29
In your mind, how much time spent forms part of the difficulty of the game?
在您的观念中，花费时间的多少也是游戏难度的一部分吗？

Informant 002 13:54:04
Yes, I may not have much time to do the repetitive things.
是的，我可能没有太多时间去做重复的事情。

Informant 002 13:54:21
Some things are repeated, and I hate doing repetitive things.
有些东西是重复进行的，我很讨厌做重复的事情。

Informant 002 13:54:35
If there is a way to improve efficiency. That makes sense to me.
如果有办法提高效率。那对我来说还是有意义的。

Researcher 13:54:56
What is the purpose of improving efficiency?
请问提高效率的目的是什么呢？

Informant 002 13:55:29
To quickly be stronger.
迅速表牛逼

Informant 002 13:55:33
变

Researcher 13:55:39
What I want to ask is, what are the differences about the game experience before improving the efficiency and after improving the efficiency?
我想问的是，提高效率以前和提高效率之后的游戏体验有什么区别？

Informant 002 13:56:01
There are differences.
有区别

Informant 002 13:56:22
Before the efficiency (is) improved. The gaming experience is difficult, and after improving the (efficiency), the gaming experience is relatively simple.
提高之前的。游戏体验比较艰难，提高之后的游戏体验就比较简单。

Informant 002 13:56:56
The speed of completing some daily (tasks) will be faster.
有些日常性的东西。完成的速度就会变快。

Informant 002 13:57:11
Then you are able to enter the next stage of the game, and go for the harder targets.
然后你就可以进入游戏的下一阶段，去攻略更难的目标。

Researcher 13:58:15
I see.
原来如此。

Researcher 13:58:56
We shall return to the anxious game experience that we just mentioned. What would you do if you had an anxious experience in a game?
我们回到刚才说的焦虑的游戏体验。如果您在游戏中有焦虑的经历，您会怎么做?

Informant 002 13:59:12
Keep playing the game.
继续玩游戏。

Researcher 13:59:46
Continue to play the game will let you enter the state of flow experience as we just talked about?
继续玩游戏的话会让您进入我们刚才所谈到的心流体验的状态吗？

Informant 002 14:00:18
Yes, but (I) may re-enter the state of anxiety intermittently.
会的，但是会接歇性的重新进入焦虑状态。

Researcher 14:00:38
Ok. Under this circumstance, will you buy in-game goods to alleviate anxiety?
好的。那么在这种情况下，您会购买游戏内的商品来缓解焦虑吗？

Informant 002 14:00:49
No.
不会

Informant 002 14:01:03
They are things from two different levels.
这是两个不同层面的事情

Researcher 14:01:09
Umm, please continue.
恩，您说

Informant 002 14:01:39
This matter cannot be solved through purchasing.
就是这件事情不能通过消费来解决。

Informant 002 14:01:50

Because the purchasing behaviour does not solve the source of the problem.
因为消费这个行为没有解决问题的根源。

Researcher 14:02:47
I understand. For you. The root of anxiety is that you can't do other things when you play games. But there is a need to entertain through gaming. There is such a contradiction, is it?
我明白了，对您来说。焦虑的根源是玩了游戏就不能做其它事情。但是又有想在游戏内娱乐的需求。有这样的矛盾，是吗？

Informant 002 14:02:57
Yes.
嗯

Informant 002 14:03:13
I rarely get anxious because of the game itself.
我很少因为游戏本身产生焦虑。

Researcher 14:03:15
Is there a game mechanism that makes you feel anxious?
有没有游戏机制让您觉得焦虑的情况？

Informant 002 14:03:57
(I would feel anxious) when conducting the raid in a dune of World of Warcraft for the first time.
wow 开荒会有一些

Informant 002 14:04:11
Because things have not been experienced, there will be no anxiety after the experience.
因为事情没有经历过，经历过以后也就不会有焦虑的心情。

Informant 002 14:04:24
It may not be anxious. It’s a worry about the unknown.
也不能算焦虑吧，是算对未知的担心

Informant 002 14:04:28
Afraid.
害怕

Informant 002 14:04:47
Now I play more the competitive games. There doesn't exist mechanism that makes me anxious.
我现在玩的更多的是竞技对战类的游戏。不存在游戏机制让我焦虑的情况

Researcher 14:04:54
Ok. Which type of games do you usually play more? Do you have more games that do not include in-game goods or do you play more free games?
好的。您一般玩哪种游戏比较多？是不包含游戏内购的游戏玩得比较多还是免费游戏玩得比较多？

Informant 002 14:05:11
I play more free games.
我玩免费游戏比较多。

Informant 002 14:05:19
Because the threshold is relatively low
因为门槛比较低。

Informant 002 14:05:40
The total cost of trail is low.
试错的全本。

Informant 002 14:05:48
成本低

Researcher 14:06:54
We talked about the cost of trial. If you feel that the experience of this attempt is not very good, what would you do?
我们谈到了试错的成本。如果你能觉得这个尝试的体验不太好，您会怎么做？

Informant 002 14:07:07
Abandon the game.
弃游

Researcher 14:07:44
What kind of gaming experience will make you feel like you want to give up the game?
什么样的游戏体验会让您感到想要放弃游戏呢？

Informant 002 14:08:18
When the system is too complicated.
系统太复杂。

Researcher 14:08:50
When you feel that the system is too complicated, what is your inner feeling?
感觉系统太复杂的时候，您内心是什么感受？

Informant 002 14:09:08

So complicated. .
好麻烦。。

Informant 002 14:09:10
Do not want to play.
不玩了

Researcher 14:09:31
Do you have the anxious feeling?
会不会有焦虑的感觉?

Informant 002 14:09:54
No.
不会

Informant 002 14:10:09
(I) just feel it’s a trouble.
就是觉得麻烦

Researcher 14:10:12
Ok. Have you had a boring experience during the game?
好的。您在游戏时有过无聊的经历吗？

Informant 002 14:10:16
I don't play (the games) that has violated my original intention of playing them.
违背了我玩游戏的初衷就不玩了。

Informant 002 14:10:23
There are many situations
有好多种情况。

Informant 002 14:10:33
After playing some games for a long time, I naturally don't play them anymore. However, I will go back to play them in the future.
有些游戏玩的时间久了，自然而然就不玩了，但是过段时间还会回去。

Researcher 14:10:34
Well, you can mention them one by one.
恩，您可以一条条地说、

Informant 002 14:10:48
Some are boring at the first place. I just don't play them anymore.
有些是一开始玩的就觉得没意思。就不玩了。

Informant 002 14:11:21
There are still some things can be found after playing.
还有一些事。玩起来以后发现

Informant 002 14:11:38
If the gap between ordinary players and RMB players is too large, then I don't play.
普通玩家跟人民币玩家差距实在太大就不玩了

Informant 002 14:11:50
More or less like this.
差不多就这样。

Researcher 14:11:56
Ok. Why do you think that after playing some games for a long time, they naturally become not fun?
好的。为什么您会觉得有些游戏玩得时间久了，就自然地不好玩了呢？

Informant 002 14:12:17
I know. Like Clash of clans, I have been playing it for five years.
我知道啊，就比如部落冲突，我已经玩了五年了。

Informant 002 14:12:25
I have given up many times and went back many times to play.
已经放弃过好多次又回去玩。

Informant 002 14:12:33
The game itself has a certain rhythm.
就是游戏本身也是有一定的节奏的。

Informant 002 14:12:50
When you have adapted to something that appealed to you, it just lose the original appeal, and you wouldn't always think about it.
当你适应了那些很吸引你的东西以后，就失去了本来的吸引力，你就不会一直想。

Researcher 14:13:19
Does Clash of clans sometimes updates new content?
请问部落冲突有时常更新新的内容吗？

Informant 002 14:13:37
有更新。差不多一个季度更新一次。

Researcher 14:14:18
Do you think the updated contents are challenging?

您觉得更新的内容富有挑战性吗？

Informant 002 14:15:14
If we only talk about Clash of clans, its updated contents generally have two directions.
更新的内容一般有两个指向如果我们只说部落冲突的。

Informant 002 14:15:26
One direction is to reduce the difficulty for the beginners.
一个是降低入门玩家的难度

Informant 002 14:15:40
Another is to increase the upper limit of the game.
一个是提高游戏的上限

Informant 002 14:16:05
This game itself is challenging.
这个游戏本身就是富有挑战性

Researcher 14:17:37
I see. You just mentioned that you have given up many times and went back many times to play the game. What is the reason?
原来如此。刚才您谈到，已经放弃过好多次又回去玩。请问是什么原因呢？

Informant 002 14:18:15
I still have the nostalgia.
还是有情怀在

Researcher 14:18:55
What kind of opportunity can attract you back to a game that you have already given up?
什么样的契机能吸引您回到一个本来已经放弃的游戏？

Informant 002 14:18:58
For example, I asked my colleague that which is more delicious, the oversea McDonald's or the burger shop at his door.
就比如我问过我同事是国外的麦当劳好吃。 还是他家门口的汉堡小店好吃

Informant 002 14:19:18
He said that both of them are delicious, but he still like the one at his door.
他说都好吃，但是还是喜欢吃门口的

Informant 002 14:19:22
Because (he) used to.

因为 used to

Informant 002 14:19:40
Sometimess I get really boring. Sometimes I see some updates and want to go back to check.
有的时候是闲的无聊了。有的时候是看到一些更新，想回去看看。

Researcher 14:19:52
Ok. . Although you have given up the game, will you still miss this game?
原路如此。虽然说您已经放弃了游戏，但是心里依然会挂念这款游戏吗？

Informant 002 14:19:59
冬

Informant 002 14:20:03
Yes.
会的

Informant 002 14:20:09
(Yes, yes)

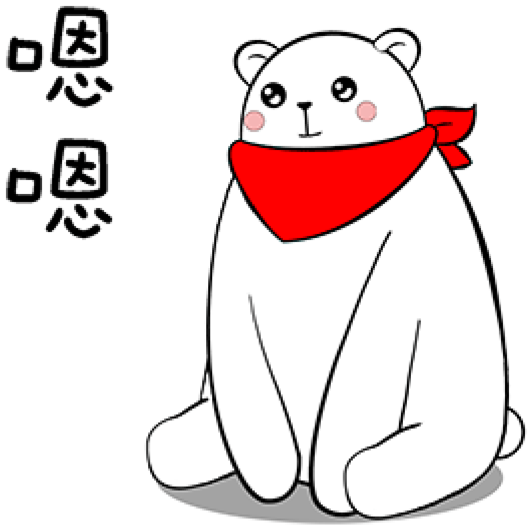

Researcher 14:20:32
You have just mentioned “When you have adapted to something that appealed to you, it just lose the original appeal, and you wouldn't always think about it.”
您刚才提到"当你适应了那些很吸引你的东西以后，就失去了本来的吸引力，你就不会一直想"

Informant 002 14:21:16
Yes.

嗯

Researcher 14:21:17
Does this "adaptation" process have anything to do with your gaming skills in the game?
请问这个"适应"的过程，和您在游戏里的游戏水平有关系吗？

Informant 002 14:22:07
Yes.
有

Informant 002 14:22:32
As you repeat more and more what you were initially interested in, the difficulty goes lower and lower.
当你越来越多重复你本来感兴趣事情。难度就会越来越低。

Informant 002 14:22:52
Additionally, your equipment is getting better and better.
然后本身你的装备也会越来越好。

Informant 002 14:22:59
Like the first raid.
就像开荒

Researcher 14:24:30
Can I divide the growth of such gaming skills into two aspects. On the one hand, your own gaming skill is improving. On the other hand, your equipment in the game is constantly accumulating.
请问我能不能把这样子游戏水平的增长分成两方面：一方面是您自身的游戏水平在提高，另一方面是您在游戏内的各种装备不断地在积累。

Informant 002 14:24:40
Yes.
是

Informant 002 14:24:46
excatly
Exactly

Researcher 14:24:49
Ok. Have you ever had the flow experience when playing the game Clash of clans?
好的。请问您有在玩部落冲突这款游戏的时候有过心流体验吗？

Informant 002 14:26:11
Yes.
有

Informant 002 14:26:39
Yes. I think that it occurs in all the games in which you can play continuously.
有的我觉得所有的你会玩下去的游戏都会有这种状况。

Researcher 14:27:26
I see. Let's go back to the topic of the boring experience. What would you do if you had a boring experience in the game?
原来如此。我们回到刚才关于无聊体验的话题，如果您在游戏中有无聊的经历，您会怎么做?

Informant 002 14:27:41
Close it and don’t play it.
关了不玩了

Researcher 14:28:07
It refers to temporary abandonment or permanent abandonment?
这边说的不玩了是暂时性不玩了还是永久性的放弃？

Informant 002 14:28:32
It’s temporary abandonment. The temporary abandonment in many cases led to permanent abandonment.
是暂时性的不玩了，如果暂时性的不玩，发生了很多就会长期的不玩。

Informant 002 14:28:43
If I have the nostalgia about (the game) itself. After a period of time, I may back to play it. It’s a cycle.
如果这个本身又有情怀在里面过一段时间之后我就又会回去玩，是一个循环。

Researcher 14:29:52
I see. How do you define temporary abandonment and permanent abandonment?
原来如此。您怎么定义暂时性不玩和长期不玩呢？

Researcher 14:30:04
What is the difference between the two for you?
这两者对您来说有什么区别？

Informant 002 14:30:20
If you don' t play it for 1-2 days or 3-4 days, it is called temporary abandonment.
一两天或者三四天不玩就要暂时性不玩。

Informant 002 14:30:35
Permanent abandonment refers to that even I have seen the icon of the game, I'm unwilling to open it.
长期性的不玩，就是我看到这个游戏的图标，我都懒得去打开它。

Researcher 14:31:25
Will you buy in-game goods to alleviate boredom?
您会购买游戏内的商品来缓解无聊吗？

Informant 002 14:31:41
No, because they are things from different levels.
不会，因为那是两个层面的东西。

Researcher 14:32:26
Well, I want to know what these two levels represent.
恩，我想了解一下这两个层面分别代表了什么？

Informant 002 14:32:47
(We mention this) dividing into two sorts of games.
分两种游戏来说。

Researcher 14:32:52
Well.
恩恩

Informant 002 14:33:12
Some (games) are story type, which requires long-term accumulation to play. The just right game difficulty is important. Neither too difficult nor too easy is correct.
有些是剧情通关类，需要长时间的积累去玩的。恰到好处的游戏难度是新一。玩家的必要条件太难或者太简单都不行。

Informant 002 14:34:09
Like this kind of game. Consumption generally does not break the difficulty. It only speeds up the progress of the game.
像这类游戏。 消费一般并不会打破难度。只会加速游戏的进展。

Informant 002 14:34:21
Very limited.
非常的有限

Researcher 14:35:09
If you have cleared a game of story type, will you feel boring?
如果剧情通关类的游戏玩通关了之后，您会觉得无聊吗？

Informant 002 14:35:11
In terms of the Battle game. Due to that it is necessary to be fair for both sides. Generally (they) only make changes to the appearance.
对战类的游戏。因为要做到对战双方的公平。一般只做外贸上的变更。

Informant 002 14:35:16
Yes.
是的。

Informant 002 14:35:29
So how to maintain the user's gaming time is pretty difficult
所以如何维护用户的游戏时长还挺难的

Researcher 14:35:57
Under this circumstance, if the game offers Expansion packages and/or Playable characters, will you purchase?
在这种情况下，如果游戏提供了扩展包或者可游玩角色，您会选择购买吗？

Informant 002 14:36:07
Yes.
会的

Informant 002 14:36:16
I have bought some DLC packages.
我买过一些 dlc 的包

Researcher 14:36:46
Ok. So in this case, will dlc packages help you to re-enter the state of flow experience?
好的。那么在这种情况下，dlc 包会帮助您重新进入心流体验的状态吗？

Informant 002 14:37:31
Yes.
会

Researcher 14:38:13
Ok. Once after having the flow experience, will you come back to the game to regain this experience?
好的。一旦获得过心流体验，您会想回到游戏中为了重新获得这种体验吗？

Informant 002 14:38:22
Yes.
是

Researcher 14:39:01

Have you had any experience of supportive purchasing? I mean purchasing in-game goods for supporting the game marker than acquiring the in-game goods themselves.
好的。您有没有过支持性购买的经历？我的意思是为了支持游戏开发商而购买游戏内商品，而不是为了获得游戏内商品本身。

Informant 002 14:39:30
No.
没有

Informant 002 14:39:40
I have not made a donation
我没做过捐赠

Researcher 14:40:22
What we are talking about here is not donating. Instead, we talk about purchasing the in-game goods, but the motivation is to support the game developer more than to the goods themselves.
我们这边说的不是捐赠。而是购买游戏内商品，但是动机是为了更多支持游戏开发商而不是获得游戏内商品本身。

Informant 002 14:40:37
No.
没有

Researcher 14:41:24
Ok. We just talked about the original intention of playing games. You said that "I don't play (the games) that has violated my original intention of playing them."
好的。我们刚才谈到了玩游戏的初衷。您说到"违背了我玩游戏的初衷就不玩了"。

Researcher 14:41:33
What is your original intention to play games?
请问您玩游戏的初衷是什么？

Informant 002 14:42:09
Naturally, there is an attraction.
自然而然的有那种吸引力

Informant 002 14:42:14
Which makes me always like to play.
就会一直想玩

Informant 002 14:42:25
Instead of bringing me all sorts of troubles.
而不是给我带来各种各样的麻烦。

Researcher 14:42:44
Do you think that the flow experience is related to the original intention of playing games?
您认为心流体验和您玩游戏的初衷有关系吗？

Informant 002 14:42:57
Yes, I think that is.
有的 我觉得就是

Researcher 14:43:10
What kind of relationship is it?
请问是什么样的关系？

Researcher 14:43:32
Oh, what you said is that your original intention of playing games is to acquire the flow experience?
噢，您说的是玩游戏的初衷就是为了获得心流体验？

Informant 002 14:43:52
Yes, that kind of immersive experience.
嗯 那种沉浸式的体验

Researcher 14:44:32
Ok. The interview is almost over. Do you have any ideas to add?
好的。访谈差不多要结束了。您还有什么观点需要补充吗？

Informant 002 14:45:29
No wonder I have played less recently.
怪不得我最近游戏玩的少

Informant 002 14:45:41
It turns out that fewer and fewer games have brought me this kind of experience.
原来是越来越少游戏给我带来这种体验了

Informant 002 14:45:51
I didn’t realise it.
我还没有意识到

Researcher 14:46:42
What factors do you think lead to the lack of this experience? It's due to that the game is too difficult and your game skill is too low. Or due to the game is too simple and your game skill is too high?
您认为是什么因素导致了您缺乏这种体验呢？是因为游戏太难，您的游戏水平太

低。还是游戏太简单，您的游戏水平太高呢？

Informant 002 14:47:21
Currently, some (gaming) patterns are just change a lot of skins.
现在的。有些模式越来越类似很多只是换皮

Informant 002 14:47:24
Not interesting
没意思

Researcher 14:48:33
Can I interpret as…
我能不能理解成

Researcher 14:48:57
Your skill for a determined type of game is already enough high, but the challenges of many games are similar?
您对于单一种类的游戏技能已经很高了，但是他们的游戏挑战都是差不多的？

Informant 002 14:49:04
Yes.
嗯

Researcher 14:49:07
Which lead you to be unable to enter the state of the flow experience?
所以导致了您无法进入心流体验的状态？

Informant 002 14:49:12
Yes.
嗯

Researcher 14:49:16
Is it boring at that moment?
这时候觉得无聊吗？

Informant 002 14:49:22
Yes.
嗯

Informant 002 14:49:29
Repetitive (things) are boring
重复性的都无聊

Researcher 14:49:31

I understand.
我明白了。

Researcher 14:49:34
These are all the questions. Thank you very much for participating in our research. Please confirm that your email address is XXXXXX@XXXXXX.com, because later we will send the JD electronic gift card to this address.
这就是全部的问题。 非常感谢您参与我们的研究。请确认您的电子邮件地址是XXXXXX@XXXXXX.com，因为稍后我们把京东电子礼品卡发送到这个地址。
