# Supplementary material for: A grounded theory approach to understanding in-game goods purchase
Source: PLoS One. 2022 Jan 27;17(1):e0262998. doi: 10.1371/journal.pone.0262998 (PMC8794092; doi:10.1371/journal.pone.0262998)
Supplement: S1 File — (ZIP) [file pone.0262998.s001.zip › Transcript 14.pdf]

Interview: 014

Informant: 004

Please note that the original transcript is in Simplified Chinese. The English translation is for internal communication among the author of this research, and it is not proofread. Potential linguistic errors may exist in the English translation.

Researcher 13:24:08
Thank you for your willingness to participate and be interviewed here. My name is XXX XXX, and I'm a PhD student in the XXX University of XXX(XXX). Currently, I'm working on a research project which focuses on videogame players' purchase motivations of in-game goods. Throughout this interview, I will ask you a series of questions and you are encouraged to express your opinions freely with emoticons. If I have questions about what you've said or need clarification about a topic or concept, I'll ask you.
感谢您愿意参加并在此接受采访。 我叫 XXX，我是市场营销学的博士生，现在我在 XXX 大学就读。 目前，我正在开展一个研究项目，专注于电子游戏玩家对游戏内购买项目的购买动机。 在整个访谈中，我会问您一系列问题，我们鼓励您自由表达您的意见和观点。因为这不是一个当面访谈，所以我们也鼓励您用 QQ 表情来表达您的情绪。 在访谈过程中，如果我对你所说的内容有疑问或需要您澄清一个主题或概念，我会问您。

Researcher 13:24:14
Are you ready?
您准备好了吗？

Informant 004 13:24:17
Yes.
好了

Researcher 13:24:20
"Flow experience" has been used by psychologist to describe a state of mind experienced by people who are deeply involved in an activity. Instance, sometimes while playing videogames, the player's action and awareness are merged, and he/she is totally connected on the gaming tasks at hand. In this state, the player loses his/her consciousness, and his/her perception of time becomes faster or slower than usual. Also, the player perceives a feeling of being in control, which empowers him/her from the fear of failure.
心理学家使用"心流体验"来描述深度参与某项活动的人所经历的心理状态。 例如，有时玩家在玩电子游戏时，他/她的动作和意识会融为一体，并且他/她完全关注手头的游戏任务。 在这种状态下，玩家失去他/她的自我意识，他/她对时间的感知变得比平时更快或更慢。 此外，玩家会感受到一种掌控全局的感觉，这使他/她免于对失败的恐惧。

Researcher 13:24:26
Think about your own gaming experience for a moment. Have you ever experienced flow while playing videogames?
请回想一下您自己的游戏经验。 您玩电子游戏时有没有经历过心流体验？

Informant 004 13:24:51
Yes.
有

Researcher 13:24:58
Please tell me what happened when you came to the flow state? I mean your behavioural and psychological activities during this course.
请告诉我您在进入到心流体验的时候发生了什么？ 我的意思是您在这个过程中的行为和心理活动。

Informant 004 13:26:34
When playing LOL, (I equipped with)6 God Armors and feel that I can casually kill the opposite players, without the limitation of their levels. I would find five (people) to fight with them.
玩 LOL 的时候 6 神装感觉自己能随便杀对面高低能随便推，一个找五个人打

Informant 004 13:26:55
I feel that I'm (absolutely invincible).

感觉自己 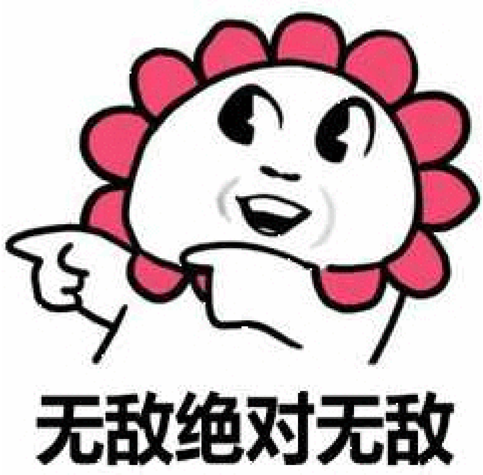

Researcher 13:27:02
Ok. Any other feelings need to be added?
恩恩。还有什么感受需要补充吗？

Informant 004 13:28:10
I feel that (although) I only have played a little time, half an hour has passed when checking the time.

感觉玩了一点点时间结果时间一看过了半小时

Researcher 13:28:34
Ok. We continue.
原路如此。我们继续。

Researcher 13:28:38
Have you had an anxious experience during the game?
您在游戏时有过焦虑的经历吗？

Informant 004 13:30:08
There is a clear opportunity to turn over the situation, (but) the teammates fatten the opposite (enemies).
有明明能翻盘结果队友浪，把对面送肥了

Informant 004 13:30:18
I would be anxious.
会很焦虑

Researcher 13:30:52
I see. At this time, will you perceive the difficulty or challenge of the game is greater than usual?
原来如此。这个时候您感知到的游戏难度或者挑战会比往常更大吗？

Informant 004 13:31:17
I would feel that I'm playing with four mental retards.
会感觉在和四个智障在玩、

Researcher 13:31:43
I understand. What do you do if you have an anxious experience in the game?
我懂了。如果您在游戏中有焦虑的经历，您会怎么做?

Informant 004 13:32:35
Hands off the keyboard, ignite a cigarette, and then communicate wildly.
双手离开键盘点上一根烟然后疯狂交流

Researcher 13:33:25
What is the meaning of "communicate wildly " here?
这里的"疯狂交流"具体是什么意思呢？

Informant 004 13:33:54
Swearing - -
脏话- -

Researcher 13:34:29
Oh, I haven't played the dota game before. Is the microphone opened while playing lol?
噢，我没有玩过 dota 类游戏。在玩 lol 的时候是开着麦克风的吗？

Informant 004 13:34:54
No, just typing.
不是就是打字

Researcher 13:35:10
Ok. What will happen after communicating wildly?
好的。疯狂交流之后会怎么做？

Informant 004 13:35:25
The voice function is available in DOTA2.
DOTA2 的话可以开语音

Informant 004 13:35:40
Play seriously – find an opportunity to turn over.
好好打- -找机会翻盘

Informant 004 13:35:58
If there is no such opportunity, then (I) communicate wildly till the end of the game.
没机会的话疯狂交流到游戏结束

Researcher 13:37:15
Ok. Under this circumstance, will you buy in-game goods to alleviate anxiety?
原来如此。在这种情况下，您会购买游戏内的商品来缓解焦虑吗？

Informant 004 13:38:08
Sometimes I will.
有时候会

Researcher 13:38:19
Under this circumstance, which types of in-game goods would you purchase in this case (Power-ups, Expansion packages, Playable characters, Cosmetics/Skins, Loot boxes, Time-savers)?
在这种情况下，您会购买哪些类型的游戏内商品呢？（增强道具，扩展包，可游玩的角色，装饰/皮肤，抽奖箱，省时道具）？

Informant 004 13:38:54
Power-ups, Expansion packages, Playable characters, Cosmetics/Skins, Loot boxes, Time-savers

增强道具，扩展包，可游玩的角色，装饰/皮肤，抽奖箱

Researcher 13:39:04
All of them?
都有吗？

Informant 004 13:39:13
Yes.
对

Researcher 13:39:57
Why do you think that buying these in-game goods can alleviate your anxiety in this situation?
为什么您觉得买这些游戏内商品在这种情况下能缓解您的焦虑情绪呢？

Informant 004 13:40:28
I (am) looking for an opportunity to fight back after being abused.
被对面虐了要找机会打回来

Researcher 13:41:01
Do you want to make yourself stronger?
是想让自己变得更强吗？

Informant 004 13:41:14
Yes.
对的

Researcher 13:41:33
Do you think the in-game goods help you to re-enter the flow state?
好的。您认为游戏内商品可以帮助您重新进入心流状态吗？

Informant 004 13:41:46
Yes.
会

Researcher 13:42:37
Ok. We just mentioned that you are anxious because of your teammates. In addition, is there any anxiety caused by the game mechanism itself?
好的。我们刚才提到了因为队友的原因导致了您焦虑。除此之外，有没有因为游戏机制本身的原因带给您焦虑感的情况？

Informant 004 13:43:21
For example, some games that can't be played without recharging money.

比如一些不充钱就不能玩的游戏

Informant 004 13:43:27
Especially mobile games.
特别是手游

Informant 004 13:43:41
In FGO, after (placing) an order, nothing is obtained.
FGO 一单下去什么都没有

Informant 004 13:43:57
At this moment, I would be very anxious.
这种时候就会很焦虑

Researcher 13:44:08
Can't play mean that the game mechanism itself determines its high difficulty?
不能玩的意思是游戏机制本身决定了它的的难度很大吗？

Informant 004 13:44:14
Yes.
对

Researcher 13:44:18
What does FGO mean?
请问 FGO 是什么意思？

Informant 004 13:44:48
Fate/Grand Order

Researcher 13:45:35
Ok. If it is anxiety for this reason, what do you do to relieve the anxiety?
好的。如果是因为这种原因产生的焦虑，您会怎么做去缓解焦虑？

Informant 004 13:46:33
In an order, the 458 is the best 5-star character, and the probability of getting this is 0.07%. If nothing is obtained after drawing, (I) either give up playing or continue playing.
一单的话是 458 最好的 5 星角色卡出的概率 0.07%，一单抽完什么都没要么不玩了，要么继续

Researcher 13:47:35
I have a lot of questions about this sentence. We talk one by one. First of all, what does "give up playing" is give up playing for a while or give up playing for a long time?

我对于这句话很多问题。我们一个个来。首先，这儿说的“不玩了”是暂时不玩还是长期不玩了？

Informant 004 13:47:48
Give up playing for a while.
暂时不玩了

Researcher 13:48:52
For you, how do you define "Give up playing temporarily"? Is this different to the "Give up playing permanently"?
对于您来说，您怎么定义"暂时不玩了"。和“长期不玩了”有什么差异呢？

Informant 004 13:50:00
Give up playing temporarily refers to abandon (the game) for two days to adjust the mood, and "Give up playing permanently" means uninstall the game and abandon it.
暂时不玩就是过两天在玩调节一下心情，长期不玩就是卸游戏退游了

Researcher 13:50:21
Will you still miss this game when you give up playing it for a while?
暂时不玩的时候心里依然会挂念这个游戏吗？

Informant 004 13:50:42
I won't play anything else.
不会玩别的了

Researcher 13:51:53
I understand. When we mentioned the relief of this type of anxiety, we talked about "one order". Does it mean to alleviate anxiety through the use of Loot boxes type in-game goods?
我了解了。我们刚才说的缓解这类型的焦虑的时候谈到了“一单”，这里指的是通过抽奖箱类型的游戏内商品来缓解焦虑吗？

Informant 004 13:52:54
Not what I mean is a loot boxes type in-game goods make people anxious.
不是我的意思是抽奖箱类型的游戏会使人焦虑

Researcher 13:53:21
Oh, Ok.
噢噢好的。

Researcher 13:53:36
Under this circumstance, will you buy in-game goods to alleviate anxiety?
那在这种情况下，您会购买游戏内的商品来缓解焦虑吗？

Informant 004 13:54:08
Sometimes I will.
偶尔会

Researcher 13:54:21
Under this circumstance, which types of in-game goods would you purchase in this case (Power-ups, Expansion packages, Playable characters, Cosmetics/Skins, Loot boxes, Time-savers)?
在这种情况下，您会购买哪些类型的游戏内商品呢？（增强道具，扩展包，可游玩的角色，装饰/皮肤，抽奖箱，省时道具）？
Informant 004 13:54:34
Expansion packages.
扩展包

Informant 004 13:55:34
Sometimes the expansion packages reward some things that only can be obtained by drawing.
有的时候拓展包会送一下原本抽奖才会送的东西

Researcher 13:56:02
Wait. Is the extension package we are talking about like dlc packages?
等下。我们这边说的扩展包是像 dlc 包那样的还是？

Informant 004 13:56:43
It should be similar to Power-ups.
应该类似于增强道具

Researcher 13:57:29
I see. Moreover, you have just mentioned "In an order, the 458 is the best 5-star character, and the probability of getting this is 0.07%. If nothing is obtained after drawing, (I) either give up playing or continue playing.". Here, "continue playing" means continue playing the game?
我明白了。另外刚才您谈到"一单的话是 458 最好的 5 星角色卡出的概率 0.07%，一单抽完什么都没要么不玩了，要么继续"。这里的"要么继续"指的是继续玩这个游戏的意思吗？

Informant 004 13:58:02
Continue drawing until I get what I want.
继续抽，抽到想要的东西为止

Researcher 13:58:31
In this process, are you spending RMB to buy Loot boxes?
在这个过程中是在花人民币购买抽奖箱吗？

Informant 004 13:58:40
Yes.
对

Researcher 13:59:17
I understand. In this case, do you think these Loot boxes and the obtained Power-ups can help you re-enter the flow state?
我懂了。在这种情况下，您认为这些抽奖箱和得到的增强道具可以帮助您重新进入心流状态吗？

Informant 004 13:59:30
Yes.
对

Researcher 13:59:40
Ok. Let’s change the subject.
好的。我们换个话题。

Researcher 13:59:43
Have you had a boring experience during the game?
您在游戏时有过无聊的经历吗？

Informant 004 13:59:55
Yes.
对

Researcher 13:59:58
Under what circumstances do you usually have?
通常在什么情况下有？

Informant 004 14:00:53
For example, when there is no progress or when (I'm) collecting materials every day: some operations that are very mechanical.
比如说毫无进展或者天天都在刷材料这种很机械的操作的时候

Researcher 14:01:21
Ok. If you have a boring experience in the game, what do you do to ease the boring experience?
原来如此。如果您在游戏中有无聊的经历，您会怎么做去缓解无聊的体验?

Informant 004 14:01:33
(I would) change a game to play.

换个游戏玩

Informant 004 14:01:56
Or find someone to PK or SOLO.
或者找人 PK 或者 SOLO

Researcher 14:02:30
In this case, the game that was played before is in a " Give up playing temporarily" state or " Give up playing permanently" state?
在这种情况下，之前玩的那个游戏是“暂时放弃”的状态还是“永久放弃”的状态？

Informant 004 14:02:44
Temporarily
暂时

Researcher 14:03:23
Ok. When you PK or SOLO with someone, do you think that the challenge or difficulty of the game is higher than operating mechanically?
好的。找人 PK 或者 SOLO 的时候，您有没有觉得游戏挑战或者难度要比机械操作的时候更高？

Informant 004 14:03:38
Yes.
会

Researcher 14:03:59
Ok. Also, would you buy in-game goods to ease boredom?
好的。另外，您会购买游戏内的商品来缓解无聊吗？

Informant 004 14:04:07
No.
不会

Researcher 14:04:55
Ok. Seeking for PK with someone or SOLO will help you enter to the flow state?
好的。找人 PK 或者 SOLO 这种方式会帮助您进入心流状态吗？

Informant 004 14:05:03
Yes.
会

Researcher 14:05:27
Once after having the flow experience, will you come back to the game to regain this experience?
一旦获得过心流体验，您会想回到游戏中为了重新获得这种体验吗？

Informant 004 14:05:42
Yes.
会

Researcher 14:05:55
Have you had any experience of supportive purchasing? I mean purchasing in-game goods for supporting the game marker than acquiring the in-game goods themselves.
好的。您有没有过支持性购买的经历？我的意思是为了支持游戏开发商而购买游戏内商品，而不是为了获得游戏内商品本身。

Informant 004 14:08:03
No.
没有

Researcher 14:08:33
Is the flow experience your purpose of playing videogames?
好的。请问获得心流体验是否是您玩电子游戏的目的？

Informant 004 14:08:35
But I did buy the physical peripheries of game developers.
不过买过游戏开发商出的实体周边

Informant 004 14:08:46
Yes.
是

Researcher 14:09:31
I see. I noticed the behaviour of buying physical peripheries. Do you think this has something to do with the flow experience?
原来如此。我注意到购买实体周边这个行为，您觉得这和心流体验有关系吗？

Informant 004 14:10:44
Yes, the potential consciousness tells me that I can (reach a state of) the combination of man and machine.
有，潜在意识告诉我我能人机合一

Informant 004 14:11:04
Maybe I’m relatively Chūnibyō.
可能我比较中二吧

Researcher 14:11:17
I see~
原来如此~

Researcher 14:11:46
That is to say, the game in which you had flow experience in the past leads you to purchase the physical peripheries. Shall I interpret like this?
也就是过去在某个游戏内有过心流体验导致了您购买这个游戏的实体周边，能这样理解吗？

Informant 004 14:12:08
Yes.
能

Researcher 14:12:29
I understand. The interview is almost over. Do you have any ideas to add?
我明白了。访谈差不多要结束了。您还有什么观点需要补充吗？

Informant 004 14:14:56
For example, the good artwork work of the game may attract me to play, Moreover, if the balance of the game mechanism has certain operability, which makes me have the flow experience, it would stimulate me to draw Loot boxes or buy peripheries.
比如说游戏的美工做的好会吸引我去玩，还有游戏的内容机制做的平衡有一定的操作性会让我产生心流体验回刺激我去开箱或者购买周边

Researcher 14:15:57
I see. Here balance of the game mechanism refers to the balance between game difficulty and your gaming skill?
原路如此。这里的游戏的内容机制做的平衡指的是游戏难度和您的游戏水平达到平衡的状态吗？

Researcher 14:17:35
There is a small problem: what is the difference drawing loot boxes after having flow experience and drawing loot boxes when it's boring?
有一个小问题：产生心流体验后的开箱和在无聊时候的开箱，这两者有什么差异？

Informant 004 14:17:46
It refers to the restrain power among roles in the game or the systems?
不是就是说游戏的角色克制或者体制之间的差距

Researcher 14:18:21
Ok. I understand.

好的。我明白了。

Informant 004 14:18:25
The drawing loot boxes when it's boring occurs in many cases when I want to collect all roles or equipment.
有无聊时候的开箱大多数是想凑齐角色或者是凑齐套装

Researcher 14:19:03
Emmm...What about drawing loot boxes after having flow experience?
Emmm...那在获得心流体验后的开箱呢？

Informant 004 14:19:07
Drawing loot boxes after having flow experience occurs when I feel I'm lucky and I want to try more.
心流体验的时候是感觉运气来了试试手气

Researcher 14:19:26
Oh! Is that the sort of invincible feeling as you said?
噢！就是刚才说的那种战无不胜的感觉？

Informant 004 14:20:00
Yes.
对啊

Researcher 14:20:13
These are all the questions. Thank you very much for participating in our research. Please confirm that your email address is XXXXXX@XXXXXX.com, because later we will send the JD electronic gift card to this address.
这就是全部的问题。 非常感谢您参与我们的研究。请确认您的电子邮件地址是XXXXXX@XXXXXX.com，因为稍后我们把京东电子礼品卡发送到这个地址。
