# Supplementary material for: A grounded theory approach to understanding in-game goods purchase
Source: PLoS One. 2022 Jan 27;17(1):e0262998. doi: 10.1371/journal.pone.0262998 (PMC8794092; doi:10.1371/journal.pone.0262998)
Supplement: S1 File — (ZIP) [file pone.0262998.s001.zip › Transcript 15.pdf]

# Interview: 015

# Informant: 009

*Please note that the original transcript is in Simplified Chinese. The English translation is for internal communication among the author of this research, and it is not proofread. Potential linguistic errors may exist in the English translation.*

Researcher 15:16:39
Thank you for your willingness to participate and be interviewed here. My name is XXX XXX, and I'm a PhD student in the XXX University of XXX(XXX). Currently, I'm working on a research project which focuses on videogame players' purchase motivations of in-game goods. Throughout this interview, I will ask you a series of questions and you are encouraged to express your opinions freely with emoticons. If I have questions about what you've said or need clarification about a topic or concept, I'll ask you.
感谢您愿意参加并在此接受采访。 我叫 XXX，我是市场营销学的博士生，现在我在 XXX 大学就读。 目前，我正在开展一个研究项目，专注于电子游戏玩家对游戏内购买项目的购买动机。 在整个访谈中，我会问您一系列问题，我们鼓励您自由表达您的意见和观点。因为这不是一个当面访谈，所以我们也鼓励您用 QQ 表情来表达您的情绪。 在访谈过程中，如果我对你所说的内容有疑问或需要您澄清一个主题或概念，我会问您。

Researcher 15:16:47
Are you ready?
您准备好了吗？

Informant 009 15:17:31
Yes.
好的

Researcher 15:18:31
Ah, now you can do the interview, can't you? I was afraid that you had other things.
啊，现在是能进行访谈的状态对吧？我怕你还有其它的事情

Researcher 15:18:37
Only to confirm.
我确认一下。。。

Informant 009 15:18:57
Yes, I can.
可以

Researcher 15:19:01
Ok.

Ok 好的。

Researcher 15:19:07
“Flow experience” has been used by psychologist to describe a state of mind experienced by people who are deeply involved in an activity. Instance, sometimes while playing videogames, the player’s action and awareness are merged, and he/she is totally connected on the gaming tasks at hand. In this state, the player loses his/her consciousness, and his/her perception of time becomes faster or slower than usual. Also, the player perceives a feeling of being in control, which empowers him/her from the fear of failure.
心理学家使用“心流体验”来描述深度参与某项活动的人所经历的心理状态。 例如，有时玩家在玩电子游戏时，他/她的动作和意识会融为一体，并且他/她完全关注手头的游戏任务。 在这种状态下，玩家失去他/她的自我意识，他/她对时间的感知变得比平时更快或更慢。 此外，玩家会感受到一种掌控全局的感觉，这使他/她免于对失败的恐惧。

Researcher 15:19:13
Think about your own gaming experience for a moment. Have you ever experienced flow while playing videogames?
请回想一下您自己的游戏经验。 您玩电子游戏时有没有经历过心流体验？

Informant 009 15:20:03
Slower perception of time. (I am) too dedicated, (and always forget the time while playing)
对时间感知比较慢 太投入了 一玩玩过头了 l

Researcher 15:20:24
Yes. You have had such experience, right?
恩恩。有过这样的体验对吧？

Informant 009 15:20:39
Yes.
对的

Researcher 15:20:44
Please tell me what happened when you came to the flow state? I mean your behavioural and psychological activities during this course.
请告诉我您在进入到心流体验的时候发生了什么？ 我的意思是您在这个过程中的行为和心理活动。

Informant 009 15:21:55
For example: starting the game at 8:00, and (I was) playing rounds after rounds. During this course, I thought the time remained at 11 o'clock.

比如：8:00 开始打游戏 打了一盘又继续打 打着打着 还以为时间停留在 9 点 哪知道已经 11 点了

Informant 009 15:22:12
Sometimes I know that it's late but I can't stop.
有时明知道晚了 还是停不下来

Researcher 15:22:39
I see. Is the feeling that you always want to continue playing?
原来如此，就是一直想继续游戏的感觉？

Researcher 15:37:53
If you are busy today, we can arrange at other times.
如果今天忙的话，我们能约在其它时间。

Researcher 14:53:29
Hi! I'm coming.
Hi！我来了

Informant 009 14:54:23
Hello.
Hello

Researcher 14:55:03
Hello. The last time we mentioned the flow state. Let's return to its definition:
您好。我们上一次提到了心流体验这个状态。我们再来回归一下它的定义：

Researcher 14:55:05
"Flow experience" has been used by psychologist to describe a state of mind experienced by people who are deeply involved in an activity. Instance, sometimes while playing videogames, the player's action and awareness are merged, and he/she is totally connected on the gaming tasks at hand. In this state, the player loses his/her consciousness, and his/her perception of time becomes faster or slower than usual. Also, the player perceives a feeling of being in control, which empowers him/her from the fear of failure.
心理学家使用"心流体验"来描述深度参与某项活动的人所经历的心理状态。 例如，有时玩家在玩电子游戏时，他/她的动作和意识会融为一体，并且他/她完全关注手头的游戏任务。 在这种状态下，玩家失去他/她的自我意识，他/她对时间的感知变得比平时更快或更慢。 此外，玩家会感受到一种掌控全局的感觉，这使他/她免于对失败的恐惧。

Informant 009 14:55:24
For example: starting the game at 8:00, and (I was) playing rounds after rounds. During this course, I thought the time remained at 11 o'clock.
比如：8:00 开始打游戏 打了一盘又继续打 打着打着 还以为时间停留在 9 点 哪知道已经 11 点了

Researcher 14:55:46
Last time, when being asked if you had flow experience when playing video games? Your answer was "For example: starting the game at 8:00, and (I was) playing rounds after rounds. During this course, I thought the time remained at 11 o'clock."
上一次，当被问起 您玩电子游戏时有没有经历过心流体验？的时候，您回答了"比如：8:00 开始打游戏 打了一盘又继续打 打着打着 还以为时间停留在 9 点 哪知道已经 11 点了""有时明知道晚了 还是停不下来"

Researcher 14:55:55
Is there any feeling to add?
请问还有什么感受要补充吗？

Informant 009 14:56:03
Yes.
对的

Informant 009 14:56:21
No.
没了

Researcher 14:56:47
Have you had an anxious experience during the game?
好的。您在游戏时有过焦虑的经历吗？

Informant 009 14:58:45
Taking playing team games as an example. The teammates responded very slowly, and he/she didn't listen to the command. You told him/her to use shield soldiers but he/she used gunman instead. Sometimes someone in the team put the whole team in the pit because he/she was too selfish and wanted to swipe the scores.
有 比方说：打团队赛 队友反应迟钝 不听指挥 让他进盾他进了队枪兵 感觉被坑了 有时候团队赛由于个别人的自私他想刷分刷 把全队坑了

Researcher 14:59:40
I see. At this time, do you feel that the difficulty or challenge of the game has become higher?
原来如此。那这个时候您会感觉游戏的难度或者挑战变大了吗？

Informant 009 15:00:06

Yes. (I) felt that the challenge and difficulty was higher.
对的  觉的挑战难度变高了

Researcher 15:00:26
I see. If you have the anxious experience in the game, what do you do to ease your anxiety?
原来如此。如果您在游戏中有焦虑的经历，您会怎么做去缓解焦虑?

Informant 009 15:01:07
Make myself stronger or get Angry and go offline.
把自己稍微再变的强点  或者气的下游戏了

Researcher 15:01:54
Ok. Let's talk about these two ways to alleviate anxiety.
恩恩。我们分别来谈谈这两种缓解焦虑的方式。

Informant 009 15:02:24
OK.
好

Researcher 15:02:30
Firstly. Make myself stronger.
首先是第一点。把自己稍微再变的强点。

Researcher 15:02:45
Through which channels would you choose to make yourself stronger?
您会选择通过哪些渠道让自己变得强呢？

Informant 009 15:02:58
Recharge money and improve my attacking power.
充钱  提高自己的攻击力

Researcher 15:03:06
Is this the only one?
只有这一种吗？

Informant 009 15:04:08
Yes.
嗯嗯

Informant 009 15:04:16
The attacking power can only be improved through this way.
只能这样提高攻击力

Informant 009 15:04:36
Or, take part in activities and get other rewards.
要么多参加活动 得到其他奖励

Researcher 15:05:02
I see. Ok, which types of in-game goods would you buy when you need to them(Power-ups, Expansion packages, Playable characters, Cosmetics/Skins, Loot boxes, Time-savers)?
原来如此。好的，在需要购买游戏内商品的情况下，您会购买哪些类型的游戏内商品呢？（增强道具，扩展包，可游玩的角色，装饰/皮肤，抽奖箱，省时道具）？

Informant 009 15:06:23
Power-ups and Loot boxes.
增强道具 抽奖箱

Researcher 15:06:50
Ok. In our first interview, we talked about the fact that farming merchants feel that they are not capable enough to participate in certain large-scale activities in the game.
好的。我们在第一次访谈中谈到，种田商人会觉得自己觉得能力不够不参与某些游戏内的大型活动。

Informant 009 15:07:15
Yes.
嗯嗯

Researcher 15:07:26
Does the "capability" here mean the player's own gaming skill or the ability that the game mechanism endows to the player?
请问这边的"能力"指的是玩家自身的游戏水平还是游戏机制赋予玩家的能力？

Informant 009 15:07:50
The ability that the game mechanism endows to the player
游戏机制赋予玩家的能力

Informant 009 15:08:00
It's mainly depends on if (the player) has recharged enough money.
只要还是看钱充的多不多

Researcher 15:08:43
I see. This is to say, when you are playing Luanshiwangzhe, if you don't recharge, the game mechanism would limit your ability to be improved, right?
原来如此，就是说，您在玩乱世王者的时候，如果不充钱，游戏里会有机制去限制您能力的提高，是吗？

Informant 009 15:09:07
Yes.
对的

Informant 009 15:09:26
Nothing is gained without recharging.
不充钱什么都没
Researcher 15:09:38
Ok. Similarly, we talked about “farming merchants” during our first interview, and you shared your experience when being a farming merchant: “I didn't want to pay for it at the beginning. My friend said that this game can make money and made me a merchant. I really didn't recharge for the first few months. However, you are a merchant and you are about to be bullied. While you are doing the collection, people are hitting you. You are not able to beat others, and you can only suffer. Later, we had a conflict with other league, and our entire league of farmers began to cultivate military commanders in order to flight for the vindication.”
好的。同样地。我们在第一次访谈的时候谈到“种田商人”。并且您分享了一些当种田商人的经历：“刚开始的时候没想充钱，我朋友说这个游戏能赚钱、让我当商人。前几个月我也真没怎么充。可你做商人要被欺负，你在采集，人家撞你，你还打不过人家，只能吃亏。后来跟人家盟杠上了，我们整个种田盟都开始培养武将了。为了打架争口气”

Researcher 15:09:43
Did you have an anxious experience when you were a farmer merchant?
请问您在当种田商人的时候，有没有焦虑的体验？

Informant 009 15:10:46
Yes, I did. Because some people like hitting farming. When you are farming well, they just hit you.
也有 因为有人喜欢撞田 你好好的在那采集 人家把你撞了

Researcher 15:11:04
What’s the meaning of “hitting farming”?
请问“撞田”的意思是？

Informant 009 15:13:11
That is, I am collecting gold coins, and then people beat me. I could have collected 10 gold coins, but after being beaten I could only get 1 coin. If he/she has the plundering skill, they can make what I have collected their own.
. I was struck by white and I could only get 1 gold coin. If he has the skills, he can plunder.
就是我在采集金币 然后人家把我采集的给撞了 我本来可以采集 10 个金币 被撞了就白辛苦了 只能拿到 1 个金币 他要是有技能还能掠夺 把我采集的占为己有

Researcher 15:14:03
I understand. How can you alleviate your anxiety in this situation?
我明白了。在这种情况下您会怎么缓解自己的焦虑感呢？

Informant 009 15:14:39
Endure. Actually, I really want to curse. I feel anxious.
忍着 心里其实很想骂人 觉得烦躁
Informant 009 15:14:59
Many people would curse to each other, but it doesn’t work.
很多人会对骂 但骂了没用

Researcher 15:15:28
Ok. Last time you also mentioned “I didn't want to pay for it at the beginning. My friend said that this game can make money and made me a merchant. I really didn't recharge for the first few months. However, you are a merchant and you are about to be bullied. While you are doing the collection, people are hitting you. You are not able to beat others, and you can only suffer. Later, we had a conflict with other league, and our entire league of farmers began to cultivate military commanders in order to flight for the vindication”
我明白了。您上次还提到“刚开始的时候没想充钱，我朋友说这个游戏能赚钱、让我当商人。前几个月我也真没怎么充。可你做商人要被欺负，你在采集，人家撞你，你还打不过人家，只能吃亏。后来跟人家盟杠上了，我们整个种田盟都开始培养武将了。为了打架争口气”

Researcher 15:15:42
Does the process of training military commanders cost money?
请问培养武将这个过程是不是要花钱？

Informant 009 15:16:00
Yes.
对的

Informant 009 15:16:17
Good military commanders are orange.
好的武将都是橙色的

Informant 009 15:16:22
Buying orange pieces are required.
要买橙色碎片

Informant 009 15:16:29
They are only available through recharging.
充值才有

Informant 009 15:16:35
They are not available without recharging.
不充没有

Researcher 15:16:38
To which category of in-game goods does military commanders belong: Power-ups, Expansion packages, Playable characters, Cosmetics/Skins, Loot boxes, Time-savers?
武将是属于 增强道具，扩展包，可游玩的角色，装饰/皮肤，抽奖箱，省时道具中的哪一类游戏内商品呢？

Informant 009 15:16:55
In the early stage, the activities didn't reward the pieces, and you could only buy them.
前期基本活动也没 没碎片送的 只能买买买

Informant 009 15:17:12
Power-ups
增强道具

Informant 009 15:17:45
And (they are) rewards of accumulative purchasing.
还有就是累充赠送

Researcher 15:18:42
I understand. We just mentioned your attempt to relieve anxiety through cursing people. However, does it work?
我了解了。我们刚才还提到了您试图通过骂人来缓解焦虑的情绪。但这种方式有用吗？

Informant 009 15:19:34
It's useless. At first, I would (curse people). Later I endure, or when I am too anxious, I hit him back.
没用 一开始还会 后来就自己忍 要么实在太烦 撞回去 或者打他城

Researcher 15:20:08
原来如此。自己忍着的时候心里依然很焦虑吗？

Informant 009 15:20:26
Uh, I felt so annoyed.
嗯嗯 觉得好烦躁

Informant 009 15:20:29
I was angry.
很生气

Researcher 15:20:34
I understand. We just mentioned another way to alleviate anxiety, that is, "go offline"
我明白了。我们刚才还提到一种缓解焦虑的方式，就是“或者气的下游戏了”

Informant 009 15:20:46
Yes.
对的

Researcher 15:20:48
Do you think this way can alleviate your anxiety?
您觉得这种方式能缓解您的焦虑吗？

Informant 009 15:21:05
It works. I cannot be anxious without looking at it.
也能 不看着就不烦心了

Informant 009 15:21:11
Do something else.
做点其他事情

Researcher 15:21:34
Ok. Does the "being angry and go offline" here means temporary give up the game?
好的。请问这里的“气得下游戏了”指的是暂时放弃游戏的意思吗？

Informant 009 15:21:42
Initially, I would "fight" directly to hit his/her castle.
最初还会“打架”直接打他城堡

Informant 009 15:21:54
Yes, (I) temporarily don’t loot at that.
嗯嗯 暂时不看了

Researcher 15:22:39
Ok. Have you ever had the experience which directly annoyed you and made you give up permanently?
好的。您有没有过一款游戏直接让您很焦虑，随后想永久放弃的经历？

Informant 009 15:22:50
Yes.
有

Researcher 15:23:07
Can you describe the experience at that time?

可以稍微描述一下那时候的经历吗？

Informant 009 15:23:13
Wangzherongyao. My skill is not as good as other people. I was keep losing.
王者农药 技术不如人 打一盘输一盘

Informant 009 15:23:33
I was enough weak. (But my) teammates were weaker than me.
自己够菜了 队友比自己还菜

Informant 009 15:23:39
There was no way to fight
完全没法打

Researcher 15:25:03
I see. How do you define the difference between giving up a game temporarily and giving up a game permanently? When is it for you to give up a game temporarily, when is it for you to give up a game permanently?
原来如此。您如何定义暂时放弃一款游戏和永久放弃一款游戏的差异呢？什么时候对您来说是暂时放弃一款游戏，什么时候算是永久放弃一款游戏呢？

Informant 009 15:25:53
If I give up a game permanently, I would uninstall it and wouldn't touch it again.
我永久放弃一款游戏 就卸载了 不会再去碰了

Informant 009 15:26:25
Regarding to give up a game temporarily, I don't uninstall. After resting for a moment, I will back to play.
暂时的话 会不卸载 缓过来了 又去玩了

Researcher 15:26:32
So, the behaviour of "uninstall" is very significant to separate these two concepts, right?
原来如此，就是"卸载"这个行为是最显著界定这两个概念的，是吗？

Informant 009 15:26:42
Yes.
对的

Informant 009 15:27:18
Sometimes, there are fewer and fewer friends playing a game, and I don't want to play as well.
有时候一个游戏 玩的朋友越来越少了 自己也会不想玩了

Researcher 15:28:05

Ok. We just talk about how you alleviate the anxiety using a variety of methods, including purchasing in-game goods (Power-ups and Loot boxes) to alleviate the anxiety.
好的。我们刚才谈到了您通过多种方式缓解焦虑。其中就包括了购买游戏内商品（增强道具 抽奖箱）来缓解焦虑。

Researcher 15:28:22
Do you think the in-game goods help you to enter the flow state?
您认为游戏内商品可以帮助您进入心流状态吗？

Informant 009 15:30:08
What’s flow state?
什么叫心流状态呀

Researcher 15:30:36
It is the state of the flow experience as we have mentioned at the beginning. “Flow experience” has been used by psychologist to describe a state of mind experienced by people who are deeply involved in an activity. Instance, sometimes while playing videogames, the player’s action and awareness are merged, and he/she is totally connected on the gaming tasks at hand. In this state, the player loses his/her consciousness, and his/her perception of time becomes faster or slower than usual. Also, the player perceives a feeling of being in control, which empowers him/her from the fear of failure.
就是我们一开始就提到的心流体验的状态。心理学家使用“心流体验”来描述深度参与某项活动的人所经历的心理状态。 例如，有时玩家在玩电子游戏时，他/她的动作和意识会融为一体，并且他/她完全关注手头的游戏任务。 在这种状态下，玩家失去他/她的自我意识，他/她对时间的感知变得比平时更快或更慢。 此外，玩家会感受到一种掌控全局的感觉，这使他/她免于对失败的恐惧。

Informant 009 15:31:33
Yes.
有吧

Researcher 15:31:57
Yes. Once after having the flow experience, will you come back to the game to regain this experience?
好的。请问，一旦获得过心流体验，您会想回到游戏中为了重新获得这种体验吗？

Informant 009 15:32:07
When playing the game seriously, I can't listen to what others are telling me.
玩游戏认真的时候 都听不进去别人和你讲什么

Informant 009 15:32:31
If (I) give up temporarily, (I) will. If (I) give up permanently, (I) will not.
暂时放弃会 永久的不会

Researcher 15:32:42
I see.
原来如此。

Informant 009 15:33:11
Respect to the permanent case, (it means that I) am not interested in this game, and (I) will not.
永久的 对这个游戏没兴趣了 就不会有了

Researcher 15:33:27
I understand. Let’s move to the next topic.
我懂了。我们进入下一个话题。

Researcher 15:33:30
Have you had a boring experience during the game?
您在游戏时有过无聊的经历吗？

Informant 009 15:33:34
Yes.
有

Researcher 15:33:42
Under what circumstances do you usually have?
通常在什么情况下有？

Informant 009 15:33:45
After playing a videogame for a long time, it will be boring.
一个游戏玩久了 是会无聊的

Informant 009 15:34:00
I have been playing Luanshiwangzhe for two years.
我这个乱世王者玩了 2 年了

Researcher 15:34:11
Yes, please continue.
恩恩，您说

Researcher 15:34:44
Please describe some details of the game that gradually makes you bored.
麻烦请描述一下一款游戏逐渐让您觉得无聊的种种细节

Informant 009 15:34:51
For example, there are fewer and fewer people playing in one server, and it no longer

makes sense. Or (I) have upgraded to the highest level, and (I) feel like there is nothing to do.
比方说这个区玩的人越来越少 感觉没什么意义了 要么升级升到最高了 感觉没什么好做的了

Informant 009 15:35:16
It will be boring without new activities.
不出新的活动 就会无聊

Informant 009 15:35:27
Or if the activities are unchanged, it's boring.
要么活动一成不变也很无聊

Informant 009 15:35:41
Playing mechanically causes numbness.
机械式的玩 会麻木

Researcher 15:36:06
At this time, if there is new update of contents, but you need to pay for them (forms like Extension packages or new Playable characters). Would you choose to buy?
在这个时候，如果有新内容更新，但是需要花钱购买的形式（扩展包或者新的可游玩角色）。您会选择购买吗？

Informant 009 15:36:15
Yes, I would.
会啊

Informant 009 15:36:44
If I don't buy them, nothing leaves to be played.
不买 更没的玩了

Researcher 15:37:03
Ok. Generally, how do you choose to ease the boring experience? In addition to buy Expansion packages and Playable characters, as what we just said,
好的。一般您会选择怎么缓解无聊的体验？除了我们刚才说的购买扩展包或者新的可游玩角色

Informant 009 15:37:35
There is tired period.
有厌倦期的

Researcher 15:38:02
Emmm... What stages does the tired period generally include?
Emmm...请问厌倦期一般包含了哪些阶段？

Informant 009 15:38:10
For example, like recently, (I) am busyat work. I just go online to check, collect vegetable, and complete the tasks.
就好比最近上班忙 我就上去看看 收收菜 完成下任务

Informant 009 15:38:38
Sometimes I am boring, so I chat with people in the league.
有时候无聊 就跟盟里的人聊聊天

Informant 009 15:39:07
Or there are too many tasks, and I don't want to do them. I can't continuously doing them, I'll get bored.
要没任务太多 不想做了 做不完的做 也会厌倦

Researcher 15:39:54
I see. Does boredom leads to your temporary abandonment or permanent abandonment of the game?
原路如此。那无聊会不会导致您想暂时放弃游戏或者永久放弃游戏?

Informant 009 15:40:40
Yes. RO was really boring. In the end, there was no one chatting even in the league. Then (I) abandoned (it).
会 ro 仙境传说 是真的蛮无聊的 玩到后面盟里连聊天的人都没 就放弃了

Informant 009 15:41:41
When the role reached the highest level, and there were no new levels. The dungeon bosses were too weak to be beaten. All people were there beating them. In the end, all items were worthless.
它等级满了 没升 然后 boss 副本太好打了 人人在那围着打 最后道具都不值钱了

Informant 009 15:42:00
Then good bye.
就拜拜了

Researcher 15:42:05
At that moment, did you think the difficulty of the game was getting lower?
这个时候有觉得游戏的难度变低了吗?

Informant 009 15:42:15
Yes.
对的

Informant 009 15:42:28
In addition to “hook up”, there was hook up.
除了挂机还是挂机

Informant 009 15:42:53
When there were more people (around) the boss, the server became stuck, and there was no way to play.
boss 人一多 服务器就卡 没法玩

Informant 009 15:42:57
The experience was bad.
体验差

Researcher 15:43:07
Ok. We just mentioned there if there is update of new contents, but you need to pay for them (Extension packages and new playable characters). Would you choose to buy them?
好的。我们刚才提到。如果有新内容更新，但是需要花钱购买的形式（扩展包或者新的可游玩角色）。您会选择购买它们。

Researcher 15:43:08
Do you think these in-game goods can help you to re-enter to the flow state?
您认为这些游戏内商品可以帮助您重新进入心流状态吗？

Informant 009 15:44:04
Yes, they can. Partially they can.
能吧 小部分能

Informant 009 15:44:17
In fact, the most important (thing) is mechanism.
其实最主要的还是机制

Researcher 15:44:31
What's the reason for another part that cannot lead you to the flow state?
另外一部分不能您觉得什么原因呢？

Researcher 15:45:06
After purchasing an Expansion pack or a new Playable character, is it still boring?
是购买了扩展包或者新的可游玩角色后，依然让您处于无聊的状态还是？

Informant 009 15:45:10
For example, (if) there is not groundbreaking and there are always such activities, after reaching the highest level...
游戏没突破性 比方说永远就这么点活动 满级了

Informant 009 15:45:25
, after reaching the highest level, what can (I) do?
满级了　还能干嘛

Informant 009 15:45:31
The level cannot be enhanced.
没的升级了

Informant 009 15:45:36
It feels like saturated.
感觉饱和了

Researcher 15:45:46
There are no new challenges in the game, right?
游戏内没有新的挑战，是吗？

Informant 009 15:45:53
Yes.
对的

Researcher 15:46:23
I see.
原来如此。

Researcher 15:47:21
Have you ever had such experience: In a game which you felt boring previously, but later when you suddenly went to play it again, you didn't feel boring?
您有没有一段时间没有玩的游戏，之前觉得无聊，后来突然又去玩了一下又觉得不无聊的经历？

Informant 009 15:47:44
Yes. Wangzherongyao.
有　王者荣耀

Researcher 15:48:22
Previously you felt bored, then you didn't feel bored playing it later. Why do you think this happens?
之前是觉得无聊的，后来上去玩了又觉得不无聊，您觉得这是为什么呢？

Informant 009 15:49:05
I played with my colleagues before, and everyone gave up playing. Then I was also not interested. Later I began to play the Wangzherongyao where I met new friends, and they also played Wangzherongyao. Then we started to team up and play using the

microphone.
之前和同事一起玩 然后大家都不玩了 我也兴趣淡了 后来玩乱世王者 认识新朋友了 他们也玩荣耀 然后开始组队开黑了

Researcher 15:50:05
I see. I understand.
原来如此。我了解了。

Researcher 15:51:06
In addition, is there such circumstance: You haven't played a game for a period of time, and your gaming skill is decreased, but at this time you find the game is interesting?
另外有没有一款游戏一段时间没玩，对于一款游戏技巧生疏了，反而有觉得好玩的情况？

Informant 009 15:51:24
No.
这个没

Researcher 15:51:28
Ok.
ok 好的。

Researcher 15:51:33
Have you had any experience of supportive purchasing? I mean purchasing in-game goods for supporting the game marker than acquiring the in-game goods themselves.
您有没有过支持性购买的经历？我的意思是为了支持游戏开发商而购买游戏内商品，而不是为了获得游戏内商品本身。

Informant 009 15:51:50
No.
好像没有

Researcher 15:52:08
Ok. Is acquiring the flow experience your purpose of playing videogames?
好的。请问获得心流体验是否是您玩电子游戏的目的？

Informant 009 15:52:18
I played RO in my childhood. I recalled (the game) playing its mobile version, but I didn't make consumption.
ro 仙境传说 童年玩的 回忆一下玩这个手游 但没消费

Informant 009 15:53:07
Playing games is mainly to kill time or close the distance of the friends.
玩游戏主要还是打发下时间 或者拉近小伙伴们的距离

Researcher 15:54:16
Ok. Here, “kill time” means make yourself not bored?
好的。这边所说的打发时间是为了让自己不无聊吗？

Informant 009 15:54:31
Yes.
对的

Informant 009 15:54:34
Yes.
对的

Researcher 15:54:56
The interview is almost over. Do you have any ideas to add?
我明白了。访谈差不多要结束了。您还有什么观点需要补充吗？

Informant 009 15:55:17
No.
木有了

Researcher 15:56:31
These are all the questions. Thank you very much for participating in our research. Please confirm that your email address is XXXXXX@XXXXXX.com, because later we will send the JD electronic gift card to this address.
这就是全部的问题。 非常感谢您参与我们的研究。请确认您的电子邮件地址是XXXXXX@XXXXXX.com，因为稍后我们把京东电子礼品卡发送到这个地址。
