# Supplementary material for: A grounded theory approach to understanding in-game goods purchase
Source: PLoS One. 2022 Jan 27;17(1):e0262998. doi: 10.1371/journal.pone.0262998 (PMC8794092; doi:10.1371/journal.pone.0262998)
Supplement: S1 File — (ZIP) [file pone.0262998.s001.zip › Transcript 16.pdf]

Interview: 016

Informant: 012

Please note that the original transcript is in Simplified Chinese. The English translation is for internal communication among the author of this research, and it is not proofread. Potential linguistic errors may exist in the English translation.

Researcher 15:52:28
Thank you for your willingness to participate and be interviewed here. My name is XXX XXX, and I'm a PhD student in the XXX University of XXX(XXX). Currently, I'm working on a research project which focuses on videogame players' purchase motivations of in-game goods. Throughout this interview, I will ask you a series of questions and you are encouraged to express your opinions freely with emoticons. If I have questions about what you've said or need clarification about a topic or concept, I'll ask you.
感谢您愿意参加并在此接受采访。 我叫 XXX，我是市场营销学的博士生，现在我在 XXX 大学就读。 目前，我正在开展一个研究项目，专注于电子游戏玩家对游戏内购买项目的购买动机。 在整个访谈中，我会问您一系列问题，我们鼓励您自由表达您的意见和观点。因为这不是一个当面访谈，所以我们也鼓励您用 QQ 表情来表达您的情绪。 在访谈过程中，如果我对你所说的内容有疑问或需要您澄清一个主题或概念，我会问您。

Researcher 15:52:38
Are you ready?
您准备好了吗？

Informant 012 15:53:06
Yes.
好的

Researcher 15:53:24
In the questionnaire, I have seen in-game products that you have purchased skin type in-game goods. What are your motivations for purchasing them?
在问卷中，我有看到您购买过皮肤类的游戏内商品。请问您购买它们的动机是什么呢？

Informant 012 15:53:37
They look good.
比较好看

Researcher 15:54:30
Well, do you buy more skins in stand-alone games or buy more in online games?
嗯，您在单机游戏里买皮肤多还是在网络游戏里买得多？

Informant 012 15:54:38
In online games.
网络游戏

Researcher 15:55:19
Do you think people around you (offline or online) have an impact on your purchase of in-game goods?
您觉得您周围的人（线下或线上）对您购买游戏内商品有影响吗？

Informant 012 15:55:31
Yes.
有啊

Researcher 15:55:44
Well, we can talk more about this.
嗯嗯，我们可以多谈谈这点。

Researcher 15:56:06
Can you tell me how people around you influence your purchase of in-game goods?
可以说一下您周围的人是怎么影响您购买游戏内商品的吗？

Researcher 15:56:37
You can give a few examples.
您可以举几个例子来谈。

Informant 012 15:56:43
For example, in the League of Legends, there is a new skin, and I want to buy it became I think it looks good. However, my wife does not allow me to buy, then I am not going to buy it.
比如英雄联盟，新出了一个皮肤，我想买，我觉得好看，我老婆不给我买，那我就不买了。

Informant 012 15:57:10
Moreover, friends are the case. If (my) friends say it is not good, or that is not worth the price, I will not buy it.
还有朋友啊，朋友说不好看，或者说不值那个价格啊，我也不会买

Researcher 15:57:32
Well, let's talk one by one. First of all, what is the reason why your wife won't let you buy (the skin)?
嗯嗯，我们一个个来谈。首先，您老婆不让您买的理由一般是什么？

Informant 012 15:57:44
There is no need to talk about this.

这个就不需要谈了吧

Informant 012 15:57:56
I think this has nothing to do with what we are talking about
我觉得这个跟我们谈的没有关系

Researcher 15:58:30
Ok. If you feel that there are sensitive topics, we can choose to skip them.
好的。如果接下来有觉得比较敏感的话题，我们可以选择跳过。

Researcher 15:59:23
Let's talk about the impact your friends have on your buying process.
我们来谈一下您的朋友对您的购买过程影响的情况。

Informant 012 15:59:49
If (my) friends say it is not good, or that is not worth the price, I will not buy it.
朋友说不好看，或者不值那个价格，所以我就不买了

Researcher 16:00:04
Well, I want to know if the friends mentioned here are generally friends in the real life or friends in online games?
嗯嗯，我想了解一下这边说的朋友一般是现实中的朋友还是网络游戏中的朋友呢？

Informant 012 16:00:06
And if I don’t like very much neither…
如果我自己又不是太喜欢的话

Informant 012 16:00:15
In the real life.
现实

Researcher 16:00:53
Are these friends playing the same game with you?
这些朋友也是和您玩同样的游戏吗？

Informant 012 16:00:58
Yes.
嗯

Informant 012 16:01:02

Researcher 16:02:05
I see. Can I understand this way: Before buying a skin, you would generally mention this purchase intention to your friends, and then listen to their opinions?

原来如此。我能不能这样理解，在购买皮肤前，您一般会把这个购买意向和您的朋友说，然后听听他们的意见？

Informant 012 16:02:51
No, for example, when there is a new skin, I will ask him if he buy or not, and then talk about the skin together.
不是，比如说要出新皮肤了。我会问他买不买，然后一起聊聊皮肤好不好

Informant 012 16:03:02
用

Researcher 16:04:13
I see. I need to confirm one thing. I just saw in the questionnaire that you have only bought in-game goods of skin type in the last 6 months. Is that true?
原来如此。我稍微确认一下，刚才在问卷中我看到最近 6 个月中您只买过皮肤这一类的游戏内商品，是这样吗？

Informant 012 16:04:27
Yes.
对

Researcher 16:04:33
You haven't bought other types of goods, have you?
没有买过其它类型的商品，对吧？

Informant 012 16:04:38
No.
对

Researcher 16:05:12
Well, would you buy the same skinss as your friends around you or some different skins?
嗯嗯，您会和周围的朋友买一样的皮肤还是不一样的皮肤呢？

Informant 012 16:05:49
I play League of Legends, and there is limited number of skins in the game. There is no difference.
我是要英雄联盟的，皮肤总共就那么多，有什么一样不一样的

Researcher 16:07:04
Well, I personally haven’t played a lot the dota type of games. Isn't there a lot of skin in each character in League of Legends?
嗯嗯，我个人没有怎么玩过 dota 类的游戏。在英雄联盟里是不是每一个角色拥有的皮肤数量不是很多？

Informant 012 16:07:41
Averagely, one hero has about 5 or 6 (skins).
一个英雄平均下来大概 5，6 个

Researcher 16:09:33
Ok. We just said that buying skin is for good looks. Is it more pleasing to yourself or to others?
好的。我们刚才说到了买皮肤是为了好看，请问更多的是让自己赏心悦目呢还是让别人也能看到?

Informant 012 16:10:14
It must be to myself. I only care about looking at it comfortably, and I don't care about how others look. No matter what I do, it's not perfect for others.
肯定是自己了，自己看着舒服就行了，在乎别人怎么看，怎么做都不完美

Researcher 16:11:24
I understand. In another case, for example, if you see other players having a skin in the game, will this event affect your purchase of the skin?
我明白了。另一种情况，比如您在游戏中看到其它玩家拥有一个皮肤，这个事件会对您购买皮肤有影响吗？

Informant 012 16:11:34
No.
没有

Researcher 16:12:02
Ok. In addition to the League of Legends, what other games do you usually play?
好的。除了英雄联盟，一般您还玩什么游戏?

Informant 012 16:12:07
There is no more.
没有了

Researcher 16:12:46
Uh huh. I don't know much about the League of Legends. Is there a system like a league in this game?
嗯嗯。我不太了解英雄联盟，请问这个游戏里有没有类似工会的系统？

Informant 012 16:12:53
(No).

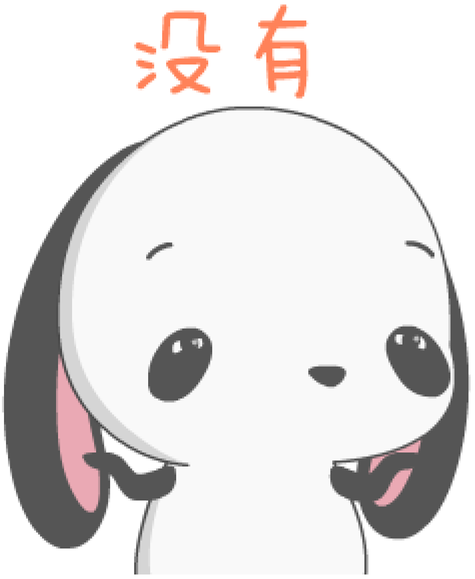

Researcher 16:13:29
Is it in the League of Legends that everyone has less chance of socialising?
是不是在英雄联盟里，大家社交的机会比较少？

Informant 012 16:13:45
No, it’s not the case.
也不是，

Informant 012 16:14:03
Do you know Wangzherongyao?
王者荣耀，你知道吗

Researcher 16:14:10
Yes, I know it.
嗯嗯我知道

Informant 012 16:14:20
The game is similar to Wangzherongyao.
跟王者差不多

Researcher 16:15:15
I only played dota maps in the age of war3. I feel that this kind of game focuses more on competition, doesn't it?
我只在 war3 年代玩过 dota 地图。感觉这类游戏更注重竞技，是吗？

Informant 012 16:15:23
Yes, it does.
对

Researcher 16:15:46
Do you usually have a lot of communication with players in the game?
一般您会和游戏内的玩家进行很多交流吗？

Informant 012 16:15:47
There are S series finals tomorrow, you can take a look.
明天有 S 系列决赛，你可以看一下，

Informant 012 16:15:52
Rarely
很少

Informant 012 16:16:16
You can use “Ni” instead of “Nin”.
你可以用你，不要您

Informant 012 16:16:21
I’m not used to it.
不习惯

Researcher 16:16:33
I almost never play this kind of game now, the operation is too bitter... I'm old.

现在几乎不玩这类游戏啦，操作台苦手了...人老了

Researcher 16:16:46
Haha, ok.
哈哈，好的。

Informant 012 16:16:46

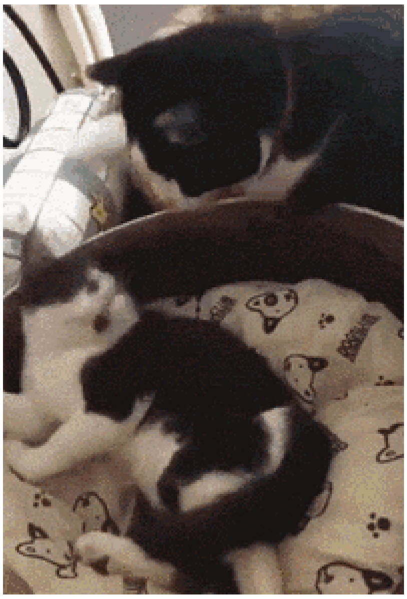

Researcher 16:18:05
Let's go back to the question just now, that is, you have very little communication with other players in the League of Legends, and then they don't affect your purchase decision to buy skins. Is that true?
我们回到刚才的问题，也就是说，您和英雄联盟里的其它玩家交流很少，然后他们也不影响您购买皮肤的购买决策，是这样吗？

Informant 012 16:18:15
Yes.
对

Researcher 16:18:36
Then it’s family and friends who have more influence on your buying decisions, right?
然后对您购买决策影响更多的是家人和朋友，对吧？

Informant 012 16:18:49
Yes.
嗯

Researcher 16:19:10
Well, how many friends are we talking about here?
嗯嗯，这边说的朋友是多少个人？

Informant 012 16:19:18
A few.
几个

Researcher 16:19:39
A few?
个位数的朋友？

Informant 012 16:19:44
Yes.
对

Researcher 16:21:38
Ok. In general, under what circumstance does the dialogue of skins happen between you and your friends? Online or offline?
好的。一般你和你的朋友在聊游戏内的皮肤这个对话是在什么场景下发生的？我指的是线上还是线下？

Informant 012 16:22:10
Online.
线上

Informant 012 16:22:45
I'm not with my friends in the same place.
跟朋友都不在一个地方

Researcher 16:23:30
Ok. Do you communicate using the in-game chat or through other social media tools (QQ, WeChat)?
好的，一般是在游戏内聊天还是通过其它的社交媒体工具(QQ,微信)呢?

Informant 012 16:23:45
In side the game.
游戏内

Researcher 16:24:12
Ok, I want to know if these friends know each other?
好的，我想知道这些朋友之间都相互认识吗？

Informant 012 16:24:30
Yes, the friends in the real life.
对，现实中的朋友

Researcher 16:26:05
Ok. When talking about these skins, do you communicate through group chatting or through private chatting?

原来如此，一般在谈论这些皮肤的时候，是通过大家一起群聊还是通过密聊来沟通呢？

Informant 012 16:26:57
We just mention this topic while playing the game, instead of talking about this intentionally.
一般不会因为这是讨论，就是打游戏聊天的时候说起来，顺便说一下

Informant 012 16:27:10
I can tell you like this.
我这么给你说吧

Researcher 16:27:20
Yes, please.
嗯嗯，你说。

Informant 012 16:27:54
Buying skins can't affect me a lot. I can buy them or not. I don't discuss about this sort of small things intentionally.
买皮肤这事，影响不了我什么，可以买，也可以不买，不会因为这种小事专门讨论之类的事

Informant 012 16:28:01
Do you understand?
我这么说你可以明白嘛

Researcher 16:28:41
Yes, I do.
嗯恩，我了解了。

Informant 012 16:28:50

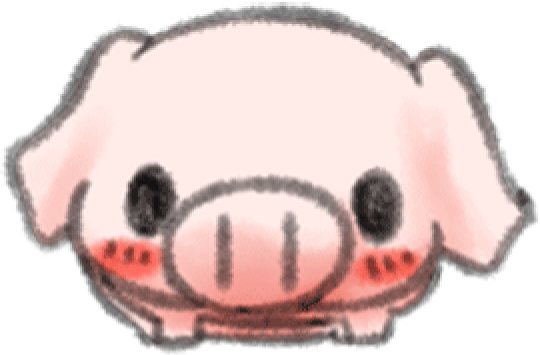

Informant 012 16:29:43
The game is just a game, and it can't affect my life. I play it when I have nothing to do.
游戏只是个游戏，影响不了我的生活，有空没事的时候玩一玩

Researcher 16:30:13
Well, I understand. In addition, the experience of friends around you is generally richer than you or your game experience is more abundant than them?
嗯嗯，了解了。另外，你身边的朋友游戏经验一般比你丰富还是你的游戏经验比他们丰富？

Informant 012 16:30:35
Almost the same.
都差不多

Researcher 16:30:35
Or everyone's experience in playing games is similar, and no one is a very skilled in games.
还是大家玩游戏的经验都差不多，没有哪一个是特别高玩的朋友

Researcher 16:30:45
Ok, I see.
嗯嗯，好的。

Researcher 16:31:46
Ok, the interview is almost over. Do you have any ideas to add?
OK。访谈差不多要结束了。您还有什么观点需要补充吗？

Informant 012 16:31:56
No.
没有

Informant 012 16:32:00
(No)

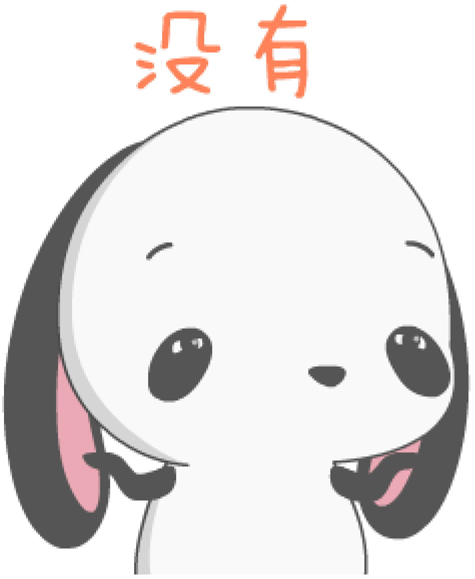

Researcher 16:32:14
These are all the questions. Thank you very much for participating in our research. Please confirm that your email address is XXXXXX@XXXXXX.com, because later we will send the JD electronic gift card to this address.
这就是全部的问题。 非常感谢您参与我们的研究。请确认您的电子邮件地址是XXXXXX@XXXXXX.com，因为稍后我们把京东电子礼品卡发送到这个地址。
