# Supplementary material for: A grounded theory approach to understanding in-game goods purchase
Source: PLoS One. 2022 Jan 27;17(1):e0262998. doi: 10.1371/journal.pone.0262998 (PMC8794092; doi:10.1371/journal.pone.0262998)
Supplement: S1 File — (ZIP) [file pone.0262998.s001.zip › Transcript 17.pdf]

Interview: 017

Informant: 010

Please note that the original transcript is in Simplified Chinese. The English translation is for internal communication among the author of this research, and it is not proofread. Potential linguistic errors may exist in the English translation.

Researcher 17:06:35
Thank you for your willingness to participate and be interviewed here. My name is XXX XXX, and I'm a PhD student in the XXX University of XXX(XXX). Currently, I'm working on a research project which focuses on videogame players' purchase motivations of in-game goods. Throughout this interview, I will ask you a series of questions and you are encouraged to express your opinions freely with emoticons. If I have questions about what you've said or need clarification about a topic or concept, I'll ask you.
感谢您愿意参加并在此接受采访。 我叫 XXX，我是市场营销学的博士生，现在我在 XXX 大学就读。 目前，我正在开展一个研究项目，专注于电子游戏玩家对游戏内购买项目的购买动机。 在整个访谈中，我会问您一系列问题，我们鼓励您自由表达您的意见和观点。因为这不是一个当面访谈，所以我们也鼓励您用 QQ 表情来表达您的情绪。 在访谈过程中，如果我对你所说的内容有疑问或需要您澄清一个主题或概念，我会问您。

Researcher 17:06:40
Are you ready?
您准备好了吗？

Informant 010 17:08:15

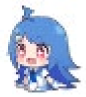

Researcher 17:08:22
Ok. In the last interview we mentioned the concept of "Flow experience". Let's review this concept:
好的。我们上一次访谈的时候提到了”心流体验“这个概念。我们来回顾一下这个概念：

Researcher 17:08:29
"Flow experience" has been used by psychologist to describe a state of mind experienced by people who are deeply involved in an activity. Instance, sometimes while playing videogames, the player's action and awareness are merged, and he/she is totally connected on the gaming tasks at hand. In this state, the player loses his/her consciousness, and his/her perception of time becomes faster or slower than usual.

Also, the player perceives a feeling of being in control, which empowers him/her from the fear of failure.
“心理学家使用“心流体验”来描述深度参与某项活动的人所经历的心理状态。 例如，有时玩家在玩电子游戏时，他/她的动作和意识会融为一体，并且他/她完全关注手头的游戏任务。 在这种状态下，玩家失去他/她的自我意识，他/她对时间的感知变得比平时更快或更慢。 此外，玩家会感受到一种掌控全局的感觉，这使他/她免于对失败的恐惧。”

Researcher 17:08:37
In the last interview, you mentioned: “When I am playing the videogame, I actually don't know what's happening in the outside world.”,” Sometimes I would unconsciously answer my friends’ words.”,” But I forget all while playing.”
您上次有谈到“是的，我竟然玩游戏玩的不知道外界发生了什么”，“有时候会无意识回答朋友的话”，“但是玩好游戏忘记了”，“玩游戏的时候非常容易抛开现实中的一些事情”

Researcher 17:08:45
After acquiring the flow experience, how does this experience end? Which events would cause the termination of this experience?
请问您在获得心流体验之后，这个体验一般是怎么结束的？哪些事件导致了这种体验的终止？

Informant 010 17:09:11
It ends after I stop the gaming.
在我停止游戏之后停止

Informant 010 17:09:21
Calling from family members.
家人的呼唤

Informant 010 17:09:26
Or there is an important telephone call.
或许有重要的电话

Researcher 17:09:40
I see. Under what circumstances will you stop the game?
原来如此。一般在什么情况下会停止游戏？

Informant 010 17:10:09
For example, if there is a call from an important customer, I’m required to answer the customer's questions professionally.
比如有重要客户的电话，需要专业性的回答客户的问题

Informant 010 17:10:24
Or family members call me to go out and eat.
或者家人喊出门或者就餐

Informant 010 17:10:35
Or maybe I just get tried.
也可能是累了

Researcher 17:10:36
In addition to the objective reasons you just said to stop the game, will there be a situation that you subjectively want to stop the game?
除了您刚才说的客观的原因停止游戏，会不会有自己想停止游戏的情况？

Informant 010 17:10:46
It should be that I'm tried.
那就累了吧

Researcher 17:10:46
Emm... tried.
嗯嗯，累了

Researcher 17:10:52
Ok.
好的，ok。

Informant 010 17:11:04
Sometimes it will be uncomfortable after playing for a long time.
有时候玩的太久眼睛会不舒服

Researcher 17:11:16
I see. Ok.
原来如此。好的。

Researcher 17:11:17
We talked about the "anxious experience" last time: "Otherwise, some (people) cannot go on and stop in the middle way.","When being stuck in a stage for too long time, I would like to abandon the play.","I can be very annoyed when I cannot continue, not happy."
我们上次谈到了"焦虑的体验": "不然有的打了一半就玩不下去了", "有时候关卡卡太久，就会不想玩了", "打不下去的时候就会很烦躁，不开心"

Informant 010 17:11:19
Or it's too late

或者实在太晚了

Researcher 17:11:22
Do you think that the end of the flow experience is related to the anxious experience?
您认为心流体验的终止和焦虑的体验有关系吗？

Informant 010 17:11:45
Yes, there is relationship.
有关系

Researcher 17:11:57
Can you tell me more about this?
您能就这个再说点细节吗？

Informant 010 17:12:04
Because it is one of the factors that stop me from playing games.
因为他会成为我停止游戏的一个因素之一

Researcher 17:12:38
I see. When you feel anxious, the flow experience is therefore terminated, and at this time you want to end the game?
原来如此。是不是当您感觉到焦虑的时候，心流体验也就终止了，并且在这个时候就想结束游戏了？

Informant 010 17:12:49
The flow experience is good and bad. This is bad case, which makes me feel annoyed.
心流体验有好的有坏的。这种就属于坏的，会让心情烦躁

Informant 010 17:12:53
and I just don’t want to play.
就不想玩了

Researcher 17:13:10
I see. Moreover, We talked about the "boring experience" last time: “It doesn't make sense to play anymore. There are a lot of games nowadays, and many of them are similar. They let you to buy this or to buy that. You only spend a few minutes playing before they let you to the money for opening the stages.”, “Mr Love: Queen's Choice… It's just like reading a novel book.”, “There is also Star Dream
. Every day doing the repetitive things”
我明白了。另外，我们上次谈到了“无聊的体验”："感觉玩下去没意义了。现在很多游戏吧。雷同很多，还动不动就让你买这个买那个，你才玩了几分钟就让花钱开启关卡","很直接的就是你充几块钱买个道具才让你下去","恋于制作人吧...感觉就是在看小说书", "还有一个明星志愿也是。每天都是重复的事情"

Researcher 17:13:15
Do you think that the end of the flow experience is related to the boring experience?

您认为心流体验的终止和无聊的体验有关系吗？

Informant 010 17:14:39
Yes.
有

Researcher 17:14:56
Could you share more details?
可以再分享些细节吗？

Informant 010 17:14:59
I think that the mood is very important in gameplay.
我觉得玩游戏心情很重要

Researcher 17:15:06
Ok, please continue.
嗯嗯，您说

Informant 010 17:15:09
If (I) don't feel happy when playing, why should I play?
如果玩的不开心，为什么要玩呢

Informant 010 17:15:24
The previous mentioned things make me feel bad.
刚刚说的那些都让我心情会不好

Informant 010 17:15:31
Then, there is no need to continue.
那就没有继续下去的必要

Informant 010 17:16:07
A game without new ideas can make people feel boring. Then there is no need to play.
一个游戏没有新意，也会让人觉得无趣。那就也没必要玩

Researcher 17:17:13
I understand. Summarising our previous conversation, I divide it into four situations.
1. Objective forces make you to stop the game, so the flow experience is forced to be terminated.

2. Getting tired, and you take the initiative to stop the game. The flow experience is terminated.
3. Feeling anxious, the flow experience is terminated.
4. Feeling bored, the flow experience is terminated.
Is there anything else that you need to add?
我明白了。总结下我们刚才的谈话，分成 4 种情况。
1. 客观不可抗力让您停止游戏，所以心流体验被迫被终止。
2. 主观上累了，主动停下游戏，心流体验终止。
3. 感觉焦虑，心流体验终止。
4. 感觉无聊，心流体验终止。
请问还有什么需要补充的吗？

Informant 010 17:18:57
(No)

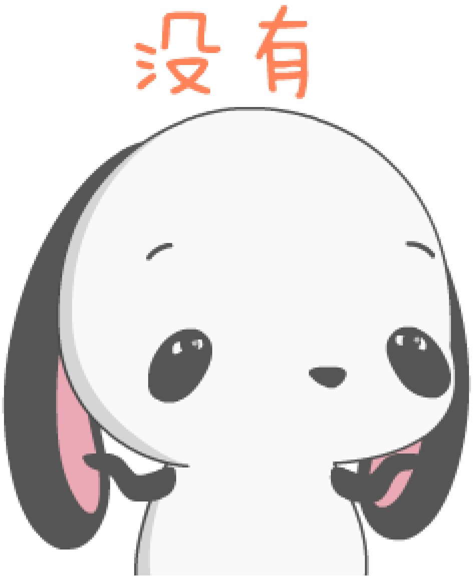

Researcher 17:19:16
Ok. Let's talk about the skin type in-game goods. You said in the first interview "Emmm, in fact, sometimes buying a skin for the character is also to show off.
", "Because it looks so much better than without the skin."
好的。我们现在来谈一下皮肤类型的游戏内购。您在第一次访谈的时候说到"emmm，其实有时候买角色皮肤啥也是为了装逼吧"，"因为看起来很就比没皮肤的厉害"

Researcher 17:19:24
Do you think people around you (offline or online) have an impact on your purchase of in-game goods?
您认为您周围的人（线下或线上）对您购买游戏内商品有影响吗？

Informant 010 17:19:51
There is influence.
有影响

Researcher 17:20:03
How do people around you influence your purchase of in-game goods? Can you give me some examples?
请问您周围的人如何影响您购买游戏内商品？ 您能给我一些例子吗？

Informant 010 17:20:34
n the game, I want to have sister skins with my friendly sisters. Moreover, there are couple skins with my own cp.
游戏里的话，会想和友好的姐妹有姐妹皮肤。也会有和自己的 cp 有情侣皮肤

Researcher 17:21:09
Ah, it is. You want to have the same skin as the person who is intimate with you, is that the case?
啊，原来如此。是想和自己亲近的人拥有一样的皮肤，是这样吗？

Informant 010 17:21:22
Before my cp wanted to send a skin that fitted my arms, but I didn’t want it. As he insisted in giving me that, I bought it later.
之前我 cp 要送一个符合我兵种的皮肤给我，我不愿意要，但是他就是要给。所有后面我自己买了

Informant 010 17:21:45
I feel that it’s only a game, and there is no need to receive money from others.
感觉游戏的而已，没必要收别人的钱财

Informant 010 17:22:13
I would have more similar skins with the person who is intimate with me.
和自己亲近的人一样的皮肤会多一些

Researcher 17:22:24
So, the "cp" here refers to a game mechanism, or is it a real interpersonal relationship?
原来如此，这边的“cp”指的是一种游戏机制呢，还是现实中的人际关系？

Informant 010 17:23:10
A game mechanism.
游戏机制

Informant 010 17:23:19
The couple in the game.
游戏的情侣

Researcher 17:23:23
Ok
ok 好的。

Informant 010 17:23:31
There are some tasks for couples.
会有一些情缘任务

Informant 010 17:23:49
In terms of the sisters, it refers to my intimate sisters in the game.
姐妹的话就是玩的很要好的很要好的

Researcher 17:24:12

I see. You said before that you have joined the league inside the game. Do the members of the league have an influence on your purchase of in-game goods?
原来如此。您之前说过您有加入游戏内的联盟，请问联盟内的成员对您购买游戏内商品有影响吗？

Informant 010 17:24:51
Sometimes the members in the league would encourage me to (buy).
有时候盟友会怂恿啊

Informant 010 17:25:08
For example, they said that this skin looked good and they liked it.
比如我说这皮肤好好看，好喜欢

Informant 010 17:25:13
They encouraged me like this.
他们也就怂恿你

Informant 010 17:25:26
I intrinsically liked the item, while there were people who encouraged me. I didn’t have enough strength.
本来就喜欢，还有人怂恿，定力不够

Informant 010 17:25:29
Then I bought it.
就买了

Researcher 17:26:13
Ah, I see. Under what circumstance does the "encouragement" type of dialogue happen in general?
啊，原来是这样。那像这样子的“怂恿”类型的对话一般发生在什么场景下？

Informant 010 17:27:01
We occasionally talk about this in WeChat group.
微信群聊天突然聊到吧

Informant 010 17:27:09
Sometimes it’s very casual chat.
有时候就是很随意的聊天

Informant 010 17:27:12
and we just move to that point.
聊到了那个点

Researcher 17:27:46
Oh, I see. The members in the WeChat group affiliate to the same game, right?
啊原来如此。微信群里都是同一个游戏的玩家，对吧？

Informant 010 17:28:59
Yes.
是的

Informant 010 17:29:28
We have many groups. Alliance management group. Alliance membership. And the whispers of our sisters.
我们有很多群。联盟管理群。联盟成员群。还有我们几个妹子的悄悄话群

Researcher 17:30:04
Ok Generally, this kind of conversations happens in a larger group or a smaller group?
好的，一般这样子的对话发生在更大的群里还是更小的群里？

Informant 010 17:30:20
It happens more in the administration group.
管理群多些

Researcher 17:30:50
Are there more people in the administration group or in the private chat group?
管理群人数更多还是悄悄话群人数更多呢?

Informant 010 17:31:17
There are more people in the administration group.

管理群多

Researcher 17:32:00
Ok. I want to confirm one thing. We just talked about "Want to be the same as the intimate people" and "Purchase after being encouraged by others" They just refer to the purchase of skin type in-game goods? Or they apply to other types of in-game goods as well? (Power-up items, Expansion packages, Playable characters, Loot boxes, Time-savers)
好的。我想确认一个事情，请问我们刚才谈到的"想要和亲近的人一样"以及"在别人怂恿下购买"只是针对皮肤类游戏内购呢？还是对其它类型的游戏内购也一样？(增强道具 扩展包 可游玩的角色 抽奖箱 省时道具)

Informant 010 17:32:21
All of them.
都有

Informant 010 17:32:46
Some of us are very evil. They win the item (that they desire), and tell that the reward is really good.
我们会有人特别坏，自己抽了道具，说奖励好

Informant 010 17:32:55
And they encourage us to participate in the lottery.
然后喊我们也去抽

Researcher 17:33:20
Will they show some good reward in the group?
他会在群里 po 那种很好的奖励出来吗？

Informant 010 17:33:37
Yes!
对！

Informant 010 17:33:46
When I draw, I got some rubbish!
然后我自己去抽！都是垃圾！

Researcher 17:33:48
And everybody was around the corner?
然后大家也蠢蠢欲动了？

Informant 010 17:33:54
(Yes)

嗯嗯~我明白了。一般像这样子的玩家总是他一个人在影响其他人还是大家都在

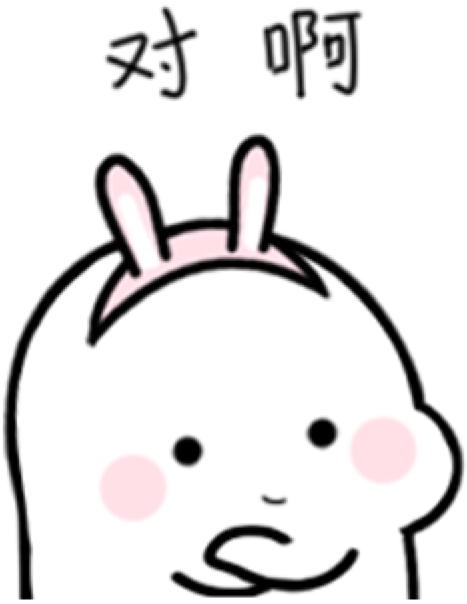

Informant 010 17:34:06
The skin is the case as well, someone said that he won the item using 10,000 Yunabao.
皮肤也是，他说他 1 万元宝抽的。

Informant 010 17:34:11
Then we were moved mentally.
然后我们就心动了

Informant 010 17:34:18
I spent 20,000 Yuanbao and gained nothing.
抽了 2 万都没

Informant 010 17:34:40
Then I needed one coupon to exchange one, and I moved on!
然后差一点券可以换一个。就继续抽了！

Informant 010 17:34:54
We all scolded him. “Are you a capper”
我们都会骂他，你是拖么

Researcher 17:34:58
Hmm~ I understand. Are players like him always affecting other people or everyone is affecting each other?
嗯嗯~我明白了。一般像这样子的玩家总是他一个人在影响其他人还是大家都在互相影响？
Informant 010 17:35:36
Everybody is affecting each other.

互相

Researcher 17:36:06
Ok. We continue. Have you purchased skins in stand-alone games?
ok。我们继续。您是在单机游戏中购买过皮肤吗？

Researcher 17:36:18
Have you purchased skins in stand-alone games?
*您在单机游戏中购买过皮肤吗？

Informant 010 17:36:23
I rarely play stand-alone games.
单机游戏玩的很少

Researcher 17:36:40
Have you purchased before in stand-alone game?
有过这样子的购买经历吗？

Informant 010 17:36:43
I play more mobile games.
一般手游比较多

Informant 010 17:36:58
Does Monopoly count?
大富翁算么

Informant 010 17:36:59
hahaha.
哈哈哈

Informant 010 17:37:13
The IPad version Monopoly. I purchased all the maps.
ipad 的大富翁。我买了全套地图

Researcher 17:37:20
Humm... I refer to the games for single person, and in this type of games, no other players play with you.
嗯嗯，我的意思是自己一个人玩的那种游戏，游戏里没有其它的玩家和你一起玩的那种

Informant 010 17:37:37
I bought the maps and characters in the Monopoly.
买过大富翁的地图和人物角色

Researcher 17:37:58
The function of the maps is to unlock new content? Or they remain the same content, and just change the texture?
地图的作用是开启新内容呢？还是和原来一样的内容，只不过换了一个贴图？

Informant 010 17:38:29
New.
新的

Informant 010 17:38:51
Maps.
地图

Informant 010 17:38:55
The gameplay is the same.
玩法是一样的

Informant 010 17:39:05
There are more maps.
就是多了地图

Informant 010 17:39:24
It should be what you said “texture”.
应该就是你说的换了贴图

Researcher 17:39:58
It turned out to be the case. So, do you think there is a difference between buying skins in stand-alone games and buying skins in online games?
原来如此。那您觉得在单机游戏中购买皮肤和在网络游戏中购买皮肤的动机会有区别吗？

Informant 010 17:40:34
As for the stand-alone games, I just want to have a look how the new maps are.
单机是喜欢玩，想看看新的地图是如何的

Researcher 17:40:48
Curiosity?
好奇？

Informant 010 17:41:02

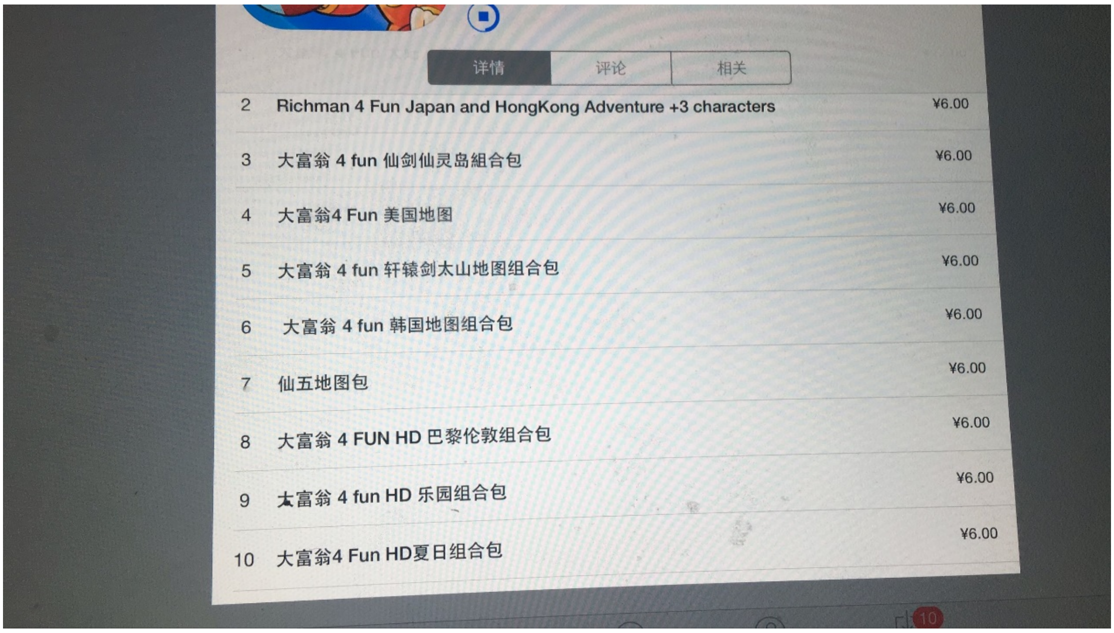

Informant 010 17:41:05
Yes, curiosity.
对好奇

Informant 010 17:41:49
I was curious how these maps were made.
就是好奇他会吧这些做成什么样子

Researcher 17:42:41
Ah, Ok. However, the producer didn’t give the preview graphics?
啊，好的。但是开发商不会给出预览图吗？

Informant 010 17:43:13
They only gave a small part of them.
只会给一小部分

Informant 010 17:43:18
They didn’t give them all.
不会给全部

Informant 010 17:43:29
And there is no interaction in the stand-alone game.
而且单机游戏他没有互动

Informant 010 17:43:39
The gameplay is always the same.

一直玩一样的

Informant 010 17:43:42
It’s boring as well.
也无聊啊

Researcher 17:44:44
I see. We have just talked about the impact of online friends on your purchase of in-game purchase decisions. Will real-life friends influence your purchase?
原来如此。我们刚才一直谈到线上的朋友对您购买游戏内购决策的影响，请问现实中的伙伴会对您的购买施加影响吗？

Informant 010 17:45:17
Yes.
会

Researcher 17:45:36
In general, how they influence you?
一般他们是怎么影响您的？

Informant 010 17:47:24
I have friends in the real life who play with me.
我有现实的朋友和我一起玩

Informant 010 17:47:38
Sometimes she like to communicate with me.
她有喜欢的就会和我交楼

Informant 010 17:47:48
And we would purchase the same items.
然后我们也会买一样的

Researcher 17:48:08
Well, the way of influence is the same as the online friends?
嗯嗯，也就是影响的方式和线上的朋友是一样的？

Informant 010 17:48:48
Similar.
差不多

Informant 010 17:48:59
But friends in the real life sometimes would stop me from purchasing.
但现实的朋友有时候会让别买

Researcher 17:49:25
Understood.
了解了。

Researcher 17:49:30
Let's talk about the lottery in the game. In the first interview, you mentioned: “Sometimes I can’t stop”,” I would be very unhappy if I always cannot get what I want or the repeat (skins).”,” I would be very happy when I get what I want.”,” Seeing others having won what I want, I will be envious”.
我们再来谈一下游戏内的抽奖。您在第一次访谈的时候谈到“有时候一抽停不下来”，“要是一直抽不到想要的或者重复的就会很不开心”，“抽到喜欢的就会很开心”，“看到别人抽到我想要的，就会很羡慕”

Researcher 17:49:40
Do you think this is a gambling-like experience?
您认为这是一种类似赌博般的体验吗？

Informant 010 17:49:55
Yes, I do
有

Informant 010 17:50:03
I would have this feeling.
有这种感觉

Informant 010 17:50:12
And I am relatively African.
而且我比较非洲人

Researcher 17:50:49
Well, apart from the feelings we mentioned last time, are there other behaviours or psychological activities when you play loot boxes?
嗯嗯，除了上次我们说的这些感觉外，您在玩抽奖箱的时候，还有其它的行为或者心理活动吗？

Informant 010 17:51:06
I would be nervous
会紧张

Researcher 17:51:31
Uh huh. Do you think this gambling-like experience affect your decision of buying in-game goods?

嗯嗯。您认为这种类似赌博般的体验会影响您购买游戏内购的决策吗？

Informant 010 17:51:41
Yes.
会

Informant 010 17:51:57
Sometimes the prize is not very good.
有时候奖品不是很好

Informant 010 17:52:06
Then I stop.
就不抽了

Researcher 17:52:54
Uh huh. Is this a contradiction with what we said last time, " Sometimes I can't stop"?
嗯嗯。这和我们上次说的"有时候一抽停不下来"有矛盾的地方吗？

Informant 010 17:53:24
In fact, there are more or less contradictions.
其实多多少少会有矛盾

Researcher 17:53:41
The mixture of wanting to continue and not wanting to continue?
既想继续又不想继续的感觉？

Informant 010 17:53:45
Yes.
对

Informant 010 17:53:48
Unwilling to give up
不死心

Researcher 17:54:27
I confirm one thing. You just said, " Sometimes the prize is not very good , then I stop
"
我确认一个事情哦。您刚才说到"有时候奖品不是很好，就不抽了"

Researcher 17:54:37
It's temporary or permanent?
是暂时不想抽还是永久性地不想抽了

Informant 010 17:54:50

There is nothing that attract me.
没有吸引我的

Informant 010 17:55:03
Temporary.
暂时

Researcher 17:56:07
I see. Also, do you think this gambling-like experience is related to the flow experience that we said before?
原来如此。另外，您认为这种类似赌博的体验和我们之前所说的心流体验有关联吗？

Informant 010 17:56:38
Yes, I have been unable to get what I want, which would let me stop the game.
有，一直抽不到自己要的。也会让我停止游戏

Researcher 17:57:12
Conversely, what happens if you always get what you want?
反过来说，如果一直抽到您想要的，会发生什么呢？

Informant 010 17:57:33
I would be happy.
那会很开心

Informant 010 17:57:39
I would keep continue playing.
会继续玩

Researcher 17:57:53
Is there the feeling of flow experience at this time?
在这个时候会有心流体验的感觉吗？

Informant 010 17:58:07
Rarely.
比较少吧

Researcher 17:58:16
I understand.
我明白了。

Informant 010 17:58:33
Because I was very happy at that time, I would like to share it with my friends.

因为那个时候很开心，会想和朋友分享

Researcher 17:59:15
Ah, just like the person in the group affected you as we just said, at this time you would show the items to others?
啊，就像刚才说的群里的那个人影响您一样，您在这个时候也会 po 给其它人？

Informant 010 17:59:25
Of course.
对啊

Informant 010 17:59:26
Hahaha.
哈哈哈

Researcher 17:59:56
OK. What do you usually say to your friends?
嗯嗯，好的。一般会和朋友们说一些什么？

Informant 010 18:00:43
How much I spend to win the prize.
我花多少抽到的

Informant 010 18:00:51
You guys could try as well.
你们也可以试试之类的

Researcher 18:01:19
Uh huh. On the contrary, will you share with your friends when you can't get your favourite items?
嗯嗯。相反抽不到心仪的道具的时候会和朋友们分享吗？

Informant 010 18:02:15
I would only
也就

Informant 010 18:02:19
complain.
会抱怨

Researcher 18:02:45
Ok. I understand.
好的。我明白了。

Researcher 18:02:47
The interview is almost over. Do you have any ideas to add?
访谈差不多要结束了。您还有什么观点需要补充吗？

Informant 010 18:02:58
No.

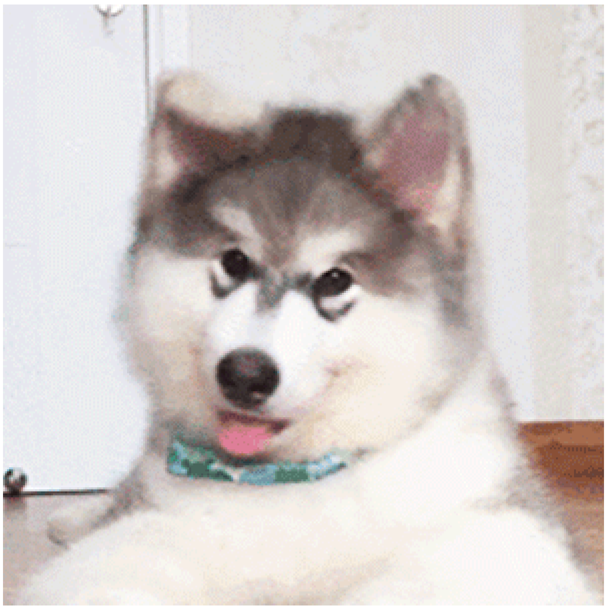

Researcher 18:03:38
These are all the questions. Thank you very much for participating in our research. Please confirm that your email address is XXXXXX@XXXXXX.com, because later we will send the JD electronic gift card to this address.
这就是全部的问题。 非常感谢您参与我们的研究。请确认您的电子邮件地址是XXXXXX@XXXXXX.com，因为稍后我们把京东电子礼品卡发送到这个地址。
