# Supplementary material for: A grounded theory approach to understanding in-game goods purchase
Source: PLoS One. 2022 Jan 27;17(1):e0262998. doi: 10.1371/journal.pone.0262998 (PMC8794092; doi:10.1371/journal.pone.0262998)
Supplement: S1 File — (ZIP) [file pone.0262998.s001.zip › Transcript 18.pdf]

Interview: 018

Informant: 009

Please note that the original transcript is in Simplified Chinese. The English translation is for internal communication among the author of this research, and it is not proofread. Potential linguistic errors may exist in the English translation.

Researcher 14:13:21
Thank you for your willingness to participate and be interviewed here. My name is XXX XXX, and I'm a PhD student in the XXX University of XXX(XXX). Currently, I'm working on a research project which focuses on videogame players' purchase motivations of in-game goods. Throughout this interview, I will ask you a series of questions and you are encouraged to express your opinions freely with emoticons. If I have questions about what you've said or need clarification about a topic or concept, I'll ask you.
感谢您愿意参加并在此接受采访。 我叫 XXX，我是市场营销学的博士生，现在我在 XXX 大学就读。 目前，我正在开展一个研究项目，专注于电子游戏玩家对游戏内购买项目的购买动机。 在整个访谈中，我会问您一系列问题，我们鼓励您自由表达您的意见和观点。因为这不是一个当面访谈，所以我们也鼓励您用 QQ 表情来表达您的情绪。 在访谈过程中，如果我对你所说的内容有疑问或需要您澄清一个主题或概念，我会问您。

Researcher 14:13:27
Are you ready?
您准备好了吗？

Informant 009 14:13:35
Yes, I'm ready.
嗯嗯 准备好了

Researcher 14:13:39
Ok. In the last interview we mentioned the concept of "Flow experience". Let's review this concept:
我们上一次访谈的时候提到了”心流体验“这个概念。我们来回顾一下这个概念：

Researcher 14:13:44
"Flow experience" has been used by psychologist to describe a state of mind experienced by people who are deeply involved in an activity. Instance, sometimes while playing videogames, the player's action and awareness are merged, and he/she is totally connected on the gaming tasks at hand. In this state, the player loses his/her consciousness, and his/her perception of time becomes faster or slower than usual. Also, the player perceives a feeling of being in control, which empowers him/her from the fear of failure.

“心理学家使用“心流体验”来描述深度参与某项活动的人所经历的心理状态。 例如，有时玩家在玩电子游戏时，他/她的动作和意识会融为一体，并且他/她完全关注手头的游戏任务。 在这种状态下，玩家失去他/她的自我意识，他/她对时间的感知变得比平时更快或更慢。 此外，玩家会感受到一种掌控全局的感觉，这使他/她免于对失败的恐惧。”

Researcher 14:13:51
In the last time, you mentioned “Slower perception of time. (I am) too dedicated, (and always forget the time while playing)”, “Sometimes I know that it's late but I can't stop.”
您上次有谈到“对时间感知比较慢 太投入了 一玩玩过头了”，“有时明知道晚了 还是停不下来”

Researcher 14:13:57
After acquiring the flow experience, how does this experience end? Which events would cause the termination of this experience?
请问您在获得心流体验之后，这个体验一般是怎么结束的？哪些事件导致了这种体验的终止？

Informant 009 14:15:00
Sometimes the gameplay lasts until 2 pm, (then) I tell myself that I must go to bed, otherwise the body will get worse.
有时候会持续到晚上 2 点 告诉自己必须要睡觉了 不然身体会变差

Informant 009 14:15:31
In the afternoon, it usually lasts until the eyes are sour or my parents tell me to go to dinner.
下午的话 一般持续到眼睛酸了 或者爸妈叫我去吃饭了

Researcher 14:16:02
Uh huh. I see. That is, you intentionally suspend the game, then the flow experience is ended, right?
嗯嗯。原来如此。也就是自己主动暂停游戏，然后心流体验就终止了，对吧？

Informant 009 14:16:11
Yes.
对的

Researcher 14:16:26
Are there some in-game events that could end your flow experience?
有没有什么游戏内的时间会让您的心流体验终止呢？

Researcher 14:16:35
*events.

*事件

Informant 009 14:17:23
Yes. I play Wangzherongyao and always lose, and the ranking falls off.
有 打王者荣耀一直输掉排位 不想打了 太气

Researcher 14:18:00
I see. This is consistent with the anxious experience that we talked about last time: “The teammates responded very slowly, and he/she didn't listen to the command. You told him/her to use shield soldiers but he/she used gunman instead. Sometimes someone in the team put the whole team in the pit because he/she was too selfish and wanted to swipe the scores.”, “Endure. Actually, I really want to curse. I feel anxious.”.
原来如此。这和我们上次谈到的焦虑的体验是一致: "打团队赛 队友反应迟钝 不听指挥 让他进盾他进了队枪兵 感觉被坑了 有时候团队赛由于个别人的自私他想刷分刷 把全队坑了"，“忍着 心里其实很想骂人 觉得烦躁”

Informant 009 14:18:17
Yes.
对的

Researcher 14:18:27
Also, last time we talked about the boring experience: “there are fewer and fewer people playing in one server, and it no longer makes sense. Or (I) have upgraded to the highest level, and (I) feel like there is nothing to do.”,” It will be boring without new activities.”,” Or if the activities are unchanged, it’s boring.”,” Playing mechanically causes numbness.”
另外，上次我们还谈到了无聊的体验：比方说这个区玩的人越来越少 感觉没什么意义了 要么升级升到最高了 感觉没什么好做的了"，“不出新的活动 就会无聊”，“要么活动一成不变也很无聊”，“机械式的玩 会麻木”

Researcher 14:18:31
Do you think that the end of the flow experience is related to the boring experience?
您认为心流体验的终止和无聊的体验有关系吗？

Informant 009 14:18:46
Yes, three are connections.
有关的

Informant 009 14:18:57
Too boring, and it’s not interesting. Then I have not intention to continue (playing).
太无聊 无趣 也没心思继续下去了

Researcher 14:19:41
Well, that is to say, even if you enter the flow experience, this experience could be

ended because of anxiety or boredom. Can I interpret like this?
嗯嗯，也就是说，即便是进入了心流体验，也会因为焦虑或者无聊而终止这种体验，我能这么理解吗？

Informant 009 14:19:55
Yes.
是的

Researcher 14:20:36
If you are always in the state of flow experience during the gameplay, would you still choose to buy in-game goods?
如果玩游戏的时候，始终处于心流体验的状态，您还会选择购买游戏内购吗？

Informant 009 14:20:49
Yes, I would purchase.
会购买

Researcher 14:20:56
Can you give me an example?
能举个例子吗？

Informant 009 14:22:23
For example, I now want to break through a realm and turn my dragons from purple to orange and gold dragons. I would buy in-game goods by purchasing dragon searching calls to draw dragons.
比方说我现在很想突破一种境界，把自己的龙脉一个个从紫色突破成橙色出金龙，我就会购买游戏内购，买寻龙令去抽龙脉

Researcher 14:23:50
Uh huh. I want to confirm one thing. Which state is the flow state? Before and after purchase?
嗯嗯。我想确认一个事情。这边购买前和购买后哪种状态是心流体验的状态？

Researcher 14:24:35
Or you are already in the state of flow experience, and you want to maintain this state by purchasing in-game goods?
或者都处于心流体验的状态，您想通过购买游戏内购来维持这种状态？

Informant 009 14:24:50
Both
都有

Informant 009 14:25:05
Before purchasing I would like to empower, and after having the lottery I am excited.

买钱想提升 买完抽奖激动

Researcher 14:25:51
I see.
原来如此。

Informant 009 14:26:35
Yes. Make the game more fulfilling.
嗯 让游戏变得更充实

Researcher 14:26:57
Sorry, I still want to know more about the details of this issue.
不好意思，我还是想更多地了解一下这个问题的细节。

Researcher 14:27:04
The previous example.
刚才的例子

Researcher 14:27:16
Purchasing dragon searching calls to draw dragons
买寻龙令去抽龙脉

Researcher 14:27:41
Before purchasing it, you are already in the state of flow experience, right?
在购买前，已经处于心流体验的状态，对吧？

Informant 009 14:28:23
Yes.
对的

Researcher 14:30:02
Ok. I understand.

好的。我明白了。

Researcher 14:30:08

我们现在来谈一下皮肤类型的游戏内购。您在第一次访谈的时候说到"像玩农药就要买皮肤 感觉每个英雄都要美美的"，"也有属性的 乱世的皮肤又拉风，又有属性"，"因为农药这个就一直在操作，有个视觉效果体验，放个技能什么的都跟皮肤有关"

Informant 009 14:30:12
比方说你紫色突破有 5 个，要 10 个才能突破成紫色。你就想现在突破，那就要

去抽了，抽奖也不一定能抽到，就会持续这个状态

Informant 009 14:30:29
Yes.
对的

Researcher 14:30:50
Ok. Do you think people around you (offline or online) have an impact on your purchase of in-game goods?
嗯嗯好的。您认为您周围的人（线下或线上）对您购买游戏内商品有影响吗？

Informant 009 14:31:08
Yes. The online one is definitely good, because it’s convenient.
有 肯定线上好 方便

Researcher 14:31:40
How do people from the online environment influence your purchase of in-game goods?
线上的人是怎么影响您购买游戏内商品的呢？

Informant 009 14:32:31
I feel that in the online environment, I prefer to behave in front of the strangers.
我感觉线上在不认识的人面前 更喜欢表现

Researcher 14:33:07
Uh huh. Can you give a concrete example?
嗯嗯。能不能举一个具体的例子呢？

Informant 009 14:34:00
I am Ok with myself. But I have seen more in the game~ Although people are very I have ordinary in the real life, they recharge a lot for making others believe that they are rich. I remember that there is a person who does the warehouse management, and he/she has to take delivery every day, however, he/she recharges a lot in the game.
我自己还好。但游戏里我见多了～明明生活过的很普通，却要充钱买很多，让别人觉得很有钱一样。记得有个人做仓管的，天天还要送外卖，游戏里充的钱却很多很多

Informant 009 14:34:25
I feel that many people now like to be pretentious in the game.
感觉现在很多人喜欢在游戏里装 B

Researcher 14:35:03
I see. You said before that you have joined the league inside the game. Do the members of the league have an influence on your purchase of in-game goods?
原来如此。您之前说过您有加入游戏内的联盟，请问联盟内的成员对您购买游戏

内商品有影响吗？

Informant 009 14:35:17
Yes.
有的

Researcher 14:35:36
How do they generally affect your in-game consumption?
他们一般是怎么影响您的游戏内消费的？

Informant 009 14:35:50
If you don’t have a treasured military commander, you can’t participate in the senior team competitions like Chuhan or Imperial City.
没有珍宝台武将的话不能参加楚汉，皇城这种高级团队赛

Researcher 14:36:41
Uh huh. Is there a mentality of wanting to be like everyone else?
嗯嗯。有一种想要和别人一样的心态？

Informant 009 14:37:19
Although I don’t achieve to be No 1., but I don’t want to be too backward.
虽然不争做第一，但也不能太落后

Researcher 14:38:16
Well, I understand. Do the people in real life have an impact on your purchase of in-game goods?
嗯嗯我明白了。那线下的人会对您购买游戏内商品有影响吗？

Informant 009 14:38:35
They also have.
也会有吧

Researcher 14:38:41
How do they generally affect your in-game consumption?
他们一般是怎么影响您的游戏内消费的？

Informant 009 14:38:49
Advance and retreat jointly.
共同进退

Informant 009 14:39:00
It means “Go forward” together.
就是一起“前进”

Informant 009 14:39:07
Making ourselves stronger.
把自己弄牛逼

Researcher 14:39:37
Well, what kind of people would have an impact on your in-game consumption?
嗯嗯，一般什么样的人会对您的游戏内消费产生影响？

Informant 009 14:40:31
For example, in this battle type of games, when people hit my boyfriend, he would be very angry. Then, he would recharge the money, be stronger, and fight back. There is a sort of protective mentality.
我们这种对战的，比方说我男朋友，看到有人打他，就很生气，就会充钱，变牛逼打回去，有种保护的心理

Informant 009 14:40:40
He would do the same if I have been beaten.
他看到我被打同样也会

Researcher 14:41:47
Ok, I understand. I need to confirm one thing. Does the influence of these people that we have just mentioned only affects the purchase of skin types? Or they apply to other types of in-game goods as well? (Power-up items, Expansion packages, Playable characters, Loot boxes, Time-savers)
原来如此，我明白了。我确定一件事情，刚才我们谈到的这些周围人对您的影响仅限于皮肤类的内购呢？还是对其它类型的游戏内购也一样？(增强道具 扩展包 可游玩的角色 抽奖箱 省时道具)

Informant 009 14:42:35
Others as well.
其他的

Informant 009 14:42:51
Respect to skins, we would buy couple skin.
皮肤的话，我们会购买情侣皮肤

Researcher 14:43:20
I see.
原来如此。

Informant 009 14:43:32
I feel it’s prettier when using together.
感觉一起用很好看

Researcher 14:43:55
Do you buy skins in stand-alone games?
您会在单机游戏中购买皮肤吗？

Informant 009 14:44:06
No.
不会

Informant 009 14:44:20
I rarely recharge money in stand-alone games.
单机游戏基本不怎么充钱

Researcher 14:44:49
Ok. Why do you buy skins in online games instead of buying them in stand-alone games?
好的。为什么您会在网络游戏中购买皮肤而不在单机游戏中购买皮肤呢？

Informant 009 14:44:56
I recharged once when I grow vegetables in farm.
就种菜的时候 玩农场的时候冲过

Informant 009 14:45:26
I feel that stand-alone games can be played slowly, which does not have the same fast rhythm as online games. I feel that online games cannot be played slowly, because later I can't keep up with the rhythm.
单机感觉自己可以慢慢玩 没网游节奏快 网游感觉慢慢玩不行 跟不上节奏

Informant 009 14:46:03
Another point for online games: Once you are left behind, you are out of the mass, which makes the gameplay boring.
网游还有其他人 你一个落后了 就脱离群众了 玩的没意思了

Informant 009 14:46:28
As for the stand-alone games, I can play and stop. It doesn't matter. I can play when I am free.
单机游戏 我玩玩停停 无所谓的 空着就玩

Informant 009 14:46:37
In terms of online games, it seems like as if there were assignments.
网游像有指标似得

Researcher 14:47:04
I see. I understand.
原来如此，我明白了。

Researcher 14:47:10
We move to the next topic.
我们继续下一个话题。

Researcher 14:47:12
Let's talk about the lottery in the game. You talked about during your first interview:" Yes. Military commanders of Treasures are also a pit. They are pumped by Luck."
我们再来谈一下游戏内的抽奖。您在第一次访谈的时候谈到"珍宝台武将也很坑的，它凭运气抽的"

Researcher 14:47:21
Do you think this is a gambling-like experience?
您认为这是一种类似赌博般的体验吗？

Informant 009 14:47:29
Yes.
对的

Researcher 14:47:33
Do you think this gambling alike experience affects your purchase of in-game goods?
您是否认为这种类似赌博般的体验会影响您购买游戏内购？

Informant 009 14:48:02
Just like gambling. If you are lucky, you can spend a small amount of money. Otherwise, if you are not lucky, you need to draw until the last one.
就像赌博 你运气好 可以花小钱抽到 运气不好 你就翻牌翻到最后一张

Informant 009 14:48:11
No.
不会

Informant 009 14:48:32
If I really want it, even if I know it is a pit, I would go to draw it.
非常想要的话 就算知道是坑 也会去抽

Informant 009 14:48:44
(I pretend as if I) had spent 3500 RMB to buy it.
就当花 3500 块买的

Researcher 14:49:01
Even if you always can't acquire the desired item, will you still draw for it?

即便是总是抽不到，依然会去抽吗？
Informant 009 14:49:20
(Yes Yes Yes Yes)

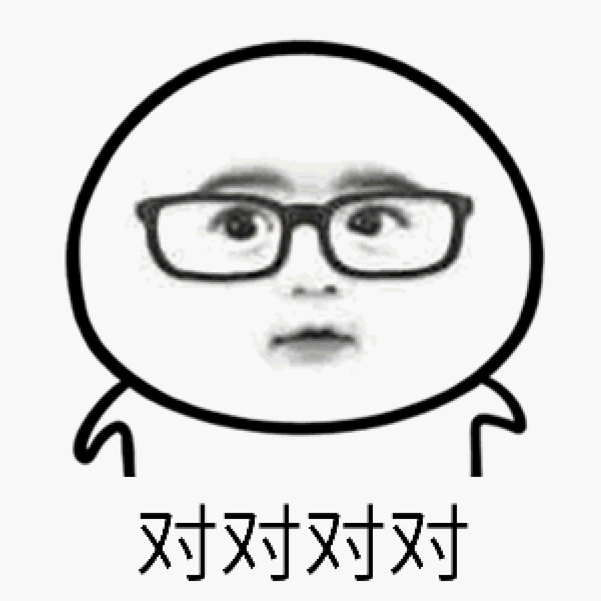

Informant 009 14:49:54
Because there are 15 (items) in total, which are not repeat. In the end, there will be one.
因为它一共就 15 个 不会重复的 抽到底总会有

Researcher 14:50:02
Ok. Generally, when you are participating in a lottery, what do you think mentally?
好的。一般您在进行抽奖的时候，心里一般是怎么想的？

Informant 009 14:50:29
I hope I could get the items before the last round, and I would feel lucky if I get the item in the first round.
希望不要抽到底 能立马中就发了

Informant 009 14:50:46
Although I know it’s impossible.

虽然知道这是不可能的

Informant 009 14:51:04
Tencent is not that good.
腾讯哪有那么好

Researcher 14:51:17

Uh huh. Do you think this kind of gambling-like experience is related to before mentioned flow experience?
嗯嗯。您认为这种类似赌博般的体验和我们刚才所说的心流体验有关系吗？

Informant 009 14:52:15
Yes, both of them are hard to resist.
有吧 都让人无法抗拒

Researcher 14:53:39
Ok, I understand.
好的，我明白了。

Informant 009 14:53:50
Yes Yes.
嗯嗯

Researcher 14:54:20
In other words, when you are participating in a lottery, you already have a psychological expectation: In the end, I can get the desired item in the last round of the lottery, right?
也就是说，您在进行抽奖的时候，已经有了心理预期：大不了最后一次抽奖才抽到想要的，是吗？

Informant 009 14:55:26
Yes.
对的

Researcher 14:56:44
Ok. I have done for this part. I would like to go back to the flow experience as we started, because I still have some questions.
好的。这部分的问题我问完了。我想再回到我们刚开始的心流状态，因为我还是有一些疑问。

Informant 009 14:56:58
Ok.
好的

Researcher 14:57:27
What is your status if you have not experienced the anxious experience and the boring experience considering that you are already in the flow state?
请问如果在已经进入心流体验的状态下，没有焦虑体验和无聊体验的干扰，您的状态是什么样的？

Informant 009 14:58:16

激动 兴奋

Researcher 14:58:29
In other words, you still maintain the flow experience, right?
也就是依然保持着心流状态，对吧？

Informant 009 14:58:30
I feel I can go to the next round after this round.
感觉打完一盘再来一盘

Informant 009 14:58:35
Yes.
对的

Researcher 14:58:58
You will not go offline until you are tired or too late?
直到累了或者太晚了才会下线？

Informant 009 14:59:28
The flow experience is strongest when (playing) the ranking. I need to accumulate constantly the points to achieve Golden, Platinum, and King.
上排位最心流状态最强 我要不断的积分 才能从黄金到铂金到钻石到王者

Informant 009 14:59:30
Yes.
对的

Informant 009 14:59:40
When I really can’t play anymore, I go offline.
实在玩不动了 就下线了

Informant 009 14:59:59
After all, I’m an employee, and I can’t afford to stay up late.
毕竟上班族 熬夜伤不起

Informant 009 15:00:06

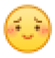

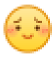

Researcher 15:00:28
Does the process of accumulating points cost money?
这个积分的过程花钱吗？

Informant 009 15:00:39
It doesn’t
不花钱

Informant 009 15:00:45
As long as I win.
只要能赢

Informant 009 15:00:49
It depends on the skill.
看水平

Researcher 15:02:24
Ok. We just mentioned: “I am Ok with myself. But I have seen more in the game~ Although people are very I have ordinary in the real life, they recharge a lot for making others believe that they are rich. I remember that there is a person who does the warehouse management, and he/she has to take delivery every day, however, he/she recharges a lot in the game.”,” I feel that many people now like to be pretentious in the game.”
好的。我们刚才还谈到了“ 我自己还好。但游戏里我见多了～明明生活过的很普通，却要充钱买很多，让别人觉得很有钱一样。记得有个人做仓管的，天天还要送外卖，游戏里充的钱却很多很多”，“感觉现在很多人喜欢在游戏里装 B”

Researcher 15:02:35
Do you think this is a conspicuous consumption?
您觉得这是一种炫耀性消费吗？

Informant 009 15:02:48
More or less.
有点

Researcher 15:03:09
Does the conspicuous consumption of others affect your in-game goods purchasing?
请问他人的炫耀性消费会对您的游戏内商品消费产生影响吗？

Informant 009 15:03:18
No, there is not influence.
没影响

Informant 009 15:03:26
I just watch, and I don’t compare.
就看看 不攀比

Researcher 15:03:46
I see, ok.
原来如此，好的。

Researcher 15:03:47
The interview is almost over. Do you have any ideas to add?
访谈差不多要结束了。您还有什么观点需要补充吗？

Informant 009 15:03:55
Nope.
木有了

Researcher 15:04:14
These are all the questions. Thank you very much for participating in our research. Please confirm that your email address is XXXXXX@XXXXXX.com, because later we will send the JD electronic gift card to this address.
这就是全部的问题。 非常感谢您参与我们的研究。请确认您的电子邮件地址是XXXXXX@XXXXXX.com，因为稍后我们把京东电子礼品卡发送到这个地址。
