# Supplementary material for: A grounded theory approach to understanding in-game goods purchase
Source: PLoS One. 2022 Jan 27;17(1):e0262998. doi: 10.1371/journal.pone.0262998 (PMC8794092; doi:10.1371/journal.pone.0262998)
Supplement: S1 File — (ZIP) [file pone.0262998.s001.zip › Transcript 19.pdf]

Interview: 019

Informant: 013

Please note that the original transcript is in Simplified Chinese. The English translation is for internal communication among the author of this research, and it is not proofread. Potential linguistic errors may exist in the English translation.

Researcher 10:14:00
Thank you for your willingness to participate and be interviewed here. My name is XXX XXX, and I'm a PhD student in the XXX University of XXX(XXX). Currently, I'm working on a research project which focuses on videogame players' purchase motivations of in-game goods. Throughout this interview, I will ask you a series of questions and you are encouraged to express your opinions freely with emoticons. If I have questions about what you've said or need clarification about a topic or concept, I'll ask you.
感谢您愿意参加并在此接受采访。 我叫 XXX，我是市场营销学的博士生，现在我在 XXX 大学就读。 目前，我正在开展一个研究项目，专注于电子游戏玩家对游戏内购买项目的购买动机。 在整个访谈中，我会问您一系列问题，我们鼓励您自由表达您的意见和观点。因为这不是一个当面访谈，所以我们也鼓励您用 QQ 表情来表达您的情绪。 在访谈过程中，如果我对你所说的内容有疑问或需要您澄清一个主题或概念，我会问您。

Researcher 10:14:05
Are you ready now?
您准备好了吗？

Informant 013 10:14:11
Yes.
嗯

Researcher 10:14:26
You mentioned in the previous questionnaire that you have purchased different sorts of in-game goods in the past six months, including Cosmetic/Skins and Loot boxes.
您在之前的问卷中提到您在近六个月内购买过不同种类的游戏内购，包括装饰/皮肤和抽奖箱。

Informant 013 10:14:31
Text or voice?
文字 or 语音？

Informant 013 10:14:43
Yes.
对

Researcher 10:14:58
Text. Because I need to do script compilation later.
文字吧。因为我之后需要做脚本编译

Researcher 10:15:01
What are your motivations for purchasing Cosmetic/Skins type in game goods?
请问您购买装饰/皮肤类游戏内购的动机是哪些？

Informant 013 10:15:25
Cosmetic/Skins type is for good looking.
装饰类就是好看呗

Informant 013 10:16:10
After all, there is no enhancement of attributes.
毕竟没有游戏属性提升

Researcher 10:16:30
Humm, OK.
嗯好的。

Researcher 10:16:31
Do you buy more skins in stand-alone games or buy more skins in online games?
您是在单机游戏中购买皮肤多还是在网络游戏中购买皮肤多？

Informant 013 10:16:49
In Online games.
网游

Informant 013 10:16:59
Skins are rarely sold in a stand-alone games.
单机里很少卖皮肤。。

Researcher 10:17:11
Why do you buy more skins in online games than stand-alone games?
为什么您在网络游戏中购买皮肤要比单机游戏多呢？

Informant 013 10:18:04
It’s for purely fun gameplay.
纯粹是玩的开心

Informant 013 10:18:10
Attractive effects.
特效好看

Researcher 10:18:19
Ok, I see.
恩，原来如此。

Researcher 10:18:21
Do you think people around you (offline or online) have an impact on your purchase of in-game goods?
您认为您周围的人（线下或线上）对您购买游戏内商品有影响吗？

Informant 013 10:18:39
No.
没有

Researcher 10:19:13
In other words, if you think that the special effects are good, you will buy it. Is that true?
也就是说，您认为特效或者外观好看，就会去购买，是这样吗?

Informant 013 10:19:25
Yes.
对的

Researcher 10:19:29
Have you ever joined the league in online games?

您在网络游戏内加入过工会吗？

Informant 013 10:19:36
Yes.
嗯

Researcher 10:20:08
If the members in the league show the in-game goods they buy, will they affect your willingness to buy?
如果工会内的小伙伴们晒出他们购买的游戏内购,会对您的购买意愿产生影响吗？

Informant 013 10:20:19
No.
不会

Researcher 10:20:41
I see. Ok. Let's move on to the next topic.
原来如此。好的。我们继续下一个话题。

Informant 013 10:20:43

If they all buy it… maybe I’m not going to buy.
都买的话。。我有很能就不买了

Researcher 10:20:57
Oh, it turn out to be the case.
啊原来是这样。

Researcher 10:21:12
Can you give an example of what happened in the past?
您可以举一个过去发生过的例子吗？

Informant 013 10:21:20
Which one?
哪种？

Researcher 10:21:33
The case that others have bought the skin, and your purchasing intention falls down.
就是看到别人都购买了，您的购物意愿下降的例子
Informant 013 10:22:00
At that time, the starlight dragon in Warcraft
当时魔兽里的星光龙

Researcher 10:22:27
Well, it’s too early. I also paid attention to it at the time.
嗯嗯，比较早了。我当时也有关注过。

Researcher 10:22:50
The 7.0 era, right?
7.0 时代的，对吧。

Informant 013 10:23:03
The Panda version.
熊猫人版本

Researcher 10:23:13
Humm, my memory is wrong.
嗯嗯，记错了。

Informant 013 10:23:17
I couldn’t get it, and I was prepared to purchase it in the mall.
刷不出来，准备在商城买

Researcher 10:23:31
At that time, everyone else had a starlight dragon, and then you didn’t want it anymore,

right?
那时候就是别人都有了星光龙，您就不想要了，是吗？

Informant 013 10:23:31
Then I saw several dragons later.
后来看见了好几条

Informant 013 10:23:41
Yes, I didn’t purchase it.
嗯，就没买

Researcher 10:24:21
At that time, you didn’t buy Starlight Dragon, but would you choose to buy the other in-game goods using the money that initially for purchasing the Starlight Dragon?
在那个时候，没有买星光龙，但是您会选择拿原来想买星光龙的钱买其它的内购吗？

Informant 013 10:24:52
Nope.
没有，

Researcher 10:25:10
Ok. Let's talk about the purchase of loot boxes.
好的。我们接下来来谈谈抽奖箱的购买。

Researcher 10:25:16
What are your motivations of purchasing loot box type in-game goods?
请问您购买抽奖箱类游戏内购的动机是哪些？

Informant 013 10:25:34
这个为了里面的奖励

Researcher 10:25:55
That is to acquire other in-game goods, right?
也就是能开出其它的游戏内购，对吧？

Informant 013 10:26:11
Yes.
对的

Researcher 10:26:15
Do you have a gambling-like experience in the process of drawing with the loot box?
您在抽奖的过程中，有没有过类似赌博般的体验？

Informant 013 10:26:30
This is a kind of gambling.
这个就是一种赌博吧

Informant 013 10:26:36
The reward is very attractive
奖励很诱人

Informant 013 10:26:44
The probability is very limited.
几率却很小

Researcher 10:27:10
Ok. Would your mindset or behaviours be different when having this sort of experience?
What changes in mindset and behavior when you are in this experience?
好的。那您处于这种体验的时候，心态和行为上有什么变化？

Informant 013 10:27:43
Generally, I would like to follow my heart.
一般开的时候都是想随缘

Informant 013 10:28:20
One time I opened a lot boxes but got nothing.
有过一次性开了大量的却什么都没出

Researcher 10:28:32
What did you think at that time?
这时候心里是怎么想的？

Informant 013 10:28:47
Open~ I have money ~ Just open.
开啊~有钱~开
Researcher 10:29:03
If you don't acquire the desired item, you just continue opening boxes, right?
没有开到想要的就继续开，对吗？

Informant 013 10:29:09
Yes.
对

Researcher 10:29:23
Will there be some negative emotions?
心里会不会有一些负面情绪？

Informant 013 10:30:09
If it’s over budget but the item doesn’t show, I would be bit angry.
预算开完了也不出，会有点气

Researcher 10:30:42
I see. In another case, if you get what you want from the box, what do you think?
原来如此。另一种情况，如果是开到自己想要的呢？心里会怎么想？

Informant 013 10:30:53
Then, I would be happy.
那就开心了

Researcher 10:31:07
Do you think this gambling alike experience affects your purchase of in-game goods?
您认为这种类似赌博般的体验会影响您购买游戏内购的决策吗？

Informant 013 10:31:08
I could use it myself or change for some RMBs. Both are good.
自己用掉啊，换 RMB 啊，都好

Informant 013 10:31:21
Now I don’t.
现在不会了

Researcher 10:31:33
How did it affect before?
以前会怎么影响？

Informant 013 10:32:00
Now I basically play games without boxes.
现在玩的基本上没有开盒子的

Informant 013 10:32:26
Occasionally there are one or two games.
就算有，也是偶尔一两个

Researcher 10:32:49
I would like to know more details. Why did you open more boxes before, but now you open less?
我想了解一些细节。请问为什么以前开盒子多，但是现在开得少了呢？

Informant 013 10:33:32
Concretely, games like DNF or Honkai Impact 3.
具体说 DNF 或崩坏 3 这种

Researcher 10:33:40
Yes, please continue.
嗯嗯您说

Informant 013 10:34:15
I was merged in DNF, I spent some pocket money in the game.
DNF 原来玩的入迷，有点零钱也要花进去

Informant 013 10:34:37
Gradually I play less.
现在慢慢玩的少了

Informant 013 10:34:46
My characters have been established.
人物也成型了

Informant 013 10:34:53
Then my needs are less.
需求就少了

Informant 013 10:35:25
Items can be brushed out, or purchasing them directly
东西也能刷出来，或直接买道具

Researcher 10:35:33
I understand. This is to say that there is less and less unlocked contents in the game, so there is no incentive to open boxes again, right?
我明白了，也就是游戏内未解锁的内容越来越少了，就没有动力再去开箱子了，对吧？

Informant 013 10:35:40
Yes.
对

Researcher 10:36:09
Understood. Let's move on to the next topic.
了解了。我们来继续下一个话题。

Researcher 10:36:11
“Flow experience” has been used by psychologist to describe a state of mind experienced by people who are deeply involved in an activity. Instance, sometimes while playing videogames, the player’s action and awareness are merged, and he/she is totally connected on the gaming tasks at hand. In this state, the player loses his/her

consciousness, and his/her perception of time becomes faster or slower than usual. Also, the player perceives a feeling of being in control, which empowers him/her from the fear of failure.
心理学家使用“心流体验”来描述深度参与某项活动的人所经历的心理状态。 例如，有时玩家在玩电子游戏时，他/她的动作和意识会融为一体，并且他/她完全关注手头的游戏任务。 在这种状态下，玩家失去他/她的自我意识，他/她对时间的感知变得比平时更快或更慢。 此外，玩家会感受到一种掌控全局的感觉，这使他/她免于对失败的恐惧。

Researcher 10:36:18
Please recall your own gaming experience. Have you had the flow experience when playing video games?
请回想一下您自己的游戏经验。 您玩电子游戏时有没有经历过心流体验？

Informant 013 10:37:15
Yes, I had.
有的吧

Researcher 10:37:24
Please tell me what happened when you came to the flow state? I mean your behavioural and psychological activities during this course.
请告诉我您在进入到心流体验的时候发生了什么？ 我的意思是您在这个过程中的行为和心理活动。

Informant 013 10:38:16
Play through the night.
玩到通宵啊

Informant 013 10:38:44
Mentally...There is no mental movement.
心理。。。没什么活动吧

Informant 013 10:39:11
Play more~ play more?
再玩会~再玩会？

Researcher 10:39:13
I see. When you are in this state, would you increase your gaming time?
原来如此。就是处于这个状态的时候，游戏时间会变长吗？

Informant 013 10:39:40
Yes.
是的

Informant 013 10:39:40
Ok.
嗯

Researcher 10:39:48
Yes.
好的。

Researcher 10:39:49
How do you end the flow experience after having gained this experience? What events have caused the termination of this experience?
请问您在获得心流体验之后，这个体验一般是怎么结束的？哪些事件导致了这种体验的终止？

Informant 013 10:40:42
Tasks in real life or someone else is calling.
现实里的任务或者别人叫着

Researcher 10:41:11
That is, the objective factors of the surrounding environment force you to end the game.
也就是周围环境的客观因素让您被迫终止游戏。

Informant 013 10:41:23
Yes.
对

Informant 013 10:41:37
The gameplay cannot be ended.
游戏玩不完

Researcher 10:41:47
Is there a situation in which you have already entered the state of flow experience, but you subjectively suspend the game?
有没有自己已经进入了心流体验的状态，但是自己主动暂停游戏的情况？

Informant 013 10:42:29
I could be hungry. I also remember that sometimes the task ends, and I would find new tasks.
这个可能就是饿的，我记得有时任务完成也会再去找新任务

Informant 013 10:42:47
Keep playing.
继续玩下去

Researcher 10:43:22
In other words, some physiological needs (hungry to eat, etc.) would make you end this experience, right?
也就是说，一些生理需求（饿了要吃饭等等）会让您终止心流体验，对吧？

Informant 013 10:43:43
Yes.
嗯，

Researcher 10:43:52
Ok. Have you had the anxious experience while playing games?
好的。您在游戏时有过焦虑的经历吗？

Informant 013 10:44:10
No, I haven’t.
应该没有

Informant 013 10:44:22
Just for entertainment.
为了娱乐嘛

Researcher 10:44:27
Ok. Have you had the boring experience in the game?
好的。那您在游戏时有过无聊的经历吗？

Informant 013 10:44:42
I should have.
应该有的

Researcher 10:44:50
Under what circumstance do you have?
通常在什么情况下有？

Informant 013 10:45:03
After brushing fatigue value
刷完疲劳值

Informant 013 10:45:10
Strength value.
体力值

Informant 013 10:45:15
Yes…
嗯。。。

Researcher 10:45:28
What would you do if you had a boring experience in a game? Will you buy in-game goods to alleviate anxiety?
如果您在游戏中有无聊的经历，您会怎么做?您会购买游戏内的商品来缓解无聊吗？

Informant 013 10:45:42
No.
不会

Informant 013 10:45:57
But
但是

Informant 013 10:46:27
If the item is for refilling the fatigue value, I would buy it.
要是有补充疲劳值的我可能也会买

Researcher 10:46:57
Uh huh. I don't know much about the concept of fatigue value. How does it generally work?
嗯嗯。我不太了解疲劳值这个概念，一般它是怎么运作的？

Informant 013 10:47:57
It is a thing that limits how many times you can enter to the maps or clear the stages.
就是每天限制你进多少地图，闯多少关的一个东西

Informant 013 10:48:12
It refills automatically at 6 am every day.
每天凌晨 6 点自动补满

Researcher 10:49:04
I see. If the fatigue value is used up and you still want to play in that day, you would consider purchasing the in-game item to recharge the fatigue value.
原来如此，就是当天您还想玩，但是疲劳值用完了。这个时候您会考虑购买充值疲劳值的内购，对吗？

Informant 013 10:49:22
I would.
应该会的

Researcher 10:49:40
If at this time, you don’t purchase this type of items, what is the gaming experience like?

如果这个时候不购买这种道具的话，游戏体验是什么样的？

Informant 013 10:50:17

When the strength is 0， there is no experience.
体力为 0，就没有体验了

Researcher 10:50:30
The game will become boring?
游戏会变得无聊？

Informant 013 10:50:36
I can only stay in town and cannot beat the enemies.
只能呆在城镇里，不能刷怪
Informant 013 10:50:51
Can.
会

Researcher 10:51:17
I see.
原来如此。

Informant 013 10:51:33
Yes.
嗯

Researcher 10:51:33
Once after having the flow experience, will you come back to the game to regain this experience?
另外，一旦获得过心流体验，您会想回到游戏中为了重新获得这种体验吗？

Informant 013 10:52:17
No, no.
没有吧，没有

Researcher 10:52:37
Ok. Generally, what are the reasons for you to give up a game?
好的。一般您放弃一款游戏的原因有哪些？

Informant 013 10:52:37
Maybe I don't want to play in the short term.
可能短期内还不想玩了

Informant 013 10:53:00

I meet new games, or have something important to do
遇到新的游戏，或有很重要的事情忙

Researcher 10:53:52
I see. In other words, after having the flow1experience, you don’t want to play the same game in a short period of time. Is that true?
原来如此。也就是说，获得过心流体验后，可能短期内就不想玩同一款游戏了，是这样吗？

Informant 013 10:54:28
Umm, Yes.
嗯，是

Researcher 10:54:56
嗯嗯好的。访谈差不多要结束了。您还有什么观点需要补充吗？

Informant 013 10:55:07
At that time, it was lol, dnf, wow, playing back and forth.
当时就是 lol ，dnf ，wow，来回换着玩

Informant 013 10:55:25
Generally, (I played) in a period of time.
经常一段时间就玩够了

Researcher 10:56:05
I see, I understand. These are all the questions. Thank you very much for participating in our research. Please confirm that your email address is XXXXXX@XXXXXX.com, because later we will send the JD electronic gift card to this address.
原来如此。我了解了。这就是全部的问题。非常感谢您参与我们的研究。请确认您的电子邮件地址是 XXXXXX@XXXXXX.com，因为稍后我们把京东电子礼品卡发送到这个地址。
