# Supplementary material for: A grounded theory approach to understanding in-game goods purchase
Source: PLoS One. 2022 Jan 27;17(1):e0262998. doi: 10.1371/journal.pone.0262998 (PMC8794092; doi:10.1371/journal.pone.0262998)
Supplement: S1 File — (ZIP) [file pone.0262998.s001.zip › Transcript 2.pdf]

Interview: 002

Informant: 002

Please note that the original transcript is in Simplified Chinese. The English translation is for internal communication among the author of this research, and it is not proofread. Potential linguistic errors may exist in the English translation.

Researcher 14:51:59
Thank you for your willingness to participate and be interviewed here. My name is XXX XXX, and I'm a PhD student in the XXX University of XXX(XXX). Currently, I'm working on a research project which focuses on videogame players' purchase motivations of in-game goods. Throughout this interview, I will ask you a series of questions and you are encouraged to express your opinions freely with emoticons. If I have questions about what you've said or need clarification about a topic or concept, I'll ask you.
感谢您愿意参加并在此接受采访。 我叫 XXX，我是市场营销学的博士生，现在我在 XXX 大学就读。 目前，我正在开展一个研究项目，专注于电子游戏玩家对游戏内购买项目的购买动机。 在整个访谈中，我会问您一系列问题，我们鼓励您自由表达您的意见和观点。因为这不是一个当面访谈，所以我们也鼓励您用 QQ 表情来表达您的情绪。 在访谈过程中，如果我对你所说的内容有疑问或需要您澄清一个主题或概念，我会问您。

Researcher 14:52:08
Are you ready?
您准备好了吗？

Informant 002 14:52:12
Yes.
嗯

Researcher 14:52:52
In the previous survey, you mentioned that you purchased certain types of in-game purchases. What is your motivations for purchasing a Cosmetic/Skins in-game purchase?
在之前的调查问卷中，您已经提到您购买了某些类型的游戏内购买项目。您购买装饰/皮肤类游戏内购买项目的动机是什么？

Informant 002 14:53:06
There are promotions
有优惠活动

Informant 002 14:53:08
Good looking.
好看

Researcher 14:53:26
Can you share more details?
能再说得详细一点吗？

Informant 002 14:53:30
Commonly used heroes. I bought them to pleasure myself.
常用的英雄，买一个悦人悦己

Informant 002 14:53:41
Well, let me open the computer.
嗯 等我开电脑吧

Informant 002 14:53:45
(Please wait for) 2 minutes.
2 分钟

Researcher 14:53:51
Let’s talk on computer, which is faster.
开电脑说吧，快一些

Informant 002 14:55:27
I’m coming
来了

Researcher 14:55:31
Ok.
好的

Informant 002 14:55:37
Recently, I have purchased manys skins in the Wangzhe Rongyao.
最近购买的都是王者荣耀的皮肤

Informant 002 14:55:57
For the type of games like Wangzhe Rongyao, you will use some preferred heroes.
王者荣耀，这种类型的游戏，有些你会用的比较多的有偏好的英雄

Informant 002 14:56:19
Each hero has a lot of different skins, with the different price. However, the overall ability maintains the same.
每个英雄会有很多款不同的皮肤，价格不一样，但是总体增加的能力是一样的

Informant 002 14:56:38
When the promotion campaign is being launched, there will be some discounts for skins for my commonly used favorite heroes. If I still haven't had the skin of these heroes...

正好搞市场活动，有皮肤打折+常用的喜欢的英雄+这个英雄我还没有皮肤

Informant 002 14:56:40
I will just buy them.
就买了

Researcher 14:57:06
In other words, these in-game goods do not change the hero's ability values, and they only change the appearance. Is this the case?
也就是说，这些内购并不改变英雄的各项能力数值，只改变外观，是这样吗？

Informant 002 14:57:36
Just change a little bit.
Basically, nothing will be changed. The ability will be enhanced up to 5%.
只改变 1 点点
基本不改变，增强最多 5%吧

Informant 002 14:57:46
The enhancement of ability is incidental. They mainly change the appearance.
增强只能附带的，主要改变外观

Informant 002 14:58:14
I didn’t buy them for enhancing the ability.
我不是为了增强去买的

Informant 002 14:58:20
One more point.
哦 还有一点

Researcher 14:58:23
Ok. Just now we said the word "good-looking". What does it mean?
好的。刚才我们说到“好看”这个词，具体是什么意思呢？

Informant 002 14:58:32
This is to say. Many people own the skins for their favorite heroes.
就是基本大多数人，常用的英雄都会有皮肤

Informant 002 14:58:40
A kind of mental state like "a few people owe the skins, and I also want them".
有一点人家也有，我也想要的心理

Informant 002 14:58:53
The special design, not the standard appearance.
就是特殊的设计，不是 standard 的外观

Informant 002 14:58:58
Different UI and dubbing
不同的 UI 和配音

Researcher 14:59:16
In other words, when you see someone else has some skins, you also want them. Is that true?
也就是说，您在看到别人有某些皮肤的时候，自己也就想要了，是这样吗？

Informant 002 14:59:30
Yes, but the real trigger is the discount.
有的，但是真正的 trigger 是打折

Informant 002 14:59:36
Sometimes I always want them, but I haven't bought them.
一直有想要，但是没买

Informant 002 15:00:05
Because I know that the skins will be discounted from time to time.
因为我知道时不时会打折

Informant 002 15:00:10
So I wait.
所以在等

Researcher 15:00:26
If there is no discounts, will you consider to buy skins?
如果没有打折的时候您还会考虑购买皮肤吗？

Informant 002 15:00:32
I didn’t.
我自己没买过

Informant 002 15:00:40
There is only one possibility.
只有 1 种可能

Informant 002 15:00:44
It is: this skin has never been discounted,
要么从来不打折

Informant 002 15:00:50
If the skins have been discounted before, I will wait for promotion period to buy them.

如果有打过折，我就会等到打折的时候买

Researcher 15:01:50
Ok. We have already talked about the topic of the purchase process. I want to know how you usually buy a skin type in-game goods? Please tell me a general process.
好的。我们刚才已经谈到了购买流程的话题了。我想知道您通常怎么样购买皮肤类的内购？　请告诉我一个一般流程。

Informant 002 15:02:40
Click to buy - pop up payment page to confirm the payment amount - call WeChat payment.
点购买--弹出付款页面确认付款金额--呼起微信支付

Researcher 15:03:18
Is this process done outside the game or in the game?
这个过程是在游戏外进行的还是在游戏内进行的呢？

Informant 002 15:03:33
Before the WeChat is called, the process is inside the game.
呼起微信支付之前是游戏内的

Researcher 15:04:23
Ok.
好的。

Researcher 15:04:55
During the purchase of in-game items, do you frequently search for other information about the skin type in-game goods ? For example, do you learn more from your friends or from the official website of the game?
在游戏内商品的购买过程中，您是否经常搜索皮肤类游戏内商品的其他信息？例如，向您的朋友或在游戏的官方网站了解更多信息？

Informant 002 15:05:35
Respect to this question,
你这个问题

Informant 002 15:05:37
I read 3 times.
我看了 3 遍

Informant 002 15:05:41
What do you want to ask?
你想问什么

Informant 002 15:05:47
Searching before purchasing?
买之前是不是会搜搜看？

Researcher 15:05:56
Yes, that's what it means.
对，就是这个意思

Informant 002 15:06:00
No.
不会

Informant 002 15:06:12
Sometimes I check the pushing information. If I had not seen it, I would not have known the introduction of new skins.
看到推送了看一眼，没看到推送我也不知道会有新皮肤出来

Informant 002 15:06:17
I will not take the initiative to search.
也不会去主动去搜索

Informant 002 15:06:26
Because the skin itself is optional.
因为皮肤本身可有可无

Informant 002 15:06:36
The same for heroes, as you said, not the ability hasn't been enhanced.
英雄一样用，就像你说的，不是能力增强

Researcher 15:06:49
Ok, that is to say. When you are learning about a new skin, you mainly rely on the information pushing in the game, right?
好的，也就是说。你在了解一个新皮肤的时候，主要靠游戏内的信息推送对吗？

Researcher 15:07:05
If they don't push, you don't know. Is is correct?
如果他们不推送的话，您就不知道，可以这样说吗？

Informant 002 15:07:05
Information pushed by In-game/official account of the game in WeChat.
游戏内/个别的游戏微信号推送

Informant 002 15:07:17
Inaccurate

不准确

Informant 002 15:07:25
If the new skins are introduced, the information will be pushed a lot inside the game.
游戏更新了新皮肤，游戏内是会大量推送的

Researcher 15:07:27
Well, please add it.
嗯，请您补充一下

Informant 002 15:07:40
Sometimes when you don't play, you can't see the Pop-up push in the game.
但是有的时候不玩的时候，你就看不到游戏内的弹窗推送

Informant 002 15:07:47
However, I still will review some WeChat official accounts of the games.
但是还是会看一些游戏公众号

Informant 002 15:07:53
If they do not push me the notifications.
如果这些游戏工作号也不推送

Informant 002 15:07:55
Then I will never know.
我就不知道了

Researcher 15:08:40
Ok, that is to say, there are two main channels for you to acquire the information of these products: in-game information pushing and out-game information pushing, right?
好的，也就是说，您了解这些商品信息的渠道主要有两个：游戏内的推送和游戏外的推送，是吗？

Informant 002 15:08:45
Yes.
嗯

Researcher 15:08:50
Out-game channel mainly relies on WeChat, right?
游戏外主要是微信，对吗？

Researcher 15:08:56
WeChat official accounts.
微信公共号

Informant 002 15:09:24
Yes
嗯

Informant 002 15:09:58
One more point to be added.
还可以补充一点

Researcher 15:10:03
Please continue.
您说

Informant 002 15:10:04
I watch videogame live.
我会看游戏直播

Informant 002 15:10:08
I will see them during the live.
直播的时候，会看到

Informant 002 15:10:26
I would feel: “Ay? It looks pretty good.”
会“艾？挺好看的艾”

Informant 002 15:10:28
Something like this.
类似这样的

Researcher 15:12:11
Ok. I would like to ask: The WeChat payment has just mentioned, the money is paid to buy the skin directly, or or is paid to recharge the in-game currency,which serves to buy skins?
好的。我想问一下，刚才说到的微信支付，支付的钱是直接购买皮肤还是充值成游戏内货币，通过游戏内货币再购买皮肤？

Informant 002 15:12:35
Recharge to purchase directly.
直接充值购买

Informant 002 15:12:48
Aaa…
哦

Informant 002 15:12:49

No.
不对

Informant 002 15:12:53
It is recharged into the in-game currency.
是充值成游戏内货币

Informant 002 15:13:08
Because I always recharge the amount of money that I need, which makes no difference for me.
因为我都是多少钱，就冲多少，所以对我来说没区别

Researcher 15:13:34
Ok. The skins you have mentioned can be purchased through in-game mechanisms or they must be purchased by recharging real money?
好的。您说的这些皮肤能通过游戏内的机制购买吗?还是必须通过充值真钱来购买？

Informant 002 15:13:57
No.
不能

Informant 002 15:14:01
Only through RMB.
只能通过 RMB

Researcher 15:15:02
Ok. For you, after purchasing the skins, do you think the playability has been increased?
好的。对于您来说，购买皮肤后，您会觉得游戏性增加了吗？

Informant 002 15:15:28
Well, it will increase the gaming time in order to use the new skin.
嗯 会为了用用新皮肤而增加游戏时间

Researcher 15:16:26
It's interesting. Can you tell me some details?
这很有趣。能说一下细节吗？

Researcher 15:16:43
Why purchasing new skins can increase your gaming time?
为什么购买新皮肤能增加您的游戏时间？

Informant 002 15:17:26
Because the UI is different, the dubbing is different, and the action is different.

因为 UI 不同，配音不同，动作不同

Informant 002 15:17:41
The hand feeling is different. Moreover,…
用来手感不一样，另外

Informant 002 15:17:54
I have already paid money for them. I want to experience them more frequently, otherwise, the money is wasted.
会为了花了钱，而多体验体验，不然白花了

Researcher 15:19:59
Ok, how do you understand the word "playability"?
好的，请问一下您怎么理解"游戏性"这个词？

Informant 002 15:20:24
That is…
就是

Informant 002 15:20:41
Whether it takes up your time.
会不会吸住你的时间

Researcher 15:21:16
That is, the longer you play in the game, the higher the playability of this game is for you, is that the case?
也就是在游戏里游玩的时间越久，对您来说这个游戏的游戏性就越高，是这样吗？

Informant 002 15:21:31
Yes.
嗯

Researcher 15:22:25
Ok. In addition to the "Wangzhe Rongyao" game, have you purchased in-game goods in other games?
好的。您除了“王者荣耀”这款游戏外，还在其他游戏内购买过内购吗?

Informant 002 15:22:41
Yes.
有啊

Informant 002 15:22:48
Just spent 60 yuan to buy a horse
刚花了 60 买了匹马

Informant 002 15:22:58
It is a game, which is similar to the martial artes type game.
是一个，类似武林群侠传的游戏

Researcher 15:23:05
In which game?
在哪款游戏?

Informant 002 15:23:24
For running in the map, the horse is needed to speed up the process. RMB is the only way to purchase speed up items.
要跑图，有马加移速，只能 RMB 购买增加移速的道具

Informant 002 15:23:37
It is a small game: Baozou Yingxiong Tan.
是个小游戏，暴走英雄坛

Informant 002 15:23:46
A mobile game on Android.
安卓手游

Researcher 15:24:23
Ok. In other words, this horse does not only have the effect of improving the appearance, but also the specific functional use. Is that right?
好的。也就是说，这个马不单单有外观提升的作用，还有具体的功能性的用途，是这样吗？

Informant 002 15:24:33
Yes.
嗯

Researcher 15:25:05
Is the motivation for buying this horse different from the skin you just mentioned?
您买这个马的动机和刚才说的皮肤有区别吗？

Informant 002 15:25:14
Yes.
有

Researcher 15:25:23
I'm listening.
洗耳恭听

Informant 002 15:25:25
The skin is mainly a change in appearance, and the horse is mainly an enhancement of the attributes.
皮肤主要是外观的改变，马主要是属性的增强

Informant 002 15:25:27
It runs fast.
跑的快

Informant 002 15:25:35
Speed up the gaming progress.
加快游戏进度

Researcher 15:26:05
Why it is needed to speed up the gaming progress?
为什么要加快游戏进度呢？

Informant 002 15:26:40
This is an exploration game
这是一个探索类游戏

Informant 002 15:26:49
The deeper you explore, the stronger you will be
探索的程度越深，你就越强

Informant 002 15:26:58
Horse, can speed up the progress of exploration
马，可以加快探索的进度

Researcher 15:27:40
In other words, without this horse, the game will become more boring?
也就是说，没有这个马，游戏会变得比较无聊？

Informant 002 15:27:52
It will become slower.
会变得比较慢

Informant 002 15:28:10
For example, from A place to B place, you need 1 minute to reach the destination, but with hourse it takes 30 seconds.
本来从 A 到 B，你需要 1 分钟，有了马比如 30S

Informant 002 15:28:18

Increase game efficiency per unit of time.
增加单位时间的游戏效率

Informant 002 15:28:32
I feel that in different types of games,
感觉就是 不同类型的游戏

Informant 002 15:28:40
the needs of purchasing are different.
会有不同的购买的需求

Informant 002 15:29:05
Baozou Yingxiong Tan is more like a stand-alone game.
这个暴走英雄坛，偏单机

Researcher 15:30:10
I see. How do you know the product information of this horse? Is it also through the in-game information pushing, WeChat information pushing and live broadcast as you just mentioned?
原来如此。您怎么了解到这个马的产品信息的？也是通过刚才说的游戏内推送，微信推送和直播三类方式吗？

Informant 002 15:30:30
Only in-game pushing.
只有游戏内推送

Researcher 15:31:15
Ok, then after learning about the existence of this product, did you still have acquired more information from other sources?
好的，那么在了解到这个产品的存在后，您还有没有从其它渠道去了解更多的信息？

Researcher 15:31:24
Apart from that in-game pushing.
除了那条游戏内推送

Informant 002 15:31:24
Yes.
有

Researcher 15:31:31
For example?
比如说？

Informant 002 15:31:37
I searched it using Baidu to see wether there are other items which have the same speeding up effects but do not need to be purchased using RMB.
我百度了一下，有没有其他增加移动速度的道具，可以不用 RMB 买的

Informant 002 15:31:42
I didn't find that, so I bought the horse.
发现没有，就买了
Informant 002 15:31:53
Oh, you said that the acknowledge of this item.
哦，你说了解到这个道具啊

Informant 002 15:31:55
There is no other channel.
没有其他渠道

Researcher 15:32:20
In other words, you will evaluate whether you can use the items to achieve the same effect. Is that true?
也就是说您会评估是否可以用免费的道具来达成相同的效果，是这样吗？

Informant 002 15:32:30
Yes.
是的

Researcher 15:33:37
Backing to the previous theme, will you make the similar assessment for the skin type items also? For example, using the free items to achieve the same effects.
话说回来，对于刚才我们谈到的皮肤道具，您也会进行类似的评估吗？比如想用免费的道具来达成相同的效果。

Informant 002 15:34:11
Yes.
会

Researcher 15:34:25
Do you also use Baidu to search for that?
也是通过百度去搜索吗？

Informant 002 15:34:26
There is a channel, which is especially difficult...
有这个渠道，特别的难

Informant 002 15:34:47

I didn't search for that, but somehow, I knew.
那个倒是没搜过，因为 SOMEHOW 我就知道

Researcher 15:35:04
What is the difficult point?
难的点在哪里？

Informant 002 15:35:35
Two difficult points. 1. Redeeming the skin type items requires a long time to accumulate, and those look prettier require more time accumulation.
2 个难点，1 是兑换皮肤需要的道具需要很长时间的积累，越好看的需要积累的时间越长

Informant 002 15:35:47
2. They cannot be redeemed anytime, because there is a window period.
2.不是随时随地都能兑换，有窗口期

Researcher 15:36:04
So you almost never acquire the skin through the in-game mechanism, right?
所以您就几乎从来不通过游戏内的机制来获取皮肤，对吧？

Informant 002 15:36:56
I also obtain them.
也获得的

Informant 002 15:36:58
Incidentally.
顺便获得

Researcher 15:37:15
Occasionally? right
偶尔？对吗

Informant 002 15:37:27
Yes.
嗯

Informant 002 15:37:37
Because the mechanism determines that you only can get them occasionally.
因为机制就决定了，你偶尔才能获得

Researcher 15:37:46
Right.
原来如此

Researcher 15:37:48
Another question. For the two types of items that you have just mentioned, they are permanent items? or time-limited items?
另外一个问题。对于您刚才谈到的两种道具，它们都是永久道具吗？还是有时间限制的道具

Informant 002 15:38:31
Permanent.
永久

Researcher 15:39:45
Ok. We just mentioned two different types of in-game products: skin and functional items. When you buy in-game goods, do you have a priority in mind? For example, would you give priority to buying some types of product to a other types of product?
好的。我们刚才提到了两种不同类型的游戏内商品：皮肤类和功能类道具。 当您购买游戏内商品时，您是否心里有一个优先顺序。比如比起一类游戏内商品您会优先购买另一类商品？

Informant 002 15:40:06
No.
不会

Informant 002 15:40:09
They are completely two different games.
完全不同的 2 个游戏

Researcher 15:40:36
What if there are two types of goods in the same game?
如果在同一游戏内存在这两类商品呢？

Researcher 15:41:00
Or have you never had such experience?
或者说您从来没有这样的经验？

Informant 002 15:41:36
Let me think…
我想想

Researcher 15:41:40
Humm..
恩恩

Informant 002 15:41:47、

It seems that there is no both A and B.
好像不存在既有 A，又有 B 的

Informant 002 15:41:54
Intersting
Interesting

Informant 002 15:42:25
That is, if there is pure skin type…
就是说，如果有纯皮肤类的

Informant 002 15:42:34
Let’s say like this.
这样说吧

Informant 002 15:42:44
If there is, I will give the priority to those items which can only obtained by RMB.
如果有，我会优先选择只能用 RMB 买的

Informant 002 15:43:38
Then, there is no case that the skin and enhancement types items which are separated.
然后，并不存在皮肤类和道具增强类拆分开的

Informant 002 15:43:42
Most of them are merged
大部分是合并的

Informant 002 15:44:10
A few Power-ups items can be obtained through RMB or inside the game.
少数增强类的，是可以 RMB 获得，或者游戏内获得的

Informant 002 15:44:15
That is…separated.
就是，拆开的

Researcher 15:44:30
In other words, in most games you have played, skin types items come with some power-up effects. Is that true?
也就是说，您玩的大部分游戏中，皮肤类道具都是自带一些增强效果的，是这样吗？

Informant 002 15:44:38
Yes.
嗯

Researcher 15:45:23

These are all the questions. Thank you very much for participating in our research. Please confirm that your email address is XXXXXX@XXXXXX.com, because later we will send the JD electronic gift card to this address.

这就是全部的问题。 非常感谢您参与我们的研究。请确认您的电子邮件地址是XXXXXX@XXXXXX.com，因为稍后我们把京东电子礼品卡发送到这个地址。
