# Supplementary material for: A grounded theory approach to understanding in-game goods purchase
Source: PLoS One. 2022 Jan 27;17(1):e0262998. doi: 10.1371/journal.pone.0262998 (PMC8794092; doi:10.1371/journal.pone.0262998)
Supplement: S1 File — (ZIP) [file pone.0262998.s001.zip › Transcript 20.pdf]

Interview: 020

Informant: 007

Please note that the original transcript is in Simplified Chinese. The English translation is for internal communication among the author of this research, and it is not proofread. Potential linguistic errors may exist in the English translation.

Researcher 8:31:37
Thank you for your willingness to participate and be interviewed here. My name is XXX XXX, and I'm a PhD student in the XXX University of XXX(XXX). Currently, I'm working on a research project which focuses on videogame players' purchase motivations of in-game goods. Throughout this interview, I will ask you a series of questions and you are encouraged to express your opinions freely with emoticons. If I have questions about what you've said or need clarification about a topic or concept, I'll ask you.
感谢您愿意参加并在此接受采访。 我叫 XXX，我是市场营销学的博士生，现在我在 XXX 大学就读。 目前，我正在开展一个研究项目，专注于电子游戏玩家对游戏内购买项目的购买动机。 在整个访谈中，我会问您一系列问题，我们鼓励您自由表达您的意见和观点。因为这不是一个当面访谈，所以我们也鼓励您用 QQ 表情来表达您的情绪。 在访谈过程中，如果我对你所说的内容有疑问或需要您澄清一个主题或概念，我会问您。

Researcher 8:31:41
Are you ready now?
您准备好了吗？

Informant 007 8:31:45
Yes
嗯

Researcher 8:31:58
Let's talk about the skin type in-game goods. You mentioned in our first interview "Of course, for the sake of handsome.", "In fact, it's just for good looking."
我们现在来谈一下皮肤类型的游戏内购。您在第一次访谈的时候说到"当然是为了帅啊"，"其实就是为了好看吧"，"因为农药这个就一直在操作，有个视觉效果体验，放个技能什么的都跟皮肤有关"

Researcher 8:32:03
Do you think people around you (offline or online) have an impact on your purchase of in-game goods?
您认为您周围的人（线下或线上）对您购买游戏内商品有影响吗？

Informant 007 8:32:24
Yes.

有的

Researcher 8:32:32
How do people around you influence your purchase of in-game goods? Could you give me some examples?
请问您周围的人如何影响您购买游戏内商品？ 您能给我一些例子吗？

Informant 007 8:33:25
(After) someone buys a new skin, (he/she would mention) how special is the effect. After these blablabla, I would subconsciously check the (product) when I'm playing the game.
XXX 买了新的皮肤 这特效 这造型 巴拉巴拉一推之后 自己玩的时候肯定会下意识的去看看

Informant 007 8:33:34
If I fail to resist the temptation, I would purchase the (item).
没忍住的话 就会买下了

Researcher 8:33:57
Ok. Under what circumstance does the conversation usually happen?
嗯嗯，一般这个对话是在什么场景下发生的？

Informant 007 8:34:07
Because in many cases you can't always play the game alone.
因为很多情况下 你不可能一直一个人玩单机游戏的

Informant 007 8:34:26
In most of the online games, people play together. There are interactions.
大多数网络游戏 是大家一起玩 互动的

Informant 007 8:34:54
As a result, (I) cannot stand the encouragement from my colleagues or friends, hahaha.
所以 会经不起被同事或朋友 怂恿的 哈哈哈

Researcher 8:35:01
So this happens in the chatting channels in the game, right?
也就是在游戏的聊天频道里进行这些交流，对吗？

Informant 007 8:35:25
There are many cases, such as face-to-face communications among colleagues, there are also communications on WeChat.
这个就比较多了 比如同事之间面对面也有 微信语音之间交流 也有

Informant 007 8:35:39

Yes, (we) communicate inside the game as well.
是的 游戏内交流也有

Researcher 8:36:51
I see, what kind of people are those who talk these words with you?
原来如此，一般周围和您说这些话的人都是什么样的人呢？
Researcher 8:37:00
Friends in the real life?
现实中的朋友吗？

Informant 007 8:37:16
Yes, they are friends in the real life if the (communication) is realised face-to-face.
嗯 面对面的都是现实中的朋友

Researcher 8:37:27
Have you ever joined the league in online games?
您在网络游戏内加入过工会吗？

Informant 007 8:37:42
Yes.
有

Researcher 8:38:06
Will the members of the league affect the decision-making of your in-game purchase?
请问工会里的成员会对您游戏内购的决策有影响吗？

Informant 007 8:39:09
I feel that the final decision is in my own hands. The league members in the game would only help me to do some analyses: The difference between purchase or not.
我觉得噢 最终决定买与不买 决策权还是在自己手中的 而游戏内工会成员只会帮你分析 买与不买之间的区别

Informant 007 8:39:24
There are more or less influence.
少许还是有那么一些影响的

Informant 007 8:40:27
Let's just say that the league members who have bought the in-game goods. After they tell me the pros and cons of the purchase, it would greatly affect my purchase decision.
这么说吧 工会成员内已经买过内购的人 告诉你买与不买之间的利弊后 这倒会极大的影响你购买的决策

Researcher 8:40:45
I see, we just talked about the impact of others via the word of mouth. Is there any non-

linguistic influence in the purchase process?
原来如此，我们刚才谈到了别人对您口口相传的影响。在这个购买过程中存不存在非语言类的影响呢？

Researcher 8:41:06
Including other players' behaviours or your purchase of in-game goods.
包括游戏内其它玩家的行为对您购买内购的影响。

Informant 007 8:42:00
If we only discuss the skin type in-game goods. There is the influence of words. Buying skin is for good looking. Of course, showing off in front of others.
单论皮肤内购的话　大多数还是存在语言类的影响　　买皮肤就是为了好看　当然要跟别人炫耀啊

Informant 007 8:42:11
If there is no one to show off, why should I purchase skins? They are just a bunch of data. Hahaha.
没人炫耀买了干嘛呀　就一堆数据　　哈哈哈

Researcher 8:43:04
Ok. That is, on the one hand, you feel that the skin looks good, on the other hand, you want to show off in front of people, right?
好的。也就是一方面自己觉得皮肤好看，另一方面也是为了在人前炫耀，对吗?

Informant 007 8:43:24
Hahaha yes.
哈哈哈　是的

Informant 007 8:44:23
If the skill is bad, then skins make up the thing.
技术不行　皮肤来凑

Researcher 8:44:32
Conversely, if someone else has a skin that you don't have, and show off in front of you. Does this have an impact on your in-game purchase decisions?
反过来说，如果别人有一个您没有的皮肤，并且在您面前炫耀。这会对您的游戏内购决策有影响吗？

Informant 007 8:45:04
Yes, people are jealous. If I don't have that, I certainly would be envious.
嗯　　人都有嫉妒心的嘛　自己没有的　当然是羡慕的

Informant 007 8:45:45
I would consider from my own needs. Do I often play this hero? Do I have the skill to

play it well? Does the shape of the skin conform to my aesthetic viewpoint?
从自身的需求考虑 这个英雄我经常玩吗？我玩得溜吗？皮肤的造型符合我的审美吗？

Informant 007 8:46:18
After self-struggling, I would decide whether to buy or not.
经过自我挣扎之后 就会决定买与不买了呗

Researcher 8:46:50
I see. In other words, after having the envious emotion, you would still make a rational consideration to decide whether or not to buy?
原来如此。也就是说，在羡慕的情绪后，您依然会进行一个理性的思考，来决定是否购买？

Informant 007 8:47:20
Yes.
嗯

Researcher 8:47:57
But does this emotion affect your rational thinking?
但这种情绪会对您的理性思考造成影响吗？

Informant 007 8:48:29
More or less.
会有一些的

Informant 007 8:49:26
The rational thinking tells me that not to buy, because they are just a bunch of data. Moreover, the envious emotion tells me to buy it and I'm not going to be fooled.
理性告诉我 不要买 只是一堆数据而已 嫉妒的情绪会告诉我 不买白不买买不了吃亏买不了上当

Researcher 8:50:12
Ah, I see. It's said that your envious emotion would l interfere with your rational thinking, right?
啊原来如此。就是嫉妒的情绪会干扰您的理性思考，对吗？

Informant 007 8:50:21
Yes.
是呀

Researcher 8:50:47
Ok. You just talked about "If the skill is bad, then skins make up the thing." How do I interpret this sentence?

好的。刚才您谈到"技术不行　皮肤来凑　"，请问我怎么理解这句话？

Informant 007 8:51:27
The skill is too bad, which makes impossible to show up. So, skins are the only way to show off.
技术太差 炫耀不起来　只能靠皮肤来炫耀了

Researcher 8:52:07
I see.
原来如此。

Researcher 8:52:24
Have you purchased the loot boxes in-game goods in the last 6 months?
请问您最近 6 个月内有购买过抽奖箱类型的游戏内购吗？

Informant 007 8:53:01
Ah, to tell you the truth, no.
啊　如实回答的话　没有

Informant 007 8:53:10
I couldn't find many games to play in the last 6 months.
最近 6 个月都游戏荒

Researcher 8:53:59
Ok.
好的 。

Researcher 8:55:43
In our last interview, you talked about having the flow experience is your purpose of playing games. In this interview, you just mentioned buying skin in order to show off. Is socialising also the purpose of your gaming?
我们在上一次访谈中，您谈到了获得心流体验是您玩游戏的目的。在这次访谈中，您刚才也提到了购买皮肤为了炫耀。请问社交是不是也是您玩游戏的目的呢？

Informant 007 8:56:10
Yes.
是的

Informant 007 8:56:37
I remember that when I first entered the society, in my first job, I was playing the same game with the people around me.
记得那时候刚刚踏入社会　第一份工作的时候　就是和周围的人一起玩同一款游戏　才慢慢熟悉起来的

Researcher 8:57:07
People around you refers to your colleagues?
周围的人是第一份工作的同事吗？

Informant 007 8:57:14
Yes.
对的

Researcher 8:57:35
What is the name of that game?
请问那款游戏的名称是什么？

Informant 007 8:57:47
LOL League of Legends
LOL　英雄联盟

Researcher 8:58:57
I see. Have you ever spent any money in that game?
原来如此。您在那款游戏中进行过消费吗？

Informant 007 8:59:58
Yes, I have.
嗯　有过

Researcher 9:00:34
Did the surrounding colleagues have an influence on your in-game purchasing at that time? If you still remember.
请问那时候周围的同事对您游戏内购物有影响吗？如果您还记得的话。

Informant 007 9:01:22
Yes.
有的

Informant 007 9:01:46
When we had lunch at noon, and when we were free in the afternoon, everyone people would discuss.
中午吃饭的时候　下午闲暇的时候　大家都会讨论

Informant 007 9:02:06
At that time, if rare skins were on sale, people would discuss.
那时候会出限定的稀有皮肤　都会讨论的

Researcher 9:02:47
I see.

原来如此。

Researcher 9:03:25
We shall continue. Have you purchased skins in stand-alone games?
我们继续。您有在单机游戏内购买过皮肤吗？

Informant 007 9:03:38
No, I haven’t.
没有过

Informant 007 9:04:15
In most stand-alone games, skin type in-game goods cannot be found.
大多数单机游戏　也不会推出皮肤这种内购的

Researcher 9:04:52
Ok. I see.
原来如此，我明白了。

Researcher 9:05:15
Now, let’s review the contents of the last interview.
现在让我们回顾下上次访谈的内容。

Researcher 9:05:23
We mentioned the concept of "Flow experience" last time. Let's review this concept.
“Flow experience” has been used by psychologist to describe a state of mind experienced by people who are deeply involved in an activity. Instance, sometimes while playing videogames, the player’s action and awareness are merged, and he/she is totally connected on the gaming tasks at hand. In this state, the player loses his/her consciousness, and his/her perception of time becomes faster or slower than usual. Also, the player perceives a feeling of being in control, which empowers him/her from the fear of failure.
我们上一次提到了”心流体验“这个概念。我们来回顾一下这个概念
“心理学家使用“心流体验”来描述深度参与某项活动的人所经历的心理状态。 例如，有时玩家在玩电子游戏时，他/她的动作和意识会融为一体，并且他/她完全关注手头的游戏任务。 在这种状态下，玩家失去他/她的自我意识，他/她对时间的感知变得比平时更快或更慢。 此外，玩家会感受到一种掌控全局的感觉，这使他/她免于对失败的恐惧。”

Researcher 9:05:35
Please tell me how do you end this experience after you have gained it? Which events led to the end of this experience?
请问您在获得心流体验之后，这个体验一般是怎么结束的？哪些事件导致了这种体验的结束？

Informant 007 9:06:27
Generally, it ends with the end of the game content.
一般也就是随着游戏内容结束而结束吧

Researcher 9:06:54
After clearing the game, the flow experience ends, right?
就是游戏通关了，心流体验也就结束了，是吗？

Informant 007 9:06:55
Taking LOL and DOTA as examples, after ending a round of competition, the experience is almost ended.
比如 LOL DOTA 一把比赛结束的同时体验也差不多结束了

Researcher 9:07:05
Ah, I see.
啊原来如此。

Informant 007 9:07:08
More or less like this.
差不多吧 是这么个意思

Researcher 9:07:30
I understand.
我明白了。

Researcher 9:07:36
Are there any other events?
还有没有其它的情况呢？

Informant 007 9:08:37
There are the cases that the flow experience is ended by the outside forces.
受外界的影响 结束心流体验的也会有

Informant 007 9:08:58
Mom and Dad call me for some urgent things, and they need my help.
爸妈喊你有什么急事需要你帮忙

Informant 007 9:09:01
Like this kind of things.
这种的也会有

Informant 007 9:09:04
But they are occasional

但都是偶尔的

Researcher 9:09:51
I see.
原来如此。

Researcher 9:10:00
We talked about the "anxious experience" last time. You mentioned “I have! Very anxious! Broken heart!”,” When playing DOTA2...It's really hard to say.”, “It usually happens in the moba type games. When I can't communicate well with my teammates, I will feel very anxious.”. Do you think that the end of the flow experience is related to the anxious experience?
我们上次谈到了“焦虑的体验”，您提到“ 有！非常焦虑！操碎了心！”，“打 DOTA2 呀　真的是一言难尽啊”，“一般发生在这种 moba 类游戏中，与队友交流不通，会觉得很焦虑。”。您认为心流体验的终止和焦虑的体验有关系吗？

Informant 007 9:10:41
It doesn't matter. Anxiety is only generated during the flow experience, the ending of the heart flow experience doesn’t cause the anxious experience.
没有关系　　焦虑只是在心流体验中产生的　而不是心流体验终止造成的

Researcher 9:12:23
Well, I explain this point. We are not talking about the causal relationship between the end of flow experience and anxiety. It's the impact of an anxious experience on your flow experience.
嗯，我解释一下这个点。我们不是在谈心理体验的终止和焦虑的因果关系。而是焦虑的体验对您心流体验的影响。

Informant 007 9:12:55
Oh Oh, I understand, it affects.
哦哦　明白了　　会的

Researcher 9:13:13
Generally, how does the anxiety affect your flow experience?
一般焦虑的情绪会怎么影响您的心流体验呢？

Informant 007 9:13:19
I have done things like alt+F4 to quite the game…
直接 alt+F4　退出游戏的事情 我也做过。。。

Researcher 9:13:34
That is to directly suspend the game, right?
也就是直接暂停游戏了，对吧？

Informant 007 9:13:36
My mood was down, and I didn’t want to go on.
心情会比较差嘛　　不想再继续下去

Informant 007 9:13:47
If I kept going on, it only would make me feel worse.
继续下去　只会更加影响心情

Informant 007 9:13:51
It was better to quit directly
还不如直接退出

Researcher 9:14:00
Ok, I understand.
好的，我明白了。

Researcher 9:14:10
Moreover, We talked about the "boring experience" last time, you mentioned “When the difficulty of the game is very high, and when the gaming enters to a very obvious repeated loop.” Do you think that the end of the flow experience is related to the boring experience?

另外，我们上次谈到了“无聊的体验”，您提到“当游戏难度非常难的时候　还有就是游戏进入一种很显而易见的循环的时候”、您认为心流体验的终止和无聊的体验有关系吗？

Informant 007 9:14:53
Yes, I do.
也会有的

Researcher 9:15:07
Generally, how does the boring experience affect your flow experience?
一般无聊的感觉会怎么影响您的心流体验呢？

Informant 007 9:15:15
If I cannot perceive any gaming experience, I would feel very bored, and I cannot enter to the flow experience.
没有任何游玩体验了　感觉非常无聊了　也不会进入心流体验这种状态

Informant 007 9:15:37
Only boring lefts.
只剩下枯燥 无聊了

Researcher 9:15:56

Have you ever been bored after you have entered the flow experience?
有没有已经进入心流体验后，又觉得无聊的情况？

Informant 007 9:16:54
For example, LOL, after entering the flow experience, the upwind play would make me anxious and nervous, and the downwind play would make me complacent and boring.
比如说 LOL 这种的　进入心流体验后　逆风局会让你焦虑 紧张　顺风局会让你嘚瑟　并且无聊

Informant 007 9:17:01
These situations happen.
也是会有这种情况发生的

You sent a window jitter.
你发送了一个窗口抖动。

Researcher 9:17:19
Sorry for pressing the wrong one.
不好意思按错了

Informant 007 9:17:25
No problem.
诶关系

Informant 007 9:17:26
No problem.
没关系

Informant 007 9:17:40
It's almost like this. I don't know whether you could understand what I have said.
差不多是这么个意思　不知道我表达的 你能理解伐

Researcher 9:18:01
Yes, I understand.
嗯嗯，我明白的。

Researcher 9:18:44
Ok. I have almost done. The interview is almost over. Do you have any viewpoints to add?
ok。我问得差不多了，访谈差不多要结束了。您还有什么观点需要补充吗？

Informant 007 9:18:57
I have nothing to add.
我没有需要补充的

Informant 007 9:19:10
Do you need that I add something more?
你这边还有哪些需要我补充的吗？

Informant 007 9:19:17
I still have free time.
我这边还有空余时间呢

Researcher 9:19:47
Well, just a little bit, I want to know more about one point.
 嗯嗯，就一个小点我想再了解一下

Researcher 9:19:58
We just talked about the flow experience, anxiety, and boredom.
刚才我们谈到了心流体验，焦虑，和无聊。

Informant 007 9:19:58
Yes, please continue.
嗯　你说

Researcher 9:20:17
My understanding of what you just said is that these three states are mutually exclusive.
我对于您刚才说的话的理解是，这三种状态是互斥的

Researcher 9:20:36
Is there only one state at a time, is that the case?
就是在同一时间内只存在一种状态，是这样吗？

Informant 007 9:20:51
Yes, I think so.
是的　我认为是的

Informant 007 9:21:02
Anxiety and boredom can't exist at the same time.
焦虑和无聊　不可能同时存在啊

Researcher 9:21:40
We just talked about "I have done things like alt+F4 to quite the game...
"
我们刚才谈到"直接 alt+F4　退出游戏的事情 我也做过。。。"

Researcher 9:22:09
Can I interpret that at this time, it is already anxious experience, has the mood jumped

from the flow experience to the anxiety?
我能不能认为在这种时候，就已经是焦虑的情绪了，已经从心流体验跳出来到焦虑的心情了？

Informant 007 9:22:19
Yes.
是的

Researcher 9:23:13
It turns out that I think I have sorted things out clearly.
原来如此，我想我把事情都整理清楚了。

Researcher 9:23:21
These are all the questions. Thank you very much for participating in our research. Please confirm that your email address is XXXXXX@XXXXXX.com, because later we will send the JD electronic gift card to this address.
这就是全部的问题。 非常感谢您参与我们的研究。请确认您的电子邮件地址是XXXXXX@XXXXXX.com，因为稍后我们把京东电子礼品卡发送到这个地址。
