# Supplementary material for: A grounded theory approach to understanding in-game goods purchase
Source: PLoS One. 2022 Jan 27;17(1):e0262998. doi: 10.1371/journal.pone.0262998 (PMC8794092; doi:10.1371/journal.pone.0262998)
Supplement: S1 File — (ZIP) [file pone.0262998.s001.zip › Transcript 21.pdf]

Interview: 021

Informant: 014

Please note that the original transcript is in Simplified Chinese. The English translation is for internal communication among the author of this research, and it is not proofread. Potential linguistic errors may exist in the English translation.

Researcher 5:59:11
Thank you for your willingness to participate and be interviewed here. My name is XXX XXX, and I'm a PhD student in the XXX University of XXX(XXX). Currently, I'm working on a research project which focuses on videogame players' purchase motivations of in-game goods. Throughout this interview, I will ask you a series of questions and you are encouraged to express your opinions freely with emoticons. If I have questions about what you've said or need clarification about a topic or concept, I'll ask you.
感谢您愿意参加并在此接受采访。 我叫 XXX，我是市场营销学的博士生，现在我在 XXX 大学就读。 目前，我正在开展一个研究项目，专注于电子游戏玩家对游戏内购买项目的购买动机。 在整个访谈中，我会问您一系列问题，我们鼓励您自由表达您的意见和观点。因为这不是一个当面访谈，所以我们也鼓励您用 QQ 表情来表达您的情绪。 在访谈过程中，如果我对你所说的内容有疑问或需要您澄清一个主题或概念，我会问您。

Researcher 5:59:16
Are you ready now?
您准备好了吗？

Informant 014 5:59:21
Yes
嗯

Researcher 5:59:42
I have seen in the questionnaire you filled out that you have purchased different kinds of in-game goods within six months, including cosmetics/skins. What are your motivations for purchasing cosmetics/skins type game in-game goods?
我在您之前填写的问卷中有看到半年内您有购买过不同种类的游戏内购，其中包括装饰/皮肤。请问您购买装饰/皮肤类游戏内购的动机是哪些？

Informant 014 6:00:10
For good looking.
就好看

Informant 014 6:00:14
Needs of the game.

游戏需要

Researcher 6:00:38
How do I understand the second point? Can you give an example?
请问第二点我怎么理解呢？能举一个例子吗？

Informant 014 6:00:52
Be stronger.
变强大

Informant 014 6:01:07
There are properties.
有属性

Researcher 6:01:37
Um... that's a kind of skin that not only changes the appearance but also comes with attributes, right?
嗯...也就是一类不单单改变外观，并且附带属性的皮肤，对吗？

Informant 014 6:01:46
Yes.
是的

Researcher 6:02:03
Do you think people around you (offline or online) have an impact on your purchase of in-game goods?
好的。您认为您周围的人（线下或线上）对您购买游戏内商品有影响吗？

Informant 014 6:02:16
No.
没有

Researcher 6:02:40
Ok. Have you ever purchased skins in stand-alone games?
好的。您之前有在单机游戏中购买过皮肤吗?

Informant 014 6:02:51
I have not played stand-alone game.
没玩过单机

Researcher 6:04:08
Um um... I have seen that you play games on Android system, that is, until now, what you play only are limited in mobile online games, right?

嗯嗯...我之前有看到您在 Android 系统上玩游戏，也就是截止目前为止，您玩的都是手机网络游戏，对吗？

Informant 014 6:04:18
Yes.
是的

Researcher 6:05:38
Uh huh. I want to know more about the answer to the "For good looking" you just mentioned. Can you share some details with me? For example, when buying a good-looking skin, some psychological activities.
嗯嗯。我想再了解一下您刚才谈到的"就好看"这个回答。您能分享给我一些细节吗？比如在购买一个好看的皮肤的时候，心理上的一些活动。

Informant 014 6:07:13
I just think about that through having this skin, the attributes can be strengthened. Look looking. No other considerations.
就想着有这个皮肤给自己加强属性 外观好看 没有别的想法

Researcher 6:08:13
Uh huh. What we just talked about is the case of the skin with attributes. Have you ever purchased a skin without attributes? That is, the item that only changes the appearance and does not change the attribute.
嗯嗯。我们刚才谈到的都是带有属性的皮肤的状况，请问您之前有购买过不带属性的皮肤吗？也就是只改变外观，不改变属性的道具。

Informant 014 6:08:27
No
没有

Informant 014 6:08:47
The games that I play usually contain skins with attributes.

一般皮肤都带属性 我玩的游戏

Researcher 6:09:30
I see. When you buy such items, do you pay more attention to the appearance of the skin or the properties of the skin?

原来如此。请问您在购买这类道具的时候，是更关注皮肤的外观还是皮肤的属性呢?

Informant 014 6:09:48
Appearance, Hahaha, the attributes come secondary.
外观吧哈哈属性第二

Informant 014 6:10:24
But I generally consider these two aspects globally.
但是一般考虑两样结合的

Researcher 6:10:34
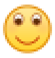I see.

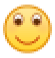原来如此。

Informant 014 6:11:43
Yes.
嗯

Researcher 6:12:01
After buying the skin, will you show it to your friends around you?
请问您在购买皮肤后，会有意地秀给周围的朋友看吗？

Informant 014 6:12:11
Yes Hahaha.
会哈哈哈

Informant 014 6:12:25
After they buy (the skins), they also show (them).
他们买了也会秀

Researcher 6:12:37
So, at this time, what is the reaction of others in general?
原来如此，在这个时候，一般别人的反应是怎么样的?

Informant 014 6:12:49
There are no actions.
没反应

Informant 014 6:12:51
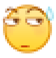

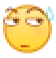

Researcher 6:13:39
Well, have you experienced the case that after you show the skins in front of them, they also purchase the similar skin?
嗯嗯，有没有在您秀给他们看了之后，他们购买了相似的皮肤的情况？

Informant 014 6:14:08
Without showing it, they would buy it anyway.
不用秀他们喜欢也会买的

Researcher 6:15:23
I see. In another case, what do you think if you see the friends around you buy new skin and show it to you?
原来如此。另外一种情况，如果您看到周围的小伙伴们购买了新皮肤，并且秀出来给您看，您会怎么想？

Informant 014 6:15:55
I feel very annoyed because they show it off, hahahaha
觉得很烦炫耀哈哈哈哈
Informant 014 6:15:59

Researcher 6:16:19
I sometimes think so, haha.

我有时候也会这么想，哈哈

Researcher 6:16:31
Ok, I see.
ok，好的。

Informant 014 6:16:34
Hahaha
哈哈哈

Researcher 6:16:57
In other words, there is a relatively negative emotion, right?
也就是说，会有一种比较负面的情绪，对吗？

Informant 014 6:17:14
Yes.
对的

Researcher 6:18:11
After this incident, will you still choose to buy the same skin? Or you would intentionally avoid to purchase this skin and instead go to buy other skins?
在发生这个事件后，您会选择去购买相同的皮肤吗？还是会有意避开这个皮肤而转而去购买其他外观的皮肤？

Informant 014 6:18:30
No.
不会的

Informant 014 6:18:39
I buy it if I like it.
自己喜欢就买

Researcher 6:19:14
In other words, if someone shows off in front of you, your would be annoyed, but it will not affect your purchasing decision. Can I interpret like this?
也就是说，别人在您面前秀，心里会比较烦躁，但并不会影响您的购买决策。我能这么理解吗？

Informant 014 6:19:32
Yes.
嗯

Researcher 6:19:56
Ok, let's move on to the next topic.
好的，我们进入下一个话题。

Researcher 6:20:04
What are the motivations for purchasing a loot boxes type in-game goods?
请问您购买抽奖箱类游戏内购的动机是哪些？

Informant 014 6:20:45
In case of a white face, I won the prize.
万一脸白中奖了呢

Researcher 6:21:48
I see. Can I understand this mentality as a gambling-like experience?
原来如此。我能不能把这个心态理解成一种类似赌博般的体验？

Informant 014 6:21:57
Yes, Hahaha.
对哈哈

Researcher 6:22:13
Ok.

好的。

Researcher 6:22:41
So, before the draw, your psychological expectation is you could get the items you want or not?
所以，在抽奖前，您的心理预期是抽得到您想要的物品还是抽不到？

Informant 014 6:23:05
It must be that I can get it, but actual it happens rarely.
肯定是抽到呀但是很少抽到过

Researcher 6:23:42
I see. I just think about that I can get it, but the result is always the opposite of what you think, is that the case?
原来如此。就是想着能抽到，但是结果和自己想的相反，是这样吗？

Informant 014 6:23:51
Yes.
是的

Researcher 6:24:25
At this time, what is your mood like?
在这种时候，您的情绪是怎么样的？

Informant 014 6:24:53
I don’t think about it anymore.
想着以后再也不弄了

Informant 014 6:24:55
Cut my hands.
剁手

Researcher 6:25:26
Hum...any other thoughts?
嗯嗯... 还有其它的想法吗？

Informant 014 6:26:37
I implicitly abuse the TiMi Studio.
暗骂天美工作室

Informant 014 6:26:39
Hahaha
哈哈哈

Researcher 6:27:16
I see. Which games produced by TiMi Studio do you usually play?
原来如此。您一般玩天美工作室的哪几款游戏？

Informant 014 6:27:47
Luanshiwangzhe
乱世王者

Informant 014 6:27:54
I play it with Baohuo, who plays it with me together.
把你推荐给我的宝货一起的

Researcher 6:28:49
Well, after the previous interview, I have more or less understanding of this game.
嗯嗯 经过之前的访谈，我对这款游戏多多少少有一些理解

Informant 014 6:28:55
The Karting is not of Timi, is it?
卡丁车不是天美的吧

Informant 014 6:29:10

嗯这个游戏特别吸钱

Researcher 6:29:14
What's the complete name of Karting?
卡丁车的全名是？

Informant 014 6:29:21
Popkart

跑跑卡丁车

Researcher 6:30:00
This one?
这一款？

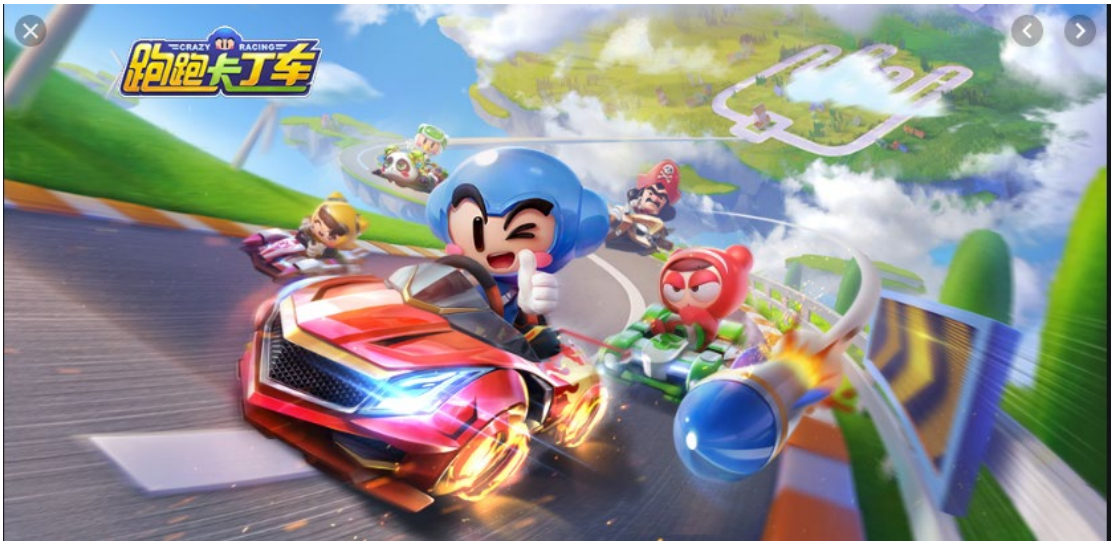

Researcher 6:30:46

It should be a game developed by NEXON and administrated by Tiancity .

应该是 NEXON 开发的，世纪天成代理的游戏

Informant 014 6:31:01
Yes, yes.
对对

Informant 014 6:31:11
I have impression about this after you mentioned that.
你这么说我有印象

Researcher 6:31:28
I see. Good.
原来如此。好的。

Researcher 6:31:33
Let's go back to the topic just now. From a behavioral point of view, if you can't get what you want, will you continue to draw? Or will you give up?
我们回到刚才的话题，从行为角度上看，如果抽不到您想要的，您还会继续抽吗？还是会就此作罢？

Informant 014 6:31:52
I will not continue. It’s a pit.
不会继续 就是个坑

Informant 014 6:31:54
Yes.
对

Researcher 6:32:25
In the other case, if you get the item you want, will you continue to draw?
另一种情况，如果抽到了您想要的道具，您还会继续抽吗？

Informant 014 6:32:35
No, Haha.
不会哈哈

Informant 014 6:33:18
But your question… at the beginning, I want to draw it until I can get it.
但是你这个问题 一开始我想要的就要抽到能抽到为止。。

Researcher 6:33:42
So, can I understand a contradictory state?
原来如此，我能不能理解成一种矛盾的状态？

Informant 014 6:33:50
Yes.
嗯

Researcher 6:34:05
It’s just that mentally I don’t want to draw anymore because it’s a pit. But behaviorally you would still draw, is that the case?
就是心里想着不抽了，就是个坑。但是行为上依然还会继续抽，是这样吗？

Informant 014 6:34:26
I buy it at the end because I wanted it from the beginning.
买到低是因为一开始就想要

Informant 014 6:34:43
Sometimes I just give it a try when I’m boring.
试试运气就是闲得无聊试一下

Informant 014 6:34:45
In case I get it.

万一中了

Researcher 6:35:46
I see. The situation of the lottery we are talking about is in the game Luanshiwangzhe, is it?
原来如此。我们在谈的这种抽奖的情况是在乱世王者这款游戏中，是吗？

Informant 014 6:35:58
Yes.
嗯

Informant 014 6:36:06
The Karting is a sideline game.
卡丁车是业务游戏

Researcher 6:36:26
Ah, how do I understand the concept of "sideline game"?
啊，我怎么理解"业务游戏"这个概念呢？

Informant 014 6:36:40
It refers to the games that I play when I'm bored.
就是无聊的时候玩

Informant 014 6:36:49
I generally play Luanshiwangzhe.
主要还是玩乱世王者

Researcher 6:38:12
I see. According to the previous interviews, I learned that the lottery in the Luanshiwangzhe can be mainly divided into two categories. The first category is the items that can only be obtained through the lottery, and the other type is the item that can be obtained by other means rather than the lottery, but it is more difficult to obtain without drawing. Is my understanding correct?
原来如此。根据之前的访谈，我了解到乱世王者中的抽奖主要分为两类，第一类是只能通过抽奖获得的道具，另一类是能通过抽奖以外的方式获得的道具，但是比较难获得。我的理解对吗？

Informant 014 6:41:27
Emm, yes.
嗯是的

Researcher 6:42:18
If it is an item that can only be obtained through a lottery, what do you do if you want it, but you always cannot get it?

如果是只能通过抽奖获得的道具，如果您很想要，但是一直抽不到，您会怎么做？

Informant 014 6:42:45
If at the beginning I want the item, I would decide to draw until the end.
一开始决定想要的话抽到底

Researcher 6:43:23
I see. So there is a psychological expectation, and you have the psychological preparation of getting the desired item at the last round, right?
原来如此。就是有一个心理预期，做好了最后一个抽到想要的道具的心理准备，对吗？

Researcher 6:47:01
Hi~~~Are you there?
Hi~~你在吗？

Informant 014 6:47:58
Yes.
在

Researcher 6:48:00
The interview time has lasted for a long time, are you tired?

访谈时间比较久，你是不是累了？

Informant 014 6:48:26
Yes.
是的

Informant 014 6:48:34
My face is relatively black.
我比较脸黑

Researcher 6:49:06
:) It doesn't matter, if you are tired, we shall stop here.
：）不要紧，如果你累了的话我们就到此为止吧。

Researcher 6:49:28

If you think of something to add later, you can leave a message.

如果之后想到了什么需要补充的话，也可以给我留言。

Informant 014 6:50:25
I'm not tired
不累

Informant 014 6:50:32
What I have answered refers to the question above.
刚才回答的是上面的

Informant 014 6:50:48

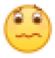

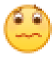

Researcher 6:50:55
Ok, ok, give me another 10 minutes? It's almost there.
嗯嗯好的，再给我 10 分钟好吗？马上就好

Researcher 6:52:46
I see. I will summarize the topic we have just mentioned about the loot boxes. There are two situations.
1. When you get the item you want: You do not continue the draw.
2. When you don't get the item you want: Mentally you don't continue drawing, but behaviorally you continue drawing.
原来如此。我归纳一下我们刚才关于抽奖箱的话题，有以下两种情况。
1. 抽到自己想要道具的情况：不继续抽了。
2. 抽不到自己想要道具的情况：心理想着不继续抽了，但是行为上还会继续抽。

Researcher 6:52:51
Can I interpret like this?
我这样理解对吗？

Informant 014 6:53:40
I would not draw behaviorally.
行为也不会抽哈哈

Informant 014 6:53:47
If I decide (to get the item) at the beginning, I would continue drawing.
一开始决定要的话会继续抽

Researcher 6:54:45
This is the case, that is to say, the behaviour can be divided into two situations:

1. The items you really want: draw until the end.
2. Other items: Take a chance.
原来如此，也就是说这边分为两种情况：
1. 自己很想要的道具：抽到底。
2.其它道具：碰运气。

Researcher 6:54:51
Can I interpret like this?
我能这样理解吗？

Informant 014 6:55:36
Yes yes yes.
对对对

Informant 014 6:55:40

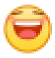

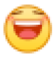

Informant 014 6:55:52
After all, the condition doesn't allow me to acquire everything I want.
毕竟条件不允许不能每个都想得到的

Informant 014 6:55:56

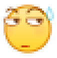

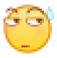

Researcher 6:57:00
I understand. Excuse me, is there any situation in which you have not decided on the items you want before the lottery, just want to buy the lottery box in order to try your luck?
我明白啦。请问，有没有您在抽奖前，完全没有决定好自己想要的道具的情况，就只想为了碰碰运气而去购买抽奖箱的经历？

Informant 014 6:57:31
Yes.
有

Informant 014 6:57:51
Just try a few rounds and stop.

就图个运气抽几下就收手

Informant 014 6:57:54
After that, I feel regret.
抽完就后悔了

Researcher 6:58:32
Does this regretful mentality affect your play game itself?
这种后悔的心态会影响到您游玩游戏本身吗？

Informant 014 6:58:43
No, it doesn't.
那倒没有

Informant 014 6:58:46
I'm ok after a while.
过一会就好了

Informant 014 6:59:01
Then I feel regret, because it's better to buy more foods and clothes.
就后悔充钱了还不如多买点吃的穿的

Researcher 6:59:14
But this regretful mentality will affect your continued draw of the loot boxes, right?
但是这种后悔的心态会影响到您继续玩抽奖箱，对吗？

Informant 014 6:59:25
Yes.
会

Informant 014 6:59:39
Mainly because others are lucky.. They draw a few rounds and get it.
主要是因为别人运气好。。抽几下中了

Informant 014 6:59:46
I think I can try either. . .
心思自己也试试。。。

Informant 014 6:59:55

Then... I get nothing.

然后。。。我啥都没抽到

Researcher 7:00:22
I would like to ask a few more questions. In other words, the result of someone else's Loot box draw affects your purchase of the Loot boxes. Can I understand like this?
我稍微多问几句哦。也就是说，别人的抽奖箱结果是会影响到您购买抽样箱的，我能这么理解吗？

Informant 014 7:00:42
Yes.
对的

Informant 014 7:01:02

I suppose that this is game planning, which serves to enhance consumption .

估计这个就是游戏策划 促进消费

Informant 014 7:01:42
For one skin, you need to spend 4000 if drawing to the end, and if you get the white face, you would spend 500 or 2000 to get it.
一个皮肤抽满中到是大概 4000 的话脸白的 500，要么 2000 就能抽到
Informant 014 7:01:55
So many people hold the mentality of trying to be luck.
所以抱着试试的心态的人很多

Researcher 7:02:06
Could you check my ideas to see if I'm right:
1. See others picking up great items.
2. You also want that item.
3. Try to draw (but not have the idea of drawing to the end)
4. Regret
您看一下我这个思路归纳对不对：
1. 看到别人抽到很棒的道具。
2. 自己也想要那个道具。
3.尝试去抽（但是没有做好抽到底的觉悟）
4.后悔

Researcher 7:02:11
Can I interpret like this?
我这样理解对吗？

Informant 014 7:02:54
Yes.

嗯

Researcher 7:03:10
I see. Are the figures we just mentioned are RMBs?
原来如此。我们刚才谈到的这些数字是人民币吗?

Informant 014 7:03:17
Somebody spend 4000 and get 2.
有的人 4000 还可以抽到 2 个

Informant 014 7:03:18
Yes.
是的

Informant 014 7:03:24
The skins in this game are all like this.
这个游戏皮肤都这样

Informant 014 7:03:29
The most expensive ones cost 5000 – 6000.
最贵 5000.6000

Informant 014 7:03:31

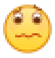

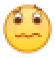

Researcher 7:03:34

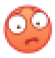That’s expensive!

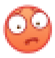 好贵！

Informant 014 7:03:38
Yes.
是啊

Informant 014 7:03:45
So this game is especially expensive.
所以这个游戏特别费钱

Informant 014 7:04:05

In the league, there is a person who spent 5000 but only got one (item), and (he/she) was sad.

盟里有一个脸黑的 5000 抽了一个都要哭死了

Researcher 7:04:45
Well, if others cannot get what they want, does this affect your intention to participate in the draw of Loot boxes.
嗯嗯，请问别人抽不到想要道具的体验会影响到您购买抽奖箱吗？

Informant 014 7:05:46
No.
不会

Researcher 7:06:17
Hum, only the case that other acquire their desired items affects you, right?
嗯嗯，只有别人抽中的经历才会影响，对吗？

Informant 014 7:06:37
It is not considered the impact
也不算是影响吧

Informant 014 7:06:40
There no feeling.
没啥感觉

Informant 014 7:07:02
They affect me if they get what they need using just a limited amount of money.
就是花最少的钱抽到会有点影响

Researcher 7:07:22
I see. I understand.
原来如此。我明白啦！

Researcher 7:07:23
The interview is almost over. Do you have any viewpoints to add?
访谈差不多要结束了。您还有什么观点需要补充吗？

Informant 014 7:07:40

No.

没有了

Informant 014 7:07:51

Do you want to develop a game?

你要开发游戏吗

Researcher 7:08:20

No, I am not developing games. I am a marketing researcher .

没有啦，我不是开发游戏的。我是市场营销专业的

Informant 014 7:08:27

Researcher 7:08:39

I'm doing a research about in-game goods purchasing .

在做一个游戏内购消费的课题

Researcher 7:08:43
These are all the questions. Thank you very much for participating in our research. Please confirm that your email address is XXXXXX@XXXXXX.com, because later we will send the JD electronic gift card to this address.
这就是全部的问题。 非常感谢您参与我们的研究。请确认您的电子邮件地址是XXXXXX@XXXXXX.com，因为稍后我们把京东电子礼品卡发送到这个地址。
