# Supplementary material for: A grounded theory approach to understanding in-game goods purchase
Source: PLoS One. 2022 Jan 27;17(1):e0262998. doi: 10.1371/journal.pone.0262998 (PMC8794092; doi:10.1371/journal.pone.0262998)
Supplement: S1 File — (ZIP) [file pone.0262998.s001.zip › Transcript 3.pdf]

Interview: 003

Informant: 003

Please note that the original transcript is in Simplified Chinese. The English translation is for internal communication among the author of this research, and it is not proofread. Potential linguistic errors may exist in the English translation.

Researcher 7:23:33
Thank you for your willingness to participate and be interviewed here. My name is XXX XXX, and I'm a PhD student in the XXX University of XXX(XXX). Currently, I'm working on a research project which focuses on videogame players' purchase motivations of in-game goods. Throughout this interview, I will ask you a series of questions and you are encouraged to express your opinions freely with emoticons. If I have questions about what you've said or need clarification about a topic or concept, I'll ask you.
感谢您愿意参加并在此接受采访。 我叫 XXX，我是市场营销学的博士生，现在我在 XXX 大学就读。 目前，我正在开展一个研究项目，专注于电子游戏玩家对游戏内购买项目的购买动机。 在整个访谈中，我会问您一系列问题，我们鼓励您自由表达您的意见和观点。因为这不是一个当面访谈，所以我们也鼓励您用 QQ 表情来表达您的情绪。 在访谈过程中，如果我对你所说的内容有疑问或需要您澄清一个主题或概念，我会问您。

Researcher 7:23:42
Are you ready?
您准备好了吗？

Informant 003 7:23:51
👌

👌

Researcher 7:24:06
In the previous survey, you mentioned that you purchased certain types of in-game purchases, including Power-ups, Expansions, Playable characters, Cosmetic/Skins, and Loot boxes.
在之前的调查问卷中，您已经提到您购买了某些类型的游戏内购买项目。包括增强道具，扩展包，可游玩角色，装饰/皮肤，抽奖箱和省时道具。

Researcher 7:24:20
What are your motivations for purchasing Power-ups type in-game goods?
您购买增强道具类游戏内购买项目的动机是什么？

Informant 003 7:24:57

To acquire happiness.
获得快乐

Researcher 7:25:11
Can you tell me more?
能说得详细一些吗？

Informant 003 7:25:46

To acquire happiness…which is a pure purpose.
就是获得快乐啊。。纯粹的目的

Informant 003 7:25:52
Stronger and more handsome
更强更帅

Researcher 7:26:32
"Stronger" I can understand, but how do I understand the word "more handsome"?
"更强"我能理解，但是我怎么理解"更帅"这个词？

Informant 003 7:26:49
After you spent the money, it makes you to feel...
就是让你感觉你花了钱之后

Informant 003 7:26:55
that you can acquire better appearances.
可以获得更好看的外观

Researcher 7:27:29
Ok. So, do you think there is a relationship between happiness and playability?
好的。那么您觉得活得快乐和游戏性之间有关系吗？

Informant 003 7:27:56
The playability should go first.
必须先有游戏性

Informant 003 7:28:04
The presence of playability leads people to recharge.
有了游戏性才会让人充值

Informant 003 7:28:15
If the game is not fun, no matter how good the appearance is, it doesn't work.
如果游戏不好玩，造型再好也没用

Researcher 7:28:54
Very interesting description. Let's move on to the next topic. How do you usually purchase Power-ups? Please tell me a general process.
很有意思的描述。我们继续下一个话题。您通常怎么样购买增强道具？ 请告诉我一个一般流程。

Informant 003 7:29:22
First, I check the event to see whether there is something I need or like.
先看看活动是否是自己需要的喜欢的

Informant 003 7:29:28
Then I count whether it is worthwhile
然后算一下是否值得

Informant 003 7:29:36
Then, I use the WeChat or Alipay to scan the code.
然后微信或者支付宝扫码

Researcher 7:29:52
The event you have just mentioned refers to the irregular activities organised inside the game?
这边说的活动指的是游戏内组织的不定期活动吗？

Informant 003 7:30:36
Yes.
是的

Researcher 7:30:59
So, what are the differences between such irregular activities and the game on a regular day?
那么这类不定期活动和平日里的游戏有什么区别呢？

Informant 003 7:31:38
I don't think there is any difference.
我觉得没有什么区别

Informant 003 7:32:05
For me, I don't care whether it is irregular or periodic.
对我来说不关心定不定期

Researcher 7:32:17
So, the meaning of event refers to the promotion?
所以活动的意思指的是促销？

Informant 003 7:32:19
I only care about the content provided during the activity. Do I need it or not.
我只关心这活动提供的内容我是否需要

Researcher 7:32:49
Is there any content only be sold during the event, not on regular days?
还是有一些内容只在活动期间出现，平日里不卖？

Informant 003 7:33:21
Respect to this, according to the games which currently I'm playing…
这个按照我目前玩的游戏来说

Informant 003 7:33:57
The fixed festival events are not necessarily better than the random activities on regular days.
节日的固定活动并不一定会比平时的随机活动好

Informant 003 7:34:10
Whether recharging or not dependents on one's needs.
这个是否充值完全是按照自己需求来

Informant 003 7:34:20
It is not associated with irregular or regular activities.
跟定不定期没有关联

Informant 003 7:34:45
Whether recharging is purely depends on whether the products provided by the company are attractive or not.
是否充值纯粹只取决于厂商提供的产品是否有诱惑力

Researcher 7:35:36
Ok. Can you please describe how the activities in the game are generally carried out?
好的。能否请您描述一下游戏内的活动一般是怎么进行的？

Informant 003 7:36:09
All activities are essentially the same.
所有的活动其实本质都是一样的

Informant 003 7:36:19
Products are provided, and consumers recharge to acquire the products.
提供产品，消费者充值获得产品

Researcher 7:37:04
So, in terms of the price, there are more discounts during the events than on regular

days?
那么在价格方面，在活动期间会不会比起平日里更优惠？

Informant 003 7:37:10
No, it is not.
并不会

Informant 003 7:37:49
The so-called discounts are only claimed by the companies themselves.
所谓的优惠只是厂商自己宣称的

Informant 003 7:38:12
The price of the same content will not be changed according to the regular days or events.
同一种内容并不会因为平时发售或者节日发售价格发生变化

Informant 003 7:38:24
Instead, activities during the festivals have a larger scale.
反而节日的活动会有更大的规模

Informant 003 7:38:34
which requires more money to participate.
需要更多的资金去参与

Researcher 7:39:17
The larger scale means that there are more available goods, so you need to prepare more funds. Can I interpret it like this?
更大的规模的意思是有更多的商品可买，所以您才需要准备更多的资金，我可以这么理解吗？

Informant 003 7:40:30
Yes, you can. However, there is another reason that students may have more money during the holiday season.
可以这么理解，但还有一个原因就是学生在节日可能会更有钱一点

Informant 003 7:40:36
This is determined by the market.
这是市场决定的

Informant 003 7:40:59
This also explains why during the Chinese New Year there always more activities are.
这也是为什么过年总是活动最多的时候

Researcher 7:41:04

So, the activities in the game are sometimes linked to the real festivals?
所以游戏内的活动有时候和现实中的节日是挂钩的？

Informant 003 7:41:18
That's for sure
那肯定的

Informant 003 7:41:49
This is determined by the interests
这是利益所决定的

Researcher 7:42:09
Ok. We continue. So when you buy other in-game goods, including Expansions, Playable characters, Cosmetic/Skins, Loot boxes and Time-savers, do you follow the same purchase process?
好的。我们继续。那么您在购买其他游戏内购，包括扩展包，可游玩角色，装饰/皮肤，抽奖箱和省时道具的时候，也遵循同一个购买流程吗？

Informant 003 7:42:22
I want to emphasize something.
我强调一下

Informant 003 7:42:33
There is no process for purchasing goods.
购买东西没有流程

Informant 003 7:42:36
It is not so complicated.
没有那么复杂

Informant 003 7:42:43
In one sentence
用一句话来说

Informant 003 7:42:48
If I like, I buy.
喜欢就买

Informant 003 7:43:21
Like- click to pay
喜欢—点击付款

Informant 003 7:43:25
That’s it.

完事

Researcher 7:43:50
Ok. Therefore, in general, these in-game goods are purchased directly with real money or are needed to be recharged first to the game currency and then purchased using the game currency?
好的。所以一般这些游戏内购是用真钱直接购买还是需要先充值游戏内的货币再用游戏内的货币购买。

Informant 003 7:44:03
Both.
都有

Informant 003 7:44:17
The difference is regulated according to national laws.
这个根据国家法律不同

Informant 003 7:44:20
Different forms
形式也不同

Informant 003 7:44:34
But, essentially, they are real money purchasing.
但是本质上都是真实货币购买

Researcher 7:45:35
Ok. So how do you get the information of the in-game goods?
好的。那么您是怎么样获取这些游戏内购的信息的呢？

Informant 003 7:45:56
App or official website push
app 或者官网推送

Researcher 7:46:42
Does the app mentioned here refer to the game app or social media apps?
这边说的 app 是指游戏 app 还是其它社交媒体 app 呢？

Informant 003 7:46:57
Both.
都有

Researcher 7:47:27
Ok. So what is the official website push? In the form of mail?
好的。那么官网推送指的是？以邮件的形式吗？

Informant 003 7:47:55
There are also advertisements in the game.
游戏内也会有广告

Informant 003 7:48:02
There are also advertisements on the official website.
网站上也会有广告

Informant 003 7:48:25
Some mails are on PS4
有些比如 ps4 也会有邮件

Researcher 7:50:03
I see. So, after you know the existence of the in-game goods, will you learn through some channels to better understand the information of these products?
原来如此。那么在您知道了这些游戏内购的存在之后，会不会通过一些渠道去更好地了解这些产品的信息？

Informant 003 7:50:30
The products which require channels to learn...
需要渠道了解的产品

Informant 003 7:50:35
are not good products.
不是好产品

Informant 003 7:50:52
Good products should be simple and clear.
好产品应该简洁明了

Informant 003 7:51:10
Only bad things only need the bells and whistles
只有糟糕的东西才需要花里胡哨

Researcher 7:51:39
In other words, the more intuitive the product, the more attractive to you?
也就是说，越直观的产品越能吸引您？

Informant 003 7:52:10
Not necessarily.
并不是

Informant 003 7:52:21

There are not so many reasons for spending.
消费没有那么多理由

Informant 003 7:52:24
If I like, I buy.
喜欢就买

Informant 003 7:52:33
If your product design is complicated
如果你产品设计的很复杂

Informant 003 7:52:42
then who has so much time to study
谁有那么多时间去研究

Informant 003 7:53:02
It's the thing of degree.
这个是度的把握

Informant 003 7:53:31
What I only express is that complex activities often do not make people like them.
我仅仅表达的是，复杂的活动往往并不让人喜欢

Informant 003 7:53:45
But it does not mean that the simpler the activity is, the better
但不代表越简单的活动是越好的

Informant 003 7:54:34
For example, through an event, an item can be purchased directly for 1000 yuan.
打个比方，一个活动 1000 块钱可以直接买一个道具

Informant 003 7:54:57
Another event is a draw, with a guaranteed (acquisition) for 1500 and an average (acquisition) for 500.
另一个活动是抽奖，保底 1500，人均 500 可以获得

Informant 003 7:55:02
Which one do you think is better?
你觉得是哪个好呢

Informant 003 7:55:17
You can say that the first one is more direct, but the second one is not complicated.
你可以说第一个更直接，但第二个也不复杂

Informant 003 7:55:33
And the second one can give you a pleasant surprise.
而且第二个还能给你一种惊喜的快乐

Researcher 7:56:17
I see. You have just mentioned the loot boxes items. In addition to the joy of surprise, what are other purchasing motivations?
原来如此。您刚才提到抽奖箱类道具，除了这种惊喜的快乐，还有什么其它的购买动机吗？

Informant 003 7:56:41
Surprise is just an added...
惊喜只是附加

Informant 003 7:56:52
Eventually, to acquire products.
最终还是看获得产品

Researcher 7:57:20
It turned out to be the case. Let's move on to the next topic. When you purchase in-game goods, will you evaluate the alternative solutions of in-game purchase? For example, acquiring the same item in a free way?
原来如此。我们进入下一个话题。您购买游戏内购的时候，会不会去比较游戏内购的替代方案？比如说通过免费的方式来获取相同的道具？

Informant 003 7:57:37
No
不会

Informant 003 7:57:45
Time is money.
时间就是金钱

Informant 003 7:58:09
Free often requires a larger cycle
免费往往需要更大的周期

Researcher 8:00:02
I understand. Speaking of this, I recall time-savers types in-game goods.
我明白了。说到这个，我想到了省时道具类的游戏内购。

Informant 003 8:00:21
The time-saver is a kind of product.
省时也是一种产品

Informant 003 8:00:35
It is essentially the same as other contents
和其他内容本质是一样的

Researcher 8:01:04
So your motivation for buying time-savers items is to exchange money for time. Can I understand like this?
所以您购买省时道具类产品的动机是为了用金钱换取时间,我可以这样理解吗?

Informant 003 8:01:22
Partially yes.
部分是

Researcher 8:01:33
Can you describe the other part?
能描述下另外那部分吗?

Informant 003 8:02:27
Another reason is that sometimes you can't get the final reward if you don't buy it.
另一部分原因是有时候你不买就无法获得最终的奖励

Informant 003 8:02:46
Some activities are beyond the limits of normal people.
有些活动是超过正常人极限的

Researcher 8:03:08
Can you give an example?
可以举个例子吗?

Informant 003 8:03:50
For example, they would design an activity that requires you to fight 7 days X 24 hours.
比如他设计一个活动是需要你 7X24 小时肝的

Informant 003 8:03:59
After buying the item, it only requires 2 hours a day.
买了道具只要一天 2 小时

Informant 003 8:04:54
Or some items are very attractive when they come out, but when you finish it through free methods, there will be a better substitute.
又或者有些道具出来的时候很有吸引力，但当你通过免费完成时，以后有了更好的代替品出现

Informant 003 8:05:08
At this time, pure free items are no longer attractive.
这时候纯粹的免费道具已经没有吸引力了

Researcher 8:06:01
I understood.
我明白了。

Researcher 8:06:16
Then the better alternatives are often charged, right?
那么这边的更好的替代品往往是要收费的，是吗？

Informant 003 8:07:01
Yes.
是的

Researcher 8:07:47
We have just talked about the concept of "final reward". What do you mean by that?
我们刚才谈到了"最终的奖励"这个概念，具体是指的什么意思呢？

Informant 003 8:08:13
It is the literal meaning
就是字面的意思

Researcher 8:08:50
Does this "final reward" have to be obtained through the internal mechanisms of the game?
这个"最终的奖励"是必须要通过游戏内部机制获得的吗？

Informant 003 8:09:22

(Do you think that) a modifier can be used?

难道可以用修改器嘛

Researcher 8:10:14
This means that this "final reward" cannot be bought with money, but the items you acquire with money can help you get this reward. Can I interpret like this?
也就是说这个"最终的奖励"不能用钱买，但是您用钱买的道具能帮助您获得这个奖励，我可以这样说吗？

Informant 003 8:10:34
Yes, you can.
可以这样理解

Researcher 8:12:15
Ok. What kind of game does this "final reward" generally appear in?
好的。这个"最终的奖励"一般是出现在哪种游戏中呢？

Informant 003 8:12:32
It appears in a successful company.
出现在一个成功的公司里

Informant 003 8:12:38
It is not related to the game.
和游戏无关

Informant 003 8:13:15
No matter how good the game is, it goes down without good planning.
再好的游戏没有好的策划一样倒闭

Researcher 8:14:31
Ok. Let's talk about the Playable characters. In many games, some characters are paid for use. What are your motivations for buying them?
好的。我们再聊一下可游玩角色。在很多游戏内，有些角色是需要付费使用的。您购买它们的动机是什么呢？

Informant 003 8:15:25
Same as other types of recharge.
和其他类型充值都是一样的

Informant 003 8:15:32
All for a better gaming experience.
都是为了更好的游戏体验

Researcher 8:17:00
Ok. Then there are some differences in the Expansions type in-game goods, and we often only buy it with real money. Can you please talk about the motivations to buy such goods?
好的。那么扩展包类的内购有一些不一样，我们往往只能用真钱购买。能请您谈一下购买这类内购的动机吗？

Informant 003 8:17:10
There is no difference.
没有区别

Informant 003 8:17:24
Think about it carefully.

你仔细想想

Informant 003 8:17:46
Does the expansion pack also provide you with a more complete gaming experience?
扩展包不也是提供了你更完整的游戏体验吗

Researcher 8:18:40
So do you think that the "complete game experience" is part of the playability?
所以您认为 "完整的游戏体验"是游戏性的一部分？

Informant 003 8:18:52
That's for sure.
那肯定的

Researcher 8:19:39
Ok. We know there are different types of in-game goods. When you buy in-game goods, do you have a priority in mind? For example, would you give priority to buying some types of product to another types of product?

好的。我们知道有不同类型的游戏内商品。 当您购买游戏内商品时，您是否心里有一个优先顺序。比如比起一类游戏内商品您会优先购买另一类商品？

Informant 003 8:20:40
Having played for a long time is more preferred
玩的时间久的更优先

Researcher 8:21:27
Does the long playing time refer to the game itself?
玩的时间久是指游戏本身吗？

Informant 003 8:21:48
Yes.
是的

Researcher 8:22:49
No, what we are talking about is, for example, there are several options: Power-ups, Expansions, Playable characters, Cosmetic/Skins, Loot boxes and Time-savers. Will you have a priority in your purchase?
不，我们在说的是，比如有增强道具，扩展包，可游玩角色，装饰/皮肤，抽奖箱和省时道具几个选项。您会在购买的时候心里有一个优先顺序吗？

Informant 003 8:23:25
No, I will not.
不会

Researcher 8:24:40
These are all the questions. Thank you very much for participating in our research. Please confirm that your email address is XXXXXX@XXXXXX.com, because later we will send the JD electronic gift card to this address.
这就是全部的问题。 非常感谢您参与我们的研究。请确认您的电子邮件地址是XXXXXX@XXXXXX.com，因为稍后我们把京东电子礼品卡发送到这个地址。
