# Supplementary material for: A grounded theory approach to understanding in-game goods purchase
Source: PLoS One. 2022 Jan 27;17(1):e0262998. doi: 10.1371/journal.pone.0262998 (PMC8794092; doi:10.1371/journal.pone.0262998)
Supplement: S1 File — (ZIP) [file pone.0262998.s001.zip › Transcript 4.pdf]

Interview: 004

Informant: 004

Please note that the original transcript is in Simplified Chinese. The English translation is for internal communication among the author of this research, and it is not proofread. Potential linguistic errors may exist in the English translation.

Researcher 10:29:36
Thank you for your willingness to participate and be interviewed here. My name is XXX XXX, and I'm a PhD student in the XXX University of XXX(XXX). Currently, I'm working on a research project which focuses on videogame players' purchase motivations of in-game goods. Throughout this interview, I will ask you a series of questions and you are encouraged to express your opinions freely with emoticons. If I have questions about what you've said or need clarification about a topic or concept, I'll ask you.
感谢您愿意参加并在此接受采访。 我叫 XXX，我是市场营销学的博士生，现在我在 XXX 大学就读。 目前，我正在开展一个研究项目，专注于电子游戏玩家对游戏内购买项目的购买动机。 在整个访谈中，我会问您一系列问题，我们鼓励您自由表达您的意见和观点。因为这不是一个当面访谈，所以我们也鼓励您用 QQ 表情来表达您的情绪。 在访谈过程中，如果我对你所说的内容有疑问或需要您澄清一个主题或概念，我会问您。

Researcher 10:29:40
Are you ready?
您准备好了吗？

Informant 004 10:29:48
Yes.
好了

Researcher 10:29:56
In the previous survey, you mentioned that you purchased certain types of in-game purchases, including Power-ups, Expansions, Playable characters, Cosmetic/Skins, and Loot boxes.
在之前的调查问卷中，您已经提到您购买了某些类型的游戏内购买项目。包括增强道具，扩展包，可游玩角色，装饰/皮肤和抽奖箱。

Researcher 10:30:03
What are your motivations for purchasing Power-ups type in-game goods?
您购买增强道具类游戏内购买项目的动机是什么？

Informant 004 10:30:39
Faster upgrades and faster push rates in maps.
更快升级还有加快推图速度

Researcher 10:31:06
How can I understand the concept “faster push rates in maps”.
我怎么理解"加快推图速度"这个概念呢？

Informant 004 10:31:41
For example, in Honkai Impact 3rd, many high-level weapons are required to recharge.
就像崩坏 3 的高级武器都是需要充值抽的

Informant 004 10:31:57
If you don't have high-level weapons, the push speed will be slow.
没有高级武器的话推图速度会很慢

Researcher 10:32:35
Ah, I understand, so to get such items, you need to draw them first with a loot box. Is that true?
啊我明白了，所以要获取这类道具需要先用抽奖箱抽取，是这样吗？

Informant 004 10:32:43
Yes.
对的

Informant 004 10:32:54
Moreover, there is something like a monthly card.
还有一下像月卡之类的

Researcher 10:33:05
So, in this case, can the look box be acquired by the in-game mechanism? Or you still need to use real money to buy?
那么在这种情况下，抽奖箱能考游戏内机制获取吗？还是需要使用真钱来购买？

Informant 004 10:33:39
They can be obtained by in-game mechanism but very slowly.
可以靠游戏内机制获取但是很慢

Researcher 10:34:32
Is there a situation where you can purchase Power-ups directly?
有没有可以直接购买增强道具的情况？

Informant 004 10:35:05
There are also some items like auxiliary role upgrades, evolutions, double experience, and double game currency.
还有一些比如说双倍经验和双倍游戏币之类的辅助角色升级和进化

Informant 004 10:36:09
There is also a fighting PC end game called "Splash Fighters", which basically cannot be played without buying RMB weapons.
还有就是以前玩的一款叫热血英豪的格斗端游是不购买 RMB 武器基本上没法玩的

Researcher 10:37:02
Honkai Impact 3rd is mobile online game and Splash Fighters is PC online game, is this true?
崩坏 3 是手机网络游戏，热血英豪是 PC 端网游，是这样吗？

Informant 004 10:37:07
Yes.
对的

Researcher 10:37:31
How do you usually buy these in-game items? Please tell me a general process.
您通常怎么样购买这些游戏内道具？ 请告诉我一个一般流程。

Informant 004 10:39:15
Under normal circumstances, there is a promotion announcement... for example, from XX(day) to XX(day), items or newly introduced products will be sent as gifts after purchasing certain products.
一般情况下是推广公告比如说几号到几号开始搞活动购买那些商品会赠送道具或者是新推出的商品测试服很厉害会买

Researcher 10:39:48
From which channels do you know these promotional announcements?
这些推广公告您是从哪些渠道获知的？

Informant 004 10:40:21
Online game official website and APP pushing information
网游官网和 APP 推送信息

Informant 004 10:40:34
Also, there is in-game pushing.
还有就是游戏内推送

Researcher 10:40:42
The APP you have mentioned refers to game APP or social media APP?
这边的 APP 指的是游戏 APP 还是社交媒体 APP 呢？

Informant 004 10:41:35
Similar to Tappap and game assistant

类似 taptap 和游戏助手

Informant 004 10:41:59
Rarely on social media, but on some video platforms.
社交媒体的话比较少还有一些视频网站

Informant 004 10:42:08
Such as A and B webs.
比如说 AB 站

Researcher 10:42:32
I see. So, during the in-game events, what are the differences comparing with regular days?
原来如此。那么在游戏内活动期间，和平日里的游戏会有哪些不同？

Informant 004 10:43:28
During the event, for example, League of Legends and Dota require purchasing a pass to get rare videos and skins.
活动期间的话，比如说英雄联盟和刀塔需要购买通行证才能获取稀有的视频和皮肤

Informant 004 10:44:06
These items and accessories are not available on regular days.
平日的话这些道具和饰品是买不到的

Researcher 10:44:35
It's interesting. Can you talk about the relationship between the pass and the rare items?
这很有趣。您能具体说一下通行证和稀有道具间的关系吗？

Informant 004 10:46:27
There is an indirect relationship. For example, some jewelry can only be exchanged at the pass stage, and the items that can be purchased are not as attractive as rare products.
有简介的关系吧，比如说有些饰品只有通行证阶段才能兑换或者购买平时能购买的商品没有稀有商品吸引力高

Informant 004 10:47:10
Attributes or display effects are much brighter than normal items.
属性或者显示特效比普通的炫很多

Researcher 10:47:29
I understand, that is to say, the pass is a prerequisite for purchasing other items. There is no way to buy rare items without a pass.
噢我懂了，也就是说通行证是一个购买其它道具的前提。没有通行证的话就没有

办法购买稀有道具。

Researcher 10:47:40
Is it ture?
是这样吗？

Informant 004 10:47:58
Yes, purchase or exchange.
对的购买或者兑换

Informant 004 10:48:57
Of course, when you purchase a pass, you will also get a discount when you purchase some limited items.
当然购买通行证的话在购买限定商品的时候也有一定的优惠

Researcher 10:49:18
Ah, so it also serves as a discount coupon?
啊，所以它还有一个打折券的作用？

Informant 004 10:49:38
There is also a reward mechanism
对的还有一下奖励机制

Informant 004 10:49:41
Some.
一些

Researcher 10:50:34
Then the pass itself needs to be purchased with real money and cannot be obtained through in-game mechanisms. But rare items that can be purchased through a pass can be obtained through real money and in-game currency. Am I right?
那么通行证本身是需要用真钱购买的，并不能通过游戏内机制获取。但是通过通行证能购买的稀有道具是能通过真钱和游戏内货币获取的。我能这样理解吗？

Informant 004 10:50:36
In this kind of activity, the game often takes a long time.
在这种活动的时候游戏时常会比较久

Informant 004 10:52:08
Yes.
可以

Researcher 10:53:17
We just talked about the Power-ups, and you said that your motivation to buy is mainly

to speed up the game. So what are the motivations for you to purchase an Expansions, Playable characters, Cosmetic/Skins, and Loot boxes?
我们刚才谈到了增强道具，您说到了购买动机主要是加快游戏进度。那么您购买扩展包，可游玩角色，装饰/皮肤和抽奖箱的动机分别是什么呢？

Informant 004 10:53:44
Wait a minute, let me open the door.
稍等我去开一下门

Researcher 10:53:49
Ok.
Ok

Informant 004 10:58:55
In terms of the Expansions, like Resident Evil, after purchasing DLCs, new contents and new stages are availables to be played. Cosmetic/Skins and Loot boxes are mainly cool as they look different. League of Legends, Dota, and Overwatch are like this. These items do not destroy the game balance but look cool.
拓展包的话像生化危机的 DLC 购买以后会有新的资料片和新关卡可以玩，装饰皮肤抽奖箱只要是看起来很酷炫与众不同感觉很帅像英雄联盟刀塔守望先锋之类的就是属于这种不破坏游戏平衡然后看起来很酷炫

Informant 004 11:00:38
If the game character is new, the new character is always very attractive. Many high-level players introduce their trail video.
游戏角色的话新的角色总是很吸引人，很多高玩会出教程试玩

Informant 004 11:00:52
Mainly there are more gaming methods.
主要是玩法变多了

Researcher 11:01:51
Where do the high-level player generally appear?
高玩一般在哪些地方出现？

Informant 004 11:02:17
On live websites and video websites.
直播网站和视频网站

Informant 004 11:02:35
They do live or videos themselves.
他们会开直播或者自己做视频

Researcher 11:02:49

Ok. You said that the new characters are attractive, specifically why?
好的。您说到新角色吸引人，具体是为什么？

Informant 004 11:03:32
Appearance and unique skill attributes of characters.
角色的外形和独特的技能属性

Researcher 11:04:16
Understood. Speaking of more playability, do you think that diversified gameplay is an important part of playability?
了解了。说到玩法变多了，您认为多元化的玩法是游戏性的重要组成部分吗？

Informant 004 11:04:25
Yes.
对的

Researcher 11:05:02
Ok. We continue the topic of the purchase process of the game props just now. After knowing the existence of these in-game goods, will you go through some channels to search for details of these items during the purchase process?
好的。我们继续刚才游戏道具的购买过程这个话题。在知道了这些游戏内商品的存在后，在购买过程中，您是否会去通过一些渠道搜索这些商品的详细信息？

Informant 004 11:06:19
Yes. For example, Tieba and official introduction
会，比如说贴吧和官方介绍

Informant 004 11:07:18
Also in QQ group and WeChat group.
还有比如说 QQ 群和微信群

Researcher 11:08:45
It turned out to be the case. When you purchase in-game goods, will you evaluate the alternative solutions of in-game purchase? For example, acquiring the same item in a free way?
原来如此。在游戏内商品的购买过程中，您是否经常评估游戏内购的替代方案？比如尝试用免费的方式获取相同效果的道具？

Informant 004 11:10:06
Yes, but this will take a lot of time. Due to that I have already had a job, I do not have much time to do tasks.
会但是这种会比较耗费时间因为上班了所以没呢么多时间去做任务

Researcher 11:11:12

Ok. We know there are different types of in-game goods, including Power-ups, Expansions, Playable characters, Cosmetic/Skins, Loot boxes, and time savers. When you buy in-game goods, do you have a priority in mind? For example, would you give priority to buying some types of product to another types of product?
好的。我们知道有不同类型的游戏内商品 包括增强道具，扩展包，可游玩角色，装饰/皮肤，抽奖箱和省时道具。 当您购买游戏内商品时，您是否心里有一个优先顺序。比如比起一类游戏内商品您会优先购买另一类商品？

Informant 004 11:12:40
For example, a limited-time item or a product which has a bonus during the event will be purchased first.
会比如说限时商品或者就是在活动期间有加成的商品会优先购买

Researcher 11:14:23
I see.
原来如此。

Researcher 11:17:35
These are all the questions. Thank you very much for participating in our research. Please confirm that your email address is XXXXXX@XXXXXX.com, because later we will send the JD electronic gift card to this address.
这就是全部的问题。 非常感谢您参与我们的研究。请确认您的电子邮件地址是XXXXXX@XXXXXX.com，因为稍后我们把京东电子礼品卡发送到这个地址。
