# Supplementary material for: A grounded theory approach to understanding in-game goods purchase
Source: PLoS One. 2022 Jan 27;17(1):e0262998. doi: 10.1371/journal.pone.0262998 (PMC8794092; doi:10.1371/journal.pone.0262998)
Supplement: S1 File — (ZIP) [file pone.0262998.s001.zip › Transcript 5.pdf]

Interview: 005

Informant: 005

Please note that the original transcript is in Simplified Chinese. The English translation is for internal communication among the author of this research, and it is not proofread. Potential linguistic errors may exist in the English translation.

Researcher 13:11:07
Thank you for your willingness to participate and be interviewed here. My name is XXX XXX, and I'm a PhD student in the XXX University of XXX(XXX). Currently, I'm working on a research project which focuses on videogame players' purchase motivations of in-game goods. Throughout this interview, I will ask you a series of questions and you are encouraged to express your opinions freely with emoticons. If I have questions about what you've said or need clarification about a topic or concept, I'll ask you.
感谢您愿意参加并在此接受采访。 我叫 XXX，我是市场营销学的博士生，现在我在 XXX 大学就读。 目前，我正在开展一个研究项目，专注于电子游戏玩家对游戏内购买项目的购买动机。 在整个访谈中，我会问您一系列问题，我们鼓励您自由表达您的意见和观点。因为这不是一个当面访谈，所以我们也鼓励您用 QQ 表情来表达您的情绪。 在访谈过程中，如果我对你所说的内容有疑问或需要您澄清一个主题或概念，我会问您。

Researcher 13:11:17
Are you ready?
您准备好了吗？

Informant 005 13:11:22
Yes.
可以

Researcher 13:11:51
In the previous survey, you mentioned that you purchased certain types of in-game purchases, including Expansions, Playable characters, and Cosmetic/Skins.
在之前的调查问卷中，您已经提到您购买了某些类型的游戏内购买项目，包括扩展包，可游玩的角色和装饰/皮肤。

Researcher 13:12:02
What are your motivations for purchasing Expansions type in-game goods?
您购买扩展包类游戏内购买项目的动机是什么？

Informant 005 13:12:26
Comfortable looking, and cool playing.
看着舒服，玩的爽

Researcher 13:12:52
Can you talk more about this?
您能就这个再说点细节吗？

Informant 005 13:14:26
For example, I buy some skins for my characters because of cool special effects, which makes game playing more interesting.
比如会购买游戏角色皮肤，有的会有酷炫的特效，玩起来更带劲。

Researcher 13:15:19
Ok, we just talked about Cosmetic/Skins. How do you usually buy Cosmetic/Skins? Please tell me a general process.
好的，我们刚才谈到了装饰/皮肤。您通常怎么样购买装饰/皮肤呢？ 请告诉我一个一般流程。

Informant 005 13:16:30
First, I recharge in the game, then I go to the game store to buy.
先给游戏充值，再去游戏商城购买

Researcher 13:16:56
Do you firstly recharge the in-game currency?
是先充值成游戏内的货币吗？

Informant 005 13:16:58
Now there are also direct purchasing methods, and the payment is via WeChat QR code.
现在也有直接点购买，通过二维码微信付款的

Informant 005 13:17:17
Yes.
对

Researcher 13:17:47
Ok, how do you generally get the product information of the Cosmetic/Skins?
好的，您一般是如何获取装饰/皮肤的产品信息的？

Researcher 13:18:06
What are the general channels?
一般有哪些渠道

Informant 005 13:18:24
Official advertisements
官方宣传广告

Researcher 13:18:39

In general, where do these advertisements appear?
一般这些广告在哪些场合出现呢？

Informant 005 13:20:02
In-game, game related forums, videogame live, etc.
游戏内，游戏相关论坛，游戏直播等

Researcher 13:21:47
I see. So, after understanding the existence of these products, will you use some channels to better understand the details of these skins?
原来如此。那么在了解有这些产品的存在后，您是否会通过某些渠道去更好地这些皮肤的详细信息？

Informant 005 13:23:28
I watch the display of the game skins on the official website or in the game to learn more about the skin details to consider whether I need to buy.
会在官方网页或游戏内观看游戏皮肤展示视屏，了解更多皮肤细节，以考虑是否需要购买

Researcher 13:24:55
I see. It turned out to be the case. When you purchase in-game goods, will you evaluate the alternative solutions of in-game purchase? For example, acquiring the same item in a free way?
原来如此。那么在皮肤购买过程中，您是否经常评估这类商品的替代方案？比如用免费的方式去获取皮肤？

Informant 005 13:26:42
I would consider. Generally, there are discounting activities from time to time, and I would select (the items) in advance to purchase them during the promotion.
会考虑，一般都会时不时打折活动，会事先选好，在打折活动的时候购买

Researcher 13:27:43
When such activities usually occur?
一般这类活动什么时候会出现？

Informant 005 13:31:22
After the festivals or official tournaments.
节日或者官方赛事后

Researcher 13:32:10
Ok. How do I understand the concept of "festival"? Is the festival here a fictitious festival in the game or is it linked to a festival in reality?
好的。我怎么理解"节日"这个概念呢？这边说的节日是游戏内虚构的节日还是和现实中的节日挂钩呢？

Informant 005 13:33:43
It's linked to real-life festivals, such as Valentine's Day, Christmas, Spring Festival, etc.
会和现实生活中的节日挂钩，比如情人节啊，圣诞节啊，春节啊，等等

Researcher 13:34:40
I see. So, during these real holiday seasons, will there be something different in the game?
原来如此。那么在这些现实中的节日期间，游戏内会有和平日不一样的地方吗？

Informant 005 13:35:53
Game scenes and background music will be changed.
游戏场景，背景音乐都会跟着一起变换

Researcher 13:36:46
Ok, so, during the festival in the real world, festivals will be synchronized in the game, along with discounts. Can I understand this?
好的，所以在现实中节日期间，游戏内也会同步进行节日活动，并且伴随打折活动。我可以这样理解吗？

Informant 005 13:37:22
Yes.
是的

Researcher 13:37:54
Ok. We continue. What does the “official tournament” specifically mean?
好的。我们继续。请问“官方赛事”具体指的是什么？

Informant 005 13:40:11
Videogame companies often hold many international videogame tournaments, where there are teams from many countries. The team that won the championship will design a special skin, which is often cool.
游戏方会举办很多国际游戏赛事，会有很多国家的战队参加，最终夺冠的队伍会专门设计一套专属皮肤，往往比较酷炫。

Researcher 13:41:07
I see. So, we talking about in a specific game?
原来如此。所以我们现在说的是具体某一个游戏吗？

Researcher 13:41:27
Or, is it a general phenomenon?
还是一个很广泛的现象？

Informant 005 13:42:54

Some famous MOBA games.
一些知名的 MOBA 游戏

Informant 005 13:43:12
Often hold tournaments.
都会经常举办赛事

Researcher 13:45:11
I understand. In other words, the whole process is like this:
1. Official tournaments are organised.
2. Introduce a dedicated skin for the teamwhich won the championship.
3. There will be discount promotions after the official tournament.

Is the whole process like this?
我明白了。也就是说整个流程是这样的：
1. 举办官方赛事。
2. 为夺冠的队伍推出一套专属皮肤。
3. 官方赛事后有打折促销活动。

整个流程是这样吗？

Informant 005 13:46:25
Yes.
对

Researcher 13:48:10
Ok. Let's change the subject. In the previous questionnaire, you also mentioned that you have purchased Playable characters type in-game goods in the past 6 months. What is your motivation for purchasing Playable characters type in-game goods?
好的。我们换个话题。在之前的问卷中，您也有谈到在过去 6 个月中有购买过可游玩的角色类的游戏内购。请问您购买可游玩的角色类游戏内购买项目的动机是什么？

Informant 005 13:51:05
It takes a long time for me to accumulate the in-game coins. As a result, when I encounter some new favourite characters, I will consider to purchase them directly by RMB.
攒游戏金币换购时间比较久，所以在碰到特别喜欢的新角色时，会考虑直接人民币购买。

Researcher 13:52:59
I see. So, is there a difference between the purchasing process of Playable characters and Cosmetic/Skins?
原来如此。那么您在购买可游玩的角色类的游戏内购的流程和刚才谈到的装饰/

皮肤类的游戏内购有没有不同呢？

Informant 005 13:53:21
No.
没有。

Researcher 13:54:05
Ok, Generally, how do you acquire product information for Playable characters type in-game goods?
好的，您一般是如何获取可游玩的角色类游戏内购的产品信息的？

Informant 005 13:56:59
Official advertising.
官方推广广告

Researcher 13:57:35
Ok. That is to say, the information channel remains to be the same as Cosmetic/Skins, as we have just mentioned, right?
好的。也就是和刚才说的皮肤类的信息渠道是一样的，对吗？

Informant 005 13:57:43
Yes.
对

Researcher 13:58:20
Ok. We know there are different types of in-game goods, including Expansions, Playable characters, and Cosmetic/Skins. When you buy in-game goods, do you have a priority in mind? For example, would you give priority to buying some types of product to another types of product?
好的。我们知道有不同类型的游戏内商品，比如扩展包，可游玩的角色和装饰/皮肤。 当您购买游戏内商品时，您是否心里有一个优先顺序。比如比起一类游戏内商品您会优先购买另一类商品？

Informant 005 14:03:43
Nope, the most important thing is whether it is attractive.
没有诶，主要还是看是否有足够的吸引力。

Researcher 14:04:13
These are all the questions. Thank you very much for participating in our research. Please confirm that your email address is XXXXXX@XXXXXX.com, because later we will send the JD electronic gift card to this address.
这就是全部的问题。 非常感谢您参与我们的研究。请确认您的电子邮件地址是XXXXXX@XXXXXX.com，因为稍后我们把京东电子礼品卡发送到这个地址。
