# Supplementary material for: A grounded theory approach to understanding in-game goods purchase
Source: PLoS One. 2022 Jan 27;17(1):e0262998. doi: 10.1371/journal.pone.0262998 (PMC8794092; doi:10.1371/journal.pone.0262998)
Supplement: S1 File — (ZIP) [file pone.0262998.s001.zip › Transcript 6.pdf]

Interview: 006

Informant: 007

Please note that the original transcript is in Simplified Chinese. The English translation is for internal communication among the author of this research, and it is not proofread. Potential linguistic errors may exist in the English translation.

Researcher 9:10:25
Thank you for your willingness to participate and be interviewed here. My name is XXX XXX, and I'm a PhD student in the XXX University of XXX(XXX). Currently, I'm working on a research project which focuses on videogame players' purchase motivations of in-game goods. Throughout this interview, I will ask you a series of questions and you are encouraged to express your opinions freely with emoticons. If I have questions about what you've said or need clarification about a topic or concept, I'll ask you.
感谢您愿意参加并在此接受采访。 我叫 XXX，我是市场营销学的博士生，现在我在 XXX 大学就读。 目前，我正在开展一个研究项目，专注于电子游戏玩家对游戏内购买项目的购买动机。 在整个访谈中，我会问您一系列问题，我们鼓励您自由表达您的意见和观点。因为这不是一个当面访谈，所以我们也鼓励您用 QQ 表情来表达您的情绪。 在访谈过程中，如果我对你所说的内容有疑问或需要您澄清一个主题或概念，我会问您。

Researcher 9:10:38
Are you ready?
您准备好了吗？

Informant 007 9:10:59
I'm ready.

我准备好了

Researcher 9:11:11
Great~What's your family name?

好~您贵姓？

Informant 007 9:11:17
Xu
许

Researcher 9:11:28
In the previous survey, you mentioned that you purchased certain types of in-game

purchases, including Expansions and Cosmetic/Skins.
好的。在之前的调查问卷中，您已经提到您购买了某些类型的游戏内购买项目,包括扩展包和装饰/皮肤。

Researcher 9:11:35
What are your motivations for purchasing Expansions type in-game goods?
请问您购买扩展包类游戏内购买项目的动机是什么？

Informant 007 9:12:55
My motivations to purchase expansions packages are: It's an endorsement of the game, and I feel that the game is not long enough. Buying the expansion package may increase the fun.
扩展包一般购买的动机　是对这款游戏的认可　同时会觉得游戏时长不够　购买扩展包 可能增加乐趣

Researcher 9:13:58
How can I understand the notion of “the game is not long enough”?
我怎么理解“游戏时长不够”这个概念？

Informant 007 9:15:01
For example, purchasing additional expansion packages increases gaming time on some stand-alone games.
比如 PC 上的单机游戏　额外的购买扩展包　增加游玩时长

Informant 007 9:15:45
Will it be my understanding of the expansion pack is wrong?

不会是我对扩展包的理解有错误吧？

Researcher 9:16:16
Don’t worry. You are right.
不不，这个理解是对的。

Researcher 9:16:51
Are there other motivations besides this?
除了这个之外还有其它的动机吗？

Informant 007 9:18:38
It depends on what the expansion package is. If it is just adding fun or difficulty or a new chapter to the original game, the reason for the purchase is what I said above.
这得看扩展包具体是什么了　如果是单单在原本游戏上增加乐趣或难度或新篇章　那购买理由就是我上述说的

Researcher 9:19:24
Ok. Do you think there is a connection between "fun" and playability?
好的。您认为“乐趣”和游戏性之间存在关联吗？

Informant 007 9:19:51
I think there is an association.
我认为是存在关联的

Researcher 9:20:09
Can you speak more in detail?
能说得更详细一些吗？

Informant 007 9:21:56
Personally , I think if the playability of the game doesn't reach my expectation, there is no place for the fun. Similarly, I will search for the games which correspond to the fun I define. So, during the game design process, they must grab buyers' heart, don't they? The "fun" of the buyers must be reflected in the game.

我个人认为噢，单单针对我个人，如果这款游戏的游戏性不达到我的心里预期 谈何乐趣？同样的 我也会找符合我乐趣的游戏 所以在游戏设计过程中肯定要抓住购买者的心里吧？把购买者的“乐趣”在游戏中充分体现出

Informant 007 9:24:28
For example, the legend of Zelda, which was so popular at that time. Many people bought the console only for that game title, which was ridiculous at that time. But, this game did that, because the playability of this game was so great: exploring+decrypting+fighting+high visual quality, etc, which meet the fun that many people pursue.
好比当时风靡一时的塞尔达传说 好多人都是为了游戏买游戏机 在以前这个是多么的荒唐 然而一款游戏做到了 就是因为游戏本身的游戏性太棒了 探索+解密+战斗+画质等等 符合太多人追求的乐趣

Informant 007 9:24:59
I was also addicted to it.

我也沉迷在其中了

Researcher 9:25:54
Ok. So, after you perceive the fun of the game, you will want to buy games and their supporting hardware. Is that ture?
原来如此。所以在感受到这种游戏内的乐趣存在之后，会让您想买游戏和配套的硬件，是这样吗？

Informant 007 9:26:28
Yes.
是的

Researcher 9:27:16
:) I have a similar experience. I bought NGC just for the Resident Evil series.
：）我也有相似的经历。以前买 NGC 就是为了生化危机系列。

Researcher 9:27:21
Ok. So what do you think will happen when you feel that playing games is "fun"? For example, will you increase the gaming time?
原来如此。那么您感觉玩游戏有“乐趣”的时候，您还会有什么反应？比如说会增加游玩时间吗？

Informant 007 9:27:23
Everyone will have a scale in mind, and they will pay for what is worthwhile.
每个人心里都会有一杆秤的　认为值得的　都会为之买单

Informant 007 9:28:31
There may be some "naive" behaviours. . . Reluctant to clear the game.

可能会有一些“幼稚”行为。。。舍不得通关

Informant 007 9:29:17
Or delete the file after clearing the stages and start again.
或者通关后　删档　重新开搞。。。

Researcher 9:29:23
Ah, it's all over because the game is cleared. So, you will never perceive the fun again. Can I interpret like this?
啊，因为通关了游戏就结束了。所以就再也体会不到乐趣了，我能这么理解吗？

Informant 007 9:29:48
Yes, more or less. The feeling of Good to the last drop. You can understand my behaviours like this .

嗯　差不多吧　　意犹未尽　应该可以这么理解我的行为

Researcher 9:30:52
Combining the expansion package that we just mentioned, when you experience this "Good to the last drop" feeling, you will buy the expansion package if it is available, right?

结合刚才我们谈到了扩展包，在您体会到这种“意犹未尽”的感觉的时候，如果有新的扩展包，您就想要去购买，是吗？

Informant 007 9:31:17
Yes, I want to buy.
是的　我会去想要购买

Informant 007 9:32:44
As I have already recognised the playability of the game itself, I can buy the expansion package without worrying.
因为游戏本体的游戏性　已经让我得到了认可　我可以不用顾虑的去购买扩展包

Researcher 9:33:24
Ok. I would like to know whether the "worthwhile" means the equivalence of the fun that game contents generate and the price you pay?
原来如此。我想知道一下您刚才说的"认为值得"的意思是指购买游戏内容所获得的乐趣和所付的价格等价的意思吗？

Informant 007 9:33:56
Yes, it does.
嗯　是这个意思

Researcher 9:34:05
Ok. Let's change a topic, How do you usually purchase an expansion package type in-game goods? Please tell me a general process.
好的。我们换一个话题。您通常怎么样购买扩展包类游戏内购？　请告诉我一个一般流程。

Informant 007 9:34:57
There is a purchase link in the same page of the game on Steam.
steam 上　一般游戏本体下面就附有 DLC 购买链接

Informant 007 9:36:10
On eshop, the game name can be searched directly. After doing that, the process of purchasing DLC is as the same as the process of purchasing a whole game.
eshop 上　可以直接搜索游戏名称　会出现游戏及游戏 DLC　购买流程也都是和购买游戏本体流程一样

Researcher 9:37:59
Ok. We just talked about the platforms like steam and eshop. Do these platforms form a channel for you to know the in-game goods?
Ok。我们刚才谈到了 steam 和 eshop 这类平台，这些平台是您了解游戏内购的渠道吗？

Informant 007 9:39:07
Not all. I also know from channels such as official accounts on WeChat or Tieba.
也不全是吧，了解到得内购渠道也来自微信公众号或者贴吧

Informant 007 9:39:42
Also there are recommendations from friends, hahaha.
也有基友推荐，哈哈哈

Researcher 9:39:48
Ok. What sort of WeChat official accounts are we talking about here. Are they official(from the game company)?
原来如此。这边所说的微信公共号一般是什么样的公共号呢？是官方的吗？

Informant 007 9:40:59
They are not official. There are information pushing from mini app, and almost all of they are free messages.
不是官方，都是一些小程序推送，而且多数是免费推送。

Researcher 9:41:58
Ok. Then, during the process of purchasing in-game goods, will you actively search for more product information about these goods?
好的。那么您在购买游戏内购的过程中，会不会去主动搜索更多的关于游戏内购的产品信息？

Informant 007 9:44:01
More or less. I will search information such as what in-game goods add. Items? Maps? Characters? etc. Or what does it affect to the game itself after purchasing.
或多或少的都会去了解的 比如内购增加了什么 道具？地图？角色？等等 又或者说购买内购后会对游戏本体游玩时影响什么

Researcher 9:44:44
Ok. So which channels do you generally use to better understand these product information?
好的。那您一般是通过哪些渠道去更好地了解这些产品信息呢?

Informant 007 9:45:50
Baidu Tieba.
百度贴吧吧

Informant 007 9:46:04
I silently watch the big speakers speaking.
默默的看大佬们发言。。。

Researcher 9:46:34
Ok. The Tieba we are talking about refers to the Tieba with the same name as the game you play?
原来如此。这边所说的贴吧是和所玩游戏同名的贴吧吗？

Informant 007 9:47:10
Yes.
嗯 是的

Researcher 9:47:52
Ok. We just talk about the recommendations from friends. In general, what kind of friends will recommend you the information of in-game goods?
好的。我们刚才谈到基友推荐，一般是什么样的朋友会推荐给您游戏内购的信息呢？

Informant 007 9:49:08
Some are friends in the real life. We discuss and exchange information in the group.
有些是现实中的好朋友 会再群里讨论 信息分享

Informant 007 9:49:47
Some are virtual friends who I know online. They recommend information of in-game goods.
有会有一些虚拟的朋友 网上认识的 会推荐内购信息

Researcher 9:50:31
Ok. So, on which platforms do you meet with these online friends?
好的。那么和这些网上认识的朋友一般在哪些平台上交流呢？

Informant 007 9:51:06
Some will be in the game, some will be in WeChat.
有些会在游戏内 有些会在微信内

Informant 007 9:51:29
Now, most of them form groups on WeChat.
现在大多数都会组建微信群

Informant 007 9:51:34
The communication will be more easily.
沟通起来比较方便

Researcher 9:52:16
Ok.
好的。

Researcher 9:52:21
We continue. I noticed that in addition to the expansion package, you also purchase the Cosmetic/Skins. What is your motivation for purchasing Cosmetic/Skins type in-game goods?
我们继续。我留意到，除了扩展包类游戏内购，您还会购买装饰/皮肤。请问您购买装饰/皮肤类游戏内购买项目的动机是什么？

Informant 007 9:52:45
Of course, for the sake of handsome.

当然是为了帅啊

Informant 007 9:53:02
Although the ability is not good, but the handsome thing is a lifetime case.
能力不行　可帅是一辈子的事情啊

Researcher 9:53:14
What's the meaning of "handsome"?

"帅"的意思是？

Informant 007 9:53:24
Hahaha. A joke, a joke.

哈哈哈　开玩笑　开玩笑

Informant 007 9:53:43
In fact, it's just for good looking.
其实就是为了好看吧

Researcher 9:54:10
Apart from the good looking, is there any other reason?
除了好看之外，还有其它的原因吗？

Informant 007 9:54:52
Nope. The cosmetics are available for DOTA2 heros. Simply for good looking.
没了　DOTA2 英雄都可以购买饰品　单纯就是为了好看

Informant 007 9:55:09
These cosmetics will not enhance any ability. But I will buy them.
这些饰品也不会增加角色任何能力值　但是就是会去购买

Researcher 9:56:31
I see. When you purchase in-game goods, will you evaluate the alternative solutions of in-game purchase? For example, acquiring the same item in a free way?
原来如此。那么在游戏内商品的购买过程中，您是否经常评估可能的替代方案？比如用免费的方式去获取相同的游戏内购？

Informant 007 9:58:29
Yes, I will. But the reality is cruel. 0 investment is basically hopeless. If the alternative solution takes too long time and the requirement is too high, it is better to buy directly.
会去评估　但现实是残酷的　0 氪档基本无望　如果替代方案时间过长　要求过高　还不如直接购买

Researcher 9:59:25
I see. So, what difficulties do you usually encounter? Can you give some examples?
原理如此。那么一般会遇到哪些困难呢？能举一些例子吗？

Informant 007 10:01:25
For example, one mobile game is called One Piece: The road to the strong. The skin of character requires 100 pieces of debris, and there are 1~2 times of redemption activities every month. If you only count on ordinary tasks, you can only redeem up to 10 pieces.
比如手游里 海贼王强者之路　角色皮肤碎片需要 100 张碎片　每个月会有 1~2 次的兑换活动　如果单单做普通任务　你最多兑换 10 张

Informant 007 10:01:27
It takes too long time.
时间太长了

Informant 007 10:01:59
At the same time, if you redeem the debris, then the other resources cannot be redeemed. More harm than good.
同时　你如果兑换了碎片　那其他的资源就兑换不了了　得不偿失

Researcher 10:04:25
I see. Therefore, the high difficulty of obtaining the items through free methods is also one　reason why you buy the in-game goods, isn't it?
原来如此。所以，通过免费获取道具过高难度的难度也是您购买游戏内购的原因，是吗？

Informant 007 10:04:46
Yes.
是的

Researcher 10:05:32
Ok. We know there are different types of in-game goods, including Expansions and Cosmetic/Skins, as we just talked. When you buy in-game goods, do you have a priority in mind? For example, would you give priority to buying some types of product to another types of product?
好的。我们知道有不同类型的游戏内商品，比如我们刚才谈到的扩展包和装饰/皮肤。 当您购买游戏内商品时，您是否心里有一个优先顺序。比如比起一类游戏内商品您会优先购买另一类商品？

Informant 007 10:07:16
Can I ask a question? The in-game goods you mentioned refer to two difficult in-game packages in one game?
能提一个问题吗 你说的游戏内商品有限购买另一类商品中 另一类是指同一个游戏 2 个内购包？

Informant 007 10:07:51
If you mean two in-game packages in a single game, there is order.
如果是同一个游戏的 2 中内购包 那是会有优先顺序的

Researcher 10:08:07
Yes. Please continue.
嗯嗯，您继续说

Informant 007 10:09:09
For example, the topic we are talking today refer to expansions and cosmetics. If these two sorts of packages exist in one single game, then I will buy the expansion first and then buy the cosmetics.
比如我们今天讨论的这个话题 扩展和饰品 2 个内购包同时存在一个游戏 那我会优先购买扩展 然后再购买饰品

Researcher 10:09:50
I see. I get it.
原来如此。我懂了。

Researcher 10:09:57
These are all the questions. Thank you very much for participating in our research. Please confirm that your email address is XXXXXX@XXXXXX.com, because later we will send the JD electronic gift card to this address.
这就是全部的问题。 非常感谢您参与我们的研究。请确认您的电子邮件地址是 XXXXXX@XXXXXX.com，因为稍后我们把京东电子礼品卡发送到这个地址。
