# Supplementary material for: A grounded theory approach to understanding in-game goods purchase
Source: PLoS One. 2022 Jan 27;17(1):e0262998. doi: 10.1371/journal.pone.0262998 (PMC8794092; doi:10.1371/journal.pone.0262998)
Supplement: S1 File — (ZIP) [file pone.0262998.s001.zip › Transcript 7.pdf]

Interview: 007

Informant: 008

Please note that the original transcript is in Simplified Chinese. The English translation is for internal communication among the author of this research, and it is not proofread. Potential linguistic errors may exist in the English translation.

Researcher 14:01:44
Thank you for your willingness to participate and be interviewed here. My name is XXX XXX, and I'm a PhD student in the XXX University of XXX(XXX). Currently, I'm working on a research project which focuses on videogame players' purchase motivations of in-game goods. Throughout this interview, I will ask you a series of questions and you are encouraged to express your opinions freely with emoticons. If I have questions about what you've said or need clarification about a topic or concept, I'll ask you.
感谢您愿意参加并在此接受采访。 我叫 XXX，我是市场营销学的博士生，现在我在 XXX 大学就读。 目前，我正在开展一个研究项目，专注于电子游戏玩家对游戏内购买项目的购买动机。 在整个访谈中，我会问您一系列问题，我们鼓励您自由表达您的意见和观点。因为这不是一个当面访谈，所以我们也鼓励您用 QQ 表情来表达您的情绪。 在访谈过程中，如果我对你所说的内容有疑问或需要您澄清一个主题或概念，我会问您。

Researcher 14:01:57
Are you ready?
您准备好了吗？

Informant 008 14:02:29
Yes.
可以

Researcher 14:02:48
What's your family name?
请问您贵姓？

Informant 008 14:03:03
It's Zhang.
我姓张

Researcher 14:03:18
Ok, Ms. Zhang. In the previous survey, you mentioned that you purchased certain types of in-game purchases, including Loot boxes.
好的，张小姐。在之前的调查问卷中，您已经提到您购买了某些类型的游戏内购买项目,包括抽奖箱。

Researcher 14:03:23
What are your motivations for purchasing Loot boxes type in-game goods?
请问您购买抽奖箱类游戏内购买项目的动机是什么？

Informant 008 14:05:01
Because there are a lot of items inside and the Loot boxes are relatively easy to get. However, the items cannot be acquired through general lottery.
因为里面的道具比较多，抽奖箱 比较容易获得，你自己想要的，但是一般性抽奖 都抽不到的

Researcher 14:05:54
So there are two types of lottery boxes mentioned here, right? What does "general lottery" mean?
所以这边所说的抽奖箱有两类,是吗？ "一般的抽奖"是指？

Informant 008 14:07:54
It's like a scratch or big turntable. There is a probability. You must pump many times or reach a certain amount of money to.
如果像刮刮乐，还有大转盘，都是有概率的，一定要抽很多次，或者达到一定金额。

Informant 008 14:08:10
Acquire the items you want.
才能抽要想要的道具

Researcher 14:09:32
You just mentioned that the motivation for choosing to buy a loot boxes type in-game goods is to get items. Why do you choose to get items through the channel of loot boxes instead of buying items directly?
您刚才谈到选择购买抽奖箱类的游戏内购的动机是为了获得道具。请问您为什么会选择通过抽奖箱这种渠道获得道具而不是直接购买道具呢？

Informant 008 14:11:12
Because I could pay less and get more. It's a sort of gambling mentality.
是因为可以以小博大，这种赌博心理，双击查看原图

Informant 008 14:12:33
The cost of direct purchase is higher than the cost of the loot box.
而直接购买的话成本比抽奖箱的成本高

Researcher 14:12:53

It’s turn out to be the case😊.

原来如此：）

Informant 008 14:13:14
Yes.
是的

Researcher 14:13:18
We continue. So how do you usually buy the loot box type in-game goods? Please tell me a general process.
我们继续。那么您通常怎么样购买抽奖箱类的道具呢？ 请告诉我一个一般流程。

Informant 008 14:14:25
If it's a mobile game, then through the client. If it's a web page game, then through web links.
手游 直接有客户端就有的，网游是网页链接的

Researcher 14:15:19
Ok. So which channels do you generally use to better understand the product information?
好的。您一般如何了解到这类内购的信息呢？一般是通过哪些渠道？

Informant 008 14:17:41
If it’s a mobile game, generally there is a notice board at the login interface. If it’s a web page game, there are WeChat official accounts.
手游的话 一般会有登陆界面的公告，网页的话官方的页面 还有微信公众号

Researcher 14:19:03
I see. So, after you know the existence of these loot boxes items, will you search for more details?
原来如此。那么您在了解到了这些抽奖箱类道具的存在后，会不会去搜索它们更多的详细信息？

Informant 008 14:20:51
😎😎😎Yes, that for sure.

😎😎😎嗯，肯定啊

Informant 008 14:21:11
I would ask other people for the odds of their draws, to see whether the chance of winning is high or not.
问问看别人抽奖几率如何，中奖几率高不高

Researcher 14:21:22

Ok~

好的~ 。

Researcher 14:21:38
To what sort of "other people" are you referring here?
这边说的“别人”指的是什么样的人呢？

Informant 008 14:22:07
Players in the game.
游戏里的玩家

Informant 008 14:22:53

Firstly, I check for the winning chance of the local tycoons .

先看土豪抽奖几率如何

Researcher 14:23:06
Understood. The situation which you chat with those players occurs inside the game or outside the game?
了解。您和这些玩家聊这些内容的场景是发生在游戏内还是游戏外呢？

Informant 008 14:23:58
Most of them are outside the game. Nowadays, games may automatically create WeChat groups.
多数在游戏外，现在游戏都会自动创建微信群

Informant 008 14:24:08
However, there also are conversations inside the game.
不过游戏内也有交流

Researcher 14:24:33
Understood. We just talked about tycoon players. How do you define certain players as tycoon players?
明白了。我们刚才说到了土豪玩家，一般您怎么定义某些玩家为土豪呢?

Informant 008 14:25:08
V12 and V15 require charging a lot of money.
V12，V15 都大把大把得充钱

Informant 008 14:25:23
It’s scary.

吓人

Researcher 14:26:40
Are these other people's top-up records visible to everyone in the game you play? Or those tycoon players tell you how much he or she has charged?
这些别人充值的记录在您所玩的游戏内都是所有人可见的吗？还是土豪玩家自己告诉您他(她)充了多少？

Informant 008 14:27:23
It's visible. There is a VIP level avatar box.
可见的，会有 VIP 等级头像框。

Informant 008 14:27:59
Sometimes, they also show the trophy in the WeChat group.
有时微信群也会晒抽奖战利品

Researcher 14:28:26
Ok. So, the V12 and V15 you just mentioned are the proof of how much money a player recharges, right?
原来如此。所以您刚才所说的 V12，V15 都是某玩家充值真钱多少的证明，对吗？

Researcher 14:29:01
The more the real money is recharged, the higher the VIP rating. Can I understand like this?
充值真钱的数量越多，VIP 等级越高。我可以这样理解吗？

Informant 008 14:29:57
Yes. Approximately, 20000 RMB is required to achieve V12 and 150000 RMB is required to achieve V15 in Tencent games.
是的 腾讯 游戏 V12 大约充值是 2W 只有 V15 要 5W 以上

Informant 008 14:30:18
As I am playing Tencent games, I understand this.
因为我在玩腾讯游戏 所以了解的

Researcher 14:31:21
I see. Did you just talk about a specific game? Can you tell me its name?
原来如此。我们刚才说的是一个具体的游戏吗？能否告诉我一下它的名字？

Informant 008 14:31:50
For example, Luanshi Wangzhe and Red Alert OL.
比较 乱是王者 ，红警 OL

Researcher 14:32:01

Ok.
Ok。

Researcher 14:32:41
I see. When you purchase in-game goods, will you evaluate the alternative solutions of Loot boxes? For example, acquiring the same item in a free way?
在游戏内商品的购买过程中，您是否经常评估抽奖箱类游戏内商品的替代商品？比如使用免费的方式去获得抽奖箱？

Informant 008 14:36:50
Generally, they are not free to get. A lottery or recharge are required, because the Loot boxes are rare and not easy to acquire.
一般都不能免费获取的，都是需要抽奖，要么就是充钱，因为在抽奖箱的都是稀有的 不是怎么容易得到的

Researcher 14:38:45
Ok. One of the reasons we just mentioned buying a loot box is to get other in-game items. Would you evaluate the use of in-game mechanisms to get other items directly without buying a loot boxes?
好的。我们刚才谈到购买抽奖箱其中的一个目的是为了获得其它游戏内道具。您会不会评估使用游戏内机制来直接获取其它道具，而不购买抽奖箱呢？

Informant 008 14:40:36
Ummm...I see.
嗯...我想想

Researcher 14:41:13
Ok☕.

嗯嗯☕

Informant 008 14:41:16
I don' t understand what the game mechanics mean. Is it the daily task in the game?
主要没明白游戏机制是指什么，是指游戏内的日常任务吗？

Informant 008 14:41:40
Like completing a task to get items, right?
类似于达成任务，获取道具，这种？

Researcher 14:41:58
Yes, it refers to various gaming methods in the game. Getting items through tasks instead of spending money.
对，就是游戏内的各种游戏方式。通过任务等不花钱的方式来获得道具

Informant 008 14:44:03
Yes. But this method requires a lot of items. For example, 10 pieces of debris are needed to acquire a character, and 20 pieces of debris are needed to upgrade it, and 40 pieces of debris are needed to get one star. It is much slower if I rely on the usual tasks.
有也是有的，但是一般这种道具，肯定要很多的，好比一个角色，集齐 10 张碎片，升级要 20 张碎片，升一颗星要 40 张。靠平时任务来得比较慢，只能慢慢耗。

Informant 008 14:44:09
It's a pit.
比较坑

Researcher 14:44:30
Understood. It takes more time and energy, doesn't it?
明白了，比较花时间和精力，是吗？

Informant 008 14:44:41
Yes yes yes.
对对对

Informant 008 14:45:26
Sign in, 100 points must be achieved in daily tasks, or XX number of enemies must be defeat.
签到咯，每日任务达成必须 100 分，或者必须击败 XX 数量敌军

Researcher 14:46:39
Ok, I see. So do you think the high difficulty of obtaining items through free methods is a reason why you buy a loot box?
好的，原来如此。所以您认为过高得获取免费道具的难度是您购买抽奖箱的原因吗？

Informant 008 14:47:28
Yes.
嗯

Informant 008 14:48:17
There also are other reasons. Such as collecting the items and upgrading more quickly.
其他原因也有，也想快点集齐道具或者升级什么的

Researcher 14:50:23
Ah~ I see. So, can I say that the more difficult it is to get free items through the game mechanisms, the higher your intention is to buy a loot box?
啊~原来如此。那么，我能不能说，通过游戏机制来获取免费道具的难度越高，

您购买抽奖箱的意愿越高呢？

Informant 008 14:51:17
Yes, you can see that. Hahaha. But, I only do what I can.
可以这么说，哈哈哈。不过也量力而行的

Researcher 14:52:07
You mean the amount of money that is spent?

这边所说的量力而行指的是花费的金钱吧？

Informant 008 14:52:48

Yes. After all, it's a virtual world.

对啊， 毕竟虚拟世界

Researcher 14:53:35
Ok~. So, in your purchasing process, do you think the promotion affects you?
好的~。那么在您的购买过程中，您认为促销会不会对您产生影响？

Informant 008 14:53:37
Playing mobile games is a common pastime, and occasionally drawing with money is very cool.
玩手游也是平时消遣，偶尔充钱抽奖也挺爽的

Researcher 14:53:50
For example, there is a price cut for loot boxes.
比如抽奖箱降价了之类的。

Informant 008 14:54:24
Definitely, it should be. Girls always purchase during 11.11 and 6.18, don't they?
肯定呀 必须的 双十一 618 那个不是购买的都是女生

Researcher 14:56:40
Ok. Generally, how the promotion affects you? I mean, I want to buy when you see the promotion, or you want to realise the purchase at the first place, but the promotion determines your idea?
原来如此。一般促销对您的影响是怎么样的？我的意思是，您是看到促销才想买，还是本来就想买，但是看到促销更坚定了想买的念头？

Informant 008 14:58:25
Of course the idea is more determined after noticing the promotion. Hahaha.

当然是看到促销更加 必须坚定的的买了 哈哈哈双击查看原图

Researcher 14:59:11

Ok~~Backing to the topic you mentioned before. You just mentioned a quite nice feeling. Does the feeling come from the experience of having acquired items, or from a gambling-like experience?

好的~~回到之前您说的那个话题。您刚才提到的挺爽的感觉，这种感觉是更多地来源于获得道具的感觉呢，还是更多来源于类似赌博的体验呢？

Informant 008 15:00:32

That is not gambling, it is a lottery.

不是赌博，是抽奖

Researcher 15:01:06

Oh alright. I mean the uncertain pleasure that the lottery brings.

噢好吧。我的意思就是抽奖带来的不确定的愉悦感。

Informant 008 15:01:28
Both. The unknown surprise during the draw, and the excitement of getting rare items.
都比较倾向吧，抽奖时未知的惊喜，获得稀有道具的兴奋

Informant 008 15:01:31
Both.
都有

Researcher 15:02:12
Ok.
好的。

Researcher 15:02:15
These are all the questions. Thank you very much for participating in our research. Please confirm that your email address is XXXXXX@XXXXXX.com, because later we will send the JD electronic gift card to this address.
这就是全部的问题。 非常感谢您参与我们的研究。请确认您的电子邮件地址是XXXXXX@XXXXXX.com，因为稍后我们把京东电子礼品卡发送到这个地址。
