# Supplementary material for: A grounded theory approach to understanding in-game goods purchase
Source: PLoS One. 2022 Jan 27;17(1):e0262998. doi: 10.1371/journal.pone.0262998 (PMC8794092; doi:10.1371/journal.pone.0262998)
Supplement: S1 File — (ZIP) [file pone.0262998.s001.zip › Transcript 8.pdf]

Interview: 008

Informant: 009

Please note that the original transcript is in Simplified Chinese. The English translation is for internal communication among the author of this research, and it is not proofread. Potential linguistic errors may exist in the English translation.

Researcher 15:06:52
Thank you for your willingness to participate and be interviewed here. My name is XXX XXX, and I'm a PhD student in the XXX University of XXX(XXX). Currently, I'm working on a research project which focuses on videogame players' purchase motivations of in-game goods. Throughout this interview, I will ask you a series of questions and you are encouraged to express your opinions freely with emoticons. If I have questions about what you've said or need clarification about a topic or concept, I'll ask you.
感谢您愿意参加并在此接受采访。 我叫 XXX，我是市场营销学的博士生，现在我在 XXX 大学就读。 目前，我正在开展一个研究项目，专注于电子游戏玩家对游戏内购买项目的购买动机。 在整个访谈中，我会问您一系列问题，我们鼓励您自由表达您的意见和观点。因为这不是一个当面访谈，所以我们也鼓励您用 QQ 表情来表达您的情绪。 在访谈过程中，如果我对你所说的内容有疑问或需要您澄清一个主题或概念，我会问您。

Researcher 15:07:02
Are you ready?
您准备好了吗？

Informant 009 15:07:11
Yes, I'm ready
嗯嗯 准备好了

Researcher 15:07:23
What's your family name?
请问您贵姓？

Informant 009 15:07:32
It's Tang.
我姓唐

Researcher 15:07:46
Ok, Ms. Tang. In the previous survey, you mentioned that you purchased certain types of in-game purchases, including Power-ups, Playable characters, Cosmetic/Skins, and Loot boxes.
好的，唐小姐。在之前的调查问卷中，您已经提到您购买了某些类型的游戏内购买项目,包括增强道具，可游玩的角色，装饰/皮肤和抽奖箱。

Researcher 15:07:51
What are your motivations for purchasing Power-ups type in-game goods?
请问您购买增强道具游戏内购买项目的动机是什么？

Informant 009 15:08:39
I want to have a better gaming experience. Only by using Power-ups can I become stronger and win others.
想让自己有更好的游戏体验，只有增强道具，自己才能变的更强，打过别人

Researcher 15:09:30
Ok. Here, what does it mean to "win others"?
好的。在这里，“打过别人”具体是什么意思呢？

Informant 009 15:11:06
For example, the one I play most now is Luanshiwangzhe. There are many treasure heroes, hero skills, and the skin which enhance attacking ability. These items are not available without paying. I must enhance my attacking ability to win others.
比如我现在玩的最多的是乱世王者，它会有很多珍宝台英雄，英雄技能，加强攻击力的皮肤，这些道具不出钱没有的，要打过别人，自己要提升自己的攻击力

Informant 009 15:11:44
If you don't purchase those items, you can't win other people or being able to participate in group activities.
如果不买这些道具的话是打不过别人的 参与团体活动的

Researcher 15:11:51
That is to say, these kinds of items can only be purchased by using real money and can not be purchased by the in-game mechanism. Is this true?
也就是说这类道具只能通过用真钱购买，而不能通过游戏内部机制获取，是这样吗？

Informant 009 15:12:57
Right, you can only have them through recharging. The internal heroes of the game are very weak.
对的 只能通过充值才能有 它自己游戏内部机制的武将都很菜的

Researcher 15:13:39
It turned out to be the case. Can you please tell me how the "group activities" just mentioned are generally carried out?
原来如此。可否请您向我描述一下刚才所说的"团体活动"一般是怎么进行的？

Informant 009 15:14:27

Here is where the pit of this game locates. After purchasing a Treasure Warrior 3000, you still only get the character. You still need to complete tasks or recharge for acquiring the attributes and the levels. If you do not participate in an activity, you may be distanced from others😂.
这个游戏也坑在这里 买个珍宝台武将 3000 你还只是获得这个武将，属性，等级还是要靠你完成任务或者充钱，一个活动不参加 可能就会和别人产生距离😂

Researcher 15:15:22
Ah, that's it. That is to say, it is generally a multi-player activity, right?
啊，是这样。也就是说一般是多人的活动，对吗？

Informant 009 15:15:54
Group activities. For example, once a week there is a competition for the Imperial City, which is the confrontation between the League and the League. There is also Jiuding competition, which is the confrontation of each server area.
团体活动，比如：每周都会有一次争夺皇城，就是盟与盟之前的对抗。还有九鼎比赛，这个是每个服务区的对抗

Researcher 15:16:20
Ok, how many people participate inthis kind of activity?
好的，这样子的活动一般需要多少人一起参与？

Informant 009 15:17:53
Yes, of course, some people play "Stand-alone game". We call those people farmers. We play attacking with a lot of soldiers. When grain, wood, iron, and gems are not enough to be produced, at this time, we can buy them from "Stand-alone game" merchants.
对的 当然也有人玩“单机游戏”，那些人我们称作种田的，我们玩攻击的，兵多，粮草，木头，铁，宝石不够生产，这时候可以问“单机游戏”的商人购买

Informant 009 15:19:15
In terms of the Imperial City, since there is a restraint of the arms, we adjust according to the enemy's troops. We have Imperial City members, and everyone is online for standing by. Respect to the number of people, in general there are 20 people, waiting to take turns to send troops.
皇城的话，因为这个是有兵种克制的，所以根据敌对出兵情况进行调整，我们会有皇城队员，大家都在线待命。人数的话一般 20 个人，等着轮流上去出兵

Researcher 15:19:35
I think this concept is quite interesting. The "Stand-alone game" mentioned here refers to a built-in mode in the game, or is it a nickname between players?

我觉得这个概念蛮有意思的，这边所说的“单机游戏”指的是游戏里的一种内置的模式。还是玩家间的戏称呢？

Informant 009 15:19:49
In the case of Jiuding, there are generally 50 people participating in each league.
九鼎的话，一般一个盟 50 个人参与

Informant 009 15:20:54
It's nicknames among players. They specialise in providing materials. These materials are divided into protected resources and non-protected resources. Non-protected resources can be sold.
玩家间的昵称 他们专门提供物资，这些物资分保护资源和非保护资源，非保护资源可以卖的

Informant 009 15:21:57

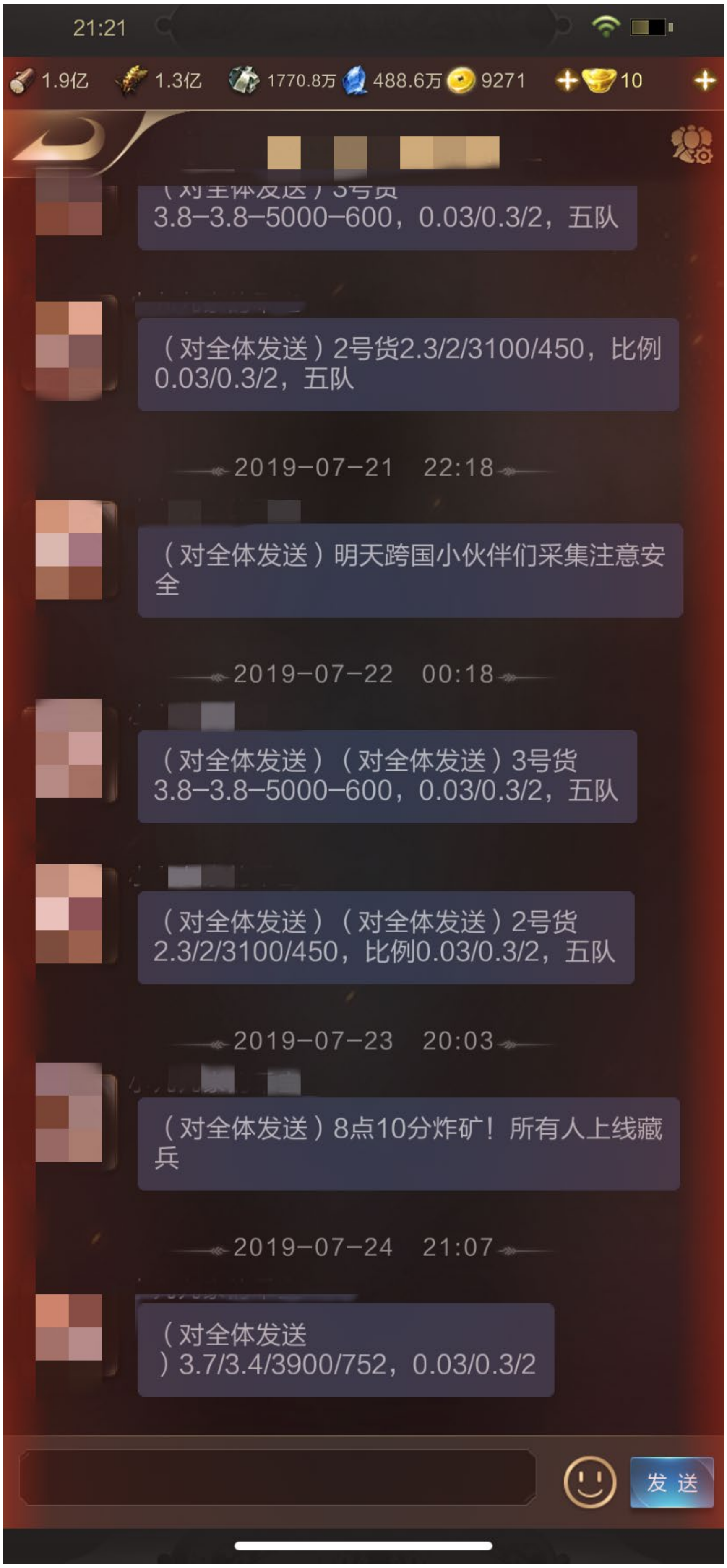
21:21
1.9亿
1.3亿
1770.8万
488.6万
9271
10
3.8–3.8–5000–600，0.03/0.3/2，五队
（对全体发送）2号货2.3/2/3100/450，比例0.03/0.3/2，五队
2019–07–21 22:18
（对全体发送）明天跨国小伙伴们采集注意安全
2019–07–22 00:18
（对全体发送）（对全体发送）3号货3.8–3.8–5000–600，0.03/0.3/2，五队
（对全体发送）（对全体发送）2号货2.3/2/3100/450，比例0.03/0.3/2，五队
2019–07–23 20:03
（对全体发送）8点10分炸矿！所有人上线藏兵
2019–07–24 21:07
（对全体发送）3.7/3.4/3900/752，0.03/0.3/2
发 送

Researcher 15:22:16
It turned out to be the case. So, why do these players only do these things? Will they participate in the activities you just mentioned?
原来如此。那为什么这些玩家只做这些事情呢?他们会参与您刚才所说的活动吗？

Informant 009 15:22:17
There are channels for selling zy in the league.
盟里有专门卖 zy 的渠道

Informant 009 15:23:29
They cannot participate in the large activities, because they can't beat anyone. They can participate Jiuding, in which the fighting power is distributed commonly, and the farming merchants can also participate.
大的活动不能参与，因为根本打不过别人，像九鼎可以参与，九鼎是分配相通战力的，他们种田的商人也能参加

Researcher 15:24:14
t turned out to be the case. The reason why they cannot participate is because they didn't buy Power-ups using RMB?
原来是这样。不能参与活动的原因是因为他们没有用人民币购买增强道具吗?

Informant 009 15:24:50
Yes. You cannot play without spending money, and you only can farm.
对滴，不花钱肯定不能玩，只能种田

Informant 009 15:25:18
In this game, who has the money is the King.
这个游戏就是谁有钱，才是王道

Informant 009 15:25:35
Tencent's games are basically like this
腾讯的游戏基本都这样

Researcher 15:26:13
I want to ask if "can not participate" means that the in-game mechanism does not allow players whose level of equipment is below a certain degree, or do other players not play with them, or do they feel that they are not able to participate in these activities?
我想追问一下这边"不能参与"的意思是游戏内机制不让装备等级不满某一个程度的玩家参与，还是其它的玩家不带他们玩，还是他们自己觉得根本没有能力参与这些活动呢？

Informant 009 15:26:33
They feel that their ability is not enough to participate.
自己觉得能力不够不参与

Informant 009 15:26:58
Because soldiers die when attacking the Imperial City. Egg touch hard stone means dead.
因为打皇城 要死兵的 鸡蛋碰硬石头只能死

Researcher 15:27:08
Ok. Have you ever been a farmer?
好的。您以前有当过种田商人吗？

Informant 009 15:27:13
Yes.
有

Researcher 15:27:38
是什么原因导致了您不当种田商人了呢？
Informant 009 15:29:51
I didn't want to pay for it at the beginning. My friend said that this game can make money and made me a merchant. I really didn't recharge for the first few months. However, you are a merchant and you are about to be bullied. While you are doing the collection, people are hitting you. You are not able to beat others, and you can only suffer. Later, we had a conflict with other league, and our entire league of farmers began to cultivate military commanders in order to flight for the vindication.
刚开始的时候没想充钱，我朋友说这个游戏能赚钱、让我当商人。前几个月我也真没怎么充。可你做商人要被欺负，你在采集，人家撞你，你还打不过人家，只能吃亏。后来跟人家盟杠上了，我们整个种田盟都开始培养武将了。为了打架争口气

Informant 009 15:30:48
There is a saying in China that people people live to strive for the vindication. Hahaha
中国有句俗语，人活着就为了争口气 哈哈哈

Researcher 15:31:12
I also have this experience when playing online games.

我在玩网络游戏的时候也有这样的经验。

Informant 009 15:31:33

Then I hooked up and began to slowly embark on the road of recharging money. Because this game is constantly updated, if you don't follow its pace, you have to be beaten behind.
然后就上钩了，开始慢慢走上充钱之路。因为这个游戏不断的更新，你不跟着它脚步，你就要落后挨打了

Informant 009 15:31:38

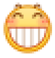

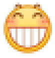

Researcher 15:32:30
We just mentioned that "not participating in an activity may cause a distance from others." Is the "other" mentioned here a friend in real life? Or the friends you know in the game?
我们刚才谈到"一个活动不参加 可能就会和别人产生距离", 这边所说的"别人"是现实中的朋友吗？还是游戏里认识的朋友？

Informant 009 15:34:22
In the game. Please think about it. Other people's attributes of military commanders are improving, and you don't improve them. Then what can you do? You can either recharge the money or Qiyou(Petrol) Ayyy~~ and the prize of Jiuding League (the whole server game) is really good. If the attributes of your military commanders are not good, then you are not eligible to participate.
游戏里的 你想啊，人家武将属性都在提高，你不提高，能干嘛，要么充钱要么汽油 哎～～而且，九鼎联赛（整个服务器的比赛）奖品很好的，你武将属性不好，也没资格参与的

Researcher 15:35:39
That's it. I would like to ask if the prizes of the Jiuding League can be obtained through RMB. Or it can only be obtained through in-game mechanisms?
是这样啊。我想问一下九鼎联赛的奖品能通过人民币获得吗？还是只能通过游戏内机制获取？

Informant 009 15:35:41
Just like the Jiuding League of this time: 120 Orange Pieces (in normal days they are only available after accumulated recharge) and 100 Dragon Searching Orders (120 gold coins)
像这次九鼎联赛，120 个橙色碎片（平时只有累计充值才会有）100 个寻龙令（120 个金币一个）

Informant 009 15:36:15

Yes, you can buy them, but the premise is that you have had a lot of money.

能买，前提要钱多

Researcher 15:37:00
It turned out to be the case. So do you think that the Luanshiwangzhe is a very competitive game?
原来如此。所以您认为乱世王者是一款对抗性很强的游戏吗？

Informant 009 15:37:11
Yes.
是的

Informant 009 15:37:12
Yes.
是的

Researcher 15:37:58
Ok. Let's change the topic. You mentioned in the questionnaire that you had purchased Cosmetic/skin in-game goods. What are your motivations for purchasing these items?
好的。我们换一个话题，您之前在问卷里也提到您有购买过装饰/皮肤类游戏内购。请问您购买这些道具的动机是什么呢？

Informant 009 15:38:23
There is also a little strategy, because you have to constantly adjust the battle attributes, and keep watch over the other people's arms.
也有一点点策略，因为你要不断的调整战斗属性，看别人出兵的兵种克制

Informant 009 15:38:36
The skin is for good looking.
皮肤为了好看

Researcher 15:38:55
Only this one reason?
就只有这一个原因吗？

Informant 009 15:39:03
For example, when playing pesticides(Wangzherongyao), I have to buy skin, and I feel that every hero must be beautiful.
像玩农药 就要买皮肤 感觉每个英雄都要美美的

Informant 009 15:39:37
也有属性的 乱世的皮肤又拉风，又有属性

Researcher 15:39:51
Why do every hero have to be beautiful?
为什么每个英雄都必须要美美的呢？

Informant 009 15:41:50
Because in the pesticide the operation is constant. There is the experience of visual effects, and skills and everything are related to the skin.
因为农药这个就一直在操作，有个视觉效果体验，放个技能什么的都跟皮肤有关

Informant 009 15:42:38
There are also dynamic skins, such as: An Qila, Sun Shangxiang
还有动态皮肤，比方说：安其拉，孙尚香

Researcher 15:43:35
But the purpose is to look good, right?
但是目的都是为了外观好看，对吗？

Informant 009 15:45:00
Yes, basically for good looking. Some skins also have attributes +10% life and magic power. It's better than have nothing.
对的，基本为了好看，皮肤也有属性+10% 生命 法强，有总比没的好

Researcher 15:45:11
Ok. I know.
Ok。了解了。

Researcher 15:45:16
We continue. We know that in many games, some characters need to be purchased with real money. What are your motivations for purchasing the Playable character type in-game goods?
我们继续。我们知道在很多游戏内，有一些人物都是需要用真钱购买的。您购买可游玩角色类游戏内购买项目的动机是什么呢？

Informant 009 15:46:43
To play better this game, and to follow the pace of the big troop. I can not be left behand, or I will Qiyou. If the rookie hinders, he is also very faceless.
更好的玩这个游戏，跟着大部队的步伐，不能脱队，不然就要汽油了，菜鸟拖后腿也很没面子的

Informant 009 15:47:04
Also a little vanity.
也有点虚荣心

Researcher 15:47:14
I just wanted to ask what "Qiyou" means.

我刚才一直想问"汽油"是什么意思

Informant 009 15:47:51
Qiyou isabandoning the game. We call it Qiyou.
汽油就是不玩了弃游 我们那边叫汽油

Informant 009 15:47:57
Haha
哈哈

Researcher 15:48:30
Is it a kind of feeling that, after a certain time of offline, you may have the feeling that you want to abandon the game?
是不是那种一段时间跟不上游戏内其它玩家的步伐，就有那种想要放弃的感觉？

Informant 009 15:49:06
Right, because once you can't keep up, when you go back to play, you need to spend more energy and recharge more money.
对的 因为你一旦跟不上了 再去玩 要花更多的精力，充更多的钱

Researcher 15:50:11
I see. I would like to know whether the degree of participation in game is related to saving money?
原来如此。我能问一下保持游戏内参与度和省钱有关系吗？

Researcher 15:50:38
Because you said if you don't play it, later you need to spend more money on it.
因为您之前说不去玩的话，之后再玩需要花更多的钱

Informant 009 15:50:48

In fact, it's a real deep pit. During the game playing, there are 20 persons belonging to the Imperial City team, who basically have recharged more than 10000.
其实真的蛮坑的，玩到后面我们盟里 20 个皇城队的，基本都充了 1 万+

Researcher 15:51:00
Are we talking about RMB?
这边说得是人民币吗?

Informant 009 15:51:07
Yes.
对的

Researcher 15:51:52
Ok. I have noticed that you also have purchased loot box type in-game goods. What are your motivations for purchasing such goods?
好的。我注意到您还购买过了抽奖箱类的游戏内购。请问您购买这类商品的动机是什么呢？

Informant 009 15:52:45
This game, if you do not want to play, then don't play anymore. Keeping up aging is difficult; you lose 2 orange pieces if you are not online every day. There are fixed activities every week. If you don't do them, then you are not rewarded. Later you can only buy them through recharging.
这个游戏，不玩就别再玩了，再跟上难，一样不上游戏，你就少 2 个橙色碎片，它每周都有固定活动的，不去做，就没奖励，你要这些奖励只能充钱买买买

Researcher 15:53:07
I see. I understand.
原来如此，我懂了。

Informant 009 15:54:28
Yes. Military commanders of Treasures are also a pit. They are pumped by Luck.
对的。珍宝台武将也很坑的，它凭运气抽的

Informant 009 15:54:31

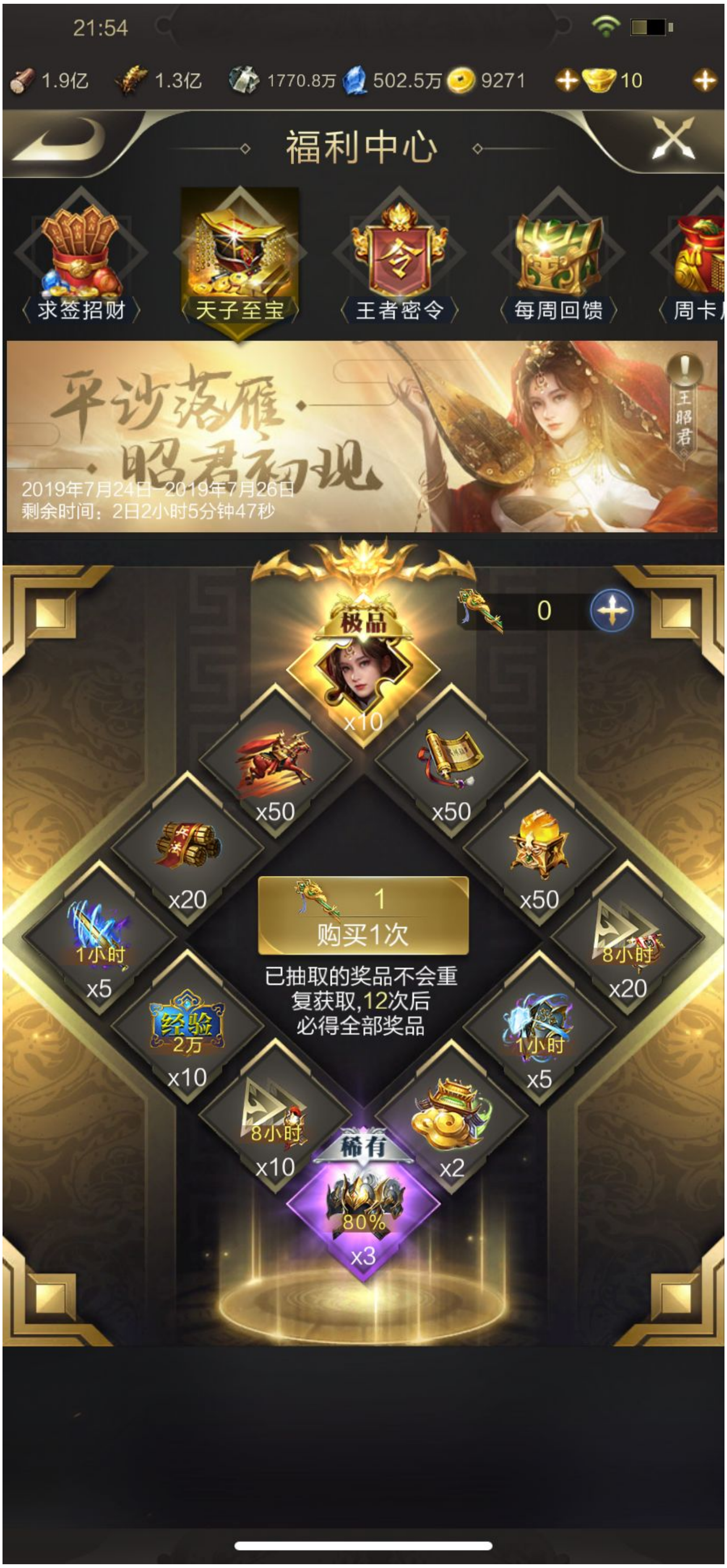
21:54
1.9亿
1.3亿
1770.8万
502.5万
9271
10
福利中心
求签招财
天子至宝
王者密令
每周回馈
平沙落雁·
·昭君初现
王昭君
2019年7月24日-2019年7月26日
剩余时间：2日2小时5分钟47秒
0
极品
x10
x50
x50
x20
x50
1
购买1次
1小时
x5
8小时
x20
已抽取的奖品不会重
复获取,12次后
必得全部奖品
经验
2万
x10
1小时
x5
8小时
x10
稀有
x2
80%
x3

Informant 009 15:55:23
This is a lottery. You need to draw for Wang Zhaojun.
这个就是抽奖形式之一，出了王昭君，你买一次抽一次

Researcher 15:55:33
Why do you choose to use the lloot box to acquire other items instead of buying them directly?
请问您为什么会选择通过抽奖箱这个方式去获取其它道具而不是选择直接购买道具呢？

Informant 009 15:55:59
Tencent doesn't allow you to buy these new heroes, that is, you need to participate in lucky draw.
腾讯就不给你买这些新出英雄，就是要你抽奖

Informant 009 15:56:01

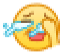

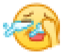

Researcher 15:56:28
So, is it possible to purchase out-fashioned heroes?
那过气的英雄能直接购买吗？

Informant 009 15:56:30
Basically, there are so many (items) above, and it's really good to draw the hero to the penultimate.
基本上上面那么多个，你抽到倒数第二个能抽到英雄已经很好了

Researcher 15:57:33
It turns out to be the case.
原来如此。

Researcher 15:57:53
We continue. So how do you usually buy the items we just talked about? Please tell me a general process.
我们继续。那么您通常怎么样购买我们刚才谈到的道具呢？ 请告诉我一个一般流程。

Informant 009 15:58:11
In terms of the out-fashioned heroes, it depends. I think no one wants these heroes by drawing using 3000 (RMB), they put them in regular draw pool, which doesn't

need 3000 (RMB). However, not many people would use these heroes. They are not useful,
过期的英雄 要看的 我觉得它是因为这些英雄没人肯花 3000 块抽，它就给你放平时武将台抽，这就不用 3000 了 但这英雄其实也没啥人用 用处不大

Researcher 15:58:50
I understand that it depends on the adjustment of the operation, is this the case?
我明白的，具体要看运营的情况调整，是这样吧？

Informant 009 15:59:16
Yes.
对的

Informant 009 16:00:52
Here, there are 2 ways to recharge. One way is buying using gold coin, and other way is one kind of ingot (gold coins can be dug, month card (68 yuan for one)), the ingot is recharging.
这边有 2 种购买方式，一种金币购买，一种元宝（金币可以通过挖，月卡（68 块钱一个））元宝就是充值了

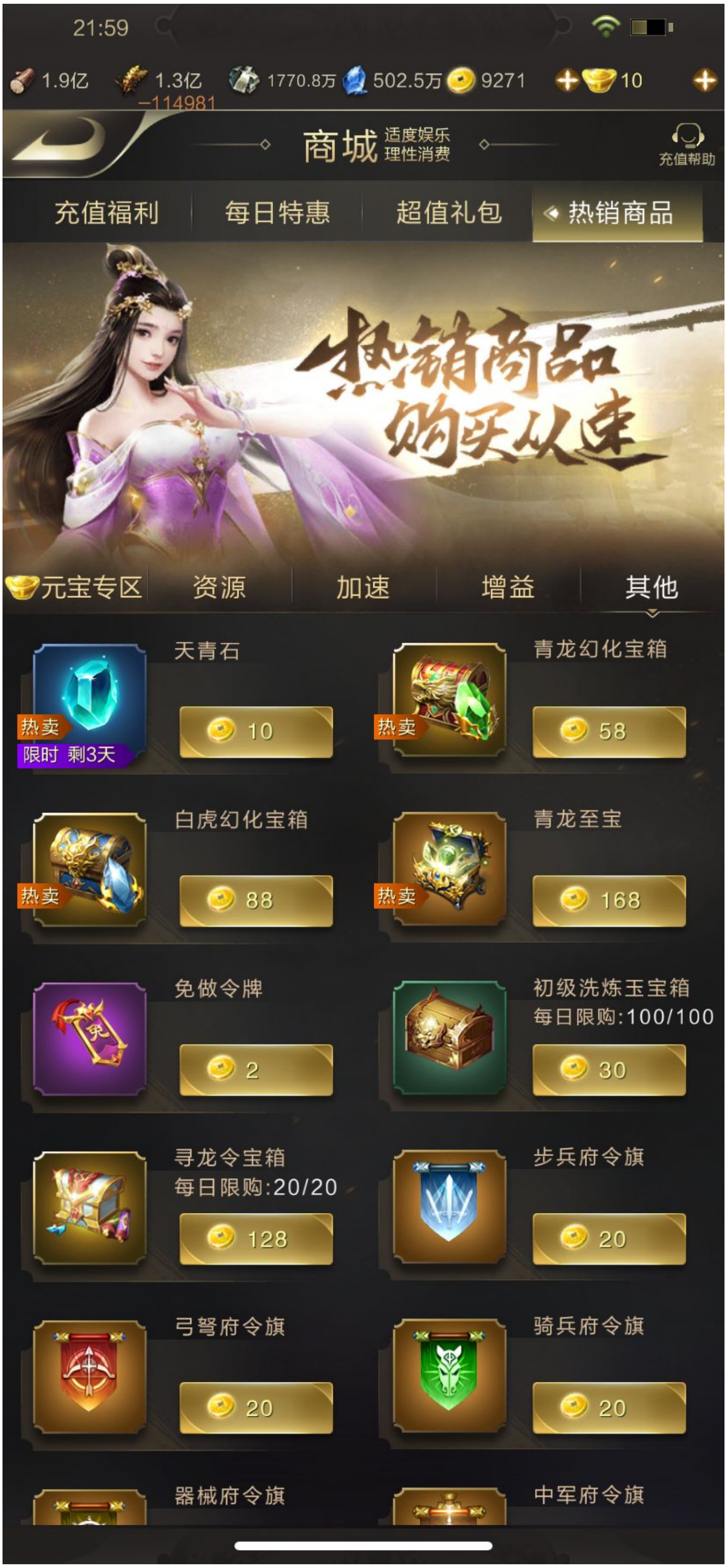
21:59
1.9亿
1.3亿
-114981
1770.8万
502.5万
9271
10
商城
适度娱乐
理性消费
充值帮助
充值福利
每日特惠
超值礼包
热销商品
热销商品
购买从速
元宝专区
资源
加速
增益
其他
天青石
热卖
限时 剩3天
10
青龙幻化宝箱
热卖
58
白虎幻化宝箱
热卖
88
青龙至宝
热卖
168
免做令牌
2
初级洗炼玉宝箱
每日限购:100/100
30
寻龙令宝箱
每日限购:20/20
128
步兵府令旗
20
弓弩府令旗
20
骑兵府令旗
20
器械府令旗
中军府令旗

Researcher 16:00:55
Ok. We went back to the question just now. How do you usually purchase the items we just talked about? Please tell me a general process.
ok。我们回到刚才那个问题。您通常怎么样购买我们刚才谈到的道具呢？ 请告诉我一个一般流程。

Researcher 16:01:49
Ok. I understand. Is the mobile payment usually used?
好的。我明白了。一般是通过手机移动支付吗？

Informant 009 16:02:33
My ingots are generally purchased by accumulative recharge. Every month there are activities of returning gold coins by consuming coins. At that time, it is most cost-effective to buy. Everyday there is a limited time task of consuming 300 gold coins. You can calculate things equal to 300 gold coins and sell them.
我元宝一般都是累计充值购买的，道具它每个月有消耗金币返金币活动，在那个时候买最合算。没天有消耗金币 300 的限时任务，你可以算好 300 金币的东西，买掉

Informant 009 16:02:42
Yes.
对的

Researcher 16:02:53
Ok. In general, from which channels did you learn about the existence of these in-game goods?
好的。一般您是从哪些渠道了解到这些游戏内购的存在的？

Informant 009 16:03:49
There are event reminders, and people in the league will send them., and little friends will tell you.
它会有活动提醒的，盟里也有人会发，小伙伴也会和你说

Informant 009 16:04:15

活动周历

活动开放受区服进度影响，最终开放情况以游戏内为主

日常活动　　大型活动

时间	活动内容					
7月22日 星期一	锦囊妙计	整军治国	王者赐福	盛世之治	——	——
7月23日 星期二	锦囊妙计	整军治国	王者赐福	制作神兵	兵甲升级	盛世之治
7月24日 星期三	整军治国	傲视群雄	制作神兵	兵甲升级	盛世之治	——
7月25日 星期四	整军治国	傲视群雄	制作神兵	兵甲升级	盛世之治	——
7月26日 星期五	整军治国		制作神兵		兵甲升级	
7月27日 星期六	名将谱		整军治国		全力冲刺	
7月28日 星期日	名将谱		整军治国		全力冲刺	

分享周历

温馨提示：
1.点击可查看每个活动详情
2.活动表每周刷新，排期仅供参考

Informant 009 16:04:25
This is a weekly event reminder.
这个是每周活动提醒

Researcher 16:04:50
Ok, the event reminder only functions in the game, right?
好的，活动提醒是游戏内才会提醒对吧？

Informant 009 16:05:20
Yes, in the game.
对的 游戏内

Informant 009 16:05:28

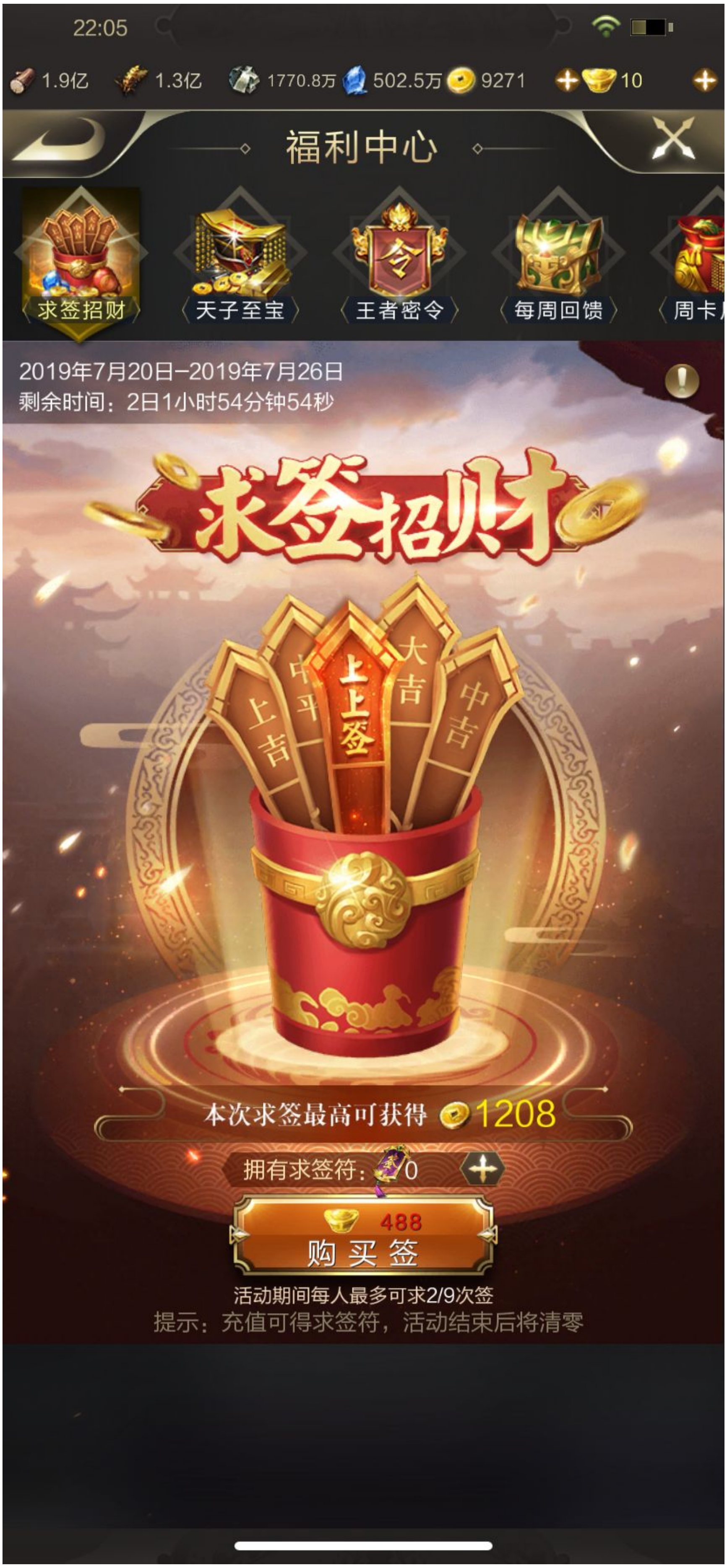
22:05
1.9亿
1.3亿
1770.8万
502.5万
9271
10
福利中心
求签招财
天子至宝
王者密令
每周回馈
2019年7月20日–2019年7月26日
剩余时间：2日1小时54分钟54秒
求签招财
上吉
中平
上上签
大吉
中吉
本次求签最高可获得 1208
拥有求签符：0
488
购买签
活动期间每人最多可求2/9次签
提示：充值可得求签符，活动结束后将清零

Informant 009 16:05:42
I basically look at the welfare center every day.
自己基本上会每天看福利中心

Researcher 16:05:49
Are the friends you are talking about here also friends in the game?
这边您说的小伙伴们也是游戏内的朋友吗？

Informant 009 16:05:59
Yes.
对的

Researcher 16:06:08
In what scenarios do you generally communicate? In the game?
一般是在哪些场景下沟通的呢？是在游戏内吗？

Informant 009 16:06:11
There is cp mode in the game, which reminds me.
游戏里有 cp 模式 cp 会提醒我的

Informant 009 16:06:19

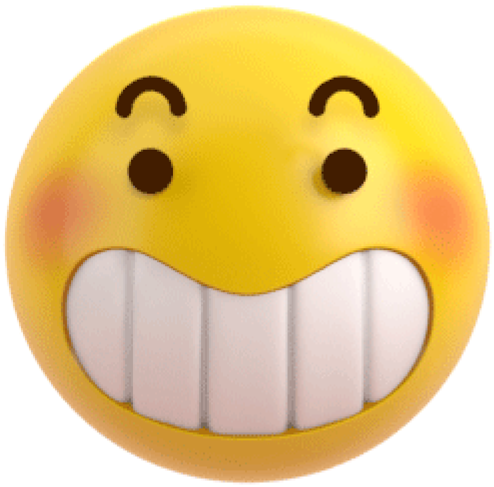

Informant 009 16:06:29
WeChat
微信

Researcher 16:06:54
I see. WeChat group?
原来如此。微信群吗？

Informant 009 16:07:02
I'm busy at work. It sends me screenshot when he notices that, or tells me that don't forget to do some activities.
我上班会比较忙，他看到了会截图我，或者告诉我啥啥啥活动别忘记做

Informant 009 16:07:24
We have a group of the game, and on cp we add WeChat (accounts), which sends me directly to WeChat.
我们有游戏群，cp 我们互加微信的 直接微信上发我

Researcher 16:07:37
I see. Is the communication in WeChat organized spontaneously by the players?
原来如此。这种微信里的沟通是玩家间自发组织的吗？

Informant 009 16:07:52
Yes.
对的

Informant 009 16:08:12
This game has a league (function) which bounds to the WeChat group.
这个游戏 有个盟绑定微信群的

Informant 009 16:08:36

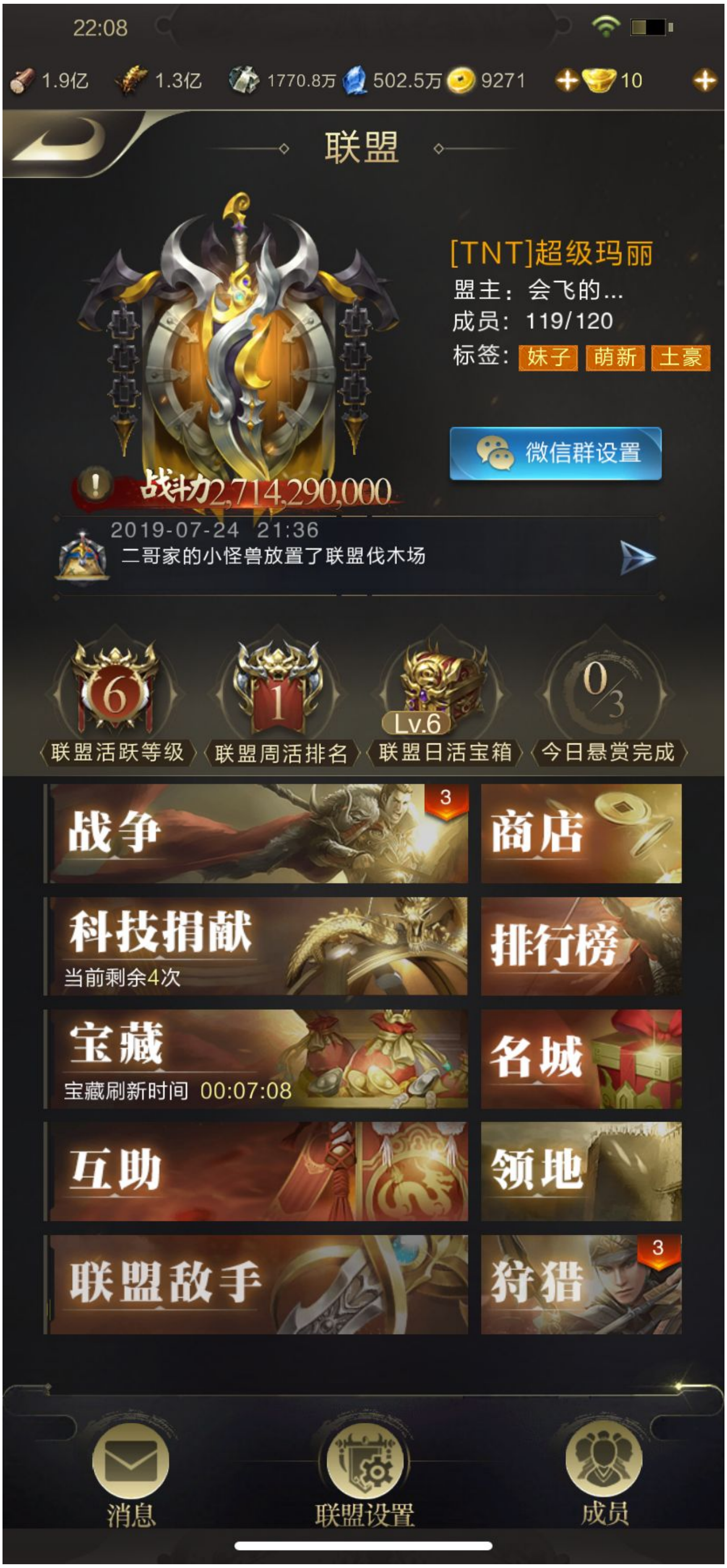
22:08
1.9亿 1.3亿 1770.8万 502.5万 9271 10
联盟
[TNT]超级玛丽
盟主：会飞的...
成员：119/120
标签：妹子 萌新 土豪
微信群设置
战斗力2,714,290,000
2019-07-24 21:36
二哥家的小怪兽放置了联盟伐木场
联盟活跃等级
联盟周活排名
Lv.6
联盟日活宝箱
今日悬赏完成
战争
商店
科技捐献
当前剩余4次
排行榜
宝藏
宝藏刷新时间 00:07:08
名城
互助
领地
联盟敌手
狩猎
消息
联盟设置
成员

Researcher 16:08:57
I see. There are mechanisms in the game which allows building easily WeChat groups, right?
原来如此。游戏内有机制能快速组建微信群，对吧？

Informant 009 16:08:59
Those who in the league can click the WeChat (button) to go to WeChat.
大家进盟的，可以点下微信 就跑微信里去了

Informant 009 16:09:04
Yes.
对的

Researcher 16:09:07
Oh. I see.
噢噢，懂了。

Informant 009 16:09:19
This function is quite good.
这个功能还是蛮好的

Researcher 16:09:48
I see. When you purchase in-game goods, will you evaluate the alternative solutions of in-game goods? For example, acquiring the same item in a free way?
我们继续。您在游戏内商品的购买过程中，您是否经常评估游戏内商品的替代方案？比如用免费的方式去获取道具？

Researcher 16:12:25
Excuse me, I am almost finished. It may take about 10-15 minutes more than expected.
不好意思，我快问完了。可能要比预计多花 10-15 分钟左右。

Informant 009 16:12:27
Yes, I should think about it. For example, the orange pieces, the flag, can be bought with money. I definitely don't want to spend money to buy them, as the pieces are gifted through accumulative recharging. While acquiring the ingot, I also can acquire pieces.
有啊 肯定要想的 比方说橙色碎片，令旗，可以用钱买的，我肯定不想花钱买啊，累计充值送碎片的，我有了元宝的同时也能拿碎片。

Informant 009 16:12:32
Don’t worry.

没事的

Researcher 16:12:41

Thank you very much.

太谢谢了。

Informant 009 16:12:45

Researcher 16:13:32
So you wanted to get the items through free methods, but why did you still realised the purchase?
所以您是想通过免费的渠道来获取道具的，但是为什么还是进行了消费呢？

Informant 009 16:14:07
Because of the cost-effective, which is actually a trick of Tencent. Sometimes you know it would be a trick but you get trapped.

因为划算　其实也是腾讯的套路，你明知是套路有时还是会被套

Informant 009 16:14:43
The debris is free, but my money was charged. I tell myself that the ingot can be used later.
碎片是免费获得了，但我钱也充了，我也告诉自己，元宝可以留着慢慢用

Researcher 16:15:07
What does the "cost-effective" mean here?

这边说的"划算"是指哪方面的划算呢？

Informant 009 16:17:49
Please think about it. You must recharge for the incogs, which serve to buy a lot of things. When a hero is released, the military commander package will be popping up, 298 ingots to buy. You can now get debris by recharging. For example, recharging 648 RMB and getting 20 debris, that's great.
你想啊，你元宝肯定要充的吧，元宝可以买很多东西的，你出了一个英雄，他有武将礼包弹出，298 元宝买。你现在充钱可以拿碎片，打个比方充 648 块钱，拿 20 个碎片，多好

Researcher 16:18:38
Ah, it means the promotion, right?
啊，是促销的意思，对吧？

Informant 009 16:18:47
Yes.
对的

Researcher 16:19:37
Ok. We know there are different types of in-game goods, including Power-ups, Playable characters, Cosmetic/Skins, and Loot boxes, as we just talked. When you buy in-game goods, do you have a priority in mind? For example, would you give priority to buying some types of product to another types of product?
好吧。我们知道有不同类型的游戏内商品，包括增强道具，可游玩的角色，装饰/皮肤和抽奖箱。 当您购买游戏内商品时，您是否心里有一个优先顺序。比如比起一类游戏内商品您会优先购买另一类商品？

Informant 009 16:23:11
Yes. In different games there are different choices. I also play Ro, which needs the comparasion: I need to purchase a staff first, or a pair of shoes, or a headgear. There is a trading platform, and you can put your own refined things on the trading platform in the game. I would check for the attributes, and I buy the items with good attributes. I usually buy weapons first.
会的，不同的游戏不同的选择。还玩 Ro，这个就个就要比了，我是要先买一个法杖呢，还是先出个鞋子，还是出头饰。这个会有交易平台，大家可以把自己精炼的东西放游戏里交易平台卖，会看下属性，觉得敲的属性好的东西会先买。一般先买武器

Researcher 16:23:57
These are all the questions. Thank you very much for participating in our research. Please confirm that your email address is XXXXXX@XXXXXX.com, because later we will send the JD electronic gift card to this address.
这就是全部的问题。 非常感谢您参与我们的研究。请确认您的电子邮件地址是XXXXXX@XXXXXX.com，因为稍后我们把京东电子礼品卡发送到这个地址。
