# Supplementary material for: A grounded theory approach to understanding in-game goods purchase
Source: PLoS One. 2022 Jan 27;17(1):e0262998. doi: 10.1371/journal.pone.0262998 (PMC8794092; doi:10.1371/journal.pone.0262998)
Supplement: S1 File — (ZIP) [file pone.0262998.s001.zip › Transcript 9.pdf]

Interview: 009

Informant: Informant 010

Please note that the original transcript is in Simplified Chinese. The English translation is for internal communication among the author of this research, and it is not proofread. Potential linguistic errors may exist in the English translation.

Researcher 9:15:20
Thank you for your willingness to participate and be interviewed here. My name is XXX XXX, and I'm a PhD student in the XXX University of XXX(XXX). Currently, I'm working on a research project which focuses on videogame players' purchase motivations of in-game goods. Throughout this interview, I will ask you a series of questions and you are encouraged to express your opinions freely with emoticons. If I have questions about what you've said or need clarification about a topic or concept, I'll ask you.
感谢您愿意参加并在此接受采访。 我叫 XXX，我是市场营销学的博士生，现在我在 XXX 大学就读。 目前，我正在开展一个研究项目，专注于电子游戏玩家对游戏内购买项目的购买动机。 在整个访谈中，我会问您一系列问题，我们鼓励您自由表达您的意见和观点。因为这不是一个当面访谈，所以我们也鼓励您用 QQ 表情来表达您的情绪。 在访谈过程中，如果我对你所说的内容有疑问或需要您澄清一个主题或概念，我会问您。

Researcher 9:15:25
Are you ready?
您准备好了吗？

Informant 010 9:15:45
Ok
OK

Researcher 9:15:50
What's your family name?
请问您贵姓？

Informant 010 9:16:10
It's Liu
姓刘

Researcher 9:16:24
Ok, Ms. Liu. Are you taking this interview at home?
好的刘小姐，您是在家里接受这个访谈吗？

Informant 010 9:16:31
Yes

是的

Researcher 9:16:48
Ok, In the previous survey, you mentioned that you purchased certain types of in-game purchases, including Power-ups, Expansion package, Playable characters, Cosmetic/Skins, and Loot boxes.
好的。在之前的调查问卷中，您已经提到您购买了某些类型的游戏内购买项目,包括增强道具，扩展包，可游玩的角色，装饰/皮肤，抽奖箱，以及省时道具。

Informant 010 9:17:01
Yes
是的

Researcher 9:17:05
What are your motivations for purchasing Power-ups type in-game goods?
您购买增强道具类游戏内购买项目的动机是什么？

Informant 010 9:17:25
To strengthen the attributes of roles.
加强角色属性

Informant 010 9:17:33
To increase the gaming experience
增加游戏体验

Informant 010 9:18:00
Also for contributing better during the league battle.
也是为了联盟战可以更好的出力

Researcher 9:18:34
Ok. I want to know what does enhancing gaming experience means to you.
好的。我想了解一下，对于您来说，增强游戏体验具体意味着什么？

Informant 010 9:19:18
Simply speaking, it's for having a good time; It's more pleasant when watching the battle report.
简单点说，就是玩的更爽，看到的战报更加赏心悦目

Informant 010 9:20:02
Kill more enemies.
杀敌杀的更多

Informant 010 9:20:13
Ranking can also go up.

排名也能上去

Researcher 9:21:21
Ok, I see. This is your mental state. So, will there be changes in the behaviour?
好的，原来如此。这是内心的感受。那么行为上会不会有变化呢？

Researcher 9:21:26
For example, the gaming time is increased?
比如游玩时间增加？

Informant 010 9:22:33
Yes, I will increase the gaming time.
会，增加时间。

Informant 010 9:23:04
Many missions require the Power-ups items.
很有任务都是需要道具加成的

Researcher 9:23:27
Ok. In addition, if you don't know how to answer a specific question, please let me know, I will give more explanations.
好的。另外，如果您对某个问题不知道怎么回答的话，请和我说，我会作出更多的解释。

Informant 010 9:23:38
The stronger gets the access to more stages.
实力最强才能闯更多关卡

Informant 010 9:23:52
Otherwise, some (people) cannot go on and stop in the middle way.
不然有的打了一半就玩不下去了

Informant 010 9:24:17
When being stuck in a stage for too long time, I would like to abandon the play.
有时候关卡卡太久，就会不想玩了

Informant 010 9:24:30
If the gaming is very smooth, I cannot stop.
玩的顺的话，就停不下来

Researcher 9:24:51
I see. When you cannot continue, how is your inner emotion or experience?
原来如此，在打不下去的时候，您内心的情绪或者体验是什么？

Informant 010 9:26:10
I can be very annoyed when I cannot continue, not happy.
打不下去的时候就会很烦躁，不开心

Informant 010 9:26:13
(Blow up)

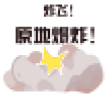

Informant 010 9:26:23
Sometimes, after failing several times,
有时候打了好几次不过

Informant 010 9:26:28
the mood will really blow up
心情真的会炸

Researcher 9:26:58
If there are Power-up items available at this time, would you buy them?
如果在这个时候有可供选择的增强道具，您会去买吗？

Informant 010 9:27:05
At this time I would buy the items.
这个时候就会买道具

Researcher 9:27:12
I see.
原来如此。

Informant 010 9:27:33
At this time, generally there are two options
这时候一般两种选择

Researcher 9:27:39
Please continue.
嗯，您说。

Informant 010 9:27:47
Buy the items or find a Big God.
买道具或者找大神带

Researcher 9:28:07
How can I under the concept of “Big God”?.
我怎么理解“大神”这个概念？

Informant 010 9:28:37
Some powerful (persons) in the game league.
游戏联盟里的特别厉害的

Informant 010 9:28:55
Sometimes he/she can lead five (persons)
有时候他可以一拖五

Researcher 9:29:34
Very powerful player, right?
很厉害的玩家，对吗？

Informant 010 9:29:37
If it's a single-player combat mission, I buy the items for myself.
单人作战任务就是自己买道具

Informant 010 9:29:45
If it’s a group (mission), then the Big God leads us.
团队的就是大神带，

Informant 010 9:29:47
Yes
对

Informant 010 9:29:54
Very powerful player.
很厉害的玩家

Researcher 9:30:44
I understand. On the other hand, you also talked about "If the gaming is very smooth, I cannot stop." In this case, how is your inner mood or experience?
我明白了。另外一方面，您也谈到了"玩的顺的话，就停不下来"。 在这种情况下，您内心的情绪或者体验是什么？

Informant 010 9:31:33
I would feel that I'm really good, and I want to do more missions and clear more stages at once.
会觉得自己很厉害，就会想一鼓作气的做掉更多的任务和关卡

Researcher 9:32:30
In this case, is your perception of time different from usual? Like feeling that time is getting fast.
在这种情况下，您对时间的感知会不会和平常有不一样的地方？比如觉得时间变得很快？

Informant 010 9:33:06

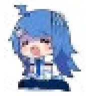

Informant 010 9:33:08
Time passed very fast. 2 hours passed during the blink of an eye.
时间过的非常快，2 个小时一眨眼就过去了

Informant 010 9:33:23
Sometimes, I plan to go to sleep after playing for an hour,
有时候想着玩一个小时就去睡觉

Informant 010 9:33:48
Then two hours passed unconsciously...and I told myself to play the last round. One more round...
然后不知不觉两个小时了...然后告诉自己在玩最后一盘。一盘就好.....

Researcher 9:34:19
I see Ok. Then, the experience that time becomes very fast appears when you are stuck in a stage?
原来如此。好的。那么这种时间变得很快的体验，在关卡在打不下去的时候，会不会出现？

Informant 010 9:34:57
In fact, when not playing very well, I also had the experience that time went very quickly.
其实打的不好的时候也会有时间过得快的体验

Informant 010 9:35:09
But the feeling is different to when I'm fighting smoothly.
但是和打的顺的时候感觉是一个不一样的

Researcher 9:35:35
主要是情绪上的区别，是吗？

Informant 010 9:35:40

Yes.
对

Informant 010 9:35:50
One is very cool, another one is very mourning.
一个很爽，一个很丧

Researcher 9:36:40
Ok. Then, in the case of "If the gaming is very smooth, I cannot stop”, would you buy in-game goods? Not necessarily the Power-up type in-game goods, here we are taking about all types of in-game goods.
Ok。那么在"玩的顺的话，就停不下来"的情况下，您会选择购买游戏内购吗？不一定是增强道具类型的，这边说的是所有类型的游戏内购。

Informant 010 9:37:02
I really like buying skins.
我很喜欢买皮肤

Informant 010 9:37:26
Regardless of the type of games.
不管啥类型的游戏

Informant 010 9:37:36
When releasing new skins or decorations,
出了好看的皮肤或者装饰品

Informant 010 9:37:43
I also want to buy.
我都会想买

Researcher 9:37:58
Why do you want to buy skin?
为什么想买皮肤呢?

Informant 010 9:38:16
Emmm...can I superficially say...Good looking?
emmm 能肤浅的说....好看么

Informant 010 9:38:21
(hahahahahahaha)

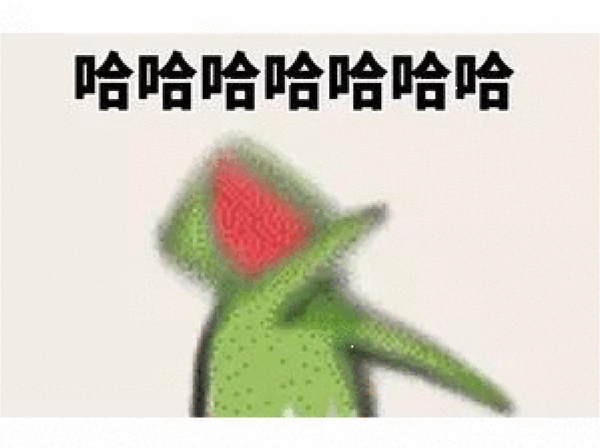

Informant 010 9:38:34
There are two types of skin in the game.
游戏里皮肤分两种

Informant 010 9:38:42
One with Power-up of attributes
一种有属性加强的

Informant 010 9:38:45
Another one is for good looking.
一种好看的

Informant 010 9:39:10
Moreover, when walking on the road, the character is different from others.
而且角色走在路上和别人不一样啊

Informant 010 9:39:16
I used to play RO.
我之前玩 ro

Researcher 9:39:30
Please continue.
恩恩，您说

Informant 010 9:39:53
(The producer/operator) introduced an ornament, which was actually useless, and it was good looking. You want what you want.
他出了装饰品，他的装饰品其实没啥大用处，就是好看，还不是你想要那个就那个

Informant 010 9:39:56
which required drawing.
需要抽的

Informant 010 9:40:03
Sometimes I got repeated (ornaments)
有时候还会抽到重复

Informant 010 9:40:17
In order to make them a whole set, I repeated drawing many times.
为了凑一套。我就抽了好多次

Informant 010 9:41:10
With the skin, it's cool when you go out setting up a wild team.
有了皮肤你出去组野队，也比较拉风吧

Informant 010 9:41:40
The skins of RO are for good looking.
ro 的皮肤为了好看

Informant 010 9:42:26
Another game that I'm playing, Luanshiwangzhe, relys more on attributes. One of the skins requires opening the Loot-box to acquire the coupon to redeem.
我玩的另一个款式王者的皮肤就真的是看属性了，他一个皮肤也是靠买箱子开券兑换

Researcher 9:42:39
I see. In other words, in some games, some skins can only be obtained through Loot-boxes, right?
原来如此，也就是说，在一些游戏里，有一些皮肤是只能通过抽奖箱获得，是吗？

Informant 010 9:42:46
Yes
对

Informant 010 9:42:59
Sometimes I can’t stop
有时候一抽停不下来

Informant 010 9:43:03

(Sad)

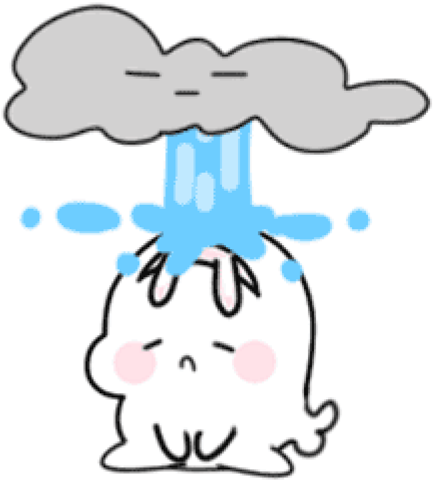

Researcher 9:43:11
So one of the reasons that you buy a loot box is to get items, right?
所以您购买抽奖箱的其中一个原因是为了获得道具，对吧？

Informant 010 9:43:21
(Yes, yes)

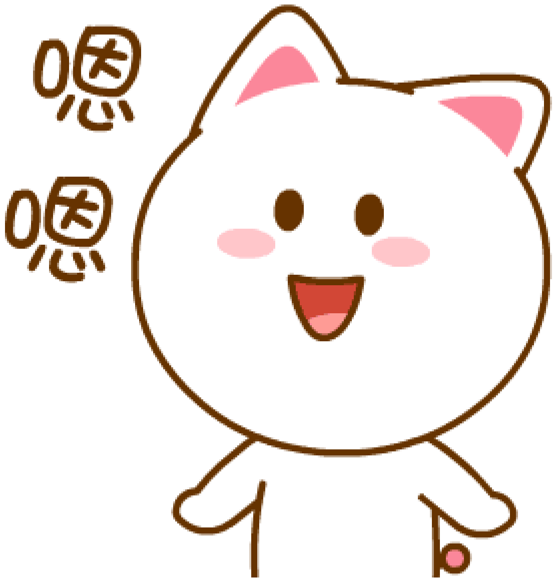

Researcher 9:43:28
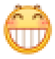 I see.

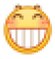 原来如此。

Informant 010 9:43:51
Emmm, in fact, sometimes buying a skin for the character is also to show off.
emmm，其实有时候买角色皮肤啥也是为了装逼吧

Researcher 9:44:07
We just said that you can't stop when drawing. How is your inner mood or experience in this circumstance?
我们刚才说到，抽得停不下来，请问在这种情况下，您内心的情绪或体验是怎么样的？

Informant 010 9:44:07
Because it looks so much better than without the skin.
因为看起来很就比没皮肤的厉害

Informant 010 9:44:49
I would be very unhappy if I always cannot get what I want or the repeat (skins).
要是一直抽不到想要的或者重复的就会很不开心

Informant 010 9:45:10
I will tell myself the game is a spam, which is a scam.
就会告诉自己垃圾游戏，骗钱的

Informant 010 9:45:23
I would be very happy when I get what I want.
抽到喜欢的就会很开心

Informant 010 9:45:39
(I will) quickly change it for that my little friends who play together with me can see it.
快点换上去给一起玩的小伙伴看

Informant 010 9:46:02
Seeing others having won what I want, I will be envious
看到别人抽到我想要的，就会很羡慕

Informant 010 9:46:14
I would think if I could try again.
就会想我要不要再去试一次

Informant 010 9:46:22
Probably I could win it.
没准就中了 ne

Researcher 9:46:56

I understand🌞.

我明白了🌞。

Researcher 9:47:26
We just talked about a lot of negative emotions. Would these negative emotions lead you wanting to give up the game?
我们刚才谈到了很多负面情绪的状况，这种负面情绪会不会导致您想放弃游戏？

Informant 010 9:47:39
Yes.
会

Researcher 9:48:15
Here are we talking about the intention of giving up? or have you given up before?
我们这边谈的是您有放弃的想法，还是之前有过放弃的行为？

Informant 010 9:48:25
I have given up before.
放弃过

Informant 010 9:48:58
But the game which I gave up before were more stand-alone or they do not have much interaction among players.
不过放弃的都是那些比较单机或者没什么玩家互动的游戏多一些

Researcher 9:49:11
Ok, what are the reasons for giving up?
好的，放弃的原因有哪些呢？

Informant 010 9:50:51
It doesn't make sense to play anymore. There are a lot of games nowadays, and many of them are similar. They let you to buy this or to buy that. You only spend a few minutes playing before they let you to the money for opening the stages.
感觉玩下去没意义了。现在很多游戏吧。雷同很多，还动不动就让你买这个买那个，你才玩了几分钟就让花钱开启关卡

Informant 010 9:51:11
In some very direct cases, you need to recharge some RMBs to buy a item, and they let you go on.

很直接的就是你充几块钱买个道具才让你下去

Informant 010 9:51:19
Then the game is very boring.
然后游戏又很无聊

Informant 010 9:52:01
Before, there was a very popular (game), called Mr love or what.
之间有个很火的什么恋什么

Informant 010 9:52:14
Mr Love: Queen's Choice (恋于制作人)
恋于制作人吧

Informant 010 9:52:16
Maybe.
好想

Informant 010 9:52:22
Yes, it is.
就是这样的

Informant 010 9:52:39
It's just like reading a novel book.
感觉就是在看小说书

Informant 010 9:53:24
There is also Star Dream(明星志愿). Every day doing the repetitive things.
还有一个明星志愿也是。每天都是重复的事情

Informant 010 9:53:34
Fortunately, you have to continue spending money to buy.
还好你一直花钱买

Researcher 9:53:42
Ok. So, when you have a boring experience, will you choose to buy in-game goods?
好的。那么在有无聊的体验的时候，您会不会选择购买游戏内购？

Informant 010 9:53:58
At the beginning I will.
一开始会

Informant 010 9:54:11
Later I feel boring.

后面就觉得没意思了

Informant 010 9:54:27
Every day repeating tasks and recharging money.
每天重复任务重复冲钱

Informant 010 9:54:46
I got tired of it and found new games.
玩腻了，找到新游戏了

Informant 010 9:55:01
If I haven't found new games, I will consider to buy some items appropriately.
没找到新的钱还会酌情买些道具

Researcher 9:55:32
Ok. I see. The reason why you bought the in-game goods at the beginning was that you thought the game was quite interesting at the beginning, right?
恩恩。原来如此。一开始会购买内购的原因是刚开始觉得这个游戏还蛮有趣的，对吗？

Researcher 9:55:55
Or you felt that it was very boring anyway, and you wonder if buying a in-game good could enhance the gaming experience?
还是觉得本来就很无聊，幻想着购买内购能提升一下游戏体验？

Informant 010 9:56:27
Both.
都会有

Researcher 9:56:54
I see. I just noticed a very interesting concept. You mentioned that the majority of the game you abandoned were stand-alone games. So, what are the reasons that prevent you from giving up online games?
原来如此。我刚才注意到了一个很有趣的概念。您提到放弃的以单机游戏居多，那么网络游戏有什么阻止您放弃的理由吗？

Informant 010 9:57:34
When you find that even after buying many Power-up items, but you still cannot catch up with the troop,
当你发现买再多道具和加强属性的道具也追不上大部队的时候

Informant 010 9:57:41
I began to want to give up.
就会想放弃了

Informant 010 9:58:00
Like the game I'm play now.
就像我现在玩的游戏

Informant 010 9:58:03
That's it.
就是这样

Informant 010 9:58:22
I feel that I'm in the bottleneck.
感觉瓶颈了

Informant 010 9:58:31
The attributes are not being able to come up.
属性都上不去了

Informant 010 9:58:42
(I) can't enter the main team.
进入不了主力队伍了

Informant 010 9:58:48
It is very mourning.
就会很丧

Researcher 9:59:17
What does the "main team" mean here?
请问这里说的"主力队伍"是什么意思？

Informant 010 9:59:18
Also (I'm) gradually considering whether to change a game.
也是在逐步考虑要不要换个游戏

Informant 010 10:00:32
The game we are playing now is called Luanshiwangzhe. It has collective activities almost every day. There are league or cross-regional activities. There are also some league tasks.
我们现在玩的游戏叫乱世王者。他几乎每天都有集体活动。会有联盟赛或者跨区赛。还有些联盟任务

Informant 010 10:01:01
The main team can represent the league to fight against other league.
主力队就是可以代表联盟去和别的联盟对战的

Informant 010 10:01:40
Even the most powerful ones can compete with (the leagues in) other servers.
甚至最厉害的一些还能和别的区一起比赛

Researcher 10:02:07
I understand. They are hardcore players and often participate in large in-game activities, is that the case?
我明白了。他们是比较硬核的玩家，经常参加游戏内大型活动，是这样吗？

Informant 010 10:02:27
The game always introduce cross-server competitions. Only the most powerful (players) can represent the league to fight.
他一直会搞跨区比赛。只有最厉害的几个才能给联盟出塞

Informant 010 10:02:30
Yes.
对

Researcher 10:03:23
A former interviewee also talked about the game, Luanshiwangzhe, she mentioned the existence of players like "farming merchants" in the game, right?
之前一位受访者也谈到了乱世王者这款游戏，她提到在游戏内有"种田商人"这类玩家的存在，对吗？

Informant 010 10:03:51
Yes, haha, I originally wanted to be a farmer. Finally, I failed.
对，哈哈我本来也是想去做种田商人的。最后失败了

Informant 010 10:04:12
Always being robbed or beaten by combat players
一直会被战斗玩家打劫或者打

Informant 010 10:04:35
Then when I got angry, I started to become a combatant.
然后一生气就也开始变成战斗号了

Researcher 10:04:58
In other words, the gaming experience is different, right?
也就是说他们的游戏体验是不同的，是吗？

Informant 010 10:05:03
Yes.
对

Informant 010 10:05:22
Farming merchants' gaming method is being beaten...being robbed.
种田商人的玩的方式就是挨打...被打劫...

Informant 010 10:05:33
It's just like QQ farm.
有种类似 qq 农场

Researcher 10:05:53
Then, from your point of view, why they decide to become farming merchants in the first place.
那您觉得为什么他们一开始要当种田商人呢？

Informant 010 10:06:03
We sometimes need to upgrade the building and buy resources from the farming merchants.
我们有时候需要升级建筑，会从种田商人哪里购买资源

Informant 010 10:06:25
Buying resources using RMB.
用人民币购买资源

Informant 010 10:06:51
The production of the resources in the account of combatant is not enough in the game.
战斗号的资源自己游戏里产出是远远不够的

Researcher 10:07:35
I see. I understand. Let's change a topic. Generally, from which channels do you know the existence of in-game goods?
原来如此，我明白了。我们换一个话题。您一般从哪些渠道了解到游戏内购的存在呢？

Informant 010 10:07:37
Many merchants have 4-5 accounts. They collect resources and sell them to us.
很多商人都是一个人 4-5 个号。采集资源然后出售给我们

Informant 010 10:07:56
WeChat accounts.
游戏微信公众号

Informant 010 10:08:03
And announcements in the game.

还有游戏公告

Informant 010 10:08:31
Sometimes the game butlers tell the hardcore players of the league.
有时候盟里的硬核玩家的游戏管家告诉他们后

Informant 010 10:08:35
They(hardcore players) would tell us.
他们也会告诉我们

Researcher 10:08:52
So, after learning about the existence of these in-game goods, will you use some channels to better understand the information of these products?
那么在了解到这些游戏内购的存在后，您会不会通过一些渠道去更好地了解这些商品的信息?

Informant 010 10:09:04
I would review the game circle.
会看游戏圈

Researcher 10:09:20
What does the game circle mean?
游戏圈指的是？

Informant 010 10:11:29
Player discussion area.
玩家讨论区

Researcher 10:12:01
Is this type of discussion area inside the game?
这类讨论区是在游戏内部的吗？

Informant 010 10:12:32

中国移动 16:12 10%

乱世王者 游戏圈

辱我一个项羽吊打了我赵云曹丕？这游戏还能玩吗？全…

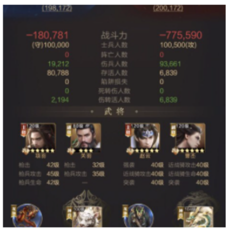
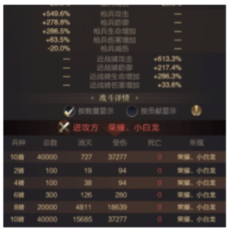
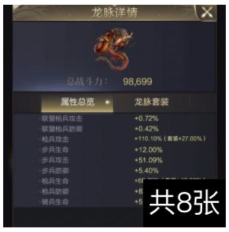
共8张

大鱼 30分钟前 41 585

弓兵还能玩吗？讨论下

核心:讨论下经过监军府，神兽幻化，注灵等系统推出后，兵种生命防御数值大幅度提升后，弓兵的输出到底在哪…

盐湖区、梁朝伟 6分钟前 8 42

穆桂英配甄宓最合适（别沉了顶上去）

穆桂英加甄宓首选 她最合适 华佗貂蝉 张角大小姐 步兵 袁绍董卓 弓兵 两个调换也行 辅助高的 就剩下甄宓 出…

布偶猫 7小时前 20 72

发表

新区集结号

召集一起进新区的大兄弟，大佬带队，要去的私

Informant 010 10:12:34

乱世王者 游戏圈

置顶 2019燃情起航丨新的征程，这乱世因你而不一样

活动 **【话题】最强王国，你的上分方法是？**

要说什么活动奖励丰富，肯定少不了我们的最强王国玩法，最强王国是以国家为单位，通过一系列的活动抢占排…

橙子好吃吗 昨天 6 48

项羽的孙子，近战叫爷爷

你项羽强我知道！骑兵打不过枪我知道，但你不能这样羞辱我一个项羽吊打了我赵云曹丕？这游戏还能玩吗？全…

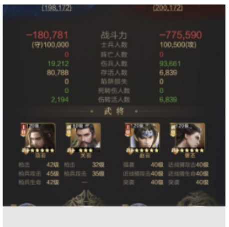

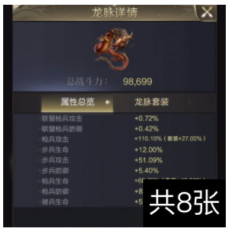

大鱼 45分钟前 37 553

全服送行，愿天堂安好。

仍然记得老段带着几个朋友入会时的情景，后来老段的爱人段嫂也加入了乱世。那个我们每天谈天说地都能几千…

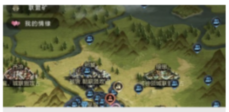

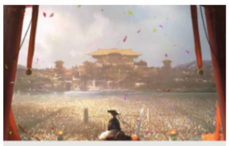

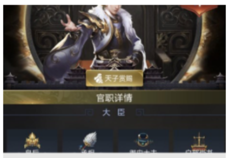

尖尖(∶ 0 っ)=周颖 1分钟前 10[illegible] [illegible]

发表

Informant 010 10:12:37

每个兵种，算上专业或者半专业武将，第四个橙将往往是三十块：弓兵，黄忠（专业），周瑜（专业），姜维（半专业）…

步兵炮 10分钟前 4 23

朱雀第三属性什么时候改成弓弩统一

感觉玩弓的受到了侮辱，骑车神兽第三属性都是双兵种通用的你连白虎第三属性都针对弓弩双兵种。为啥朱雀第三属性就…

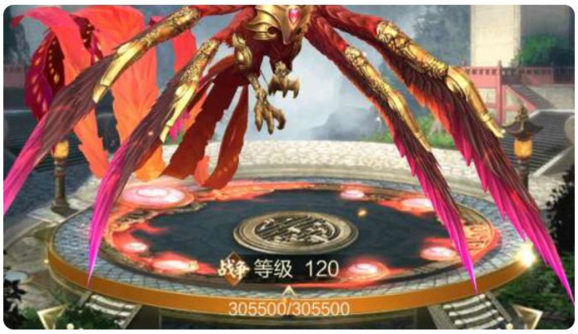

我为自己代言 10分钟前 14 57

乱世王者微信2区 热血梦 联盟 梦丶云飞 为乱世王者2周年庆典献礼

西风怒号乱飞花 将军喋血映夕霞 壮士英魂归故里 霸王挺戟又出发 无论春秋冬夏，或是坎坷泥洼&nbs

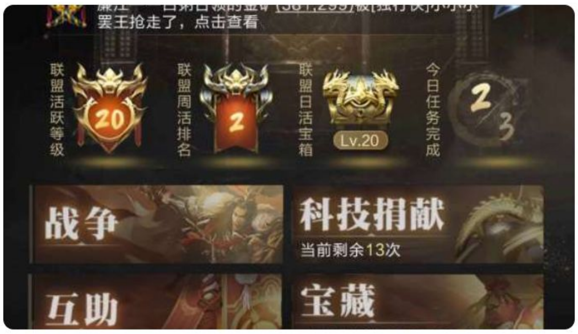

发表

演员จุ๊บ王云飞 12分钟前 11 28

Informant 010 10:12:39
It can be opened in WeChat.
微信里可以打开

Informant 010 10:12:40

微信游戏圈

微信游戏圈

发现游戏新鲜事

讨论

置顶 2019燃情起航丨新的征程，这乱世因你而不一样

【话题互动】看主公您联盟里的那些喜怒哀乐～

不知不觉间，《乱世王者》快要陪伴主公们走过两年的风风雨雨啦！在这两年的时间里，亲爱的主公们都是如何在战火纷…

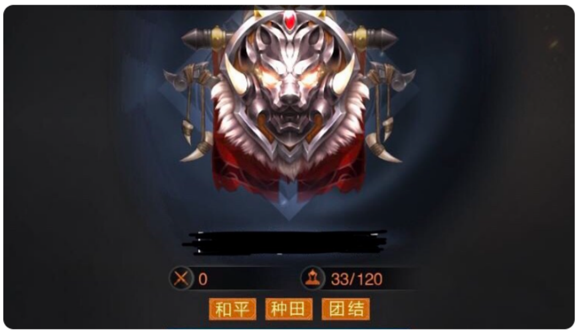

橙子好吃吗 2小时前 4 25

暴击盾说法成立嘛

曹操关羽 或者司马关羽 技能是盾生 盾墙 盾攻 龙脉盾八件套传承主盾攻击步兵攻击血量 输出来源是关羽提供的暴击 那…

李子豪 16分钟前 44 210

项羽的孙子，近战叫爷爷

你项羽强我知道！骑兵打不过枪我知道，但你不能这样羞辱我

发表

返回游戏

Informant 010 10:12:42
Also in the game.
游戏里也可以

Informant 010 10:13:09
There will be a lot of new game information inside.
里面会有很多新的游戏信息

Researcher 10:13:12

Ok, I understand, thank you for the screenshot, very intuitive .

Ok 我明白了，谢谢您提供的截图，很直观

Informant 010 10:13:30
Or some tests other (players) do in the game.
或者一些游戏里的大家的测试

Informant 010 10:14:00
There will be some battle reports done by big god level players.
会有一些大神级别的战报

Researcher 10:14:29
I understand. Sorry, the interview may have to be extended for a little while, but I am almost finished. Very sorry. . .
我了解了。不好意思，访谈可能要延长一小会儿，但是我快问完了。非常不好意思。。。

Informant 010 10:14:39
No.
没事

Researcher 10:14:52
Thank you very much~

太谢谢了~

10:14:55 你撤回了一条消息

Researcher 10:15:08
I see. When you purchase in-game goods, will you evaluate the alternative solutions of in-game goods? For example, acquiring the same item in a free way?

我们继续。您在游戏内商品的购买过程中，您是否经常评估游戏内商品的替代方案？比如用免费的方式去获取道具？

Informant 010 10:15:43
I just mentioned that in our game, there are many league missions.
我刚刚有说到我们那个游戏有很多联盟任务。

Informant 010 10:16:32
Sometimes the Ranking prices or cross-country rewards are the items which usually require a lot of money to buy.
他有时候联盟排名奖励或者跨国赛奖励的奖品就是平时要花很多钱才能买的道具

Informant 010 10:17:26
There are also ways, emmm, that may not be quite good for game planning.
还有些方式，emmm，可能对于游戏策划来说。比较不好

Informant 010 10:18:00
That is, we buy some resources from the farming players. Their resources are much cheaper than purchasing directly in the game.
就是我们会从种田玩家哪里购买一些资源，他们的资源会比直接游戏里购买便宜很多

Researcher 10:18:43
Ok. What factors prevent you from acquiring items in a free way?
好的。请问有哪些因素会阻碍您通过免费的方式获取道具呢？

Informant 010 10:19:32
There is also skin, the skin can be acquired using the boxes in the game. Some (players) acquire them very quickly through drawing as they got good luck. Some may take 5-6000 to get a skin. However, it only costs 1-2000 buying from farming merchants.
还有皮肤也是，皮肤是有一定的几率用游戏送的箱子开出来，有的人运气好。很快开出来，有的话可能要花 5-6000 才一个皮肤，然后从。种田商人哪里可能才 1-2000

Informant 010 10:19:55
Time, many tasks take a lot of time.
时间吧，很多任务非常的花费时间

Informant 010 10:20:17
Sometimes you haven't done the task in a single day or just forget to calculate the time.

有时候你一天没去做这个任务或者只是忘记计算好时间

Informant 010 10:20:23
Then the ranking is gone.
然后排名就没了

Informant 010 10:20:40
If you want, you can only pay for it yourself.
想要的话就只能自己花钱买

Researcher 10:22:20
These are all the questions. Thank you very much for participating in our research. Please confirm that your email address is XXXXXX@XXXXXX.com, because later we will send the JD electronic gift card to this address.
这就是全部的问题。 非常感谢您参与我们的研究。请确认您的电子邮件地址是XXXXXX@XXXXXX.com，因为稍后我们把京东电子礼品卡发送到这个地址。
